# Design, Synthesis, In Vitro, and In Silico Studies of 5‐(Diethylamino)‐2‐Formylphenyl Naphthalene‐2‐Sulfonate Based Thiosemicarbazones as Potent Anti‐Alzheimer Agents

**DOI:** 10.1002/ardp.70050

**Published:** 2025-07-20

**Authors:** Urva Farooq, Muhammad Islam, Zahra Batool, Suraj N. Mali, Rahul D. Jawarkar, Shailesh S. Gurav, Rima D. Alharthy, Halil Şenol, Nastaran Sadeghian, Parham Taslimi, Zahid Shafiq, Silvia Schenone

**Affiliations:** ^1^ Institute of Chemical Sciences Bahauddin Zakariya University Multan Pakistan; ^2^ Department of Basic Sciences and Humanities (Chemistry) Muhammad Nawaz Sharif University of Engineering and Technology (MNSUET) Multan Pakistan; ^3^ School of Pharmaceutical Science and Technology Tianjin University Tianjin China; ^4^ School of Pharmacy D.Y. Patil University (Deemed to be University) Navi Mumbai India; ^5^ Department of Medicinal Chemistry, Dr. Rajendra Gode Institute of Pharmacy University‐Mardi Road Amravati India; ^6^ Department of Chemistry VIVA College Virar (W) Maharashtra India; ^7^ Department of Chemistry, Science & Arts College, Rabigh Branch King Abdulaziz University Rabigh Saudi Arabia; ^8^ Department of Pharmaceutical Chemistry, Faculty of Pharmacy Bezmialem Vakif University Fatih Istanbul Türkiye; ^9^ Department of Biotechnology, Faculty of Science Bartin University Bartin Türkiye; ^10^ Department of Pharmacy University of Genoa Genoa Italy

**Keywords:** anti‐Alzheimer, MD simulation, molecular docking, naphthalene‐2‐sulfonate, thiosemicarbazones

## Abstract

Alzheimer's disease (AD) is known as one of the more devastating neurodegenerative diseases diagnosed in older people. Cholinesterase inhibitors (ChEI) can be used as an effective palliative treatment for AD. An extensive range of new biologically active 4‐(diethylamino) salicylaldehyde‐based thiosemicarbazone derivatives **5(a–u)** was synthesized and evaluated as inhibitors of cholinesterase (ChE) and monoamine oxidase (MAO) enzymes. 2,3‐Dichloro‐substituted compound **5u** was the most potent inhibitor of AChE and MAO‐A with IC_50_ values of 12.89 and 96.25 nM, respectively. In contrast, the 2,3‐dichlorophenyl‐substituted compound **5a** was the most powerful inhibitor of BChE, with an IC_50_ value of 124.72 nM. Structure–activity analysis revealed that the electron‐withdrawing substituents on the phenyl ring play a crucial role in the inhibition potential of synthesized compounds. Compound **5a** showed the strongest binding with 4BDS (−11.3 kcal/mol) via hydrogen bonds and π‐interactions. Compound **5u** exhibited high affinity with 1B41 (−8.2 kcal/mol), 2Z5X (−8.6 kcal/mol), and 2V5Z (−7.8 kcal/mol), forming key hydrogen bonds, salt bridges, and π‐interactions, highlighting its multi‐target potential. In silico ADME, pharmacokinetics, and drug‐likeness studies were conducted and compared with the standard drugs galantamine and clorgyline.

## Graphical abstract

A novel series of 4‐(diethylamino)salicylaldehyde‐based thiosemicarbazone derivatives **5(a–u)** were synthesized and screened for their potential against Alzheimer's disease, by testing for their inhibitory effects against cholinesterases (AChE and BChE) and monoaminoxidase A (MAO‐A) and B (MAO‐A). Molecular docking, ADMET, and MD simulation were performed to find the binding mode of the most potent inhibitor.

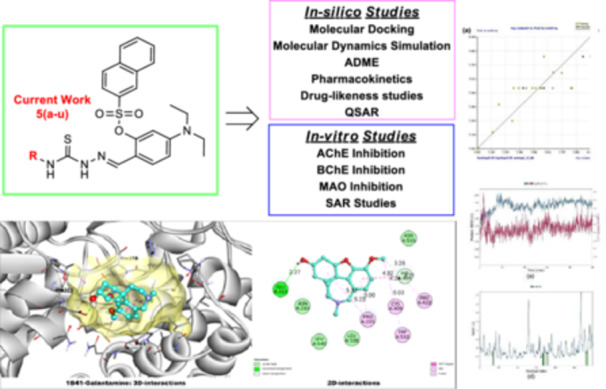

## 1 Introduction

Alzheimer's disease (AD) is a severe neurological condition linked to behavioral, psychological, and language disorders as well as memory loss [1, 2]. Numerous research studies have demonstrated that pathological conditions involving overexpression of monoamine oxidase (MAOs) and cholinesterase (ChEs) can lead to various pathological changes, such as reduced cerebral blood flow, Aβ aggregation [3], neurofibrillary tangles (NFT) [4], neuronal death, neuro‐inflammation [5], and free radical metabolic disorder [6, 7, 8]. These enzymes (ChEs and MAOs) inactivate neurotransmitters such as acetylcholine (ACh), dopamine, adrenaline, noradrenaline, and histamine in the brain [9]. The dopaminergic and cholinergic neurotransmitters in the synaptic cleft can be increased by inhibition of MAOs and ChEs [10].

ChE, specifically acetylcholinesterase (AChE, EC 3.1.1.7) and butyrylcholinesterase (BChE, EC 3.1.1.8), catalyze the hydrolysis of cholinergic neurotransmitters such as ACh and BChE. Since AChE degrades ACh more frequently than BChE, inhibiting AChE to raise ACh levels is still an effective treatment for AD [11]. This enzyme's crystal structure shows a 20 Å long gorge connecting the catalytic active site (CAS) and peripheral anionic site (PAS). Moreover, it has been noted that AChE contributes to the development of AD by inducing pro‐aggregation activity in the Aβ protein, producing reactive oxygen species (ROS), dysregulating calcium, and causing neuronal dysfunction. Thus, bioactive compounds that can specifically bind with the residues of either catalytic site (PAS or CAS) may be very helpful in inhibiting AChE while removing Aβ aggregation [12]. BChE, on the other hand, hydrolyzes both choline and aliphatic esters and is known as nonspecific pseudocholinesterase, serum ChE, or BChE [13]. It is an α‐glycoprotein and is present in the liver, most tissues, and the central and peripheral nervous systems [14].

The half‐life of BChE is about 12 days and generally falls within the range of 5900 and 13,200 IU/L [15]. It has been documented that individuals with obesity, diabetes, uremia, hyperthyroidism, and hyperlipidemia exhibit elevated activity of this enzyme [16]. Since the liver produces BChE, hepatocellular impairment causes a drop in enzyme activity. It is a biochemical indicator of organ damage; its plasma levels decrease in cirrhosis, acute and chronic liver injury, and liver metastases. In addition, low plasma BChE levels have been linked to stress (acute and chronic), inflammation, protein‐energy deficiency, and other clinical disorders [17].

Neuropsychiatric diseases such as Parkinson's disease (PD), AD, and depression, which have all become serious health problems in the world, still do not have an effective treatment, mainly due to an insufficient understanding of the multi‐component pathogenesis [18, 19]. Various approaches have been used for the treatment of neurological disorders, including natural products and prodrug strategy [20, 21]. Among the well‐studied etiologies, the irregular expression of mitochondrial enzyme MAO (EC 1.4.3.4) has been recognized as a main cause. The neurotransmitter‐catabolizing MAOs are classified into the A and B isoforms [22]. The MAO enzyme catalyzes the oxidative deamination of both endogenous and exogenous monoamines. It has important roles in metabolizing released neurotransmitters and is mainly involved in the breakdown of norepinephrine, melatonin, serotonin, and epinephrine. MAO‐A is expressed almost everywhere in the human body. MAO‐B, which degrades and metabolizes dopamine and β‐phenethylamine, is highly expressed in the central nervous system (CNS) [23]. The potential application of MAO‐B inhibitors in the treatment of neurodegenerative diseases has drawn more attention, not just for their roles in the metabolism of monoamine neurotransmitters and in mitigating oxidative stress but also for their additional neuroprotective and neurorescue properties, which are advantageous for AD treatment. Novel MAO‐B inhibitors with anti‐AD activities (such as inhibiting Aβ aggregation, chelating properties, antioxidative ability, and AChE inhibition) have recently been developed by researchers as multifunctional ligands. This bodes well for the successful treatment of several neurodegenerative diseases, including AD and PD [24, 25].

Heterocyclic derivatives are valuable precursors for the development of potential enzyme inhibitors for AD [26, 27, 28, 29]. Thiosemicarbazones are a class of chemicals with an imine moiety because of their multifunctional structure, which includes an aryl substituent that functions as a hydrophobic domain and coordination sites that contain C═S and NH groups as electron donors [30, 31, 32, 33]. They display an extensive array of pharmacological characteristics [34], which includes antifungal [35], anticancer [36], antiviral, antimalarial [37], anti‐AChE, anti‐BChE, and anti‐MAO [38] properties. Additionally, they are used as dyes [39], stabilizers [40], catalysts [41], and in the polymer industry [42, 43].

**Figure 1:**
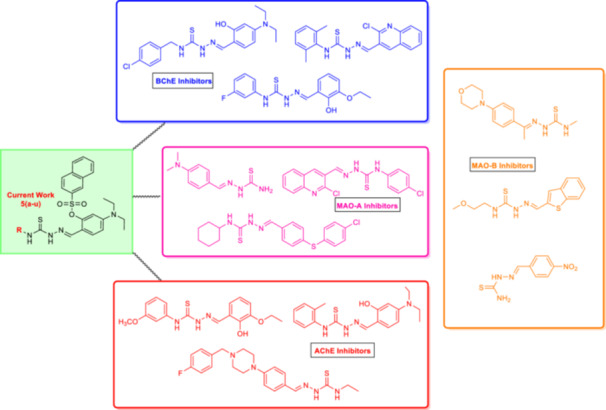
Reported structures of thiosemicarbazone‐based AChE, BChE, and MAO inhibitors.

Due to the inability of conventional monotherapies to provide long‐term relief, alternative multitargeting techniques must be investigated to address the complexity of AD. Various thiosemicarbazones have been reported to have exceptional properties, such as AChE, BChE, and MAO‐A inhibitors and MAO‐B inhibitors (Figure 1) [10, 44, 45, 46, 47].

The second pharmacophore was salicylic derivatives, which show promising properties as scaffolds for anti‐AD design. This moiety is known to have a wide range of beneficial bioactive properties, including the capacity to function as metal chelators, antioxidants, and β‐amyloid aggregation inhibitors [48, 49]. In the past, we synthesized a novel series of 4‐(diethylamino)‐salicylaldehyde thiosemicarbazones and probed them as ChE inhibitors [44].

In the present work, 4‐(diethylamino)‐salicylaldehyde‐based thiosemicarbazones **5(a–u)** are prepared and evaluated as multipotent analogs capable of concurrently inhibiting the enzymes ChE and monoamine oxidase (MAO), suggesting that they may be potentially used to treat AD.

## 2 Results and Discussion

### 2.1 Chemistry

**Scheme 1:**
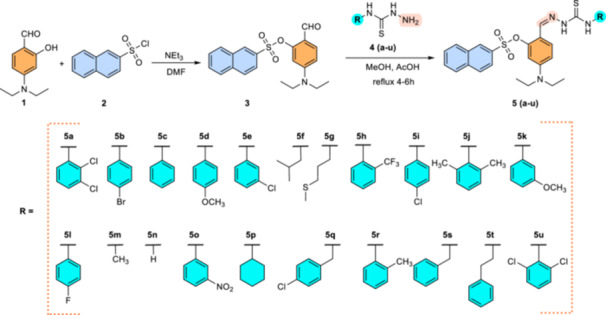
Synthetic route for the preparation of 4‐(diethylamino)salicylaldehyde‐based thiosemicarbazones.

A series of 21 derivatives, **5a–u,** have been synthesized to explore the biological potential of 4‐(diethylamino)salicylaldehyde‐based thiosemicarbazones as ChE and MAO‐A and B inhibitors. The targeted thiosemicarbazones **5a–u** were synthesized in two steps (Scheme 1).

In the first step, compound **3** was synthesized by reacting 4‐(diethylamino)salicylaldehyde **1** with naphthalene sulfonyl chloride **2** in the presence of triethylamine and DMF in an ice bath to obtain *O*‐substituted naphthalene sulfonyl‐based 4‐(diethylamino)salicylaldehyde **3**, with an excellent yield of 90%.

In the second step, to synthesize the 4‐(diethylamino)salicylaldehyde‐based thiosemicarbazones **5a–u**, the equimolar ratio of compound **3** and respective thiosemicarbazides **4a–u** was refluxed in methanol, and acetic acid was used as a catalyst. The thiosemicarbazones **5a–u** were obtained as solid residues in 85%–95% yield.

The structures of the synthesized thiosemicarbazones were confirmed using the spectral data. In ^1^H‐NMR spectra, NH–C═S protons showed a singlet between *δ* 9.72 and 9.93 ppm, while a broad singlet peak appeared from 11.29 to 11.97 ppm for NH–N═C protons. A singlet is observed in the 8.32–8.15 ppm range, which can be attributed to the azomethine hydrogen, finalizing the condensation with aldehydes. Furthermore, peaks that appeared in ^13^C‐NMR spectra also confirmed the formation of the thiosemicarbazones.

### 2.2 Biological Activity

#### 2.2.1 Structure–Activity Relationship

The current work has synthesized and evaluated a series of thiosemicarbazone derivatives **5a–u** for their activity against AChE, BChE, MAO‐A, and MAO‐B enzymes. These activity results are reported in Table 1 as IC_50_ values in the nanomolar range. SAR was explored by varying the **R** group attached to the N^4^ position of the thiosemicarbazide moiety. The results revealed that the **R** group is critical for the strong inhibitory effects. The **R** group is varied with aromatic, nonaromatic, aliphatic, and heterocyclic substituents. The synthesized compounds displayed excellent to moderate inhibition against all the isoforms of enzymes, that is, AChE, BChE, MAO‐A, and MAO‐B enzymes. It is a very encouraging behavior for the synthesized compounds, given the fact that these compounds can multitarget various enzymes that influence AD, such as AChE, BChE, MAO‐A, and MAO‐B [50]. Moreover, it is also evident from the Ki values that the compounds displayed competitive inhibition against AChE and BChE.

**Table 1:** Anti‐cholinesterase (AChE and BChE) and anti‐MAO‐A and anti‐MAO‐B potentials of synthesized compounds **5a–u** and their structures.

	IC_50_ (nM)								*K*_i_ (nM)	
Comp.	AChE	*r* ^2^	BChE	*r* ^2^	MAO‐A	*r* ^2^	MAO‐B	*r* ^2^	AChE	BChE
**5a**	14.90 ± 1.54	0.913	124.72 ± 9.13	0.982	104.17 ± 4.58	0.960	391.03 ± 9.04	0.989	11.37 ± 3.06	101.32 ± 22.43
**5b**	33.81 ± 3.41	0.906	202.05 ± 5.66	0.937	132.05 ± 7.01	0.931	409.76 ± 7.47	0.928	26.55 ± 3.34	190.57 ± 15.65
**5c**	38.42 ± 5.03	0.904	217.78 ± 8.41	0.929	188.54 ± 6.46	0.933	296.47 ± 6.01	0.990	25.43 ± 5.32	182.65 ± 15.04
**5d**	39.37 ± 2.87	0.968	252.54 ± 10.46	0.923	170.66 ± 10.42	0.935	406.18 ± 8.24	0.942	32.23 ± 5.93	234.84 ± 15.04
**5e**	31.04 ± 4.16	0.921	208.43 ± 7.53	0.951	117.13 ± 7.33	0.923	> 1000	0.935	25.36 ± 3.32	195.34 ± 11.53
**5f**	49.53 ± 5.21	0.947	253.53 ± 6.31	0.964	133.50 ± 8.04	0.901	388.10 ± 5.37	0.957	38.82 ± 6.18	239.89 ± 18.65
**5g**	80.43 ± 7.62	0.978	287.26 ± 8.57	0.970	187.66 ± 5.36	0.964	398.57 ± 7.93	0.902	72.43 ± 10.08	258.50 ± 16.14
**5h**	16.76 ± 1.35	0.949	145.03 ± 8.96	0.913	100.38 ± 4.57	0.930	249.01 ± 9.10	0.944	14.59 ± 3.36	136.01 ± 9.17
**5i**	24.81 ± 2.41	0.958	199.54 ± 9.41	0.902	121.24 ± 3.15	0.924	285.25 ± 4.02	0.912	21.02 ± 4.12	190.27 ± 13.60
**5j**	40.62 ± 2.77	0.979	265.38 ± 7.90	0.970	136.93 ± 6.21	0.981	395.37 ± 8.35	0.966	36.52 ± 5.23	228.42 ± 12.35
**5k**	116.01 ± 8.42	0.906	252.64 ± 9.15	0.977	144.71 ± 8.03	0.976	> 1000	0.952	95.75 ± 9.54	205.34 ± 10.16
**5l**	29.54 ± 2.68	0.918	200.34 ± 6.88	0.985	112.33 ± 2.78	0.907	276.97 ± 6.33	0.938	27.41 ± 3.45	182.09 ± 9.05
**5m**	38.49 ± 3.21	0.909	214.30 ± 7.13	0.963	132.28 ± 4.96	0.954	368.58 ± 11.25	0.926	30.22 ± 5.02	193.05 ± 11.62
**5n**	47.70 ± 5.67	0.970	308.43 ± 9.98	0.932	137.19 ± 6.55	0.976	395.72 ± 8.09	0.930	42.44 ± 4.10	270.16 ± 13.54
**5o**	18.93 ± 1.33	0.998	192.09 ± 6.36	0.981	122.65 ± 7.01	0.932	267.07 ± 6.35	0.905	14.25 ± 2.32	160.33 ± 9.01
**5p**	54.46 ± 4.57	0.915	254.24 ± 8.08	0.965	145.32 ± 3.57	0.985	326.68 ± 9.13	0.955	47.09 ± 6.35	226.45 ± 12.57
**5q**	19.34 ± 1.28	0.984	202.31 ± 14.86	0.963	119.06 ± 9.24	0.926	308.44 ± 6.07	0.927	17.63 ± 3.34	182.76 ± 11.48
**5r**	27.39 ± 2.42	0.912	293.55 ± 7.14	0.912	142.55 ± 6.78	0.944	495.13 ± 8.36	0.995	21.17 ± 1.05	249.09 ± 15.30
**5s**	71.43 ± 8.92	0.954	220.34 ± 8.53	0.954	131.09 ± 5.34	0.976	369.46 ± 12.04	0.975	63.86 ± 7.54	195.21 ± 13.45
**5t**	56.21 ± 4.58	0.902	271.12 ± 6.90	0.981	138.74 ± 6.02	0.932	289.02 ± 9.11	0.945	48.30 ± 7.32	226.65 ± 16.08
**5u**	12.89 ± 1.02	0.987	148.18 ± 7.35	0.973	96.25 ± 5.77	0.906	208.95 ± 5.84	0.978	9.82 ± 1.76	112.06 ± 7.61
*	101.24 ± 5.72	0.984	261.62 ± 9.03	0.945	—	—			84.05 ± 8.70	242.17 ± 13.88
**	—	—	—	—	150.62 ± 4.03	0.979	468.63 ± 16.05	0.987	—	—

*Note:* *, Galantamine; **, Clorgyline.

#### 2.2.2 AChE Inhibition

The synthesized compounds displayed excellent to moderate inhibition against AChE, with IC_50_ values ranging between 12.89 and 116.01 nM. Twenty compounds displayed superior inhibition potential compared with the standard galantamine, possessing an IC_50_ value of 101.24 nM.

Compounds **5c** and **5s** with phenyl and benzyl rings displayed IC_50_ values of 38.42 (*K*_i_ = 25.43 ± 5.32 nM) and 71.43 nM (*K*_i_ = 63.86 ± 7.54 nM), respectively. The derivatives are varied by changing the substitutions on the phenyl and benzyl rings. From the results, it can be seen that compounds with substituted phenyl and benzyl rings displayed better inhibition potential as compared with the unsubstituted rings, which revealed the importance of the substitutions.

The substitution pattern and activities of the phenyl ring can be explored as follows. The trend of IC_50_ values of the monosubstituted derivatives is given as **5o** (3‐nitrophenyl, IC_50_ = 18.93 nM) > **5i** (4‐chlorophenyl, IC_50_ = 24.81 nM) > **5r** (*o*‐tolyl, IC_50_ = 27.39 nM) > **5l** (4‐fluorophenyl, IC_50_ = 29.54 nM) > **5e** (3‐chlorophenyl, IC_50_ = 31.04 nM) > **5b** (4‐bromophenyl, IC_50_ = 33.81 nM) > **5d** (4‐methoxyphenyl, IC_50_ = 39.37 nM) > **5k** (3‐methoxyphenyl, IC_50_ = 116.01 nM). It can be seen from the trend that the inhibition potential of the compounds with electron‐withdrawing substituents, such as nitro group and halogens, outshone the compounds with electron‐donating groups such as methoxy and ethyl groups. Other than that, compounds with *para*‐substitution displayed better inhibition potency than *meta*‐substitution.

The trend of IC_50_ values of the disubstituted derivatives is given as **5u** (2,6‐dichlorophenyl, IC_50_ = 12.89 nM) > **5a** (2,3‐dichlorophenyl, IC_50_ = 14.90 nM) > **5h** (2‐trifluoromethylphenyl, IC_50_ = 16.76 nM) > **5j** (2,6‐dimethylphenyl, IC_50_ = 40.62 nM). From the IC_50_ values, it can be seen that disubstitution plays a key role in the inhibition potential of the derivatives. The three most potent members of the series are the disubstituted derivatives. Compound **5u** (*K*_i_ **=** 9.82 ± 1.76 nM) with 2,6‐dichloro substitution on the phenyl ring is the most potent member of the series.

The comparison of the strengths of aliphatic substituents as R group is as follows: **5m** (methyl, IC_50_ = 38.49 nM) > **5n** (H, IC_50_ = 47.70 nM) > **5f** (isobutyl, IC_50_ = 49.53 nM) > **5p** (cyclohexyl, IC_50_ = 54.46 nM) > **5t** (phenethyl, IC_50_ = 56.21 nM) > **5g** (3‐methylthiopropyl, IC_50_ = 80.43 nM). Compound **5m** with a methyl group displayed the highest activity in this group, but with the increase in the size of the substituent, activity starts decreasing gradually.

#### 2.2.3 BChE Inhibition

The inhibition pattern of the synthesized compounds against BChE is almost similar to the activity on AChE, with IC_50_ values ranging from 124.72 to 308.43 nM. Sixteen of the synthesized compounds displayed higher inhibition potential than the standard galantamine (IC_50_ value = 261.62 nM).

Compound **5c** and **5s** with phenyl and benzyl rings as R group displayed IC_50_ values of 217.78 (*K*_i_ = 182.65 ± 15.04 nM) and 220.34 nM (*K*_i_ = 195.21 ± 13.45 nM), respectively. Unlike against AChE, **5v** and **5s** displayed almost similar potential. The trend for monosubstitution on the phenyl ring is given as **5o** (3‐nitrophenyl, IC_50_ = 192.09 nM) > **5i** (4‐chlorophenyl, IC_50_ = 199.54 nM) > **5l** (4‐fluorophenyl, IC_50_ = 200.34 nM) > **5b** (4‐bromophenyl, IC_50_ = 202.05 nM) > **5e** (3‐chlorophenyl, IC_50_ = 208.43 nM) > **5d** (4‐methoxyphenyl, IC_50_ = 252.54 nM) > **5k** (3‐methoxyphenyl, IC_50_ = 252.64 nM) > **5r** (o‐tolyl, IC_50_ = 293.55 nM). With slight variations in the IC_50_ values of the compounds, the inhibition potential is exactly similar to that observed for AChE. Compounds with electron‐withdrawing groups displayed superior potency as compared with compounds with electron‐donating groups.

The ranking order of disubstitution on the phenyl ring is as follows: **5a** (2,3‐dichlorophenyl, IC_50_ = 124.72 nM) > **5h** (2‐trifluoromethyl phenyl, IC_50_ = 145.03 nM) > **5u** (2,6‐dichloro phenyl, IC_50_ = 148.18 nM) > **5j** (2, 6‐dimethylphenyl, IC_50_ = 265.38 nM). Compound **5a** with the 2,3‐dichloro substitution is the most potent compound of the series against BChE (*K*_i_ **=** 101.32 ± 22.43 nM). It is the second and third most powerful against the other two enzymes, AChE (*K*_i_ **=** 11.37 ± 3.06 nM) and MAO‐A, respectively.

The ranking order of the aliphatic substituents is as follows: **5m** (methyl, IC_50_ = 214.30 nM) > **5f** (isobutyl, IC_50_ = 253.53 nM) > **5p** (cyclohexyl, IC_50_ = 254.24 nM) > **5t** (phenethyl, IC_50_ = 271.12 nM) > **5g** (3‐methylthiopropyl, IC_50_ = 287.26 nM) > **5n** (H, IC_50_ = 308.43 nM). The aliphatic substitution also followed the same pattern as in AChE. In fact, with the increase in size of the substituent, the activity against BChE is diminished.

#### 2.2.4 MAO‐A Inhibition

Exploring the structure–activity relationship, the synthesized compounds displayed excellent to moderate inhibition potentials with IC_50_ values ranging between 96.25 and 188.54 nM. Eighteen compounds displayed higher inhibition potentials than the standard clorgyline, which has an IC_50_ value of 150.62 nM. It is interesting to note that the inhibition pattern of the synthesized compounds against all three enzymes, that is, AChE, BChE, and MAO‐A, is almost similar with slight variations.

**Figure 2:**
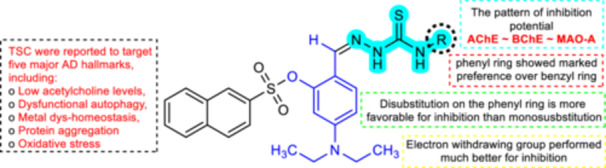
Representation of the diversity point and effect of chemical manipulation on biological activity.

Compound **5c** with unsubstituted phenyl ring displayed lower inhibition potential with an IC_50_ value of 188.54 nM than the standard clorgyline and is the least potent member of the series, while the mono and disubstituted derivatives displayed much higher potency. The ranking order of monosubstitution on the phenyl ring is given as **5l** (4‐fluorophenyl, IC_50_ = 112.33 nM) > **5i** (4‐chlorophenyl, IC_50_ = 121.24 nM) > **5o** (3‐nitrophenyl, IC_50_ = 122.65 nM) > **5b** (4‐bromophenyl, IC_50_ = 132.05 nM) > **5r** (o‐tolyl, IC_50_ = 142.55 nM) > **5k** (3‐methoxyphenyl, IC_50_ = 144.71 nM) > **5d** (4‐methoxyphenyl, IC_50_ = 170.66 nM). From the trend of IC_50_ values, the following observations can be made that electron‐withdrawing substituents are more favorable toward the inhibition against MAO‐A. With the increase in the size of the halogen substituents, the inhibition potency of the synthesized compounds starts declining (Figure 2).

The inhibition pattern for the disubstituted derivatives against MAO‐A is exactly similar to that of AChE. For the aliphatic substituents, the ranking order is as follows: **5m** (methyl, IC_50_ = 132.28 nM) > **5f** (isobutyl, IC_50_ = 133.50 nM) > **5n** (H, IC_50_ = 137.19 nM) > **5t** (phenethyl, IC_50_ = 138.74 nM) > **5p** (cyclohexyl, IC_50_ = 145.32 nM) > **5g** (3‐methylthiopropyl, IC_50_ = 187.66 nM). With the increase in the size of the substituent, the potency starts declining. All synthesized compounds contribute similarly toward inhibiting AChE, BChE, and MAO‐A and MAO B enzymes.

### 2.3 Molecular Docking Study

Molecular docking is vital for identifying potential binding sites and interactions between target molecules and proteins. This computational technique seeks an optimal conformation that minimizes the system's free energy. Predicting how a molecule interacts with a target protein aids in the design and development of new therapeutic agents by highlighting the most promising structures for further investigation [51, 52]. The docking study was accomplished using AutoDockTools 1.5.6 software [53]. Several approaches are being employed in the development of therapies for AD, with a focus on targeting key enzymes such as AChE, BChE, and MAO‐A and ‐B. Both AChE and BChE play critical roles in the regulation of neurotransmitter levels, though they differ in their distribution across brain regions. AChE is the primary target for ChE inhibitors, which aim to slow the breakdown of ACh, a neurotransmitter involved in memory and cognition. BChE, though less abundant than AChE, is also important as it functions as a co‐regulator of ACh, particularly in the later stages of AD when AChE activity decreases. Targeting both enzymes has become a promising approach in modulating cholinergic function and alleviating the cognitive decline associated with AD [10, 54, 55]. Additionally, MAO‐A enzymes are being investigated due to their role in the degradation of monoamine neurotransmitters, which also influence cognitive and behavioral symptoms in AD patients. Combining these therapeutic targets offers a multifaceted approach to addressing the complex biochemical processes underlying AD [56, 57]. With this consideration, the docking study was achieved with three target proteins with PDB Id: 1B41 (*h*‐AChE), 4BDS (*h*‐AChE), 2V5Z (*h*‐MAO‐B), and 2Z5X (*h*‐MAO‐A) (Table 2). Target 1B41 is a crystal structure of human AChE complexed with fasciculin‐II, a glycosylated protein (resolution: 2.76 Å). The receptor 4BDS is a crystal structure (resolution: 2.1 Å) of the human BChE in complex with tacrine [10, 54, 55]. The receptor 2V5Z is a crystal structure (resolution: 1.60 Å) of the human monoamine oxidase B with inhibitor safinamide [31]. And 2Z5X is a crystal structure (resolution: 2.20 Å) of human monoamine oxidase A with harmine [56, 57, 58].

**Table 2:** The binding residues and interactions of most and least bioactive scaffolds in comparison with the standard.

Target PDB Id	Comp. Id/Std.	Docking score (kcal/mol)	Binding residues and interactions [residue code (type of interaction)]
1B41 (*h*‐AChE)	5u	−8.2	ASN A:233 (hydrogen bond); ASN A:533 (hydrogen bond); GLU A:313 (hydrogen bond, salt bridge); THR A:311 (pi‐sigma); PRO A:235 (pi‐sigma, carbon‐hydrogen bond); PRO A:312 (carbon‐hydrogen bond); THR A:238 (unfavorable acceptor‐acceptor); VAL A:303 (pi‐alkyl)
	5k	−7.1	ASN A:233 (hydrogen bond); GLY A:234 (hydrogen bond); HIS A:405 (pi‐cation); GLU A:313 (pi‐anion); PRO A:235 (pi‐alkyl); VAL A:239 (alkyl)
	Galantamine	−6.9	GLU A:313 (hydrogen bond); HIS A:405 (carbon‐hydrogen bond, pi‐pi T‐shaped); PRO A:410, CYS A:409, TRP A:532 (pi‐alkyl); PRO A:235 (pi‐alkyl, alkyl)
4BDS (*h*‐BChE)	5a	−11.3	HIS A:438 (hydrogen bond, pi‐pi stacked, unfavorable positive‐positive); PHE A:329 (pi‐pi T‐shaped, Amide‐pi stacked, pi‐cation); TRP A:231 (pi‐pi stacked); GLY A:116 (pi‐pi stacked); TRP A:82 (pi‐pi stacked); LEU A:286 (pi‐alkyl); PRO A:285, TYR A:332 (alkyl)
	5n	−9.3	TYR A:332 (hydrogen bond); ASP A:70 (attractive charge); TRP A:82 (pi‐pi stacked); HIS A:438, PHE A:329 (pi‐alkyl)
	Galantamine	−9.3	HIS A:438 (hydrogen bond); SER A:198, (hydrogen bond, unfavorable donor‐donor); GLY A:115, GLY A:117 (hydrogen bond); GLU A:197 (unfavorable acceptor‐acceptor); TRP A:82, TRP A:231 (pi‐alkyl)
2Z5X (*h*MAO‐A)	5u	−8.6	TYR A:121 (hydrogen bond, pi‐alkyl); GLU A:492 (hydrogen bond, salt bridge, pi‐anion); PHE A:112 (pi‐pi T‐shaped); TYR A:124 (pi‐sulfur); TRP A:116 (pi‐sigma, pi‐alkyl); PRO A:114 (pi‐alkyl, alkyl)
	5c	−8.2	ARG A:109 (hydrogen bond); GLU A:492 (pi‐anion); TRP A:116, TYR A:121 (pi‐sigma, pi‐alkyl); TRP A:128, PHE A:112 (pi‐pi T‐shaped); TYR A:124 (pi‐pi T‐shaped, pi‐sulfur); TYR A:121 (pi‐sulfur); ALA A:111 (pi‐alkyl)
	Harmine	−8.6	GLY A:67 (carbon‐hydrogen bond); TYR A:407 (pi‐pi stacked); TYR A:444 (pi‐pi stacked); LEU A:337 (pi‐alkyl); PHE A:352 (pi‐alkyl); MET A:445, TYR A:69 (alkyl); FAD A:600 (unfavorable bump)
2V5Z (*h*MAO‐B)	**5u**	−7.8	GLU A:391 (2 hydrogen bonds, attractive charge); GLN A:392 (hydrogen bond); LEU A:250 (pi‐sigma, pi‐alkyl); TYR A:393 (pi‐sulfur, unfavorable donor‐donor); ARG A:36 (pi‐alkyl, unfavorable donor‐donor); PRO A:234 (pi‐alkyl); PRO A:277 (pi‐alkyl)
	**5k**	−6.7	GLU A:391 (hydrogen bond, attractive charge); ASP A:37 (hydrogen bond); TYR A:393 (pi‐pi T‐shaped); TYR A:44 (pi‐pi T‐shaped); ARG A:38 (carbon‐hydrogen bond); ARG A:36, PRO A:277 (pi‐alkyl); PRO A:234 (alkyl)
	Safinamide	−9.8	TYR A:398 (pi‐pi stacked); PRO A:102 (hydrogen bond); GLN A:206 (hydrogen bond); LEU A:171 (pi‐sigma); TYR A:326 (pi‐pi T‐shaped); CYS A:172 (pi‐sulfur); LEU A:164, LEU A:167 (pi‐alkyl); TRP A:119, ILE A:199 (alkyl)

**Figure 3:**
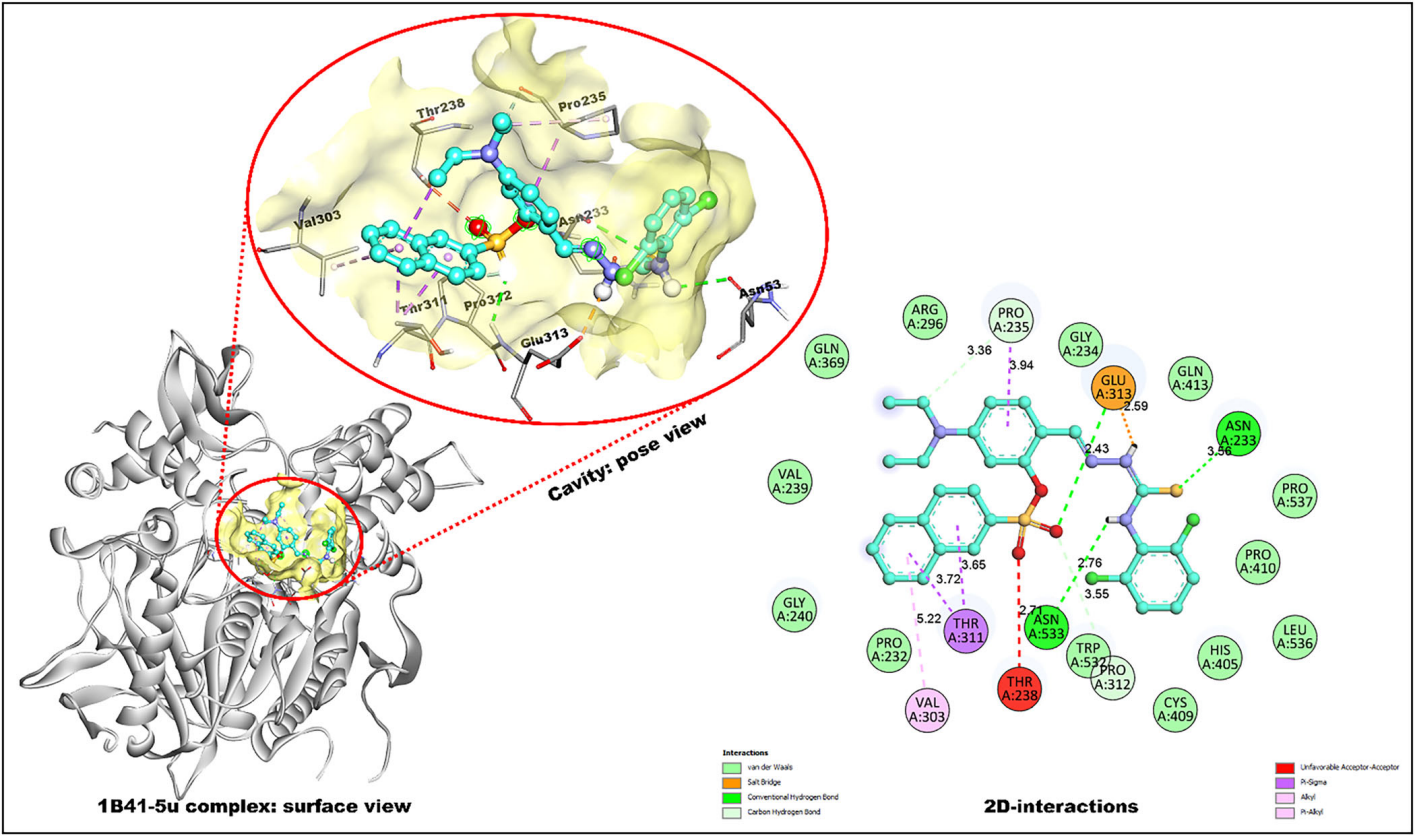
Binding interactions of compound **5u** within the 1B41 site.

The molecular docking analysis revealed that compound **5u** demonstrated the highest binding affinity (−8.2 kcal/mol) against 1B41 (Figure 3). The thione moiety established hydrogen bonding (3.56 Å) with ASN A:233, while the carbothioamide NH engaged ASN A:533 (2.76 Å).

Critically, the sulfonate oxygen formed a strong hydrogen bond (2.43 Å) and a salt bridge (2.59 Å) with GLU A:313, emphasizing electrostatic stabilization. π–σ and π–alkyl interactions were observed between the naphthalene ring and THR A:311 (3.65 Å) and VAL A:303 (3.72, 5.22 Å). The diethylamino‐phenyl group displayed π–σ (3.94 Å) and C–H bond (3.36 Å) contacts with PRO A:235, while an unfavorable acceptor–acceptor interaction (2.71 Å) was noted with THR A:238.

The least active analog, **5k** (−7.1 kcal/mol), formed two hydrogen bonds with ASN A:233 (2.30, 2.63 Å) and one with GLY A:234 (2.40 Å). It showed a π–cation interaction with HIS A:405 (4.86 Å), a π–anion interaction with GLU A:313 (3.25 Å), and π–alkyl contacts with PRO A:235 (4.75, 5.04 Å). Reference drug galantamine (−6.9 kcal/mol) interacted via H‐bonding with GLU A:313, π–π T‐shaped and C–H bonding with HIS A:405, and π–alkyl contacts with PRO A:410, CYS A:409, and TRP A:532.

**Figure 4:**
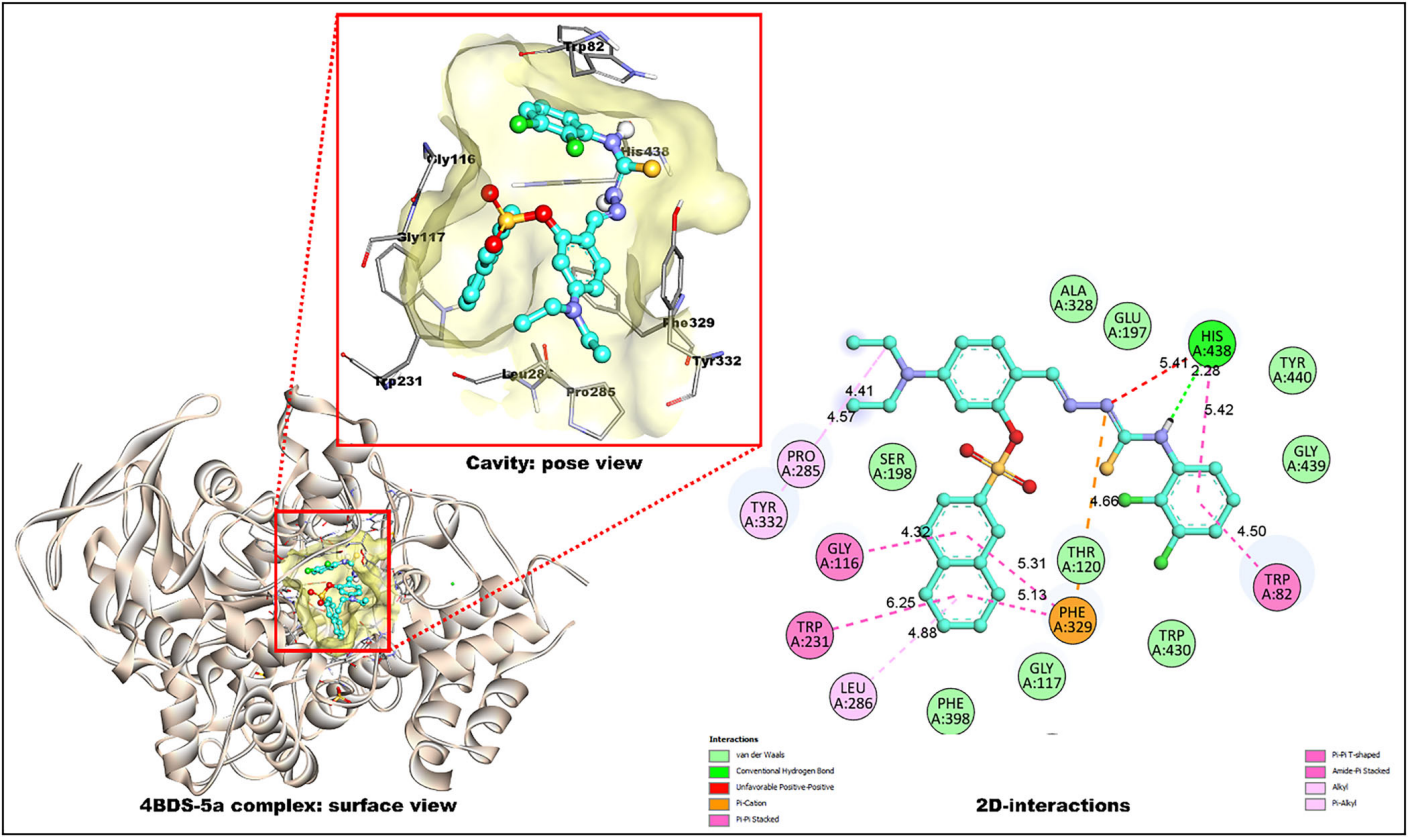
Binding interactions of compound **5a** within the 4BDS site.

Against 4BDS, compound **5a** showed the strongest binding (−11.3 kcal/mol). Its NH moiety engaged HIS A:438 via hydrogen bonding (2.28 Å), while the tricyclic naphthalene core established π–π stacking with PHE A:329, GLY A:116, and both TRP A:82 and TRP A:231 (4.32–6.25 Å) (Figure 4). Additionally, alkyl and π–alkyl interactions occurred with LEU A:286, PRO A:285, and TYR A:332. The least active **5n** (−9.3 kcal/mol) displayed a H‐bond with TYR A:332 (2.80 Å), π–π stacking with TRP A:82 (4.28, 4.73 Å), and a π–alkyl contact with HIS A:438.

**Figure 5:**
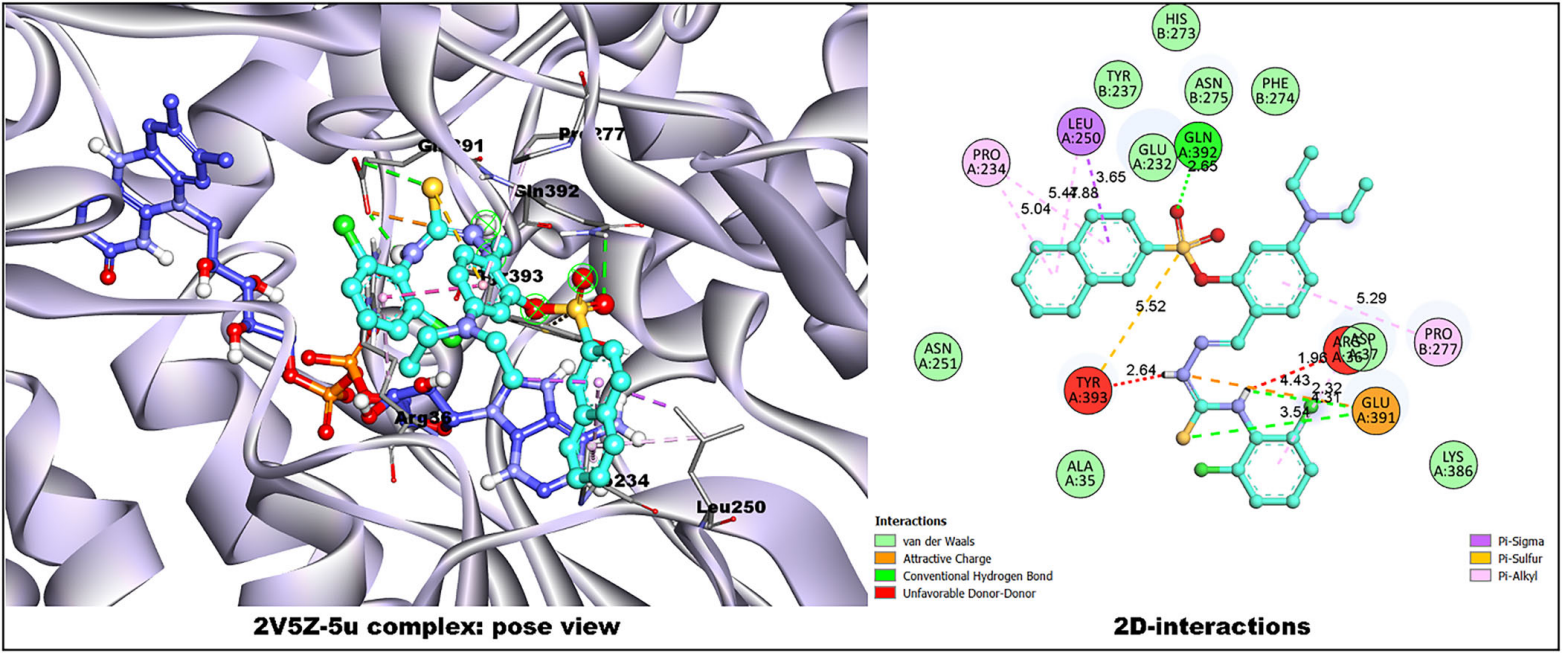
Complex of compound **5u** and target protein 2V5Z co‐crystalized with FAD molecule (blue‐colored) and their 2D‐binding interactions.

In 2Z5X, **5u** (−8.6 kcal/mol) formed hydrogen and electrostatic interactions with GLU A:492 (2.31, 2.55 Å), π–anion interaction (3.93 Å), and π–π stacking with PHE A:112. TYR A:121 participated in both H‐bonding (2.25 Å) and π–alkyl bonding (4.93 Å), while TYR A:124 established a π–sulfur bond (5.24 Å). Comparatively, harmine bound less efficiently, forming π–π and π‐alkyl interactions with TYR A:407 and LEU A:337. In 2V5Z, **5u** formed strong interactions with GLU A:391 (H‐bonds 2.32, 3.54 Å, salt bridge 4.31 Å), GLN A:392 (H‐bond 2.65 Å), and multiple π–alkyl contacts (Figure 5). The reference safinamide showed the highest binding (−9.8 kcal/mol) through extensive π–π (TYR A:398, TYR A:326), π–sulfur (CYS A:172), and hydrogen bonding (GLN A:206, PRO A:102). Detailed interactions for selected compounds have been provided in the Supporting Information.

### 2.4 Docking Validation

**Figure 6:**
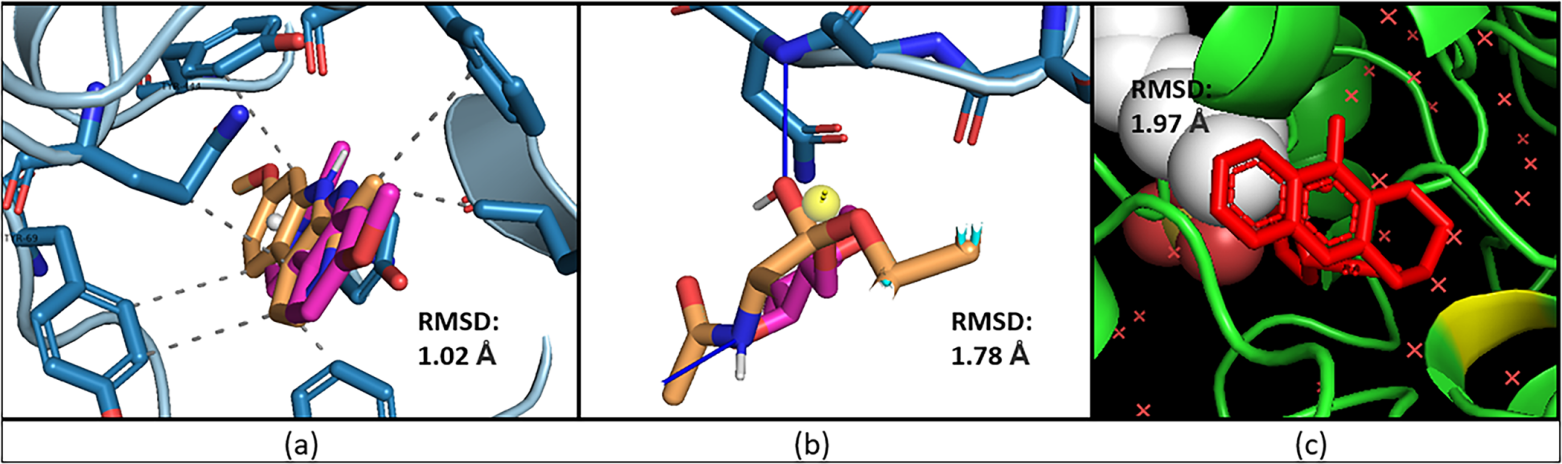
Redocking analysis and their corresponding RMSD values for target (a) *h*MAO‐A (PDB ID: 2Z5X), (b) AChE (PDB ID: 1B41), and (c) BChE (PDB ID: 4BDS).

Redocking is carried out to validate molecular docking studies, ensuring the accuracy of predicted ligand–receptor binding poses. The same protocol used in the initial docking was employed for redocking. Upon completion, the redocked complex was superimposed using Discovery Studio software. Remarkably, the redocked complex was aligned with the native complex, without any modifications (Figure 6). Some crucial amino acid residues were identified in the active pocket of each receptor, and these residues were preserved in the redocked complex of the synthesized ligand.

### 2.5 Evaluation of GA‐MLR QSAR Model

To ensure proper feature selection, the data set was split into training (≈80%) and prediction (≈20%) sets to avoid data losses. We used the “GA‐MLR” approach using “QSARINS‐2.2.4” for subjective feature selection (SFS), employing Q² _LOO_ as a fitness parameter [7]. The final model was evaluated for internal and external validity, selecting the top QSAR model based on higher *Q*² and *R*² metrics. Thus, we developed four models for each activity, Models 1‐4 as given below:

**Pki** = 7.897 + −0.214 * fnotringSC7B + −0.271 * fsp2Ssp3C7B + 0.249 * **notringC_Cl_8B”‐‐‐‐‐**model 1 (For AChE)

(**fnotringSC7B:** frequency of occurrence of carbon atom exactly at seven bonds from the non‐ring sulfur atoms; **fsp2Ssp3C7B:** frequency of occurrence of sp3 hybridized carbon atom exactly at seven bonds from the sp2 hybridized sulfur atoms; **notringC_Cl_8B:** occurrence of chlorine atom within eight bonds from the non‐ring carbon atoms).

“**pKi** = 6.565 + 0.133 * **Cl_S_6B** + 0.036 * **faccaroC6B**”‐‐‐‐‐‐‐‐‐‐‐‐‐‐‐‐model 2 (For BChE)

(**Cl_S_6B**‐ occurrence of sulfur atom within six bonds from the chlorine atom; **faccaroC6B:** frequency of occurrence of aromatic carbon atom exactly at six bonds from the acceptor atoms).

“_**P**_ **IC**_**50**_ = 6.898 + −0.034 * **com_sp3C_6A** + 0.052 * **Cl_notringC_6B** + −0.148 * **fdonnotringC6B**”‐‐‐‐model 3 (For MAO‐A)

(**com_sp3C_6A**: occurrence of sp3 hybridized carbon atom within 6 angstrom units from the center of mass of molecule; **Cl_notringC_6B**: occurrence of non‐ring carbon atoms within six bonds from the chlorine atom; **fdonnotringC6B**: frequency of occurrence of non‐ring carbon atoms exactly at 6 bonds from the donor atoms).

_**P**_ **IC**_**50**_ = **5.414** + 0.051 * com_Chyd_8A + 0.473 * fOS7B + −0.035 * C_sp3C_9B‐‐‐‐‐model 3 (For MAO‐B)

(**com_Chyd_8A:** occurrence of hydrophobic carbon atoms within 8 angstrom units from the center of mass of the molecule; **fOS7B:** frequency of occurrence of sulfur atom exactly at 7 bonds from the oxygen atoms; **C_sp3C_9B:** occurrence of sp3 hybridized carbon atoms within 9 bonds from the carbon atoms).

**Figure 7:**
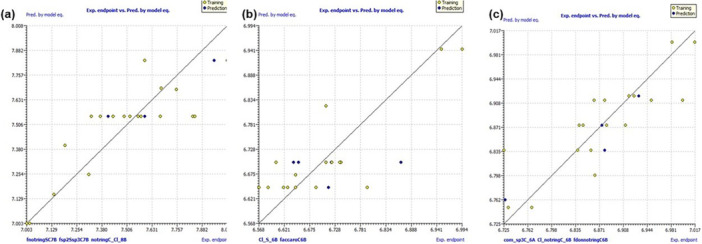
Experimental vs. predicted activities for model‐1 (a); model‐2 (b) and (c) for model‐3 obtained with QSAR equations.

All four models are depicted in Table 3 for their respective statistics and show statistical robustness among them. The QSAR plots for the correlation between experimental and observed activities are also shown in Figure 7. Other necessary plots are provided in *the* Supporting Information.

**Table 3:** Statistical parameters for developed QSAR models.

Statistical parameter	Model 1	Model 2	Model 3	Model 4
R^2^_tr	0.94	0.86	0.78	0.80
Adj‐R^2^	0.92	0.84	0.73	0.76
F(3‐13)	62.39	44.21	15.33	17.69
RSS_tr	0.09	0.03	0.03	0.09
MSE_tr	0.01	0.00	0.00	0.01
RMSE_tr	0.07	0.04	0.04	0.07
MAE_tr	0.06	0.03	0.03	0.06
s	0.08	0.04	0.04	0.08
AIC	−31.73	−52.77	−51.98	−30.39
BIC	−27.56	−49.43	−47.81	−26.23
CCC_tr	0.97	0.93	0.88	0.89
Q^2^_cv	0.90	0.80	0.65	0.78
RMSE_cv	0.09	0.05	0.05	0.33
MSE_cv	0.01	0.00	0.00	0.11
PRESS_cv	0.13	0.04	0.04	0.53
MAE_cv	0.08	0.04	0.04	0.25
R^2^_Y_scr_	0.18	0.14	0.18	0.20
MSE_ex_	0.11	0.02	0.00	0.11
RMSE_ex_	0.33	0.13	0.05	0.33
PRESS_ex_	0.53	0.08	0.01	0.53
Q^2^F_1_	−0.52	−0.68	0.47	0.81
Q^2^F_2_	−1.84	−0.71	0.09	0.84
Q^2^F_3_	−0.38	−0.32	0.61	0.84
MAE__ex_	0.32	0.12	0.04	0.23
K	1.02	1.00	1.01	0.98
K_prime	0.98	1.00	0.99	1.02
R^2^ext	0.17	0.02	0.53	0.36
CCC_ex	0.30	−0.12	0.47	−0.37
r^2^m_ExPy	0.13	−0.01	−0.04	−0.65
r^2^m_EyPx	‐0.02	0.00	0.44	−0.02
R^2^_o_	−1.14	−0.70	0.50	−0.75
R^2^_o__dash	0.11	−2.28	−0.65	−7.49
Clos_dash	0.34	141.02	2.24	21.77
Clos	7.85	44.06	0.06	3.08
r^2^_m__avg	0.05	0.00	0.20	−0.33
r^2^_m___delta	−0.15	0.01	0.48	0.63

### 2.6 Interpretations of QSAR Models

#### 2.6.1 For Model‐1

The analysis focuses on three molecular descriptors and their effects on the binding affinity (p*K*_i_) of compounds. The descriptor **fnotringSC7B** measures the frequency of carbon atoms located exactly seven bonds away from non‐ring sulfur atoms, which are not part of any cyclic structure. This descriptor has a negative coefficient (−0.214), indicating that an increase in the occurrence of these carbon atoms correlates with a decrease in p*K*_i_, suggesting reduced binding affinity for the compound. The second descriptor, **fsp2Ssp3C7B**, tracks the occurrence of sp3 hybridized carbon atoms positioned seven bonds away from sp2 hybridized sulfur atoms, which are involved in double bonds or planar structures. This descriptor also shows a negative effect on p*K*_i_ (−0.271), meaning that more frequent configurations of sp3 carbon atoms in this arrangement lead to lower predicted p*K*_i_ values, further indicating a decrease in binding affinity. In contrast, the descriptor **notringC_Cl_8B** measures the presence of chlorine atoms within eight bonds of non‐ring carbon atoms. This arrangement positively impacts p*K*_i_, implying that the proximity of chlorine to non‐ring carbon enhances binding affinity. The model's interpretation highlights these contrasting influences: the presence of chlorine near non‐ring carbon atoms increases binding affinity, while specific configurations involving carbon and sulfur atoms decrease it.

#### 2.6.2 For Model‐2

The analysis examines two molecular descriptors, **Cl_S_6B** and **faccaroC6B**, and their influence on the binding affinity (p*K*_i_) of compounds. **Cl_S_6B** quantifies the occurrence of sulfur atoms within six bonds of chlorine atoms. This descriptor has a positive coefficient (+0.133), indicating that a greater frequency of sulfur near chlorine enhances the compound's binding affinity, thus improving its potency against its target. The presence of this molecular feature is seen as beneficial for chemical activity. **faccaroC6B** assesses the frequency of aromatic carbon atoms that are exactly six bonds away from electron‐accepting atoms, such as oxygen or nitrogen. This descriptor also has a positive coefficient (+0.036), suggesting that while aromatic carbon atoms near acceptor atoms slightly contribute to increasing pKi, their effect is less pronounced than that of the Cl_S_6B descriptor. In summary, both descriptors positively influence the binding affinity of compounds: **Cl_S_6B** has a significant impact, enhancing potency, while **faccaroC6B** provides a minor boost.

#### 2.6.3 For Model‐3

The analysis explores three molecular descriptors and their impact on the inhibitory potency (pIC_50_) of compounds: **com_sp3C_6A**, **Cl_notringC_6B**, and **fdonnotringC6B**. Descriptor “**com_sp3C_6A**” refers to the occurrence of sp3 hybridized carbon atoms within a 6‐angstrom radius of the molecule's center of mass. The negative coefficient (–0.034) indicates that an increase in these carbon atoms correlates with a slight decrease in pIC_50_, suggesting that their presence weakens the biological activity of the compound. “**Cl_notringC_6B**” measures non‐ring carbon atoms located within six bonds of chlorine atoms. This descriptor has a positive coefficient (+0.052), meaning that such proximity enhances the pIC_50_ value, thereby increasing the compound's inhibitory potency and making it more effective. “**fdonnotringC6B**” assesses the frequency of non‐ring carbon atoms exactly six bonds away from electron donor atoms, like oxygen or nitrogen. This descriptor has a negative coefficient (–0.148), indicating that more non‐ring carbon atoms at this distance decrease pIC_50_, which diminishes the compound's potency.

#### 2.6.4 For Model‐4

Important molecular descriptors give us important information about how structural features affect biological activity in the QSAR model for MAO‐B inhibitors. We measure hydrophobic carbon atoms (com_Chyd_8A) within an 8‐angstrom radius from the molecule's center of mass. Because of their hydrophobic nature, the enzyme's active region contains residues like Ile‐199 and Leu‐171. These atoms are necessary to stop MAO‐B from working. When these residues and inhibitors interact hydrophobically, the binding affinity gofes up. Non‐polar parts of the inhibitor stay in the binding pocket. A positive coefficient in the QSAR model means that the inhibitory activity (PIC_50_) goes up when the hydrophobic carbon density at the center of the molecule goes up. This is what drugs like rasagiline and selegiline do to make their effects more selective and potent. One important indicator is sulfur‐oxygen connectivity (fOS7B), which measures the number of sulfur atoms that are exactly seven bonds away from oxygen atoms. Sulfur‐containing groups, like thiols and thioethers, help biological activity through electron transfer and redox interactions. Oxygen atoms, on the other hand, help hydrogen bonds form and make the substance more soluble in water. This particular connection arrangement facilitates ligand binding via both polar and nonpolar interactions. A positive coefficient (*β *> 0) shows how important this feature is for improving MAO‐B binding efficiency. For example, sulfur‐containing scaffolds like thiochromones can selectively block MAO‐B. Finally, the term sp3‐hybridized carbon atoms (C_sp3C_9B) quantifies the number of such carbons within nine bonds of any carbon atom. Excessive carbons, prevalent in aliphatic chains and saturated hydrocarbons, often diminish molecular planarity. Lack of planarity could make it harder for molecules to π‐stack, which is needed to bind to aromatic residues in MAO‐B, like Tyr‐398 and Tyr‐435. On the other hand, molecules that are too flexible might not fit correctly into the enzyme's binding pocket. If the coefficient is less than zero, it means that reducing the number of sp3‐hybridized carbons increases the inhibitory effect by making molecules flatter and more compact.

In summary, the model‐3 identifies contrasting influences on inhibitory activity: non‐ring carbon atoms near chlorine atoms enhance potency, while sp3 hybridized carbons near the center of mass and non‐ring carbons near donor atoms reduce it. These insights emphasize how specific structural arrangements can significantly affect a compound's biological activity, guiding future design efforts to optimize potency.

### 2.7 Molecular Dynamics (MD) Simulation

**Figure 8:**
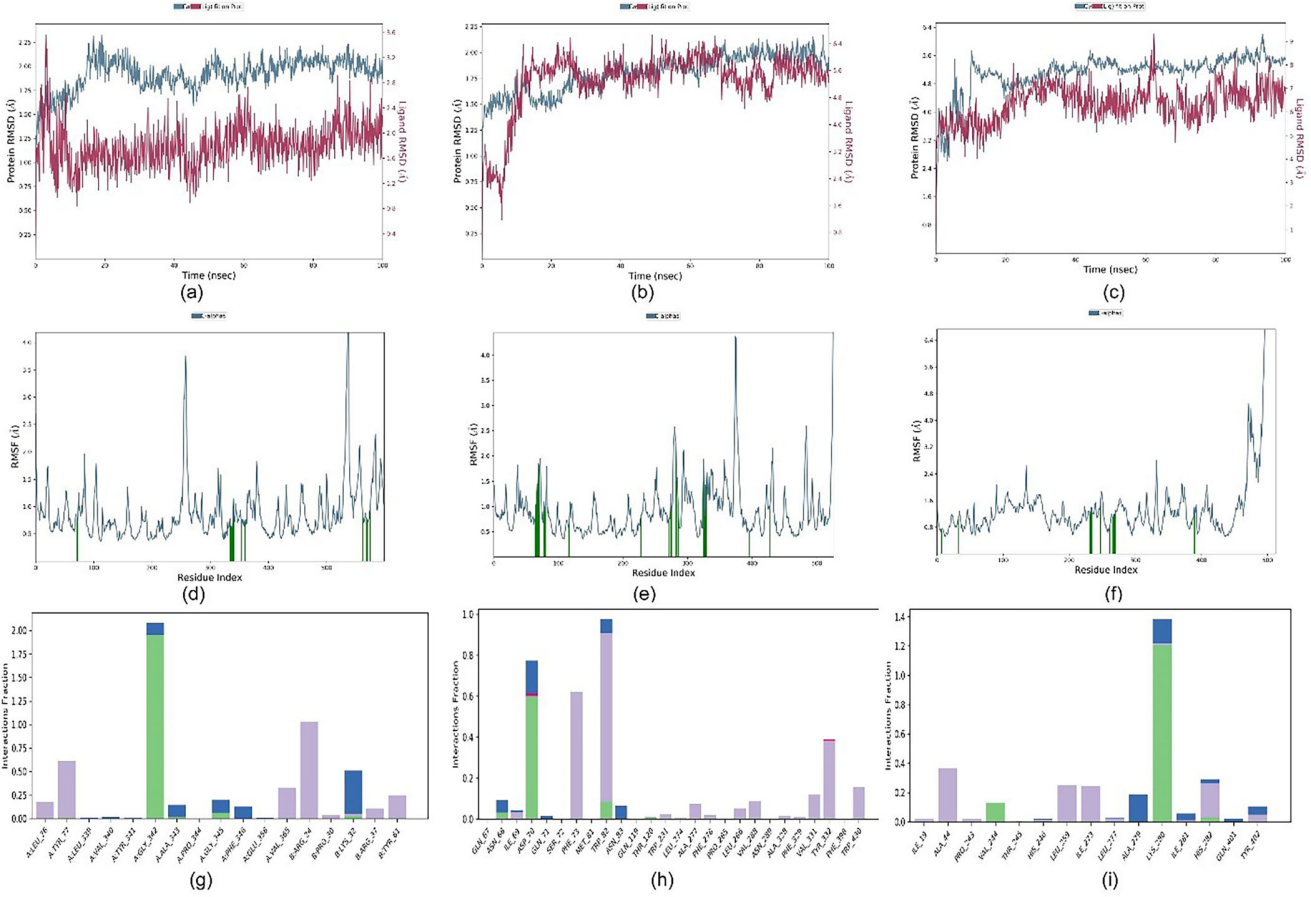
MD simulation plots for complexes “1B41_**5u**,” “4BDS_**5a**,” and “2Z5X_**5u**” over the period of 100 ns, each for (a–c) RMSD analysis; (d–f) RMSF analysis; and (g–i) ligand–protein contact plots, respectively.

**Figure 9:**
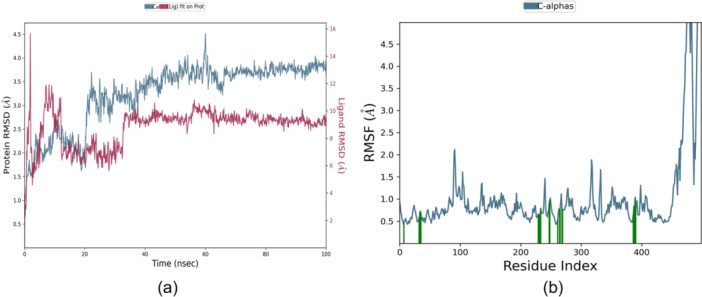
MD simulation plots (a) RMSD and (b) RMSF for complex “2v5z‐**5u**” for the period of 100 ns.

MD simulations provide crucial insights into the stability and dynamics of protein–ligand complexes. This study explored four distinct protein–ligand complexes to assess their stability and the molecular interactions involved. Thus, we simulated three docked complexes “1B41_**5 u**,” “4BDS_**5a**,” “2v5z‐**5u**,” and “2Z5X_**5u**” throughout 100 ns each (Figures 8 and 9). All three simulations retained a substantial stability over the period with fewer to no fluctuations among RMSD values. Compound **5u** showed promising interactions with AChE, while Compound **5a** showed strong interactions with BChE. Additionally, Compound **5u** effectively inhibited MAO‐A and MAO‐B. Through MD simulations, we analyzed how these molecular interactions evolved, enhancing our understanding of the dynamics within the protein–ligand complexes. These insights could pave the way for developing new drugs and therapeutics.

At the beginning of the simulation of complex “1B41_**5u**,” we observed no fluctuations of amino acid residues; however, we noticed that this complex retained the root mean square deviation (RMSD) value below 2.25 Å throughout the 100 ns simulation time (Figure 8a). The overall system consists of 43,742 atoms and 11,486 water molecules. The simulation period between 20 and 100 retained the best stability of ligand–protein stability as indicated by a constant RMSD value below 1.75 Å. Ligand‐fit‐protein was obtained very well. To see local changes in protein chains, we usually prefer to use the “RMSF” parameter. There were minor fluctuations annotated with index residues 78, in between 300 and 400, and around 580 in both complexes, that is, “1B41_**5u**” and "4BDS_5a” (Figure 8d). By analyzing the “protein secondary structure (SSE),” it was clear that there was the involvement of 22.78% Helix, 16.35% strands, and 39.14% total SSE. For both complexes, that is, “1B41_**5u**” and “4BDS_**5a**,” ligand root mean square fluctuation (l‐RMSF) values indicated key changes in ligand atom positions. For complex, “1B41_**5u**,” hydrophobic interactions were seen with Leu76, Tyr77, Glu358, Val365, Arg24, Pro30, Arg37, and Tyr61 (Figure 8g). Three H‐bonds were seen with amino acid residues such as Gly342, Gly345, and Lys32. Water bridges were observed with Val340, Gly342, Ala343, Phe346, and Lys32. Arg24 retained 91% Pi‐cationic type interaction with target 1B41.

In the case of complex, “4BDS_**5a**,” we observed a stable RMSD value below 2 Å (Figure 8b). RMSF plot also depicted fewer fluctuations around residue index 100, between 200 and 300 (Figure 8e). SSE elements showed involvement of 27.25% Helix and 14.04% strand, accounting for 41.29% total SSE. The l‐RMSF value was retained mostly below 2 Å. Four H‐bonding interactions were seen with amino acid residues such as Gln67, Asp70, Thr82, and Thr120. We annotated mostly hydrophobic interactions with Asn68, Asp70, Phe73, Trp82, Trp231, Leu274, and Trp430 (Figure 8h). Ionic interactions were seen with amino acids such as Asp70, Pro285, and Tyr332. The majority of Phe73 denoted the highest π–π stacking (34% total) interaction with ligand **5a**. While Asp70 indicated a strong negative‐charged interaction with the S atom (60%). From Figure 8, it was clear that compound **5u** also had good binding toward the MAO‐B target. This complex had 36530 atoms in the system while it was simulated. The RMSD value was retained below 4.5 Å for most of the simulation. Only minor fluctuations can be seen from the RMSF plot (Figure 9b). Protein secondary structure analysis showed 25.13% involvement of helices and 16.30% of strands in it.

In our investigation of the protein–ligand complex “2Z5X_**5u**,” we observed a stable root mean square deviation (RMSD) value consistently below 4.8 Å (Figure 8c), indicating robust stability throughout the simulation period. The RMSF analysis further illustrated minimal fluctuations, particularly around residue index 400 and within the range of 200–300, suggesting a stable structural conformation in these regions (Figure 8f). The secondary structure elements (SSE) analysis revealed a composition of 25.98% alpha helices and 15.34% beta strands, contributing to a total of 41.31% SSE, which underscores the structural integrity of the complex. The local RMSF (l‐RMSF) values were predominantly maintained below 3 Å, reinforcing the notion of localized stability among the residues. Analysis of molecular interactions highlighted four hydrogen bonds involving key amino acid residues, specifically Val244, Lys280, and His282. Additionally, we annotated several hydrophobic interactions with residues including Ile19, Ala44, Pro243, His246, Leu259, Ile273, and Tyr402 (Figure 8i), which likely contribute to the overall stability of the complex. Notably, no ionic interactions were identified within the system, indicating a lack of charge‐based stabilization. Furthermore, we observed water‐mediated interactions, or water bridges, involving residues Ala279, Ile281, Tyr402, and Lys280, which may enhance the solvation dynamics and contribute to the overall stability of the protein–ligand interaction. These findings provide valuable insights into the molecular interactions and stability characteristics of the “2Z5X_**5u**” complex, contributing to a deeper understanding of its functional dynamics.

## 3 Conclusion

A series of thiosemicarbazone derivatives (**5a–u**) were successfully synthesized and characterized through ^1^HNMR and ^13^CNMR. All the derivatives were evaluated against AChE, BChE, and MAO‐A enzymes in search of lead candidates. All compounds showed varied degrees of inhibition, ranging between 12.89 and 116.01 nM (AChE) and between 124.72 and 308.43 nM (BChE) as compared with the standard drug galantamine (IC_50_ values of 101.24 and 261.62 nM, respectively). Compound **5u** (IC_50_ = 12.89 nM) has a 2,6‐diCl substitution on the phenyl ring, and compound **5a** (IC_50_ = 124.72 nM) has a 2,3‐diCl substitution on the phenyl ring, showing strong binding interaction with the active site of enzyme, reducing the catalytic activity, and was identified to be the most active inhibitor of AChE and BChE enzymes. Compound **5u** (IC_50_ = 96.25 nM) showed the most active inhibitor of monoamine oxidase (MAO‐A) enzyme. Among all compounds, **5a** exhibited the strongest binding affinity with 4BDS (−11.3 kcal/mol), forming multiple stabilizing interactions with HIS A:438, PHE A:329, and TRP A:82. For 1B41, compound **5u** showed the best binding (−8.2 kcal/mol), engaging residues GLU A:313 and ASN A:233 through hydrogen bonding and salt bridges. Against 2Z5X, **5u** again demonstrated potent affinity (−8.6 kcal/mol), interacting via hydrogen bonds and π–anion interactions with GLU A:492 and TYR A:124. Finally, **5u** also bound strongly to 2V5Z (−7.8 kcal/mol), forming hydrogen bonds and charge interactions with GLU A:391 and GLN A:392, confirming its multi‐target potential. The promising inhibitory activities of compounds **5a** and **5u** against key enzymes related to neurological disorders, along with supportive ADME and QSAR analyses, suggest their potential as lead candidates for further drug development targeting CNS diseases. These findings encourage continued exploration and optimization of this compound series to enhance efficacy and pharmacokinetic profiles.

Our results suggested that **5a** and **5u** can be considered potential candidates for the treatment of neurological disorders such as AD.

## 4 Experimental

### 4.1 Chemistry

#### 4.1.1 General

For the synthesis of indole‐based thiosemicarbazones, all the starting materials, such as 4‐(diethylamino)salicylaldehyde, were bought from Sigma Aldrich. Chemicals and solvents, including triethylamine, methanol, DMF, glacial acetic acid, petroleum ether, and ethyl acetate, were bought from Merck and used as original. Silica gel plates with aluminum back were utilized to check the reaction progress and completion. A Bruker Ascend 400 MHz NMR spectrometer was utilized to get ^1^H and ^13^C‐NMR spectra in deuterated solvent DMSO‐d_6_ at 25°C (400 MHz for ^1^H and 101 MHz for ^13^C). NMR spectra were presented as chemical shifts (ppm), and coupling constants (*J*) were demonstrated in Hertz (Hz) to detail signal multiplicity.

The InChI codes of the investigated compounds, together with some biological activity data, are provided as Supporting Information.

#### 4.1.2 General Procedure for the Synthesis of 5‐(Diethylamino)‐2‐Formylphenyl Naphthalene‐2‐Sulfonate (**3**)

In a round‐bottom flask, 0.5 g (2.5 mmol) of 4‐(diethylamino)salicylaldehyde **1** and 0.348 mL (2.5 mmol) of triethylamine were added. DMF (3 mL) was added while stirring in an ice bath. After that, 0.56 g (2.5 mmol) of naphthalene sulfonyl chloride **2** was added with continued stirring. TLC was used to track the progress of the reaction. The reaction was terminated after an hour upon completion. The reaction mixture was poured into 20 mL of ice‐cold water. The precipitated solid was filtered and dried to obtain the product **3** in 95% yield.

#### 4.1.3 General Procedure for the Synthesis of Thiosemicarbazones **5a–u**

The targeted thiosemicarbazones were synthesized in a single‐step condensation reaction. In an oven‐dried one‐neck round‐bottom flask, equimolar quantities of 4‐(diethylamino)salicylaldehyde (0.1 g, 0.01 mmol) and respective thiosemicarbazides (**4a–u**) were added in methanol (10 mL). Three to four drops of glacial acetic acid were used as a catalyst in the reaction mixture. The reaction mixture was refluxed for 5–6 h, and then TLC was used to determine the progress of the reaction using a mixture of 1:2 petroleum ether and ethyl acetate as the eluent. After completion of the reaction, the solid product so formed was filtered, washed, and dried. The residue was crystallized with ethanol.

*(E)*‐2‐({2‐[(2,3‐Dichlorophenyl)carbamothioyl]hydrazono}methyl)‐5‐(diethylamino)phenyl naphthalene‐2‐sulfonate (**5a**): Yield: 92%; M.P: 202°C–204°C; Color: yellow; ^1^H‐NMR: *δ* (400 MHz, DMSO‐*d*_6_) 11.96 (1 H, s, NH‐N═C), 9.93 (1 H, s, S═C–NH–R), 8.66 (1 H, d, *J* = 2.0, Ar), 8.30 (1 H, s, HC═N), 8.24 (2 H, t, *J* = 8.5 Hz, Ar), 8.11 (1 H, d, *J* = 8.2 Hz, Ar), 8.04═7.91 (2 H, m, Ar), 7.75 (3 H, dqd, *J* = 16.3, 7.8, 3.7 Hz, Ar), 7.55 (1 H, dd, *J* = 8.1, 1.5 Hz, Ar), 7.40 (1 H, t, *J* = 8.1 Hz, Ar), 6.61 (1 H, dd, *J* = 9.1, 2.5 Hz, Ar), 5.94 (1 H, d, *J* = 2.5 Hz, Ar), 3.11 (4 H, q, *J* = 7.0 Hz, CH_2_), 0.81 (6 H, t, *J* = 6.9 Hz, CH_3_); ^13^C‐NMR (100 MHz, DMSO‐*d*_6_) *δ* 176.04 (C═S), 150.07 (C═N), 149.97 (Ar), 139.09 (Ar), 138.96 (Ar), 135.69 (Ar), 132.02 (Ar), 131.97 (Ar), 131.83 (Ar), 131.06 (Ar), 130.55 (Ar), 130.52 (Ar), 130.09 (Ar), 129.42 (Ar), 128.67 (Ar), 128.58 (Ar), 128.51 (Ar), 128.42 (Ar), 128.32 (Ar), 127.98 (Ar), 123.14 (Ar), 113.61 (Ar), 111.19 (Ar), 104.09 (Ar), 44.26 (CH_2_), 12.42 (CH_3_); FT‐IR Vmax (cm^–1^): 3261 (*N–H stretch*), 3136 (*Ar–H or N–H stretch*), 2970 (*C–H stretch, N‐ethyl*), 1611 (*C*═*N or Ar–C*═*C*), 1589 (*C*═*N or N–C*═*S*,), 1574 (*Ar–C*═*C stretch*), 1524 (*N–H bend or Ar–C*═*C*), 1498 (*Ar–C–C stretch*), 1451 (*CH₂ bend, N‐ethyl*), 1345 (*S*═*O sym. stretch, sulfonate*), 1264 (*S*═*O asym. stretch, sulfonate*), 1167 (*C–N or S–O stretch*), 1016 (*C–N stretch or Ar–C–H bend*), 956 (*N–H wag or C*═*S*,), 798 (*Ar–C–H out‐of‐plane*); HPLC: CH_3_CN:H_2_O = 80:20; *R*_t_: 7.620 min, purity: 98.7%; Elemental Analysis: CHN, Calcd: C, 55.90; H, 4.36; N, 9.31; found: C, 55.95; H, 4.39; N, 9.34; ESI‐HRMS (m/z): chemical formula: C_28_H_26_^35^Cl_2_N_4_O_3_S_2_, calcd [M‐H]^+^: 599.07451, found [M + H]^+^: 599.07455 *(0.04 ppm deviation)*, chemical formula: C_28_H_26_^37^Cl_2_N_4_O_3_S_2_, calcd [M‐H]^+^: 601.06860, found [M + H]^+^: 601.07213 *(3.53 ppm deviation)*.

(*E*)‐2‐({2‐[(4‐Bromophenyl)carbamothioyl]hydrazono}methyl)‐5‐(diethylamino)phenyl naphthalene‐2‐sulfonate (**5b**): Yield: 90%; M.P: 198°C–200°C; Color: yellow; ^1^H‐NMR: *δ* (400 MHz, DMSO‐*d*_6_) 11.78 (1 H, s, NH–N═C), 9.85 (1 H, s, S═C–NH–R), 8.65 (1 H, d, *J* = 2.0 Hz, Ar), 8.24 (3 H, dd, *J* = 15.7, 7.0 Hz, Ar, HC═N), 8.11 (1 H, d, *J* = 8.1 Hz, Ar), 8.04–7.94 (2 H, m, Ar), 7.75 (2 H, dddd, *J* = 26.4, 8.0, 6.9, 1.3 Hz, Ar), 7.63–7.47 (4 H, m, Ar), 6.59 (1 H, dd, *J* = 9.1, 2.5 Hz, Ar), 5.95 (1 H, d, *J* = 2.5 Hz, Ar), 3.11 (4 H, q, *J* = 7.0 Hz, CH_2_), 0.81 (6 H, t, *J* = 7.0 Hz, CH_3_); ^13^C‐NMR (100 MHz, DMSO‐*d*_6_) *δ* 175.34 (C═S), 149.97 (C═N), 149.91 (Ar), 139.02 (Ar), 135.67 (Ar), 131.95 (Ar), 131.88 (Ar), 131.27 (Ar), 131.02 (Ar), 130.53 (Ar), 130.09 (Ar), 129.21 (Ar), 128.57 (Ar), 128.42 (Ar), 127.75 (Ar), 123.14 (Ar), 117.64 (Ar), 113.63 (Ar), 110.99 (Ar), 104.10 (Ar), 44.27 (CH_2_), 12.43 (CH_3_); FT‐IR Vmax (cm^–1^): 3318 (*N–H stretch*), 3129 (*Ar–H or N–H stretch*), 2968 (*C–H stretch, N‐ethyl*), 1613 (*C*═*N or Ar–C*═*C*), 1583 (*C*═*N or N–C*═*S*,), 1551 (*N–H bend or Ar–C*═*C*), 1483 (*Ar–C–C stretch*), 1367 (*S*═*O sym. stretch, sulfonate*), 1266 (*S*═*O asym. stretch, sulfonate*), 1180 (*C–N or S–O stretch*), 1058 (*C–N stretch or Ar–C–H bend*), 907 (*N–H wag or C*═*S*,), 876 (*C–H out‐of‐plane, aromatic*), 756 (*Ar–C–H out‐of‐plane bend*); HPLC: CH_3_CN:H_2_O = 80: 20; *R*_t_: 5.766 min, purity: 98.01%; Elemental Analysis: CHN Calcd: C, 54.99; H, 4.45; N, 9.16; found: C, 55.23; H, 4.49; N, 9.19; ESI‐HRMS (*m/z*): chemical formula: C_28_H_27_^79^BrN_4_O_3_S_2_, calcd [M + H]^+^: 611.07862, found [M + H]^+^: 611.07625 *(4.12 ppm deviation)*, chemical formula: C_28_H_27_^81^BrN_4_O_3_S_2_, calcd [M + H]^+^: 613.07270, found [M + H]^+^: 613.07456 *(1.86 ppm deviation)*, C_28_H_27_^79^BrN_4_O_3_S_2_, calcd [M+Na]^+^: 633.06056, found [M+Na]^+^: 633.05825 *(2.31 ppm deviation)*, chemical formula: C_28_H_27_^81^BrN_4_O_3_S_2_, calcd [M+Na]^+^: 635.05460, found [M + H]^+^: 635.05629 *(1.69 ppm deviation)*.

(*E*)‐5‐(Diethylamino)‐2‐{[2‐(phenylcarbamothioyl)hydrazono]methyl}phenyl naphthalene‐2‐sulfonate (**5c**): Yield: 90%; M.P: 204°C–206°C; Color: yellow; ^1^H‐NMR: *δ* (400 MHz, DMSO‐*d*_6_) 11.71 (1 H, s, NH–N=C), 9.82 (1 H, s, S═C–NH–R), 8.66 (1 H, d, *J* = 1.9 Hz, Ar), 8.28 (1 H, s, HC═N), 8.24 (2 H, t, *J* = 8.5 Hz, Ar), 8.12 (1 H, d, *J* = 8.1 Hz, Ar), 8.05–7.95 (2 H, m, Ar), 7.78 (1 H, ddd, *J* = 8.2, 6.9, 1.3 Hz, Ar), 7.72 (1 H, ddd, *J* = 8.1, 6.9, 1.3 Hz, Ar), 7.62–7.53 (2 H, m, Ar), 7.36 (2 H, t, *J* = 7.9 Hz, Ar), 7.23–7.13 (1 H, m, Ar), 6.59 (1 H, dd, *J* = 9.1, 2.5 Hz, Ar), 5.94 (1 H, d, *J* = 2.5 Hz, Ar), 3.11 (4 H, q, *J* = 7.0 Hz, CH_2_), 0.81 (6 H, t, *J* = 7.0 Hz, CH_3_); ^13^C‐NMR (100 MHz, DMSO‐*d*_6_) *δ* 175.48 (C═S), 149.91, 149.83 (C═N), 139.57 (Ar), 138.72 (Ar), 135.67 (Ar), 131.96 (Ar), 131.93 (Ar), 131.02 (Ar), 130.54 (Ar), 130.51 (Ar), 130.09 (Ar), 129.24 (Ar), 128.57 (Ar), 128.47 (Ar), 128.42 (Ar), 125.84 (Ar), 125.49 (Ar), 123.15 (Ar), 113.75 (Ar), 111.00 (Ar), 104.09 (Ar), 44.26 (CH_2_), 12.43 (CH_3_); FT‐IR Vmax (cm^–1^): 3310 (*N–H stretch*), 3133 (*Ar–H or N–H stretch*), 2969 (*C–H stretch, N‐ethyl*), 1610 (*C*═*N or Ar–C*═*C*), 1590 (*C*═*N or N–C*═*S*,), 1548 (*N–H bend or Ar–C*═*C*), 1502 (*Ar–C–C stretch*), 1443 (*CH₂ bend, N‐ethyl*), 1360 (*S*═*O sym. stretch, sulfonate*), 1314 (*S*═*O asym. stretch, sulfonate*), 1264 (*C–N or S–O stretch*), 1180 (*C–N or S–O stretch*), 1079 (*C–N stretch or Ar–C–H bend*), 1013 (*C–N stretch or Ar–C–H bend*), 952 (*N–H wag or C*═*S*,), 840 (*C–H out‐of‐plane, aromatic*), 757 (*Ar–C–H out‐of‐plane bend*), 688 (*aromatic ring deformation*); HPLC: CH_3_CN:H_2_O = 80:20; Rt: 3.790 min, purity: 99.4805%; Elemental Analysis: CHN Calcd: C, 63.13; H, 5.30; N, 10.52; found: C, 63.17; H, 5.34; N, 10.67; ESI‐HRMS (*m/z*): chemical formula: C_28_H_28_N_4_O_3_S_2_, calcd [M + H]^+^: 533.16811, found [M + H]^+^: 533.16634 *(1.77 ppm deviation)*, calcd [M+Na]^+^: 555.15005, found [M+Na]^+^: 555.14798 *(2.07 ppm deviation)*.

(*E*)‐5‐(Diethylamino)‐2‐({2‐[(4‐methoxyphenyl)carbamothioyl]hydrazono}methyl)phenyl naphthalene‐2‐sulfonate (**5d**): Yield: 89%; M.P: 206°C–208°C; Color: off‐white; ^1^H‐NMR: *δ* (400 MHz, DMSO‐*d*_6_) 11.62 (1 H, s, NH–N═C), 9.72 (1 H, s, S═C–NH–R), 8.65 (1 H, d, *J* = 2.0 Hz, Ar), 8.30–8.17 (3 H, m, Ar, HC═N), 8.16–8.08 (1 H, m, Ar), 8.04–7.95 (2 H, m, Ar), 7.79 (1 H, ddd, *J* = 8.2, 6.9, 1.3 Hz, Ar), 7.72 (1 H, ddd, *J* = 8.1, 6.9, 1.3 Hz, Ar), 7.43–7.34 (2 H, m, Ar), 6.96–6.88 (2 H, m, Ar), 6.58 (1 H, dd, *J* = 9.1, 2.5 Hz, Ar), 5.94 (1 H, d, *J* = 2.5 Hz, Ar), 3.76 (3 H, s, O–CH_3_), 3.11 (4 H, q, *J* = 7.0 Hz, CH_2_), 0.81 (6 H, t, *J* = 7.0 Hz, CH_3_); ^13^C‐NMR (100 MHz, DMSO‐*d*_6_) *δ* 175.92 (C═S), 157.25 (C═N), 149.85 (Ar), 149.76 (Ar), 138.41 (Ar), 135.67 (Ar), 132.49 (Ar), 131.96 (Ar), 131.01 (Ar), 130.52 (Ar), 130.09 (Ar), 129.19 (Ar), 128.57 (Ar), 128.43 (Ar), 127.66 (Ar), 123.16 (Ar), 113.85 (Ar), 113.69 (Ar), 110.99 (Ar), 104.07 (Ar), 55.71 (O–CH_3_), 44.25 (CH_2_), 12.43 (CH_3_); FT‐IR Vmax (cm^–1^): 3278 (N–H stretch), 3104 (Ar–H or N–H stretch), 2964 (C–H stretch, N‐ethyl), 1611 (C═N or Ar–C═C), 1590 (C═N or N–C═S,), 1539 (N–H bend or Ar–C═C), 1509 (Ar–C–C stretch), 1435 (CH_2_ bend, N‐ethyl), 1399 (S═O sym. stretch, sulfonate), 1289 (S═O asym. stretch, sulfonate), 1235 (C–N or S–O stretch), 1184 (C–N or S–O stretch), 1099 (C–N stretch or Ar–C–H bend), 1069 (C–N stretch or Ar–C–H bend), 961 (N–H wag or C═S), 840 (C–H out‐of‐plane, aromatic), 769 (Ar–C–H out‐of‐plane bend); HPLC: CH_3_CN:H_2_O = 80:20; *R*_t_: 3.314 min, purity: 99.27%. Elemental Analysis: CHN Calcd: C, 61.90; H, 5.37; N, 9.96; found: C, 62.30; H, 5.557; N, 10.12; ESI‐HRMS (*m/z*): chemical formula: C_29_H_30_N_4_O_4_S_2_, calcd [M + H]^+^: 563.17867, found [M + H]^+^: 563,17662 *(2.05 ppm deviation)*, calcd [M+Na]^+^: 585.16062, found [M + Na]^+^: 585,15840 *(2.22 ppm deviation)*.

(*E*)‐2‐({2‐[(3‐chlorophenyl)carbamothioyl]hydrazono}methyl)‐5‐(diethylamino)phenyl naphthalene‐2‐sulfonate (**5e**): Yield: 90%; M.P: 195°C–197°C; Color: yellow; ^1^H‐NMR: *δ* (400 MHz, DMSO‐*d*_6_) 11.83 (1 H, s, NH–N═C), 9.90 (1 H, s, S═C–NH–R), 8.66 (1 H, d, *J* = 2.0 Hz, Ar), 8.29 (1 H, s, HC═N), 8.24 (2 H, t, *J* = 8.3 Hz, Ar), 8.15–8.09 (1 H, m, Ar), 8.06–7.96 (2 H, m, Ar), 7.81–7.75 (2 H, m, Ar), 7.72 (1 H, ddd, *J* = 8.1, 6.9, 1.3 Hz, Ar), 7.60 (1 H, ddd, *J* = 8.2, 2.1, 1.0 Hz, Ar), 7.38 (1 H, t, *J* = 8.1 Hz, Ar), 7.23 (1 H, ddd, *J* = 8.1, 2.2, 1.0 Hz, Ar), 6.60 (1 H, dd, *J* = 9.1, 2.5 Hz, Ar), 5.95 (1 H, d, *J* = 2.5 Hz, Ar), 3.12 (4 H, q, *J* = 7.0 Hz, CH_2_), 0.81 (6 H, t, *J* = 7.0 Hz, CH_3_); ^13^C‐NMR (100 MHz, DMSO‐*d*_6_) *δ* 175.22 (C═S), 150.01 (C═N), 149.94 (Ar), 141.08 (Ar), 139.15 (Ar), 135.68 (Ar), 132.57 (Ar), 131.96 (Ar), 131.88 (Ar), 131.02 (Ar), 130.54 (Ar), 130.50 (Ar), 130.09 (Ar), 130.00 (Ar), 129.20 (Ar), 128.56 (Ar), 128.42 (Ar), 125.08 (Ar), 124.08 (Ar), 123.15 (Ar), 113.59 (Ar), 110.99 (Ar), 104.09 (Ar), 44.28 (CH_2_), 12.42 (CH_3_); FT‐IR Vmax (cm^–1^): 3308 (*N–H stretch*), 3130 (*Ar–H or N–H stretch*), 3080 (*Ar–H stretch*), 2967 (*C–H stretch, N‐ethyl*), 2923 (*C–H stretch, aliphatic*), 1611 (*C*═*N or Ar–C*═*C*), 1584 (*C*═*N or N–C*═*S*), 1546 (*N–H bend or Ar–C*═*C*), 1503 (*Ar–C–C stretch*), 1472 (*CH₂ bend, N‐ethyl*), 1356 (*S*═*O sym. stretch, sulfonate*), 1302 (*S*═*O asym. stretch, sulfonate*), 1262 (*C–N or S–O stretch*), 1181 (*C–N or S–O stretch*), 1128 (*C–N stretch or Ar–C–H bend*), 1056 (*C–N stretch or Ar–C–H bend*), 952 (*N–H wag or C*═*S*), 851 (*C–H out‐of‐plane, aromatic*), 794 (*Ar–C–H out‐of‐plane bend*); HPLC: CH_3_CN:H_2_O = 80:20; Rt: 5.299 min, purity: 98.44%; Elemental Analysis: CHN Calcd: C, 59.30; H, 4.80; N, 9.88; found: C, 59.50; H, 4.98; N, 10.20; ESI‐HRMS (*m/z*): chemical formula: C_28_H_27_^35^ClN_4_O_3_S_2_, calcd [M + H]^+^: 567.12914, found [M + H]^+^: 567.12726 *(1.88 ppm deviation)*, chemical formula: C_28_H_27_^35^ClN_4_O_3_S_2_, calcd [M+Na]^+^: 589.11108, found [M+Na]^+^: 589.10885 *(2.23 ppm deviation)*, chemical formula: C_28_H_27_^37^ClN_4_O_3_S_2_, calcd [M + H]^+^: 569,12320, found [M + H]^+^: 569,12462 *(1.42 ppm deviation)*, chemical formula: C_28_H_27_^37^ClN_4_O_3_S_2_, calcd [M + Na]^+^: 591.10510, found [M+Na]^+^: 591.10634 *(1.24 ppm deviation)*.

(*E*)‐5‐(Diethylamino)‐2‐{[2‐(isobutylcarbamothioyl)hydrazono]methyl}phenyl naphthalene‐2‐sulfonate (**5f**): Yield: 86%; M.P: 164°C–166°C; Color: yellow; ^1^H‐NMR: *δ* (400 MHz, DMSO‐*d*_6_) 11.33 (1 H, s, NH–N=C), 8.65 (1 H, d, *J* = 2.0 Hz, S═C–NH–R), 8.27–8.15 (4 H, m, Ar, HC═N), 8.11 (1 H, dd, *J* = 8.2, 1.2 Hz, Ar), 7.98 (1 H, dd, *J* = 8.7, 2.0 Hz, Ar), 7.84 (1 H, d, *J* = 9.0 Hz, Ar), 7.78 (1 H, ddd, *J* = 8.2, 6.9, 1.3 Hz, Ar), 7.71 (1 H, ddd, *J* = 8.1, 6.9, 1.3 Hz, Ar), 6.59 (1 H, dd, *J* = 9.0, 2.5 Hz, Ar), 5.92 (1 H, d, *J* = 2.5 Hz, Ar), 3.40–3.34 (2 H, m, isopr. CH_2_), 3.10 (4 H, q, *J* = 7.0 Hz, CH_2_), 1.98 (1 H, dt, *J* = 13.6, 6.8 Hz isopr. CH), 0.87 (6 H, d, *J* = 6.7 Hz, isopr. CH_3_), 0.80 (6 H, t, *J* = 7.0 Hz, CH_3_); ^13^C‐NMR (100 MHz, DMSO‐*d*_6_) *δ* 177.13 (C═S), 149.66 (C═N), 149.58 (Ar), 137.72 (Ar), 135.65 (Ar), 132.00 (Ar), 131.95 (Ar), 130.98 (Ar), 130.49 (Ar), 130.05 (Ar), 128.75 (Ar), 128.55 (Ar), 128.40 (Ar), 123.13 (Ar), 114.03 (Ar), 111.08 (Ar), 104.12 (Ar), 51.16 (*N*‐CH_2_), 44.24 (CH_2_), 28.30 (isopr. CH_2_), 20.56 (isopr. CH_3_), 12.42 (CH_3_); FT‐IR Vmax (cm^–1^): 3350 (*N–H stretch*,), 3250 (*N–H stretch*), 3089 (*Ar–H stretch*), 2950 (*C–H stretch, N‐ethyl/aliphatic*), 1615 (*C*═*N or Ar–C*═*C*), 1598 (*C*═*N or N–C*═*S*,), 1475 (*CH*_*2*_ *bend, N‐ethyl*), 1385 (*S*═*O sym. stretch, sulfonate*), 1250 (*S*═*O asym. stretch, sulfonate*), 1145 (*C–N or S–O stretch*), 1039 (*C–N stretch or Ar–C–H bend*), 978 (*N–H wag or C*═*S*,), 854 (*C–H out‐of‐plane, aromatic*), 765 (*Ar–C–H out‐of‐plane bend*); HPLC: CH_3_CN:H_2_O = 80: 20; *R*_t_: 4.213 min, purity: 98.15%; Elemental Analysis: CHN Calcd: C, 60.91; H, 6.29; N, 10.93; found: C, 61.41; H, 6.49; N, 10.98; ESI‐HRMS (*m/z*): chemical formula: C_26_H_32_N_4_O_3_S_2_, calcd [M + H]^+^: 513.19941, found [M + H]^+^: 513.19768 *(1.73 ppm deviation)*, chemical formula: C_26_H_32_N_4_O_3_S_2_, calcd [M + Na]^+^: 535.18135, found [M + Na]^+^: 535.17948 *(1.87 ppm deviation)*.

(*E*)‐5‐(Diethylamino)‐2‐{[2‐(isobutylcarbamothioyl)hydrazono]methyl}phenyl naphthalene‐2‐sulfonate (**5g**): Yield: 87%; M.P: 133°C–135°C; Color: yellow; ^1^H‐NMR: *δ* (400 MHz, DMSO‐*d*_6_) 11.34 (1 H, s, NH–N═C), 8.64 (1 H, d, *J* = 1.9 Hz, S = C–NH–R), 8.29 (1 H, t, *J* = 5.9 Hz, Ar), 8.23 (2 H, dd, *J* = 8.6, 6.4 Hz, Ar), 8.17 (1 H, s, HC═N), 8.13–8.08 (1 H, m, Ar), 7.98 (1 H, dd, *J* = 8.8, 2.0 Hz, Ar), 7.86 (1 H, d, *J* = 9.0 Hz, Ar), 7.79 (1 H, ddd, *J* = 8.2, 6.9, 1.3 Hz, Ar), 7.71 (1 H, ddd, *J* = 8.1, 6.8, 1.3 Hz, Ar), 6.58 (1 H, dd, *J* = 9.1, 2.5 Hz, Ar), 5.93 (1 H, d, *J* = 2.5 Hz, Ar), 3.60 (2 H, q, *J* = 6.7 Hz, CH_2_), 3.10 (4 H, q, *J* = 7.0 Hz, CH_2_), 2.53–2.45 (4 H, m, CH_2_), 2.06 (3 H, s, S‐CH_3_), 1.84 (2 H, p, *J* = 7.2 Hz, CH_2_), 0.81 (6 H, t, *J* = 7.0 Hz, CH_3_); ^13^C‐NMR (100 MHz, DMSO‐*d*_6_) *δ* 176.93 (C═S), 149.70 (C═N), 149.61 (Ar), 137.70 (Ar), 135.65 (Ar), 131.95 (Ar), 130.98 (Ar), 130.49 (Ar), 130.06 (Ar), 128.66 (Ar), 128.55 (Ar), 128.40 (Ar), 123.14 (Ar), 114.02 (Ar), 111.03 (Ar), 104.09 (Ar), 44.24 (CH_2_), 43.05 (S‐CH_3_), 31.22 (CH_2_), 28.85 (CH_2_), 15.11 (CH_2_), 12.42 (CH_3_); FT‐IR Vmax (cm^–1^): 3361 (*N–H stretch*), 3148 (*Ar–H or N–H stretch*), 2969 (*C–H stretch, N‐ethyl*), 2914 (*C–H stretch, aliphatic*), 1613 (*C*═*N or Ar–C*═*C*), 1509 (*Ar–C–C stretch*), 1450 (*CH₂ bend, N‐ethyl*), 1358 (*S*═*O sym. stretch, sulfonate*), 1310 (*S*═*O asym. stretch, sulfonate*), 1262 (*C–N or S–O stretch*), 1160 (*C–N or S–O stretch*), 1065 (*C–N stretch or Ar–C–H bend*), 1014 (*C–N stretch or Ar–C–H bend*), 952 (*N–H wag or C*═*S*,), 885 (*C–H out‐of‐plane, aromatic*), 795 (*Ar–C–H out‐of‐plane bend*), 755 (*Ar–C–H out‐of‐plane bend*); HPLC: CH_3_CN:H_2_O = 80:20; *R*_t_: 3.451 min, purity: 98.32%; Elemental Analysis: CHN Calcd: C, 57.32; H, 5.92; N, 10.28; found: C, 57.84; H, 6.40; N, 10.68; ESI‐HRMS (*m/z*): chemical formula: C_26_H_32_N_4_O_3_S_3_, calcd [M + H]^+^: 545.17148, found [M + H]^+^: 545.16982 *(1.66 ppm deviation)*, calcd [M + Na]^+^: 567.15342, found [M + Na]^+^: 567.15149 *(1.93 ppm deviation)*.

(*E*)‐5‐(Diethylamino)‐2‐[(2‐{[2‐(trifluoromethyl)phenyl]carbamothioyl}hydrazineylidene) methyl]phenyl naphthalene‐2‐sulfonate (**5h**): Yield: 90%; M.P: 185°C–187°C; Color: yellow; ^1^H‐NMR: *δ* (400 MHz, DMSO‐*d*_6_) 11.94 (1 H, s, NH–N═C), 9.74 (1 H, s, S═C‐NH‐R), 8.67 (1 H, d, *J* = 2.0 Hz, Ar), 8.31 (1 H, s, HC = N), 8.25 (2 H, t, *J* = 9.1 Hz, Ar), 8.12 (1 H, d, *J* = 8.2 Hz, Ar), 7.99 (1 H, dd, *J* = 8.7, 2.0 Hz, Ar), 7.89 (1 H, d, *J* = 9.0 Hz, Ar), 7.83–7.67 (5 H, m, Ar), 7.48 (1 H, t, *J* = 7.6 Hz, Ar), 6.62 (1 H, dd, *J* = 9.1, 2.5 Hz, Ar), 5.91 (1 H, d, *J* = 2.5 Hz, Ar), 3.10 (4 H, q, *J* = 7.0 Hz, CH_2_), 0.79 (6 H, t, *J* = 7.0 Hz, CH_3_); ^13^C‐NMR (100 MHz, DMSO‐*d*_6_) δ 176.68 (C═S), 150.05 (C═N), 149.96 (Ar), 138.90 (Ar), 135.70 (Ar), 132.88 (Ar), 131.98 (Ar), 131.89 (Ar), 131.59 (Ar), 131.05 (Ar), 130.56 (Ar), 130.53 (Ar), 130.08 (Ar), 128.58 (Ar), 128.42 (Ar), 127.23 (Ar), 126.50 (Ar), 125.41 (Ar), 123.12 (Ar), 113.63 (Ar), 111.25 (Ar), 104.07 (Ar), 44.23 (CH_2_), 12.40 (CH_3_); FT‐IR Vmax (cm^–1^): 3305 (*N–H stretch*), 2798 (*C–H stretch, aliphatic*), 1610 (*C*═*N or Ar–C*═*C*), 1588 (*C*═*N or N–C*═*S*,), 1554 (*N–H bend or Ar–C*═*C*), 1511 (*Ar–C–C stretch*), 1457 (*CH₂ bend, N‐ethyl*), 1371 (*S*═*O sym. stretch, sulfonate*), 1314 (*S*═*O asym. stretch, sulfonate*), 1271 (*C–N or S–O stretch; possible C–F stretch contribution*), 1186 (*C–N or S–O stretch*), 1096 (*C–N stretch or Ar–C–H bend*), 1063 (*C–N stretch or Ar–C–H bend*), 956 (*N–H wag or C*═*S*), 755 (*Ar–C–H out‐of‐plane bend*), 545 (*S–O bending or sulfonate fingerprint*); HPLC: CH_3_CN:H_2_O = 80:20; *R*_t_: 5.205 min, purity: 96.52%; Elemental Analysis: CHN Calcd: C, 57.99; H, 4.53; N, 9.33; found: C, 58.40; H, 4.87; N, 9.45; ESI‐HRMS (*m/z*): chemical formula: C_29_H_27_F_3_N_4_O_3_S_2_, calcd [M + H]^+^: 601.15549, found [M + H]^+^: 601.15356 *(1.93 ppm deviation)*, calcd [M + Na]^+^: 623.13744, found [M + Na]^+^: 623.13537 *(2.07 ppm deviation)*.

(*E*)‐2‐({2‐[(4‐Chlorophenyl)carbamothioyl]hydrazono}methyl)‐5‐(diethylamino)phenyl naphthalene‐2‐sulfonate (**5i**): Yield: 92%; M.P: 199°C–201°C; Color: pale yellow; ^1^H‐NMR: *δ* (400 MHz, DMSO‐*d*_6_) 11.78 (1 H, s, NH–N═C), 9.86 (1 H, s, S═C–NH–R), 8.65 (1 H, d, *J* = 2.0 Hz, Ar), 8.27 (1 H, s, HC═N), 8.24 (2 H, t, *J* = 8.3 Hz, Ar), 8.11 (1 H, d, *J* = 8.1 Hz, Ar), 8.05–7.94 (2 H, m, Ar), 7.78 (1 H, ddd, *J* = 8.1, 6.9, 1.3 Hz, Ar), 7.76–7.68 (1 H, m, Ar), 7.67–7.58 (2 H, m, Ar), 7.47–7.34 (2 H, m, Ar), 6.59 (1 H, dd, *J* = 9.1, 2.5 Hz, Ar), 5.95 (1 H, d, *J* = 2.5 Hz, Ar), 3.11 (4 H, q, *J* = 7.0 Hz, CH_2_), 0.81 (6 H, t, *J* = 7.0 Hz, CH_3_); ^13^C‐NMR (100 MHz, DMSO‐*d*_6_) *δ* 175.43 (C═S), 149.97 (C═N), 149.90 (Ar), 139.00 (Ar), 138.57 (Ar), 135.68 (Ar), 131.96 (Ar), 131.89 (Ar), 131.02 (Ar), 130.53 (Ar), 130.09 (Ar), 129.43 (Ar), 129.20 (Ar), 128.57 (Ar), 128.42 (Ar), 128.34 (Ar), 127.44 (Ar), 123.15 (Ar), 113.65 (Ar), 110.99 (Ar), 104.09 (Ar), 44.26 (CH_2_), 12.43 (CH_3_). FT‐IR Vmax (cm^–1^): 3314 (*N–H stretch*), 3132 (*Ar–H or N–H stretch*), 2970 (*C–H stretch, N‐ethyl*), 1613 (*C*═*N or Ar–C*═*C*), 1587 (*C*═*N or N–C*═*S*,), 1550 (*N–H bend or Ar–C*═*C*), 1509 (*Ar–C–C stretch*), 1406 (*CH₂ bend, N‐ethyl*), 1367 (*S*═*O sym. stretch, sulfonate*), 1314 (*S*═*O asym. stretch, sulfonate*), 1180 (*C–N or S–O stretch*), 1132 (*C–N or S–O stretch*), 1080 (*C–N stretch or Ar–C–H bend*), 1012 (*C–N stretch or Ar–C–H bend*), 952 (*N–H wag or C*═*S*), 907 (*C–H out‐of‐plane, aromatic*), 757 (*Ar–C–H out‐of‐plane bend*), 690 (*aromatic ring deformation*); HPLC: CH_3_CN:H_2_O = 80: 20; Rt: 5.440 min, purity: 98.10%; Elemental Analysis: CHN Calcd: C, 59.30; H, 4.80; N, 9.88; found: C, 59.54; H, 4.94; N, 9.97; ESI‐HRMS (m/z): chemical formula: C_28_H_27_^35^ClN_4_O_3_S_2_, calcd [M + H]^+^: 567.12914, found [M + H]^+^: 567.12717 *(1.97 ppm deviation)*, calcd [M+Na]^+^: 589.11108, found [M+Na]^+^: 589.10886 *(2.22 ppm deviation)*, chemical formula: C_28_H_27_^37^ClN_4_O_3_S_2_, calcd [M + H]^+^: 569.12320, found [M + H]^+^: 569.12464 *(1.44 ppm deviation)*, calcd [M + Na]^+^: 591.10510, found [M + Na]^+^: 591.10654 *(1.44 ppm deviation)*.

(*E*)‐5‐(Diethylamino)‐2‐({2‐[(2,6‐dimethylphenyl)carbamothioyl]hydrazono}methyl)phenyl naphthalene‐2‐sulfonate (**5j**): Yield: 86%; M.P: 219°C–221°C; Color: off‐white; ^1^H‐NMR: *δ* (400 MHz, DMSO‐*d*_6_) 11.62 (1 H, s, NH‐N═C), 9.60 (1 H, s, S═C‐NH‐R), 8.68 (1 H, d, *J* = 2.0 Hz, Ar), 8.25 (3 H, dd, *J* = 16.0, 7.7 Hz, Ar), 8.09 (2 H, dd, *J* = 20.5, 8.6 Hz, Ar), 8.00 (1 H, dd, *J* = 8.7, 2.0 Hz, Ar), 7.80 (1 H, t, *J* = 7.5 Hz, Ar), 7.72 (1 H, t, *J* = 7.5 Hz, Ar), 7.20–7.02 (3 H, m, Ar), 6.55 (1 H, dd, *J* = 9.0, 2.5 Hz, Ar), 5.90 (1 H, d, *J* = 2.5 Hz, Ar), 3.09 (4 H, q, *J* = 7.0 Hz, CH_2_), 2.16 (6 H, s, CH_3_), 0.79 (6 H, t, *J* = 6.9 Hz, CH_3_); ^13^C‐NMR (100 MHz, DMSO‐*d*_6_) *δ* 176.59 (C═S), 149.78 (C═N), 149.64 (Ar), 137.87 (Ar), 137.72 (Ar), 136.95 (Ar), 135.68 (Ar), 131.98 (Ar), 131.05 (Ar), 130.52 (Ar), 130.07 (Ar), 129.09 (Ar), 128.58 (Ar), 128.42 (Ar), 127.97 (Ar), 127.22 (Ar), 123.19 (Ar), 114.13 (Ar), 111.05 (Ar), 104.00 (Ar), 44.22 (CH_2_), 18.56 (CH_3_), 12.42 (CH_3_); FT‐IR Vmax (cm^–1^): 3295 (*N–H stretch*), 3094 (*Ar–H or N–H stretch*), 2966 (*C–H stretch, N‐ethyl*), 1612 (*C*═*N or Ar–C*═*C*), 1590 (*C*═*N or N–C*═*S*), 1509 (*Ar–C–C stretch*), 1435 (*CH₂ bend, N‐ethyl*), 1395 (*S*═*O sym. stretch, sulfonate*), 1284 (*S*═*O asym. stretch, sulfonate*), 1262 (*C–N or S–O stretch*), 1187 (*C–N or S–O stretch*), 1068 (*C–N stretch or Ar–C–H bend*), 1017 (*C–N stretch or Ar–C–H bend*), 965 (*N–H wag or C*═*S*), 855 (*C–H out‐of‐plane, aromatic*), 759 (*Ar–C–H out‐of‐plane bend*), 548 (*S–O bending or sulfonate fingerprint*); HPLC: CH_3_CN:H_2_O = 80:20; *R*_t_: 4.186 min, purity: 99.77%; Elemental Analysis: CHN Calcd: C, 64.26; H, 5.75; N, 9.99; found: C, 64.45; H, 5.93; N, 10.46; ESI‐HRMS (*m/z*): chemical formula: C_30_H_32_N_4_O_3_S_2_, calcd [M + H]^+^: 561.19941, found [M + H]^+^: 561.19732 *(2.09 ppm deviation)*, calcd [M + Na]^+^: 583.18135, found [M + Na]^+^: 583.17899 *(2.36 ppm deviation)*.

(*E*)‐5‐(Diethylamino)‐2‐({2‐[(3‐methoxyphenyl)carbamothioyl]hydrazono}methyl)phenyl naphthalene‐2‐sulfonate (**5k**): Yield: 90%; M.P: 185°C–187°C; Color: pale yellow; ^1^H‐NMR: *δ* (400 MHz, DMSO‐*d*_6_) 11.72 (1 H, s, NH–N═C), 9.77 (1 H, s, S═C–NH–R), 8.66 (1 H, d, *J* = 2.0 Hz, Ar), 8.25 (3 H, dd, *J* = 15.9, 7.0 Hz, Ar), 8.11 (1 H, d, *J* = 8.1 Hz, Ar), 8.05–7.94 (2 H, m, Ar), 7.83–7.67 (2 H, m, Ar), 7.31 (1 H, t, *J* = 2.2 Hz, Ar), 7.28–7.17 (2 H, m, Ar), 6.76 (1 H, dd, *J* = 8.2, 2.5 Hz, Ar), 6.59 (1 H, dd, *J* = 9.1, 2.5 Hz, Ar), 5.94 (1 H, d, *J* = 2.5, Ar), 3.76 (3 H, s, O‐CH_3_, 3.11 (4 H, q, *J* = 7.0 Hz, CH_2_), 0.81 (6 H, t, *J* = 7.0 Hz, CH_3_). ^13^C‐NMR (100 MHz, DMSO‐*d*_6_) *δ* 175.16 (C═S), 159.45 (C═N), 149.92 (Ar), 149.85 (Ar), 140.67 (Ar), 138.81 (Ar), 135.67 (Ar), 131.96 (Ar), 131.93 (Ar), 131.02 (Ar), 130.53 (Ar), 130.51 (Ar), 130.08 (Ar), 129.28 (Ar), 129.16 (Ar), 128.57, 128.42 (Ar), 123.15 (Ar), 117.67 (Ar), 113.68 (Ar), 111.17 (Ar), 111.01 (Ar), 110.93 (Ar), 104.10 (Ar), 55.60 (O‐CH_3_), 44.27 (CH_2_), 12.42 (CH_3_); FT‐IR Vmax (cm^–1^): 3309 (*N–H stretch*), 3126 (*Ar–H or N–H stretch*), 2965 (*C–H stretch, N‐ethyl*), 2833 (*C–H stretch, aliphatic*), 1610 (*C*═*N or Ar–C*═*C*), 1592 (*C*═*N or N–C*═*S*), 1547 (*N–H bend or Ar–C*═*C*), 1511 (*Ar–C–C stretch*), 1453 (*CH*_*2*_ *bend, N‐ethyl*), 1407 (*CH*_*2*_ *or S*═*O sym. stretch*), 1365 (*S*═*O sym. stretch, sulfonate*), 1265 (*S*═*O asym. stretch, sulfonate*), 1181 (*C–N or S–O stretch*), 1080 (*C–N stretch or Ar–C–H bend*), 1042 (*C–N stretch or Ar–C–H bend*), 952 (*N–H wag or C*═*S*,), 859 (*C–H out‐of‐plane, aromatic*), 759 (*Ar–C–H out‐of‐plane bend*), 649 (*aromatic ring deformation or S–O bend*); HPLC: CH3CN: H2O = 80: 20; tR: 3.928 min, purity: 99.42%; Elemental Analysis: CHN Calcd: C, 61.90; H, 5.37; N, 9.96; found: C, 62.45; H, 5.67; N, 10.14; ESI‐HRMS (*m/z*): chemical formula: C_29_H_30_N_4_O_4_S_2_, calcd [M + H]^+^: 563.17867, found [M + H]^+^: 563.17684 *(1.83 ppm deviation)*, calcd [M + Na]^+^: 585.16062, found [M + Na]^+^: 585.15858 *(2.04 ppm deviation)*.

(*E*)‐5‐(Diethylamino)‐2‐({2‐[(4‐fluorophenyl)carbamothioyl]hydrazono}methyl)phenyl naphthalene‐2‐sulfonate (**5l**): Yield: 95%; M.P: 183°C–185°C; Color: yellow; ^1^H‐NMR: *δ* (400 MHz, DMSO‐*d*_6_) 11.72 (1 H, s, NH–N═C), 9.83 (1 H, s, S═C–NH–R), 8.66 (1 H, d, *J* = 1.9 Hz, Ar), 8.31–8.20 (3 H, m, Ar), 8.15–8.07 (1 H, m, Ar), 8.05–7.95 (2 H, m, Ar), 7.79 (1 H, ddd, *J* = 8.2, 6.9, 1.3 Hz, Ar), 7.72 (1 H, ddd, *J* = 8.1, 6.8, 1.3 Hz, Ar), 7.59–7.48 (2 H, m, Ar), 7.25–7.14 (2 H, m, Ar), 6.58 (1 H, dd, *J* = 9.1, 2.5 Hz, Ar), 5.94 (1 H, d, *J* = 2.5 Hz, Ar), 3.11 (4 H, q, *J* = 7.0 Hz, CH_2_), 0.81 (6 H, t, *J* = 7.0 Hz, CH_3_); ^13^C‐NMR (100 MHz, DMSO‐*d*_6_) *δ* 175.86 (C═S), 161.16 (C═N), 158.76 (Ar), 149.93 (Ar), 149.85 (Ar), 138.77 (Ar), 135.95 (Ar), 135.92 (Ar), 135.68 (Ar), 131.96 (Ar), 131.92 (Ar), 131.02 (Ar), 130.53 (Ar), 130.09 (Ar), 129.17 (Ar), 128.57 (Ar), 128.43 (Ar), 128.20 (Ar), 128.12 (Ar), 123.15 (Ar), 115.20 (Ar), 114.98 (Ar), 113.74 (Ar), 110.98 (Ar), 104.08 (Ar), 44.26 (CH_2_), 12.43 (CH_3_); FT‐IR Vmax (cm^−1^): 3314 (*N–H stretch*), 3133 (*Ar–H or N–H stretch*), 2967 (*C–H stretch, N‐ethyl*), 2920 (*C–H stretch, aliphatic*), 1610 (*C*═*N or Ar–C*═*C*), 1551 (*N–H bend or Ar–C*═*C*), 1504 (*Ar–C–C stretch*), 1404 (*CH*_*2*_ *bend, N‐ethyl*), 1363 (*S*═*O sym. stretch, sulfonate*), 1301 (*S*═*O asym. stretch, sulfonate*), 1266 (*C–N or S–O stretch*), 1180 (*C–N or S–O stretch*), 1057 (*C–N stretch or Ar–C–H bend*), 950 (*N–H wag or C*═*S*), 906 (*C–H out‐of‐plane, aromatic*), 860 (*C–H out‐of‐plane, aromatic*), 758 (*Ar–C–H out‐of‐plane bend*), 650 (*aromatic ring deformation or S–O bend*); HPLC: CH_3_CN:H_2_O = 80:20; *R*_t_: 3.838 min, purity: 99.61%; Elemental Analysis: CHN Calcd: C, 61.07; H, 4.94; N, 10.17; found: C, 61.45; H, 5.43; N, 10.67; ESI‐HRMS (*m/z*): chemical formula: C_28_H_27_FN_4_O_3_S_2_, calcd [M + H]^+^: 551.15869, found [M + H]^+^: 551.15653 *(2.16 ppm deviation)*, calcd [M + Na]^+^: 573.14063, found [M + Na]^+^: 573.13842 *(2.21 ppm deviation)*.

(*E*)‐5‐(Diethylamino)‐2‐{[2‐(methylcarbamothioyl)hydrazono]methyl}phenyl naphthalene‐2‐sulfonate (**5m**): Yield: 93%; M.P: 205°C–207°C; Color: off‐white; ^1^H‐NMR: *δ* (400 MHz, DMSO‐*d*_6_) 11.35 (1 H, s, NH–N═C), 8.64 (1 H, d, *J* = 1.9 Hz, S═C–NH–R), 8.30–8.14 (4 H, m, Ar), 8.11 (1 H, d, *J* = 8.2 Hz, Ar), 7.98 (1 H, dd, *J* = 8.8, 2.0 Hz, Ar), 7.87 (1 H, d, *J* = 9.0 Hz, Ar), 7.75 (2 H, dt, *J* = 29.8, 7.3 Hz, Ar), 6.58 (1 H, dd, *J* = 9.1, 2.6 Hz, Ar), 5.93 (1 H, d, *J* = 2.6 Hz, Ar), 3.10 (4 H, q, *J* = 7.0 Hz, CH_2_), 2.97 (3 H, d, *J* = 4.5 Hz, *N*‐CH_3_), 0.81 (6 H, t, *J* = 6.9 Hz, CH_3_); ^13^C‐NMR (100 MHz, DMSO‐*d*_6_) *δ* 177.63 (C═S), 149.70 (C═N), 149.59 (Ar), 137.42 (Ar), 135.65 (Ar), 131.94 (Ar), 130.98 (Ar), 130.49 (Ar), 130.07 (Ar), 128.56 (Ar), 128.48 (Ar), 128.41 (Ar), 123.13 (Ar), 114.09 (Ar), 111.05 (Ar), 104.05 (Ar), 44.22 (CH_2_), 31.14 (*N*‐CH_3_), 12.43 (CH_3_); FT‐IR Vmax (cm^−1^): 3343 (*N–H stretch*), 3134 (*Ar–H or N–H stretch*), 2964 (*C–H stretch, N‐ethyl*), 1615 (*C*═*N or Ar–C*═*C*), 1548 (*N–H bend or Ar–C*═*C*), 1515 (*Ar–C–C stretch*), 1470 (*CH₂ bend, N‐ethyl*), 1443 (*CH*_*2*_ *or C–N bend*), 1350 (*S*═*O sym. stretch, sulfonate*), 1260 (*S*═*O asym. stretch, sulfonate*), 1187 (*C–N or S–O stretch*), 1130 (*C–N or S–O stretch*), 1016 (*C–N stretch or Ar–C–H bend*), 955 (*N–H wag or C*═*S*), 885 (*C–H out‐of‐plane, aromatic*), 815 (*C–H out‐of‐plane, aromatic*), 758 (*Ar–C–H out‐of‐plane bend*); HPLC: CH_3_CN:H_2_O = 80:20; *R*_t_: 2.489 min, purity: 96.03%; Elemental Analysis: CHN Calcd: C, 58.70; H, 5.57; N, 11.91; found: C, 58.94; H, 5.77; N, 12.30; ESI‐HRMS (*m/z*): chemical formula: C_23_H_26_N_4_O_3_S_2_, calcd [M + H]^+^: 471.15246, found [M + H]^+^: 471.15087 *(1.59 ppm deviation)*, calcd [M + Na]^+^: 493.13440, found [M + Na]^+^: 493.13249 *(0.91 ppm deviation)*.

(*E*)‐2‐[(2‐Carbamothioylhydrazono)methyl]‐5‐(diethylamino)phenyl naphthalene‐2‐sulfonate (**5n**): Yield: 91%; M.P: 245°C–247°C; Color: off‐white; ^1^H‐NMR: *δ* (400 MHz, DMSO‐*d*_6_) 11.33 (1 H, s, NH–N═C), 8.65 (1 H, d, *J* = 1.9 Hz, S = C‐NH‐R), 8.27– 8.21 (2 H, m, Ar), 8.19 (1 H, s, HC═N), 8.12 (1 H, dd, *J* = 8.1, 1.2 Hz, Ar), 8.04–7.96 (2 H, m, Ar), 7.88 (1 H, d, *J* = 9.0 Hz, Ar), 7.79 (1 H, ddd, *J* = 8.2, 6.8, 1.3 Hz, Ar), 7.72 (2 H, ddd, *J* = 8.1, 5.8, 1.3 Hz, Ar), 6.57 (1 H, dd, *J* = 9.1, 2.6 Hz, Ar), 5.90 (1 H, d, *J* = 2.5 Hz, Ar), 3.09 (4 H, q, *J* = 7.0 Hz, CH_2_), 0.79 (6 H, t, *J* = 7.0 Hz, CH_3_); ^13^C‐NMR (101 MHz, DMSO‐*d*_6_) *δ* 177.70 (C═S), 149.75 (C═N), 149.64 (Ar), 138.05 (Ar), 135.66 (Ar), 131.99 (Ar), 131.95 (Ar), 131.00 (Ar), 130.51 (Ar), 130.06 (Ar), 128.67 (Ar), 128.57 (Ar), 128.42 (Ar), 123.14 (Ar), 113.99 (Ar), 111.09 (Ar), 103.99 (Ar), 44.21 (CH_2_), 12.42 (CH_3_); FT‐IR Vmax (cm^–1^): 3299 (*N–H stretch*), 3131 (*N–H stretch, NH or Ar–H*), 3088 (*Ar–H stretch*), 2964 (*C–H stretch, N‐ethyl*), 2922 (*C–H stretch, aliphatic*), 2853 (*C–H stretch, aliphatic*), 1612 (*C*═*N or Ar–C*═*C stretch*), 1588 (*N–C*═*S or C*═ *N*,), 1507 (*Ar–C–C stretch*), 1407 (*CH₂ bend, N‐ethyl*), 1375 (*S*═*O sym. stretch, sulfonate*), 1338 (*C–N or S*═*O bend*), 1306 (*S*═*O asym. stretch, sulfonate*), 1201 (*C–N or S–O stretch*), 1127 (*C–N or S–O stretch*), 1079 (*C–N stretch or Ar–C–H bend*), 953 (*N–H wag or C*═*S*), 888 (*C–H out‐of‐plane, aromatic*), 757 (*Ar–C–H out‐of‐plane bend*); HPLC: CH_3_CN:H_2_O = 80:20; *R*_t_: 1.948 min, purity: 99.38%; Elemental Analysis: CHN Calcd: C, 57.87; H, 5.30; N, 12.27; found: C, 57.93; H, 5.46; N, 12.48; ESI‐HRMS (*m/z*): chemical formula: C_22_H_24_N_4_O_3_S_2_, calcd [M + H]^+^: 457.13681, found [M + H]^+^: 457.13494 *(1.87 ppm deviation)*, calcd [M + Na]^+^: 479.11875, found [M + Na]^+^: 479.11701 *(1.74 ppm deviation)*.

(*E*)‐5‐(Diethylamino)‐2‐({2‐[(3‐nitrophenyl)carbamothioyl]hydrazono}methyl)phenyl naphthalene‐2‐sulfonate (**5o**): Yield: 94%; M.P: 176°C–178°C; Color: yellow; ^1^H‐NMR: *δ* (400 MHz, DMSO‐*d*_6_) 11.97 (1 H, s, NH‐N═C), 10.13 (1 H, s, S═C‐NH‐R), 8.66 (2 H, d, *J* = 2.0 Hz, Ar), 8.32 (1 H, s, HC = N), 8.24 (2 H, t, *J* = 8.2 Hz, Ar), 8.11 (2 H, d, *J* = 8.1 Hz, Ar), 8.08–7.95 (3 H, m, Ar), 7.83–7.69 (2 H, m, Ar), 7.64 (1 H, t, *J* = 8.2 Hz, Ar), 6.62 (1 H, dd, *J* = 9.1, 2.6 Hz, Ar), 5.99 (1 H, d, *J* = 2.6 Hz, Ar), 3.13 (4 H, q, *J* = 7.1 Hz, CH_2_), 0.83 (6 H, t, *J* = 6.9 Hz, CH_3_); ^13^C‐NMR (100 MHz, DMSO‐*d*_6_) *δ* 175.26 (C═S), 150.10 (C═N), 150.04 (Ar), 147.72 (Ar), 140.84 (Ar), 139.56 (Ar), 135.68 (Ar), 131.96 (Ar), 131.83 (Ar), 131.73 (Ar), 131.03 (Ar), 130.53 (Ar), 130.49 (Ar), 130.09 (Ar), 129.60 (Ar), 129.16 (Ar), 128.55 (Ar), 128.42 (Ar), 123.14 (Ar), 119.78 (Ar), 119.69 (Ar), 113.49 (Ar), 111.01 (Ar), 104.14 (Ar), 44.29 (CH_2_), 12.42 (CH_3_); FT‐IR Vmax (cm^–1^): 3298 (*N–H stretch*), 3131 (*N–H stretch, NH or Ar–H*), 2964 (*C–H stretch, N‐ethyl*), 2921 (*C–H stretch, aliphatic*), 2854 (*C–H stretch, aliphatic*), 1612 (*C*═*N or Ar–C*═*C stretch*), 1589 (*N–C*═*S or NO₂ asym. stretch*), 1508 (*Ar–C–C stretch or NO₂ asym. stretch*), 1406 (*CH₂ bend, N‐ethyl*), 1374 (*NO₂ sym. stretch*), 1307 (*S*═*O asym. stretch, sulfonate*), 1264 (*C–N or S–O stretch*), 1061 (*C–N stretch or Ar–C–H bend*), 1009 (*C–N stretch or Ar–C–H bend*), 953 (*N–H wag or C*═*S*), 862 (*C–H out‐of‐plane, aromatic*), 757 (*Ar–C–H out‐of‐plane bend*); HPLC: CH_3_CN:H_2_O = 80:20; *R*_t_: 3.987 min, purity: 98.53%; Elemental Analysis: CHN Calcd: C, 58.22; H, 4.71; N, 12.12; found: C, 58.46; H, 4.93; N, 12.43; ESI‐HRMS (*m/z*): chemical formula: C_28_H_27_N_5_O_5_S_2_, calcd [M + H]^+^: 578.15319, found [M + H]^+^: 578.15113 *(2.06 ppm deviation)*, calcd [M + Na]^+^: 600.13513, found [M + Na]^+^: 600.1299 *(2.14 ppm deviation)*.

(*E*)‐2‐{[2‐(Cyclohexylcarbamothioyl)hydrazono]methyl}‐5‐(diethylamino)phenyl naphthalene‐2‐sulfonate (**5p**): Yield: 90%; M.P: 194°C–196°C; Color: pale yellow; ^1^H‐NMR: *δ* (400 MHz, DMSO‐*d*_6_) 11.29 (1 H, s, NH‐N═C), 8.64 (1 H, d, *J* = 2.0 Hz, S═C–NH–R), 8.22 (2 H, t, *J* = 8.8 Hz, Ar), 8.15 (1 H, s, HC═N), 8.11 (1 H, d, *J* = 8.2 Hz, Ar), 7.97 (1 H, dd, *J* = 8.7, 2.0 Hz, Ar), 7.83–7.68 (4 H, m, Ar), 6.58 (1 H, dd, *J* = 9.1, 2.5 Hz, Ar), 5.90 (1 H, d, *J* = 2.5 Hz, Ar), 4.16 (1 H, dp, *J* = 15.6, 5.7, 4.5 Hz, Cy. Hex), 3.08 (4 H, q, *J* = 7.0 Hz, CH_2_), 1.93–1.79 (2 H, m, Cy. Hex), 1.71 (2 H, dt, *J* = 12.7, 3.3 Hz, Cy. Hex), 1.60 (1 H, d, *J* = 12.7 Hz, Cy. Hex), 1.46–1.20 (4 H, m, Cy. Hex), 1.20–1.04 (1 H, m, Cy. Hex), 0.79 (6 H, t, *J* = 7.0 Hz, CH_3_); ^13^C‐NMR (100 MHz, DMSO‐*d*_6_) *δ* 175.59 (C═S), 149.58 (C═N), 138.17 (Ar), 135.64 (Ar), 132.06 (Ar), 131.94 (Ar), 130.95 (Ar), 130.51 (Ar), 130.49 (Ar), 130.05 (Ar), 129.21 (Ar), 128.56 (Ar), 128.40 (Ar), 123.12 (Ar), 113.87 (Ar), 111.01 (Ar), 104.18(Ar), 52.82 (*N*‐Cy. Hex), 44.25 (CH_2_), 32.42 (Cy. Hex), 25.63 (Cy. Hex), 25.35 (Cy. Hex), 12.39 (CH_3_); FT‐IR Vmax (cm^–1^): 3338 (*N–H stretch*), 3125 (*Ar–H or N–H stretch*), 2973 (*C–H stretch, N‐ethyl*), 2927 (*C–H stretch, aliphatic*), 1616 (*C*═*N or Ar–C*═*C stretch*), 1541 (*N–H bend or Ar–C*═*C*), 1508 (*Ar–C–C stretch*), 1448 (*CH₂ bend, N‐ethyl*), 1407 (*CH₂ or S*═*O sym. stretch, sulfonate*), 1373 (*S*═*O sym. stretch, sulfonate*), 1294 (*S*═*O asym. stretch, sulfonate*), 1214 (*C–N or S–O stretch*), 1183 (*C–N or S–O stretch*), 1089 (*C–N stretch or Ar–C–H bend*), 986 (*C–H out‐of‐plane, aromatic*), 953 (*N–H wag or C*═*S*), 861 (*C–H out‐of‐plane, aromatic*), 753 (*Ar–C–H out‐of‐plane bend*); HPLC: CH_3_CN:H_2_O = 80:20; *R*_t_: 6.061 min, purity: 99.24%; Elemental Analysis: CHN Calcd: C, 62.43; H, 6.36; N, 10.40; found: C, 62.64; H, 6.57; N, 10.66. ESI‐HRMS (*m/z*): chemical formula: C_28_H_34_N_4_O_3_S_2_, calcd [M + H]^+^: 539.21506, found [M + H]^+^: 539.21319 *(1.87 ppm deviation)*, calcd [M + Na]^+^: 561.19700, found [M + Na]^+^: 561.19477 *(2.23 ppm deviation)*.

(*E*)‐2‐({2‐[(4‐Chlorobenzyl)carbamothioyl]hydrazono}methyl)‐5‐(diethylamino)phenyl naphthalene‐2‐sulfonate (**5q**): Yield: 90%; M.P: 198°C–200°C; Color: pale yellow; ^1^H‐NMR: *δ* (400 MHz, DMSO‐*d*_6_) 11.51 (1 H, s, NH‐N°C═C), 8.83 (1 H, t, *J* = 6.3 Hz, S═C–NH–R), 8.65 (1 H, d, *J* = 2.0 Hz, Ar), 8.23 (3 H, q, *J* = 5.1 Hz, Ar, HC = N), 8.11 (1 H, d, *J* = 8.1 Hz, Ar), 7.97 (1 H, dd, *J* = 8.8, 2.0 Hz, Ar), 7.90 (1 H, d, *J* = 9.0 Hz, Ar), 7.79 (1 H, ddd, *J* = 8.2, 6.9, 1.3 Hz, Ar), 7.72 (1 H, ddd, *J* = 8.2, 6.9, 1.3 Hz, Ar), 7.44–7.30 (4 H, m, Ar), 6.56 (1 H, dd, *J* = 9.0, 2.5 Hz, Ar), 5.94 (1 H, d, *J* = 2.5 Hz, Ar), 4.78 (2 H, d, *J* = 6.2 Hz, *N*‐CH_2_), 3.10 (4 H, q, *J* = 7.0 Hz, CH_2_), 0.80 (6 H, t, *J* = 7.0 Hz, CH_3_); ^13^C‐NMR (101 MHz, DMSO‐*d*_6_) *δ* 177.44 (C═S), 149.80 (C═N), 149.68 (Ar), 139.12 (Ar), 138.01 (Ar), 135.66 (Ar), 131.95 (Ar), 131.89 (Ar), 131.67 (Ar), 131.02 (Ar), 130.49 (Ar), 130.07 (Ar), 129.58 (Ar), 128.54 (Ar), 128.41 (Ar), 123.16 (Ar), 113.94 (Ar), 111.02 (Ar), 104.06 (Ar), 46.28 (*N*‐CH_2_), 44.24 (CH_2_), 12.42 (CH_3_); FT‐IR Vmax (cm^–1^): 3357 (*N–H stretch*), 3146 (*N–H or Ar–H stretch*), 2370 (*possible overtone or combination band*), 2908 (*C–H stretch, aliphatic*), 1615 (*C*═*N or Ar–C*═*C stretch*), 1516 (*N–H bend or Ar–C*═*C*), 1487 (*Ar–C–C stretch*), 1409 (*CH₂ bend, N‐ethyl*), 1301 (*S*═*O asym. stretch, sulfonate*), 1216 (*C–N or S–O stretch*), 1085 (*C–N stretch or Ar–C–H bend*), 1013 (*C–N stretch or Ar–C–H bend*), 958 (*N–H wag or C*═*S*), 856 (*C–H out‐of‐plane, aromatic*), 759 (*Ar–C–H out‐of‐plane bend*), 545 (*possibly S–S or other bending mode*); HPLC: CH_3_CN:H_2_O = 80:20, *R*_t_: 5.011 min, purity: 96.94%; Elemental Analysis: CHN Calcd: C, 59.93; H, 5.03; N, 9.64; found: C, 60.23; H, 5.67; N, 9.87; ESI‐HRMS (*m/z*): chemical formula: C_29_H_29_^35^ClN_4_O_3_S_2_, calcd [M + H]^+^: 581.14479, found [M + H]^+^: 581.14272 *(2.07 ppm deviation)*, calcd [M+Na]^+^: 603.12673, found [M + Na]^+^: 603.12440 *(2.33 ppm deviation)*, chemical formula: C_29_H_29_^37^ClN_4_O_3_S_2_, calcd [M + H]^+^: 583.13890, found [M + H]^+^: 583.14021 *(1.31 ppm deviation)*, calcd [M + Na]^+^: 605.12080, found [M + Na]^+^: 603.12220 *(1.40 ppm deviation)*.

(*E*)‐5‐(Diethylamino)‐2‐{[2‐(o‐tolylcarbamothioyl)hydrazono]methyl}phenyl naphthalene‐2‐sulfonate (**5r**): Yield: 93%; M.P: 189°C–191°C; Color: off‐white. ^1^H‐NMR: *δ* (400 MHz, DMSO‐*d*_6_) 11.68 (1 H, s, NH–N═C), 9.69 (1 H, s, S═C–NH–R), 8.67 (1 H, d, *J* = 2.0 Hz, Ar), 8.25 (3 H, dd, *J* = 17.0, 8.5 Hz, Ar, HC═N), 8.12 (1 H, d, *J* = 8.2 Hz, Ar), 8.08–7.94 (2 H, m, Ar), 7.79 (1 H, ddd, *J* = 8.2, 6.9, 1.3 Hz, Ar), 7.72 (1 H, ddd, *J* = 8.2, 6.9, 1.3 Hz, Ar), 7.32 (1 H, dd, *J* = 7.3, 1.9 Hz, Ar), 7.22 (3 H, dtt, *J* = 19.0, 7.3, 3.9 Hz, Ar), 6.57 (1 H, dd, *J* = 9.1, 2.5 Hz, Ar), 5.93 (1 H, d, *J* = 2.5 Hz, Ar), 3.34 (3 H, s, CH_3_), 3.10 (4 H, q, *J* = 7.0 Hz, CH_2_), 0.80 (6 H, t, *J* = 6.9 Hz, CH_3_); ^13^C‐NMR (100 MHz, DMSO‐*d*_6_) *δ* 176.46 (C═S), 149.86 (C═N), 149.73 (Ar), 138.55 (Ar), 138.17 (Ar), 135.68 (Ar), 135.59 (Ar), 131.97 (Ar), 131.94 (Ar), 131.03 (Ar), 130.52 (Ar), 130.45 (Ar), 130.08 (Ar), 128.98 (Ar), 128.94 (Ar), 128.57 (Ar), 128.42 (Ar), 126.90 (Ar), 126.26 (Ar), 123.17 (Ar), 113.96 (Ar), 111.06 (Ar), 104.05 (Ar), 44.24 (CH_2_), 18.27 (CH_3_), 12.42 (CH_3_); FT‐IR Vmax (cm^–1^): 317 (*N–H stretch*,), 3122 (*N–H or Ar–H stretch*), 2967 (*C–H stretch, N‐ethyl*), 1618 (*C*═*N or Ar–C*═*C stretch*), 1591 (*N–C*═*S or C*═*N*,), 1540 (*N–H bend or Ar–C*═*C*), 1482 (*Ar–C–C stretch*), 1410 (*CH*_*2*_ *bend, N‐ethyl*), 1356 (*S*═*O sym. stretch, sulfonate*), 1265 (*S*═*O asym. stretch, sulfonate*), 1185 (*C–N or S–O stretch*), 1063 (*C–N stretch or Ar–C–H bend*), 956 (*N–H wag or C*═*S*), 852 (*C–H out‐of‐plane, aromatic*), 754 (*Ar–C–H out‐of‐plane bend*); HPLC: CH_3_CN:H_2_O = 80: 20; *R*_t_: 3.903 min, purity: 98.26%; Elemental Analysis: CHN Calcd: C, 63.71; H, 5.53; N, 10.25; found: C, 63.92; H, 5.68; N, 10.44; ESI‐HRMS (*m/z*): chemical formula: C_29_H_30_N_4_O_3_S_2_, calcd [M + H]^+^: 547.18376, found [M + H]^+^: 547.18202 *(1.74 ppm deviation)*, calcd [M + Na]^+^: 569.16570, found [M + Na]^+^: 569.16380 *(1.90 ppm deviation)*.

(*E*)‐2‐{[2‐(Benzylcarbamothioyl)hydrazono]methyl}‐5‐(diethylamino)phenyl naphthalene‐2‐sulfonate (**5s**): Yield: 93%; M.P: 186°C–188°C, Color: yellow; ^1^H‐NMR: *δ* (400 MHz, DMSO‐*d*_6_) 11.46 (1 H, s, NH–N═C), 8.78 (1 H, t, *J* = 6.3 Hz, S═C–NH–R), 8.65 (1 H, d, *J* = 1.9 Hz, Ar), 8.27–8.17 (3 H, m, Ar, HC═N), 8.11 (1 H, d, *J* = 8.2 Hz, Ar), 7.97 (1 H, dd, *J* = 8.7, 2.0 Hz, Ar), 7.89 (1 H, d, *J* = 9.0 Hz, Ar), 7.84–7.75 (1 H, m, Ar), 7.75–7.64 (1 H, m, Ar), 7.32 (4 H, d, *J* = 5.6 Hz, Ar), 7.24 (1 H, td, *J* = 5.8, 2.5 Hz, Ar), 6.56 (1 H, dd, *J* = 9.1, 2.5 Hz, Ar), 5.94 (1 H, d, *J* = 2.5 Hz, Ar), 4.80 (2 H, d, *J* = 6.2 Hz, *N*‐CH_2_), 3.10 (4 H, q, *J* = 7.0 Hz, CH_2_), 0.80 (6 H, t, *J* = 7.0 Hz, CH_3_); ^13^C‐NMR (101 MHz, DMSO‐*d*_6_) *δ* 177.41 (C═S), 149.77 (C═N), 149.66 (Ar), 140.02 (Ar), 137.87 (Ar), 131.95 (Ar), 131.88 (Ar), 131.02 (Ar), 130.49 (Ar), 130.07 (Ar), 128.60 (Ar), 128.56 (Ar), 128.41 (Ar), 127.67 (Ar), 127.15 (Ar), 123.16 (Ar), 113.96 (Ar), 104.06 (Ar), 46.93 (*N*‐CH_2_), 44.24 (CH_2_), 12.42 (CH_3_); FT‐IR Vmax (cm^–1^): 3359 (*N–H stretch*), 3154 (*N–H or Ar–H stretch*), 2970 (*C–H stretch, N‐ethyl*), 1613 (*C*═*N or Ar–C*═*C stretch*), 1518 (*N–H bend or Ar–C*═*C*), 1451 (*CH₂ bend, N‐ethyl*), 1409 (*CH₂ bend or Ar–C–C stretch*), 1267 (*S*═*O asym. stretch, sulfonate*), 1217 (*C–N or S–O stretch*), 1084 (*C–N stretch or Ar–C–H bend*), 1067 (*C–N stretch or Ar–C–H bend*), 1019 (*C–N stretch or Ar–C–H bend*), 960 (*N–H wag or C*═*S*), 761 (*Ar–C–H out‐of‐plane bend*); HPLC: CH_3_CN: H_2_O = 80: 20; *t*_R_: 3.819 min, purity: 98.14%; Elemental Analysis: CHN Calcd: C, 63.71; H, 5.53; N, 10.25; found: C, 63.791; H, 5.64; N, 10.34; ESI‐HRMS (*m/z*): chemical formula: C_29_H_30_N_4_O_3_S_2_, calcd [M + H]^+^: 547.18376, found [M + H]^+^: 547.18182 *(1.94 ppm deviation)*, calcd [M + Na]^+^: 569.16570, found [M + Na]^+^: 569.16373 *(1.97 ppm deviation)*.

(*E*)‐5‐(Diethylamino)‐2‐{[2‐(phenethylcarbamothioyl)hydrazono]methyl}phenyl naphthalene‐2‐sulfonate (**5t**): Yield: 95%; M.P: 195°C–197°C; Color: pale yellow; ^1^H‐NMR: *δ* (400 MHz, DMSO‐*d*_6_) 11.40 (1 H, s, NH‐N═C), 8.65 (1 H, d, *J* = 2.0 Hz, S═C–NH–R), 8.31–8.16 (4 H, m, Ar, HC═N), 8.11 (1 H, d, *J* = 8.2 Hz, Ar), 7.98 (1 H, dd, *J* = 8.8, 2.0 Hz, Ar), 7.84–7.75 (2 H, m, Ar), 7.72 (1 H, ddd, *J* = 8.2, 6.8, 1.3 Hz, Ar), 7.37–7.17 (5 H, m, Ar), 6.60 (1 H, dd, *J* = 9.1, 2.5 Hz, Ar), 5.94 (1 H, d, *J* = 2.5 Hz, Ar), 3.73 (2 H, ddd, *J* = 9.4, 7.6, 5.8 Hz, *N*‐CH_2_), 3.11 (4 H, q, *J* = 7.0 Hz, CH_2_), 2.89 (2 H, dd, *J* = 9.1, 6.4 Hz, CH_2_), 0.81 (6 H, t, *J* = 7.0 Hz, CH_3_); ^13^C‐NMR (100 MHz, DMSO‐*d*_6_) *δ* 176.83 (C═S), 149.75 (C═N), 149.66 (Ar), 139.75 (Ar), 137.68 (Ar), 135.66 (Ar), 131.95 (Ar), 131.93 (Ar), 131.00 (Ar), 130.50 (Ar), 130.06 (Ar), 129.06 (Ar), 128.93 (Ar), 128.56 (Ar), 128.42 (Ar), 126.65 (Ar), 123.14 (Ar), 113.99 (Ar), 111.05 (Ar), 104.10 (Ar), 45.37 (*N*‐CH_2_), 44.24 (CH_2_), 35.42 (CH_3_), 12.43 (CH_3_); FT‐IR Vmax (cm^–1^): 3351 (*N–H stretch*,), 3124 (*N–H or Ar–H stretch*), 3055 (*Ar–H stretch*), 2964 (*C–H stretch, N‐ethyl*), 2925 (*C–H stretch, aliphatic*), 1612 (*C*═*N or Ar–C*═*C stretch*), 1542 (*N–H bend or Ar–C*═*C*), 1513 (*Ar–C–C stretch*), 1451 (*CH₂ bend, N‐ethyl*), 1401 (*CH₂ bend or Ar–C–C stretch*), 1378 (*S*═*O sym. stretch, sulfonate*), 1266 (*S*═*O asym. stretch, sulfonate*), 1123 (*C–N or S–O stretch*), 949 (*N–H wag or C*═*S*), 857 (*C–H out‐of‐plane, aromatic*), 754 (*Ar–C–H out‐of‐plane bend*); HPLC: CH_3_CN:H_2_O = 80:20; *R*_t_: 4.649 min, purity: 99.32%; Elemental Analysis: CHN Calcd: C, 64.26; H, 5.75; N, 9.99; found: C, 64.48; H, 5.93; N, 10.19; ESI‐HRMS (*m/z*): chemical formula: C_30_H_32_N_4_O_3_S_2_, calcd [M + H]^+^: 561.19941, found [M + H]^+^: 561.19773 *(1.68 ppm deviation)*, calcd [M + Na]^+^: 583.18135, found [M + Na]^+^: 583.17930 *(2.05 ppm deviation)*.

(*E*)‐2‐({2‐[(2,6‐Dichlorophenyl)carbamothioyl]hydrazono}methyl)‐5‐(diethylamino)phenyl naphthalene‐2‐sulfonate (**5u**): Yield: 94%; M.P: 226°C–228°C; Color: pale yellow; ^1^H‐NMR: *δ* (400 MHz, DMSO‐*d*_6_) 11.89 (1 H, s, NH–N═C), 9.86 (1 H, s, S═C–NH–R), 8.68 (1 H, d, *J* = 1.9 Hz, Ar), 8.32 (1 H, s, HC═N), 8.25 (2 H, t, *J* = 8.4 Hz, Ar), 8.17–8.04 (2 H, m, Ar), 8.01 (1 H, dd, *J* = 8.7, 2.0 Hz, Ar), 7.80 (1 H, ddd, *J* = 8.2, 6.9, 1.3 Hz, Ar), 7.72 (1 H, ddd, *J* = 8.1, 6.9, 1.3 Hz, Ar), 7.54 (2 H, d, *J* = 8.1 Hz, Ar), 7.36 (1 H, dd, *J* = 8.6, 7.6 Hz, Ar), 6.58 (1 H, dd, *J* = 9.2, 2.5 Hz, Ar), 5.92 (1 H, d, *J* = 2.5 Hz, Ar), 3.10 (4 H, q, *J* = 7.0 Hz, CH_2_), 0.79 (6 H, t, *J* = 7.0 Hz, CH_3_); ^13^C‐NMR (100 MHz, DMSO‐*d*_6_) *δ* 176.95 (C═S), 149.97 (C═N), 149.83 (Ar), 138.55 (Ar), 135.74 (Ar), 135.72 (Ar), 135.69 (Ar), 131.98 (Ar), 131.91 (Ar), 131.07 (Ar), 130.53 (Ar), 130.08 (Ar), 129.68 (Ar), 128.91 (Ar), 128.71 (Ar), 128.61 (Ar), 128.44 (Ar), 123.18 (Ar), 113.86 (Ar), 111.03 (Ar), 103.98 (Ar), 44.24 (CH_2_), 12.41 (CH_3_); FT‐IR Vmax (cm^−1^): 3318 (*N–H stretch*), 3130 (*N–H or Ar–H stretch*), 2970 (*C–H stretch, N‐ethyl*), 1612 (*C*═*N or Ar–C*═*C stretch*), 1588 (*N–C*═*S or C*═*N*), 1543 (*N–H bend or Ar–C*═*C*), 1521 (*N–H bend or Ar–C*═*C*), 1435 (*CH₂ bend, N‐ethyl*), 1373 (*S*═*O sym. stretch, sulfonate*), 1350 (*S*═*O sym. stretch, sulfonate*), 1222 (*S*═*O asym. stretch, sulfonate*), 1166 (*C–N or S–O stretch*), 1068 (*C–N stretch or Ar–C–H bend*), 956 (*N–H wag or C*═*S*), 842 (*C–H out‐of‐plane, aromatic*), 777 (*C–Cl stretch, dichlorobenzene*), 757 (*C–Cl stretch, dichlorobenzene*), 546 (*C–Cl bending or skeletal vibration*); HPLC: CH_3_CN:H_2_O = 80:20; *R*_t_: 3.388 min, purity: 98.81%; Elemental Analysis: CHN anal. Calcd: C, 55.90; H, 4.36; N, 9.31; found: C, 56.34; H, 4.47; N, 9.52; ESI‐HRMS (*m/z*): chemical formula: C_28_H_26_^35^Cl_2_N_4_O_3_S_2_, calcd [M + H]^+^: 601.09016, found [M + H]^+^: 601.08809 *(2.07 ppm deviation)*, calcd [M + Na]^+^: 623.07211, found [M + Na]^+^: 623.06722 *(4.89 ppm deviation)*, chemical formula: C_28_H_26_^37^Cl_2_N_4_O_3_S_2_, calcd [M + H]^+^: 603.08420, found [M + H]^+^: 603.08546 *(1.26 ppm deviation)*, calcd [M + Na]^+^: 625.06620, found [M + Na]^+^: 625.06722 *(1.02 ppm deviation)*.

### 4.2 Inhibition Assays

#### 4.2.1 Activity and Inhibition Test for AChE and BChE

Both acetylcholine iodide (AChI) and butyrylcholine iodide (BChI) were used as substrates for the enzymatic hydrolysis reactions, with 5,5′‐dithiobis(2‐nitrobenzoic acid) (DTNB) serving as a common reagent for detecting the activities of AChE and BChE. The assay was conducted: 1 mL of 1.0 M Tris/HCl buffer (pH 8.0) was mixed with 10 μL of the sample solution in deionized water. To this mixture, 50 μL of AChE or BChE enzyme solution was added, and the reaction was incubated at room temperature for 10 min. After incubation, 50 μL of DTNB (0.5 mM) was introduced, followed by the addition of 50 μL of either AChI or BChI (10 mM), initiating the enzymatic reaction. The hydrolysis of the substrates was monitored spectrophotometrically by measuring the absorbance at 412 nm, corresponding to the formation of the yellow‐colored 5‐thio‐2‐nitrobenzoate anion. This color change is due to the reaction of DTNB with thiocholine released during the hydrolysis of AChI or BChI. For every inhibitor that was utilized, IC_50_ and *K*_i_ values were determined. In this study, C7512‐ BChE from equine serum and C3389‐ AChE from *Electrophorus electricus* (electric eel) were purchased from Sigma Aldrich. For inhibitors exhibiting an inhibitory effect, an activity (%)‐[I] graph was created, and from this graph, inhibitor doses (IC_50_ values) resulting in 50% inhibition were computed [59, 60, 61, 62].

#### 4.2.2 Study of MAO‐A inhibition

The in vitro fluorometric technique was used to assess each newly generated substance's inhibitory activity against hMAO‐A and hMAO‐B. In a horseradish peroxidase coupling reaction, the oxired reagent (10‐acetyl‐3,7‐dihydroxyphenoxazine) detects H_2_O_2_, a result of the oxidative deamination of tyramine, which is utilized as an MAO substrate. The test was carried out in two steps. First, each compound's concentrations of 10^–3^ and 10^–4^ M were investigated. In the second step, substances that demonstrated inhibitory activity at a concentration of 10^−5^–10^−9 ^M more than 50% were subjected to additional analysis. Table 1 displays the IC50 values for the recently produced compounds against the hMAO‐A and hMAO‐B isoforms. These parameters of this study were made as in previous studies [63]. We purchased these two enzymes from Sigma Aldrich (M7316‐Monoamine Oxidase A human and M7441‐ Monoamine Oxidase A human) [64].

### 4.3 Computational Methods

#### 4.3.1 Molecular Docking Studies

Protein and ligand preparation, grid generation, and docking were performed using AutoDockTools 1.5.6 [51, 52, 53]. Ligand stability was achieved by minimizing energy at the MM2 level with Chem3D Pro [51, 52, 53]. The protein structures were obtained from the Protein Data Bank (https://www.rcsb.org/), specifically *h*MAO‐A (PDB ID: 2Z5X), MAO‐B (PDB ID: 2V5Z), AChE (PDB‐ID: 1B41), and BChE (PDB ID: 4BDS), based on previous studies [31, 55, 56, 62]. Interactions between ligands and receptors were analyzed in both 2D and 3D using BIOVIA Discovery Studio Visualizer software [53]. Detailed docking methodology is provided in the supplementary information (SI) file.

#### 4.3.2 2D‐MLR‐Based QSAR Study

In our analysis, we utilized “QSARINS‐2.2.4” for QSAR analysis based on GA‐MLR (Genetic Algorithm‐Multiple Linear Regression). The chemical structures in “SMILES” format were converted to 3D using “Openbabel 3.1,” and descriptors were computed with “PyDescriptor.” The process eliminated highly intercorrelated descriptors, resulting in over 1600+ for subsequent QSAR modeling. The methodology employed for the QSAR analysis was based on our previous publication [65].

#### 4.3.3 Molecular Dynamics studies

For MDS analysis, we simulated complexes “1B41_**5 u**,” “4BDS_**5a**,” “2v5z‐5u,” and “2Z5X_**5u**” using the “Desmond” module. An explicit solvent model with TIP3P water and the OPLS‐2005 force field was used in a 10 Å × 10 Å × 10 Å periodic box. Na^+^ ions and NaCl were added for charge balance and physiological conditions. The system was simulated for 100 ns. The details for setting up the complex were followed from earlier literature [66].

## Conflicts of Interest

The authors declare no conflicts of interest.

## Supporting information

InChI Codes.

Supporting Information R1.

## Data Availability

The data that support the findings of this study are available in the supporting material of this article.
