# Supplementary material for: Design, Synthesis, In Vitro, and In Silico Studies of 5‐(Diethylamino)‐2‐Formylphenyl Naphthalene‐2‐Sulfonate Based Thiosemicarbazones as Potent Anti‐Alzheimer Agents
Source: Arch Pharm (Weinheim). 2025 Jul 20;358(7):e70050. doi: 10.1002/ardp.70050 (PMC12277872; doi:10.1002/ardp.70050)
Supplement: Supplementary file 1 — InChI Codes. [file ARDP-358-e70050-s002.docx]

|  |  | **IC_50_ (nM)** | | | | | | | | **Ki (nM)** | |
| --- | --- | --- | --- | --- | --- | --- | --- | --- | --- | --- | --- |
| **Comp.** | **InChI** | **AChE** | **r^2^** | **BChE** | **r^2^** | **MAO-A** | **r^2^** | **MAO-B** | **r^2^** | **AChE** | **BChE** |
| **5a** | **1S/C28H26Cl2N4O3S2/c1-3-34(4-2)22-14-12-21(18-31-33-28(38)32-25-11-7-10-24(29)27(25)30)26(17-22)37-39(35,36)23-15-13-19-8-5-6-9-20(19)16-23/h5-18H,3-4H2,1-2H3,(H2,32,33,38)/b31-18+** | 14.90±1.54 | 0.913 | 124.72±9.13 | 0.982 | 104.17±4.58 | 0.960 | 391.03±9.04 | 0.989 | 11.37±3.06 | 101.32±22.43 |
| **5b** | **1S/C28H27BrN4O3S2/c1-3-33(4-2)25-15-9-22(19-30-32-28(37)31-24-13-11-23(29)12-14-24)27(18-25)36-38(34,35)26-16-10-20-7-5-6-8-21(20)17-26/h5-19H,3-4H2,1-2H3,(H2,31,32,37)/b30-19+** | 33.81±3.41 | 0.906 | 202.05±5.66 | 0.937 | 132.05±7.01 | 0.931 | 409.76±7.47 | 0.928 | 26.55±3.34 | 190.57±15.65 |
| **5c** | **1S/C28H28N4O3S2/c1-3-32(4-2)25-16-14-23(20-29-31-28(36)30-24-12-6-5-7-13-24)27(19-25)35-37(33,34)26-17-15-21-10-8-9-11-22(21)18-26/h5-20H,3-4H2,1-2H3,(H2,30,31,36)/b29-20+** | 38.42±5.03 | 0.904 | 217.78±8.41 | 0.929 | 188.54±6.46 | 0.933 | 296.47±6.01 | 0.990 | 25.43±5.32 | 182.65±15.04 |
| **5d** | **1S/C29H30N4O4S2/c1-4-33(5-2)25-14-10-23(20-30-32-29(38)31-24-12-15-26(36-3)16-13-24)28(19-25)37-39(34,35)27-17-11-21-8-6-7-9-22(21)18-27/h6-20H,4-5H2,1-3H3,(H2,31,32,38)/b30-20+** | 39.37±2.87 | 0.968 | 252.54±10.46 | 0.923 | 170.66±10.42 | 0.935 | 406.18±8.24 | 0.942 | 32.23±5.93 | 234.84±15.04 |
| **5e** | **1S/C28H27ClN4O3S2/c1-3-33(4-2)25-14-12-22(19-30-32-28(37)31-24-11-7-10-23(29)17-24)27(18-25)36-38(34,35)26-15-13-20-8-5-6-9-21(20)16-26/h5-19H,3-4H2,1-2H3,(H2,31,32,37)/b30-19+** | 31.04±4.16 | 0.921 | 208.43±7.53 | 0.951 | 117.13±7.33 | 0.923 | >1000 | 0.935 | 25.36±3.32 | 195.34±11.53 |
| **5f** | **1S/C26H32N4O3S2/c1-5-30(6-2)23-13-11-22(18-28-29-26(34)27-17-19(3)4)25(16-23)33-35(31,32)24-14-12-20-9-7-8-10-21(20)15-24/h7-16,18-19H,5-6,17H2,1-4H3,(H2,27,29,34)/b28-18+** | 49.53±5.21 | 0.947 | 253.53±6.31 | 0.964 | 133.50±8.04 | 0.901 | 388.10±5.37 | 0.957 | 38.82±6.18 | 239.89±18.65 |
| **5g** | **1S/C26H32N4O3S3/c1-4-30(5-2)23-13-11-22(19-28-29-26(34)27-15-8-16-35-3)25(18-23)33-36(31,32)24-14-12-20-9-6-7-10-21(20)17-24/h6-7,9-14,17-19H,4-5,8,15-16H2,1-3H3,(H2,27,29,34)/b28-19+** | 80.43±7.62 | 0.978 | 287.26±8.57 | 0.970 | 187.66±5.36 | 0.964 | 398.57±7.93 | 0.902 | 72.43±10.08 | 258.50±16.14 |
| **5h** | **1S/C29H27F3N4O3S2/c1-3-36(4-2)23-15-13-22(19-33-35-28(40)34-26-12-8-7-11-25(26)29(30,31)32)27(18-23)39-41(37,38)24-16-14-20-9-5-6-10-21(20)17-24/h5-19H,3-4H2,1-2H3,(H2,34,35,40)/b33-19+** | 16.76±1.35 | 0.949 | 145.03±8.96 | 0.913 | 100.38±4.57 | 0.930 | 249.01±9.10 | 0.944 | 14.59±3.36 | 136.01±9.17 |
| **5i** | **1S/C28H27ClN4O3S2/c1-3-33(4-2)25-15-9-22(19-30-32-28(37)31-24-13-11-23(29)12-14-24)27(18-25)36-38(34,35)26-16-10-20-7-5-6-8-21(20)17-26/h5-19H,3-4H2,1-2H3,(H2,31,32,37)/b30-19+** | 24.81±2.41 | 0.958 | 199.54±9.41 | 0.902 | 121.24±3.15 | 0.924 | 285.25±4.02 | 0.912 | 21.02±4.12 | 190.27±13.60 |
| **5j** | **1S/C30H32N4O3S2/c1-5-34(6-2)26-16-14-25(20-31-33-30(38)32-29-21(3)10-9-11-22(29)4)28(19-26)37-39(35,36)27-17-15-23-12-7-8-13-24(23)18-27/h7-20H,5-6H2,1-4H3,(H2,32,33,38)/b31-20+** | 40.62±2.77 | 0.979 | 265.38±7.90 | 0.970 | 136.93±6.21 | 0.981 | 395.37±8.35 | 0.966 | 36.52±5.23 | 228.42±12.35 |
| **5k** | **1S/C29H30N4O4S2/c1-4-33(5-2)25-15-13-23(20-30-32-29(38)31-24-11-8-12-26(18-24)36-3)28(19-25)37-39(34,35)27-16-14-21-9-6-7-10-22(21)17-27/h6-20H,4-5H2,1-3H3,(H2,31,32,38)/b30-20+** | 116.01±8.42 | 0.906 | 252.64±9.15 | 0.977 | 144.71±8.03 | 0.976 | >1000 | 0.952 | 95.75±9.54 | 205.34±10.16 |
| **5l** | **1S/C28H27FN4O3S2/c1-3-33(4-2)25-15-9-22(19-30-32-28(37)31-24-13-11-23(29)12-14-24)27(18-25)36-38(34,35)26-16-10-20-7-5-6-8-21(20)17-26/h5-19H,3-4H2,1-2H3,(H2,31,32,37)/b30-19+** | 29.54±2.68 | 0.918 | 200.34±6.88 | 0.985 | 112.33±2.78 | 0.907 | 276.97±6.33 | 0.938 | 27.41±3.45 | 182.09±9.05 |
| **5m** | **1S/C23H26N4O3S2/c1-4-27(5-2)20-12-10-19(16-25-26-23(31)24-3)22(15-20)30-32(28,29)21-13-11-17-8-6-7-9-18(17)14-21/h6-16H,4-5H2,1-3H3,(H2,24,26,31)/b25-16+** | 38.49±3.21 | 0.909 | 214.30±7.13 | 0.963 | 132.28±4.96 | 0.954 | 368.58±11.25 | 0.926 | 30.22±5.02 | 193.05±11.62 |
| **5n** | **1S/C22H24N4O3S2/c1-3-26(4-2)19-11-9-18(15-24-25-22(23)30)21(14-19)29-31(27,28)20-12-10-16-7-5-6-8-17(16)13-20/h5-15H,3-4H2,1-2H3,(H3,23,25,30)/b24-15+** | 47.70±5.67 | 0.970 | 308.43±9.98 | 0.932 | 137.19±6.55 | 0.976 | 395.72±8.09 | 0.930 | 42.44±4.10 | 270.16±13.54 |
| **5o** | **1S/C28H27N5O5S2/c1-3-32(4-2)24-14-12-22(19-29-31-28(39)30-23-10-7-11-25(17-23)33(34)35)27(18-24)38-40(36,37)26-15-13-20-8-5-6-9-21(20)16-26/h5-19H,3-4H2,1-2H3,(H2,30,31,39)/b29-19+** | 18.93±1.33 | 0.998 | 192.09±6.36 | 0.981 | 122.65±7.01 | 0.932 | 267.07±6.35 | 0.905 | 14.25±2.32 | 160.33±9.01 |
| **5p** | **1S/C28H34N4O3S2/c1-3-32(4-2)25-16-14-23(20-29-31-28(36)30-24-12-6-5-7-13-24)27(19-25)35-37(33,34)26-17-15-21-10-8-9-11-22(21)18-26/h8-11,14-20,24H,3-7,12-13H2,1-2H3,(H2,30,31,36)/b29-20+** | 54.46±4.57 | 0.915 | 254.24±8.08 | 0.965 | 145.32±3.57 | 0.985 | 326.68±9.13 | 0.955 | 47.09±6.35 | 226.45±12.57 |
| **5q** | **1S/C29H29ClN4O3S2/c1-3-34(4-2)26-15-11-24(20-32-33-29(38)31-19-21-9-13-25(30)14-10-21)28(18-26)37-39(35,36)27-16-12-22-7-5-6-8-23(22)17-27/h5-18,20H,3-4,19H2,1-2H3,(H2,31,33,38)/b32-20+** | 19.34±1.28 | 0.984 | 202.31±14.86 | 0.963 | 119.06±9.24 | 0.926 | 308.44±6.07 | 0.927 | 17.63±3.34 | 182.76±11.48 |
| **5r** | **1S/C29H30N4O3S2/c1-4-33(5-2)25-16-14-24(20-30-32-29(37)31-27-13-9-6-10-21(27)3)28(19-25)36-38(34,35)26-17-15-22-11-7-8-12-23(22)18-26/h6-20H,4-5H2,1-3H3,(H2,31,32,37)/b30-20+** | 27.39±2.42 | 0.912 | 293.55±7.14 | 0.912 | 142.55±6.78 | 0.944 | 495.13±8.36 | 0.995 | 21.17±1.05 | 249.09±15.30 |
| **5s** | **1S/C29H30N4O3S2/c1-3-33(4-2)26-16-14-25(21-31-32-29(37)30-20-22-10-6-5-7-11-22)28(19-26)36-38(34,35)27-17-15-23-12-8-9-13-24(23)18-27/h5-19,21H,3-4,20H2,1-2H3,(H2,30,32,37)/b31-21+** | 71.43±8.92 | 0.954 | 220.34±8.53 | 0.954 | 131.09±5.34 | 0.976 | 369.46±12.04 | 0.975 | 63.86±7.54 | 195.21±13.45 |
| **5t** | **1S/C30H32N4O3S2/c1-3-34(4-2)27-16-14-26(22-32-33-30(38)31-19-18-23-10-6-5-7-11-23)29(21-27)37-39(35,36)28-17-15-24-12-8-9-13-25(24)20-28/h5-17,20-22H,3-4,18-19H2,1-2H3,(H2,31,33,38)/b32-22+** | 56.21±4.58 | 0.902 | 271.12±6.90 | 0.981 | 138.74±6.02 | 0.932 | 289.02±9.11 | 0.945 | 48.30±7.32 | 226.65±16.08 |
| **5u** | **1S/C28H26Cl2N4O3S2/c1-3-34(4-2)22-14-12-21(18-31-33-28(38)32-27-24(29)10-7-11-25(27)30)26(17-22)37-39(35,36)23-15-13-19-8-5-6-9-20(19)16-23/h5-18H,3-4H2,1-2H3,(H2,32,33,38)/b31-18+** | 12.89±1.02 | 0.987 | 148.18±7.35 | 0.973 | 96.25±5.77 | 0.906 | 208.95±5.84 | 0.978 | 9.82±1.76 | 112.06±7.61 |
| **^*^** |  | 101.24±5.72 | 0.984 | 261.62±9.03 | 0.945 | - | - |  |  | 84.05±8.70 | 242.17±13.88 |
| **^**^** |  | - | - | - | - | 150.62±4.03 | 0.979 | 468.63±16.05 | 0.987 | - | - |
