# Supplementary material for: Design, Synthesis, In Vitro, and In Silico Studies of 5‐(Diethylamino)‐2‐Formylphenyl Naphthalene‐2‐Sulfonate Based Thiosemicarbazones as Potent Anti‐Alzheimer Agents
Source: Arch Pharm (Weinheim). 2025 Jul 20;358(7):e70050. doi: 10.1002/ardp.70050 (PMC12277872; doi:10.1002/ardp.70050)
Supplement: Supplementary file 2 — Supporting Information R1. [file ARDP-358-e70050-s001.pdf]

Design, synthesis, in vitro and in silico studies of 5-(diethylamino)-2-formylphenyl naphthalene-2-sulfonate based thiosemicarbazones as potent anti-Alzheimer agents

Urva Farooq[a, ≠], Muhammad Islam[b,c, ≠], Zahra Batool[a], Suraj N. Mali[d], Rahul D. Jawarkar[e], Shailesh S. Gurav[f], Rima D. Alharthy[g], Halil Şenol[h], Nastaran Sadeghian[i], Parham Taslimi[i], Zahid Shafiq[a]* and Silvia Schenone[j]*

[a]*Institute of Chemical Sciences, Bahauddin Zakariya University, 60800, Multan, Pakistan.*

[b]*Department of Chemistry, Muhammad Nawaz Sharif University of Engineering and Technology (MNSUET), 60000, Multan, Pakistan*

[c]*School of Pharmaceutical Science and Technology, Tianjin University, 92 Weijin Road, Tianjin 300072, China.*

[d]*School of Pharmacy, D.Y. Patil University (Deemed to be University), Sector 7, Nerul, Navi Mumbai 400706, India*

[e]*Department of Medicinal Chemistry, Dr. Rajendra Gode Institute of Pharmacy, University-Mardi Road, Amravati, India*

[f]*Department of Chemistry, VIVA College, Virar (W), Maharashtra-401303, India.*

[g]*Department of Chemistry, Science & Arts College, Rabigh Branch, King Abdulaziz University, Rabigh 21911, Saudi Arabia*

[h]*Bezmialem Vakif University, Faculty of Pharmacy, Department of Pharmaceutical Chemistry, 34093 Fatih, Istanbul, Türkiye*

[i]*Department of Biotechnology, Faculty of Science, Bartin University, 74110 Bartin, Türkiye*

[j]*Department of Pharmacy, University of Genoa, Viale Benedetto XV, 3, Genoa 16132, Italy*

***Corresponding Authors**:*

Prof. Dr. Zahid Shafiq: zahidshafiq@bzu.edu.pk

Prof. Dr. Silvia Schenone: silvia.schenone@unige.it

≠ = Contributed equally

TABLE OF CONTENTS

Molecular Docking Studies Further Analysis ... 6
Materials and methods ... 6
Figure S 1. Complex of **5e**-2V5Z co-crystalized with FAD molecule ... 7
QSAR Model ... 8
Figure S 2. ^{1}H-NMR Spectrum of Compound **5a** (DMSO-*d6*, 400 MHz ... 10
Figure S 3. ^{13}C-NMR Spectrum of Compound **5a** (DMSO-*d6*, 100 MHz) ... 10
Figure S 4. HPLC Purity Analysis of Compound **5a** ... 11
Figure S 5. ESI-HRMS Spectrum of Compound **5a** ... 12
Figure S 6. ESI-HRMS Spectrum of Compound **5a** (extended) ... 12
Figure S 7. FT-IR Spectrum of Compound **5a** ... 13
Figure S 8. ^{1}H-NMR Spectrum of Compound **5b** (DMSO-*d6*, 400 MHz) ... 14
Figure S 9. ^{13}C-NMR Spectrum of Compound **5b** (DMSO-*d6*, 100 MHz) ... 14
Figure S 10. HPLC Purity Analysis of Compound **5b** ... 15
Figure S 11. ESI-HRMS Spectrum of Compound **5b** ... 16
Figure S 12. ESI-HRMS Spectrum of Compound **5b** (extended) ... 16
Figure S 13. FT-IR Spectrum of Compound **5b** ... 17
Figure S 14. ^{1}H-NMR Spectrum of Compound **5c** (DMSO-*d6*, 400 MHz) ... 18
Figure S 15. ^{13}C-NMR Spectrum of Compound **5c** (DMSO-*d6*, 100 MHz) ... 18
Figure S 16. HPLC Purity Analysis of Compound **5c** ... 20
Figure S 17. ESI-HRMS Spectrum of Compound **5c** ... 20
Figure S 18. FT-IR Spectrum of Compound **5c** ... 20
Figure S 19. ^{1}H-NMR Spectrum of Compound **5d** (DMSO-*d6*, 400 MHz) ... 21
Figure S 20. ^{13}C-NMR Spectrum of Compound **5d** (DMSO-*d6*, 100 MHz) ... 21
Figure S 21. HPLC Purity Analysis of Compound **5d** ... 22
Figure S 22. ESI-HRMS Spectrum of Compound **5d** ... 23
Figure S 23. FT-IR Spectrum of Compound **5d** ... 23
Figure S 24. ^{1}H-NMR Spectrum of Compound **5e** (DMSO-*d6*, 400 MHz) ... 24
Figure S 25. ^{13}C-NMR Spectrum of Compound **5e** (DMSO-*d6*, 100 MHz) ... 24
Figure S 26. HPLC Purity Analysis of Compound **5e** ... 25
Figure S 27. ESI-HRMS Spectrum of Compound **5e** ... 26
Figure S 28. FT-IR Spectrum of Compound **5e** ... 26
Figure S 29. ^{1}H-NMR Spectrum of Compound **5f** (DMSO-*d6*, 400 MHz) ... 27
Figure S 30. ^{13}C-NMR Spectrum of Compound **5f** (DMSO-*d6*, 100 MHz) ... 27

Figure S 31. HPLC Purity Analysis of Compound **5f** 28
Figure S 32. ESI-HRMS Spectrum of Compound **5f** 29
Figure S 33. FT-IR Spectrum of Compound **5f** 29
Figure S 34. ^{1}H-NMR Spectrum of Compound **5g** (DMSO-*d6*, 400 MHz) 30
Figure S 35. ^{13}C-NMR Spectrum of Compound **5g** (DMSO-*d6*, 100 MHz) 30
Figure S 36. HPLC Purity Analysis of Compound **5g** 31
Figure S 37. ESI-HRMS Spectrum of Compound **5g** 32
Figure S 38. FT-IR Spectrum of Compound **5g** 32
Figure S 39. ^{1}H-NMR Spectrum of Compound **5h** (DMSO-*d6*, 400 MHz) 33
Figure S 40. ^{13}C-NMR Spectrum of Compound **5h** (DMSO-*d6*, 100 MHz) 33
Figure S 41. HPLC Purity Analysis of Compound **5h** 34
Figure S 42. ESI-HRMS Spectrum of Compound **5h** 35
Figure S 43. FT-IR Spectrum of Compound **5h** 35
Figure S 44. ^{1}H-NMR Spectrum of Compound **5i** (DMSO-*d6*, 400 MHz) 36
Figure S 45. ^{13}C-NMR Spectrum of Compound **5i** (DMSO-*d6*, 100 MHz) 36
Figure S 46. HPLC Purity Analysis of Compound **5i** 37
Figure S 47. ESI-HRMS Spectrum of Compound **5i** 38
Figure S 48. FT-IR Spectrum of Compound **5i** 38
Figure S 49. ^{1}H-NMR Spectrum of Compound **5j** (DMSO-*d6*, 400 MHz) 39
Figure S 50. ^{13}C-NMR Spectrum of Compound **5j** (DMSO-*d6*, 100 MHz) 39
Figure S 51. HPLC Purity Analysis of Compound **5j** 40
Figure S 52. ESI-HRMS Spectrum of Compound **5j** 41
Figure S 53. FT-IR Spectrum of Compound **5j** 41
Figure S 54. ^{1}H-NMR Spectrum of Compound **5k** (DMSO-*d6*, 400 MHz) 42
Figure S 55. ^{13}C-NMR Spectrum of Compound **5k** (DMSO-*d6*, 100 MHz) 42
Figure S 56. HPLC Purity Analysis of Compound **5k** 43
Figure S 57. ESI-HRMS Spectrum of Compound **5k** 44
Figure S 58. FT-IR Spectrum of Compound **5k** 44
Figure S 59. ^{1}H-NMR Spectrum of Compound **5l** (DMSO-*d6*, 400 MHz) 45
Figure S 60. ^{13}C-NMR Spectrum of Compound **5l** (DMSO-*d6*, 100 MHz) 45
Figure S 61. HPLC Purity Analysis of Compound **5l** 46
Figure S 62. ESI-HRMS Spectrum of Compound **5l** 47
Figure S 63. ESI-HRMS Spectrum of Compound **5l** (extended) 47
Figure S 64. FT-IR Spectrum of Compound **5l** 48
Figure S 65. ^{1}H-NMR Spectrum of Compound **5m** (DMSO-*d6*, 400 MHz) 49
Figure S 66. ^{13}C-NMR Spectrum of Compound **5m** (DMSO-*d6*, 100 MHz) 50

Figure S 67. HPLC Purity Analysis of Compound **5m** 50
Figure S 68. ESI-HRMS Spectrum of Compound **5m** 51
Figure S 69. FT-IR Spectrum of Compound **5m** 51
Figure S 70. ^{1}H-NMR Spectrum of Compound **5n** (DMSO-*d6*, 400 MHz) 52
Figure S 71. ^{13}C-NMR Spectrum of Compound **5n** (DMSO-*d6*, 100 MHz) 52
Figure S 72. HPLC Purity Analysis of Compound **5n** 53
Figure S 73. ESI-HRMS Spectrum of Compound **5n** 54
Figure S 74. FT-IR Spectrum of Compound **5n** 54
Figure S 75. ^{1}H-NMR Spectrum of Compound **5o** (DMSO-*d6*, 400 MHz) 55
Figure S 76. ^{13}C-NMR Spectrum of Compound **5o** (DMSO-*d6*, 100 MHz) 56
Figure S 77. HPLC Purity Analysis of Compound **5o** 56
Figure S 78. ESI-HRMS Spectrum of Compound **5o** 57
Figure S 79. ESI-HRMS Spectrum of Compound **5o** (extended) 57
Figure S 80. FT-IR Spectrum of Compound **5o** 58
Figure S 81. ^{1}H-NMR Spectrum of Compound **5p** (DMSO-*d6*, 400 MHz) 59
Figure S 82. ^{13}C-NMR Spectrum of Compound **5p** (DMSO-*d6*, 100 MHz) 59
Figure S 83. HPLC Purity Analysis of Compound **5p** 60
Figure S 84. ESI-HRMS Spectrum of Compound **5p** 61
Figure S 85. FT-IR Spectrum of Compound **5p** 61
Figure S 86. ^{1}H-NMR Spectrum of Compound **5q** (DMSO-*d6*, 400 MHz) 62
Figure S 87. ^{13}C-NMR Spectrum of Compound **5q** (DMSO-*d6*, 100 MHz) 62
Figure S 88. HPLC Purity Analysis of Compound **5q** 63
Figure S 89. ESI-HRMS Spectrum of Compound **5q** 64
Figure S 90. ESI-HRMS Spectrum of Compound **5q** (extended) 64
Figure S 91. ESI-HRMS Spectrum of Compound **5q** (negative) 65
Figure S 92. ESI-HRMS Spectrum of Compound **5q** (negative-extended) 65
Figure S 93. FT-IR Spectrum of Compound **5q** 66
Figure S 94. ^{1}H-NMR Spectrum of Compound **5r** (DMSO-*d6*, 400 MHz) 67
Figure S 95. ^{13}C-NMR Spectrum of Compound **5r** (DMSO-*d6*, 100 MHz) 67
Figure S 96. HPLC Purity Analysis of Compound **5r** 68
Figure S 97. ESI-HRMS Spectrum of Compound **5r** 69
Figure S 98. FT-IR Spectrum of Compound **5r** 69
Figure S 99. ^{1}H-NMR Spectrum of Compound **5s** (DMSO-*d6*, 400 MHz) 70
Figure S 100. ^{13}C-NMR Spectrum of Compound **5s** (DMSO-*d6*, 100 MHz) 71
Figure S 101. HPLC Purity Analysis of Compound **5s** 71
Figure S 102. ESI-HRMS Spectrum of Compound **5s** 72

Figure S 103. ESI-HRMS Spectrum of Compound **5s** (extended)...................................... 72
Figure S 104. FT-IR Spectrum of Compound **5s** 73
Figure S 105. ^{1}H-NMR Spectrum of Compound **5t** (DMSO-*d6*, 400 MHz) 74
Figure S 106. ^{13}C-NMR Spectrum of Compound **5t** (DMSO-*d6*, 100 MHz) 74
Figure S 107. HPLC Purity Analysis of Compound **5t** 75
Figure S 108. ESI-HRMS Spectrum of Compound **5t** 76
Figure S 109. FT-IR Spectrum of Compound **5t** 76
Figure S 110. ^{1}H-NMR Spectrum of Compound **5u** (DMSO-*d6*, 400 MHz) 77
Figure S 111. ^{13}C-NMR Spectrum of Compound **5u** (DMSO-*d6*, 100 MHz) 77
Figure S 112. HPLC Purity Analysis of Compound **5u** 78
Figure S 113. ESI-HRMS Spectrum of Compound **5u** 79
Figure S 114. FT-IR Spectrum of Compound **5u** 79

Molecular Docking Studies Further Analysis

Materials and methods

The structure of compounds 5a-5v was built using ChemDraw Ultra 12.0 software package. The stable configuration of ligands was obtained by minimizing energy via the MM2 level, utilizing the Chem3D Pro 12.0. A known protein database bank (https://www.rcsb.org/) was used to retrieve 3D-crystal structures of proteins with PDB IDs: 1B41, 4BDS, 2V5Z and 2Z5X. Three-dimensional coordinates of the enzymes were retrieved in the PDB format. The protein and ligand preparation, grid generation, and docking were executed using a molecular modelling tool, AutoDockTools 1.5.6 software. Target proteins were pre-processed to remove all water molecules, ions, and co-crystallized ligands as they may interfere with docking. Further optimization was achieved by adding all hydrogen atoms, and assigning partial charges- Kollman charges and saved the prepared protein structure in PDBQT format.

The ligand preparation was achieved by adding the Gasteiger charges followed by merging all non-polar hydrogen atoms and then saving the ligand in PDBQT format, which is required by AutoDock Vina. The ligand was set up for docking with the help of AutoDockTools (ADT; Version 1.5.6) to define the torsional degrees of freedom to be considered during the docking process and all acyclic dihedral angles in the ligand were allowed to rotate freely. Using AutoDockTools, the grid box was defined that specifies the docking parameters was defined. The center and dimensions of the grid box were set to cover the binding site properly. The grid box parameters, including center coordinates and box size, were saved in the AutoDock Vina configuration file. Exhaustiveness (a parameter controlling the thoroughness of the search) was 8 as default.

The docking was executed by means of the AutoDock Vina program using a configuration file. After docking, the pose with the least binding energy was selected as the best-docked ligand with the corresponding receptor using the PyMOL software and; the ligand-protein complex was saved in PDB format. Further, 2D and 3D ligand-receptor interactions were analyzed using Discovery Studio visualizer software.

The protein structure 4BDS is human butyrylcholinesterase in complex with tacrine. Thus, for the docking against 4BDS, the grid center (x, y, z) coordinates of co-crystalized ligand-Tacrine were used. For 4BDS, the grid box was built with size (x,y,z) of 60 × 60 × 60 points and it was positioned with co-crystalized ligand coordinates (x,y,z): 132.994, 116.013, and 41.214. Correspondingly, For protein 2Z5X, a grid box of size (x,y,z) 40 × 40 × 40 was set along with coordinates (x,y,z): 37.986, 29.585, and -17.813 of co-crystalized ligand. For receptor 2V5Z the grid center (x, y, z) coordinates of co-crystalized inhibitor safinamide were

used, center (x, y, z) = 51.886, 156.452, 28.559 and size (x,y,z) 40 × 40 × 40.For protein 1B41 (PDB ID), the grid box was positioned in the middle of the protein having coordinates (x,y,z) 116.546, 110.33, and -134.181. The grid box constituted a large region to include the entire protein target, following the blind docking method in the absence of co-crystalized ligand [2-4].

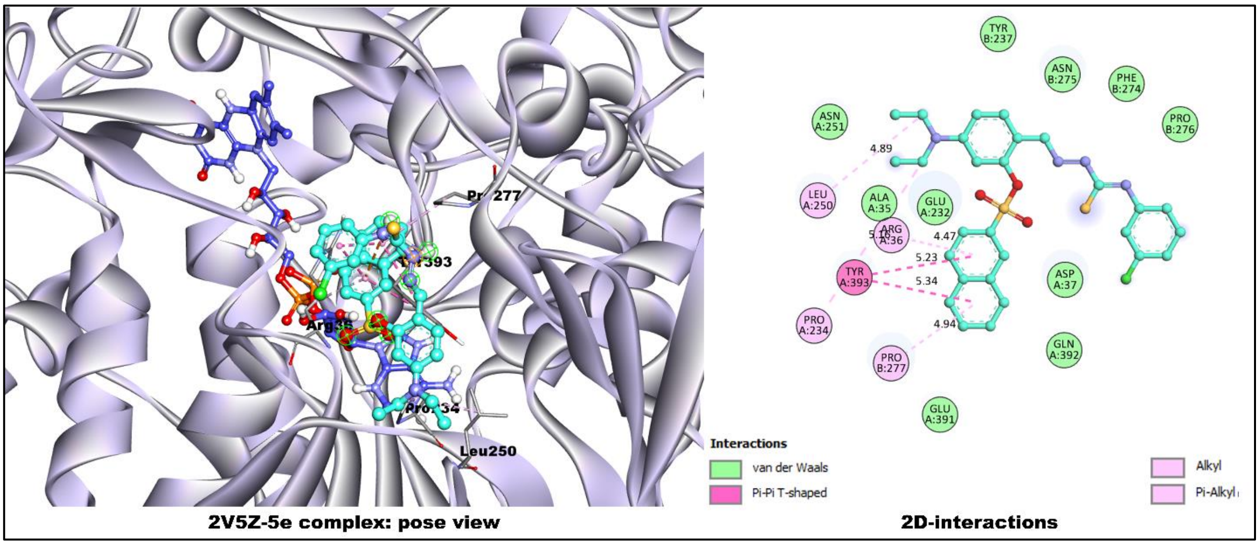

Figure S 1. Complex of **5e**-2V5Z co-crystalized with FAD molecule (blue-colored) and their 2D-binding interactions.

References

[1]. O. Trott A. J. Olson, Software News and update autodock vina: improving the speed and accuracy of docking with a new scoring function, efficient optimization, and multithreading, J Comput Chem. 31 (2009) 455–461. https://doi.org/10.1002/jcc.21334.

[2]. Lemke, C., et al., Chromenones as multineurotargeting inhibitors of human enzymes. ACS Omega 2019, 4, 22161−22168. DOI: 10.1021/acsomega.9b03409

[3]. Saquib Jalil, Rabia Basri, Mubashir Aziz, Zahid Shafiq, Syeda Abida Ejaz, Abdul Hameed, Jamshed Iqbal,Pristine 2-chloroquinoline-based-thiosemicarbazones as multitarget agents against alzheimer's disease: In vitro and in silico studies of monoamine oxidase (MAO) and cholinesterase (ChE) inhibitors, Journal of Molecular Structure, 1306, 2024, 137841,https://doi.org/10.1016/j.molstruc.2024.137841.

[4]. Qazi, S. U.; Naz, A.; Hameed, A.; Osra, F. A.; Jalil, S.; Iqbal, J.; Shah, S. A. A.; Mirza, A. Z. Semicarbazones, thiosemicarbazone, thiazole and oxazole analogues as

monoamine oxidase inhibitors: Synthesis, characterization, biological evaluation, molecular docking, and kinetic studies. Bioorganic chemistry 2021, 115, 105209.

QSAR Model

pIC_{50}=5.414 + 0.051 * com_Chyd_8A + 0.473 * fOS7B + -0.035 * C_sp3C_9B

com_Chyd_8A: occurrence of hydrophobic carbon atoms within 8 angstrom unit from the center of mass of the molecule.

fOS7B: frequency of occurrence of sulphur atom exactly at 7 bonds from the oxygen atoms.

C_sp3C_9B: occurrence of sp3 hybridized carbon atoms within 9 bonds from the carbon atoms.

Qsar parameters

R2_tr	0.8032	RMSE_ex	0.3257
Adj-R2	0.7578	PRESS_ex	0.5304
F(3-13)	17.6891	Q2F1	0.8131
RSS_tr	0.0925	Q2F2	0.8415
MSE_tr	0.0054	Q2F3	0.8364
RMSE_tr	0.0738	MAE_ex	0.2318
MAE_tr	0.058	K	0.9812
s	0.0843	K_prime	1.0169
AIC	-30.3916	R2ext	0.3604
BIC	-26.2255	CCC_ex	-0.3737
CCC_tr	0.8909	r2m_ExPy	-0.6492
Q2_cv	0.78	r2m_EyPx	-0.0196
RMSE_cv	0.3257	R2o	-0.7511
MSE_cv	0.1061	R2o_dash	-7.4853
PRESS_cv	0.5304	Clos_dash	21.7668
MAE_cv	0.2453	Clos	3.0837
R2_Yscr	0.1978	r2m_avg	-0.3344
		r2m_delta	0.6296
MSE_ex	0.1061		

Important molecular descriptors give us important information about how structural features affect biological activity in the QSAR model for MAO-B inhibitors. We measure hydrophobic carbon atoms (com_Chyd_8A) within an 8-angstrom radius from the molecule's center of mass. Because of their hydrophobic nature, the enzyme's active region contains residues like Ile-199 and Leu-171. These atoms are necessary to stop MAO-B from working. When these residues and inhibitors interact hydrophobically, the binding affinity goes up. Non-polar parts of the inhibitor stay in the binding pocket. A positive coefficient in the QSAR model means that the inhibitory activity (PIC50) goes up when the hydrophobic carbon density at the center of the molecule goes up. This is what drugs like rasagiline and selegiline do to make their effects more selective and potent.

One important indicator is sulfur-oxygen connectivity (fOS7B), which measures the number of sulfur atoms that are exactly seven bonds away from oxygen atoms. Sulfur-containing groups, like thiols and thioethers, help biological activity through electron transfer and redox interactions. Oxygen atoms, on the other hand, help hydrogen bonds form and make the substance more soluble in water. This particular connection arrangement facilitates ligand binding via both polar and nonpolar interactions. A positive coefficient ($\beta > 0$) shows how important this feature is for improving MAO-B binding efficiency. For example, sulfur-containing scaffolds like thiochromones can selectively block MAO-B.

Finally, the term sp3-hybridized carbon atoms (C_sp3C_9B) quantify the number of such carbons within nine bonds of any carbon atom. Excessive carbons, prevalent in aliphatic chains and saturated hydrocarbons, often diminish molecular planarity. Lack of planarity could make it harder for molecules to π-stack, which is needed to bind to aromatic residues in MAO-B like Tyr-398 and Tyr-435. On the other hand, molecules that are too flexible might not fit correctly into the enzyme's binding pocket. If the coefficient is less than zero, it means that reducing the number of sp3-hybridized carbons increases the inhibitory effect by making molecules flatter and more compact. Safinamide exemplifies a balance between stiff aromatic groups and little aliphatic branching; hence, it optimizes its effectiveness as a selective MAO-B inhibitor. All of these traits make the complicated connection between molecular structure and inhibitory function stand out. This makes it easier to come up with effective MAO-B inhibitors.

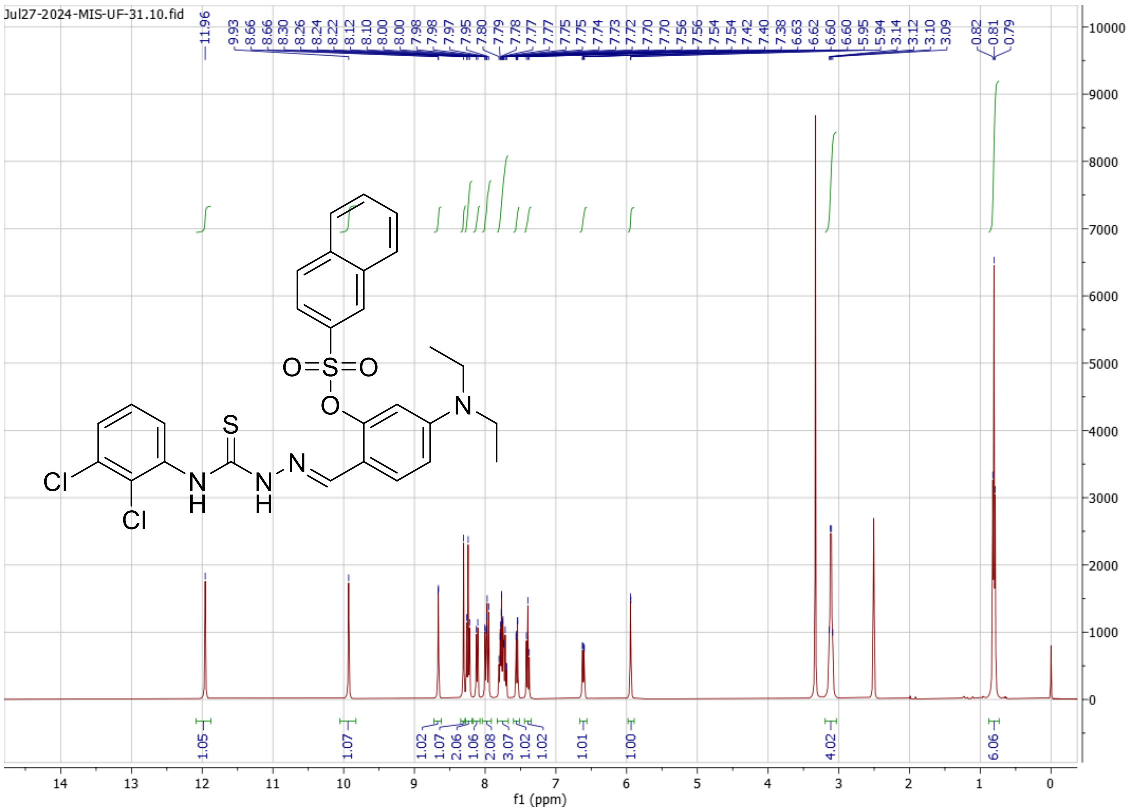

Figure S 2. ^{1}H-NMR Spectrum of Compound **5a** (DMSO-*d6*, 400 MHz

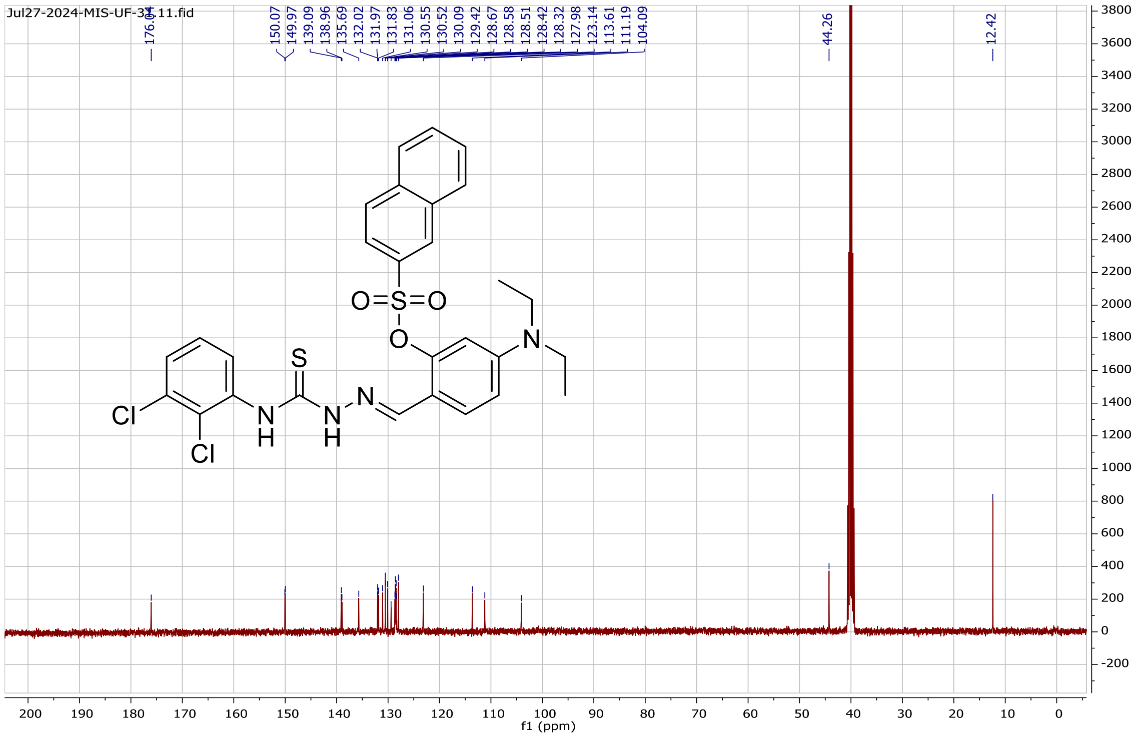

Figure S 3. ^{13}C-NMR Spectrum of Compound **5a** (DMSO-*d6*, 100 MHz)

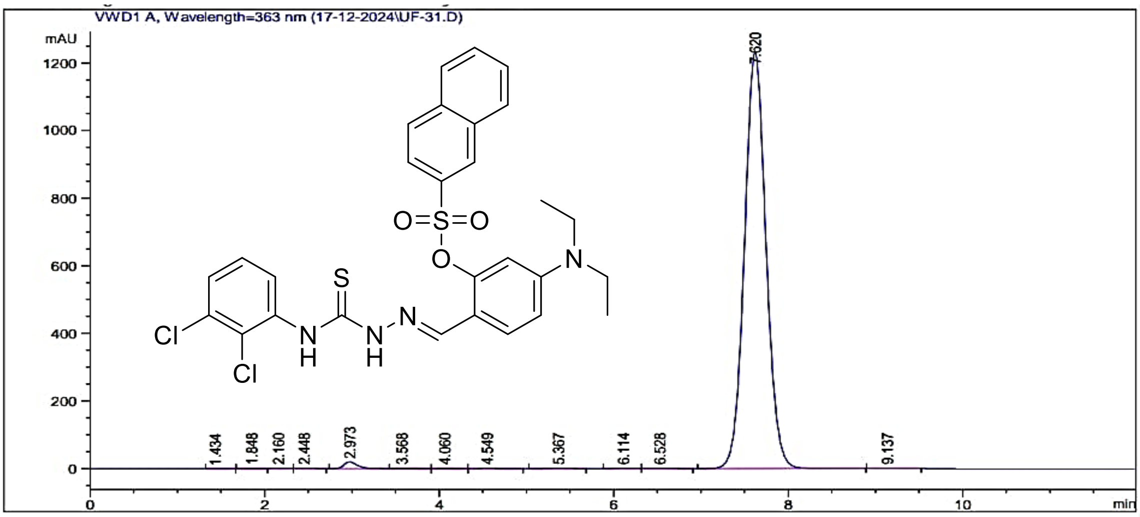

==

Area Percent Report

==

Sorted By : Signal
Multiplier : 1.0000
Dilution : 1.0000
Use Multiplier & Dilution Factor with ISTDs

Signal 1: VWD1 A, Wavelength=363 nm

Peak #	RetTime [min]	Type	Width [min]	Area [mAU*s]	Height [mAU]	Area %
1	1.434	BB	0.1358	1.45805	1.47357e-1	6.813e-3
2	1.848	BV E	0.1532	1.65824	1.40984e-1	7.749e-3
3	2.160	VV R	0.1810	8.27829	7.26445e-1	0.0387
4	2.448	VB	0.1445	9.07875	9.54357e-1	0.0424
5	2.973	BV R	0.1582	203.99185	19.53420	0.9532
6	3.568	VV E	0.1886	5.28300	3.90007e-1	0.0247
7	4.060	VB E	0.1771	3.61506	3.06103e-1	0.0169
8	4.549	BB	0.1848	4.71880	3.63877e-1	0.0221
9	5.367	BB	0.2111	13.58404	9.63324e-1	0.0635
10	6.114	BV	0.1945	6.49481	4.79369e-1	0.0304

HPLC 12/17/2024 4:07:28 PM SYSTEM Page 1 of 2

Data File D:\HPLC-DATA\Data\17-12-2024\UF-31.D
Sample Name: UF-31

Peak #	RetTime [min]	Type	Width [min]	Area [mAU*s]	Height [mAU]	Area %
11	6.528	VB	0.2337	14.22781	9.38505e-1	0.0665
12	7.620	BB	0.2658	2.11223e4	1230.59741	98.7042
13	9.137	BB	0.2338	4.91372	2.68647e-1	0.0230

Totals : 2.13997e4 1255.81059

Figure S 4. HPLC Purity Analysis of Compound **5a**

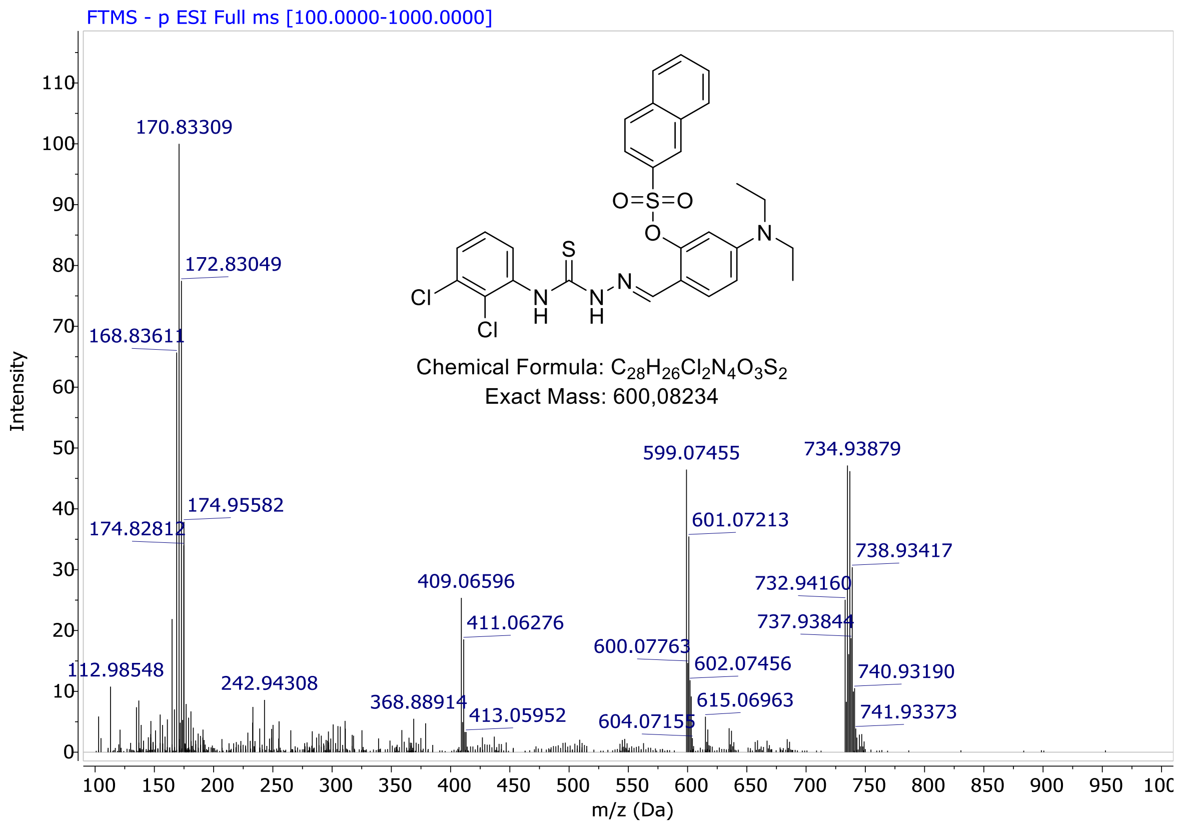

Figure S 5. ESI-HRMS Spectrum of Compound **5a**

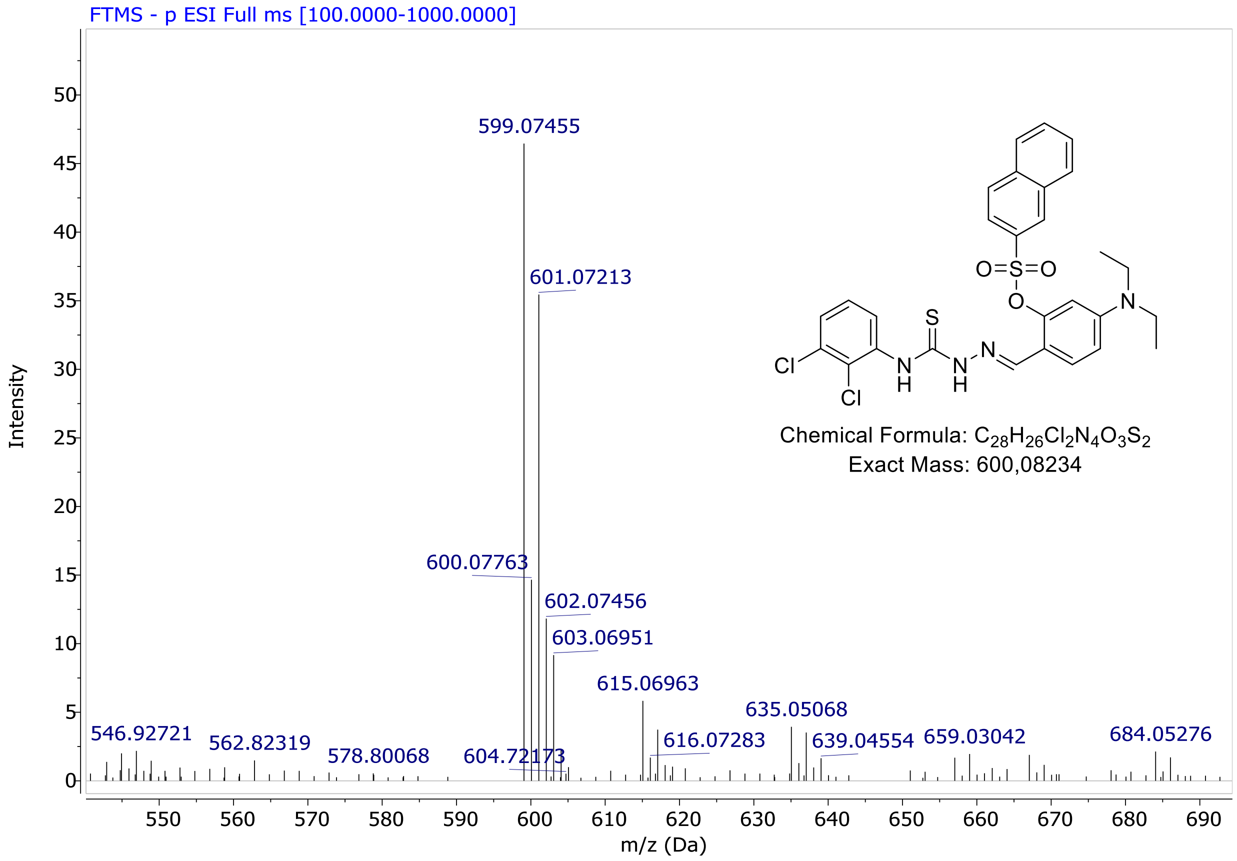

Figure S 6. ESI-HRMS Spectrum of Compound **5a** (extended)

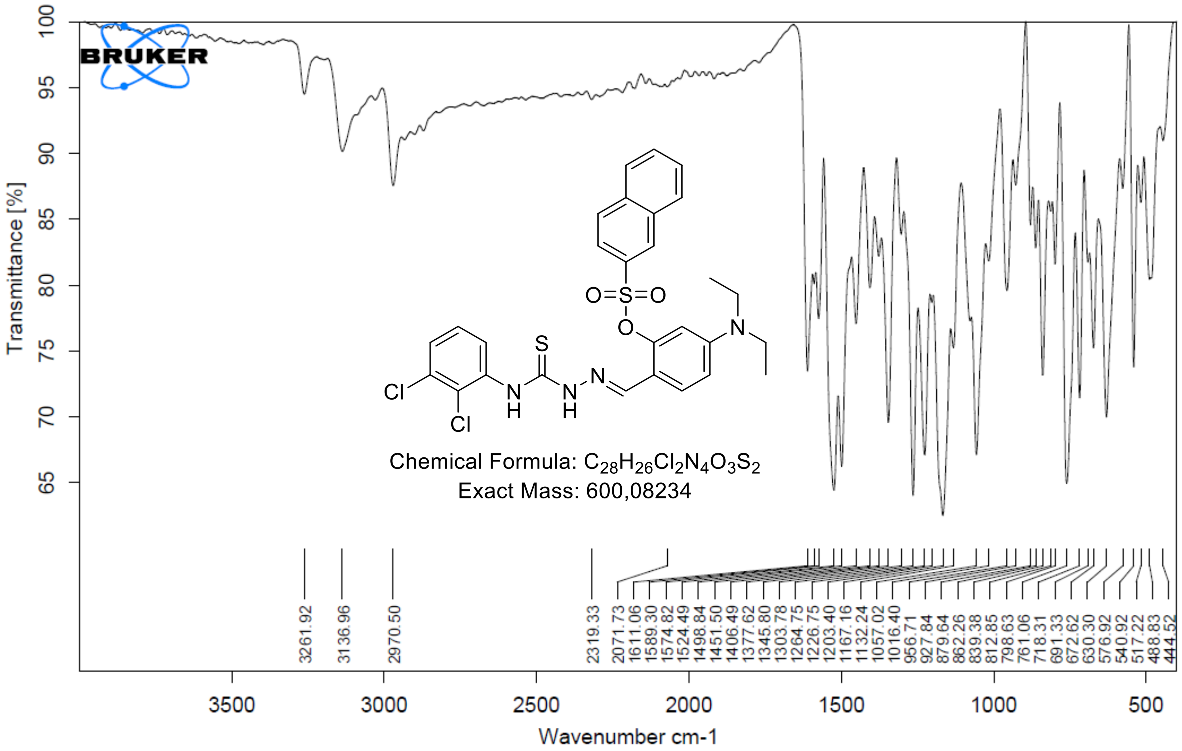

Figure S 7. FT-IR Spectrum of Compound **5a**

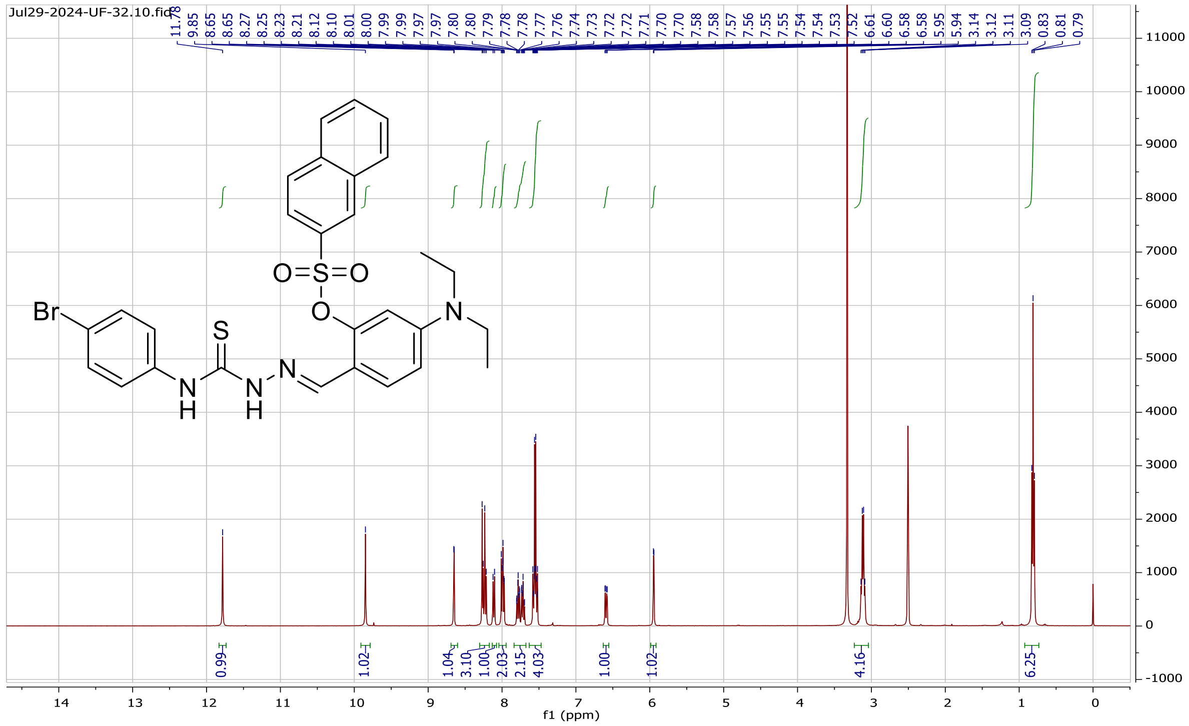

Figure S 8. ^{1}H-NMR Spectrum of Compound **5b** (DMSO-*d6*, 400 MHz)

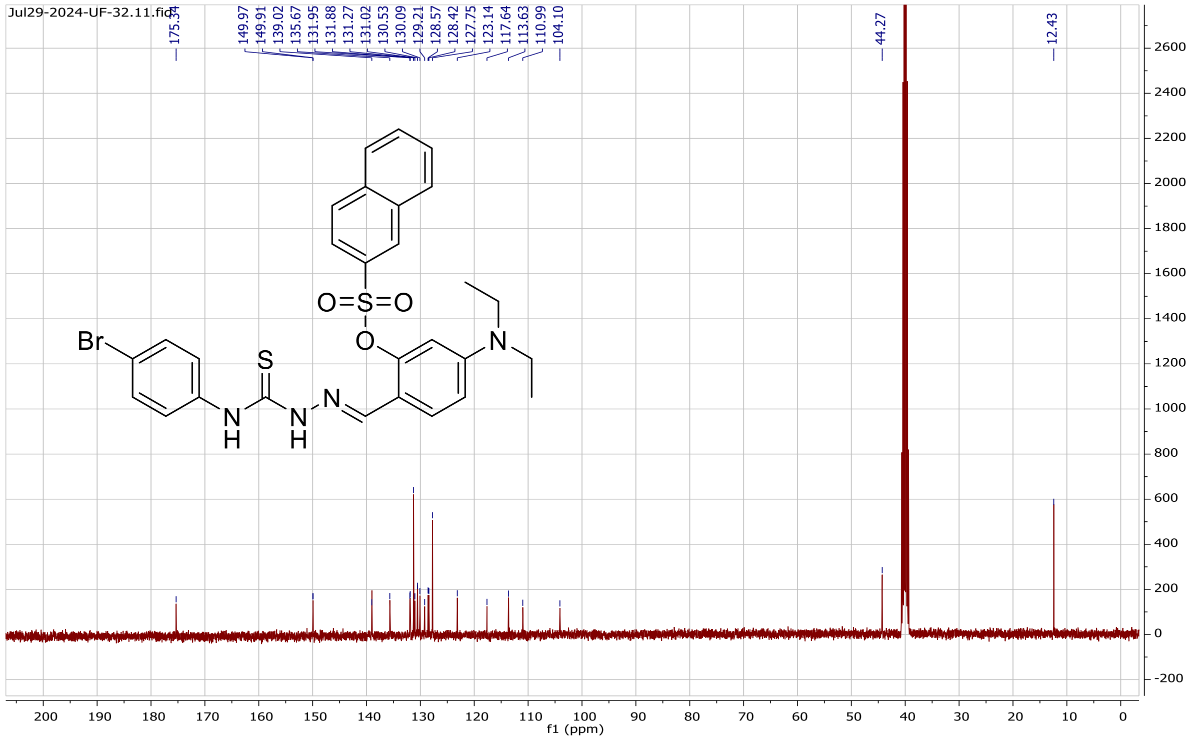

Figure S 9. ^{13}C-NMR Spectrum of Compound **5b** (DMSO-*d6*, 100 MHz)

Last changed : 5/29/2024 5:19:35 PM by SYSTEM

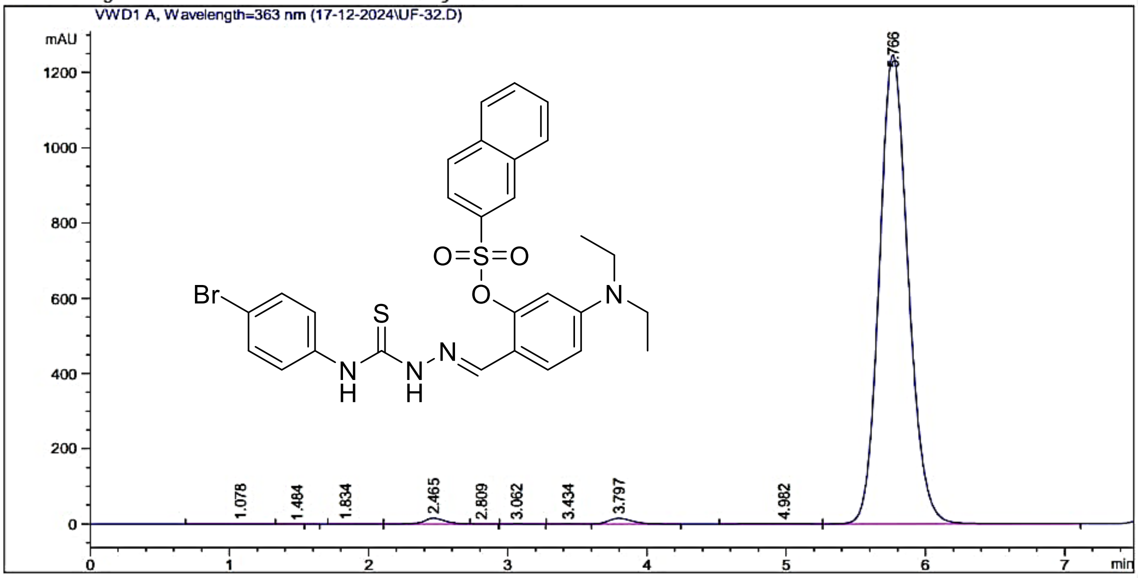

===

Area Percent Report

===

Sorted By : Signal
Multiplier : 1.0000
Dilution : 1.0000
Use Multiplier & Dilution Factor with ISTDs

Signal 1: VWD1 A, Wavelength=363 nm

Peak #	RetTime [min]	Type	Width [min]	Area [mAU*s]	Height [mAU]	Area %
1	1.078	BV R	0.2455	3.79857	1.89532e-1	0.0207
2	1.484	VV E	0.0956	2.34355e-1	3.31408e-2	1.278e-3
3	1.834	BB	0.1496	1.69731	1.54952e-1	9.257e-3
4	2.465	BV R	0.1537	157.82867	15.31502	0.8608
5	2.809	VB E	0.0877	8.23985e-1	1.28906e-1	4.494e-3
6	3.062	BB	0.1281	1.70550	1.98157e-1	9.301e-3
7	3.434	BV E	0.1497	2.13836	2.17367e-1	0.0117
8	3.797	VB R	0.1784	171.73717	14.87329	0.9366
9	4.982	BV	0.2662	23.40304	1.28592	0.1276
10	5.766	VB	0.2220	1.79727e4	1247.16541	98.0183

HPLC 12/17/2024 2:29:45 PM SYSTEM Page 1 of 2

Data File D:\HPLC-DATA\Data\17-12-2024\UF-32.D
Sample Name: UF-32

Peak #	RetTime [min]	Type	Width [min]	Area [mAU*s]	Height [mAU]	Area %
Totals :				1.83361e4	1279.56169	

Figure S 10. HPLC Purity Analysis of Compound **5b**

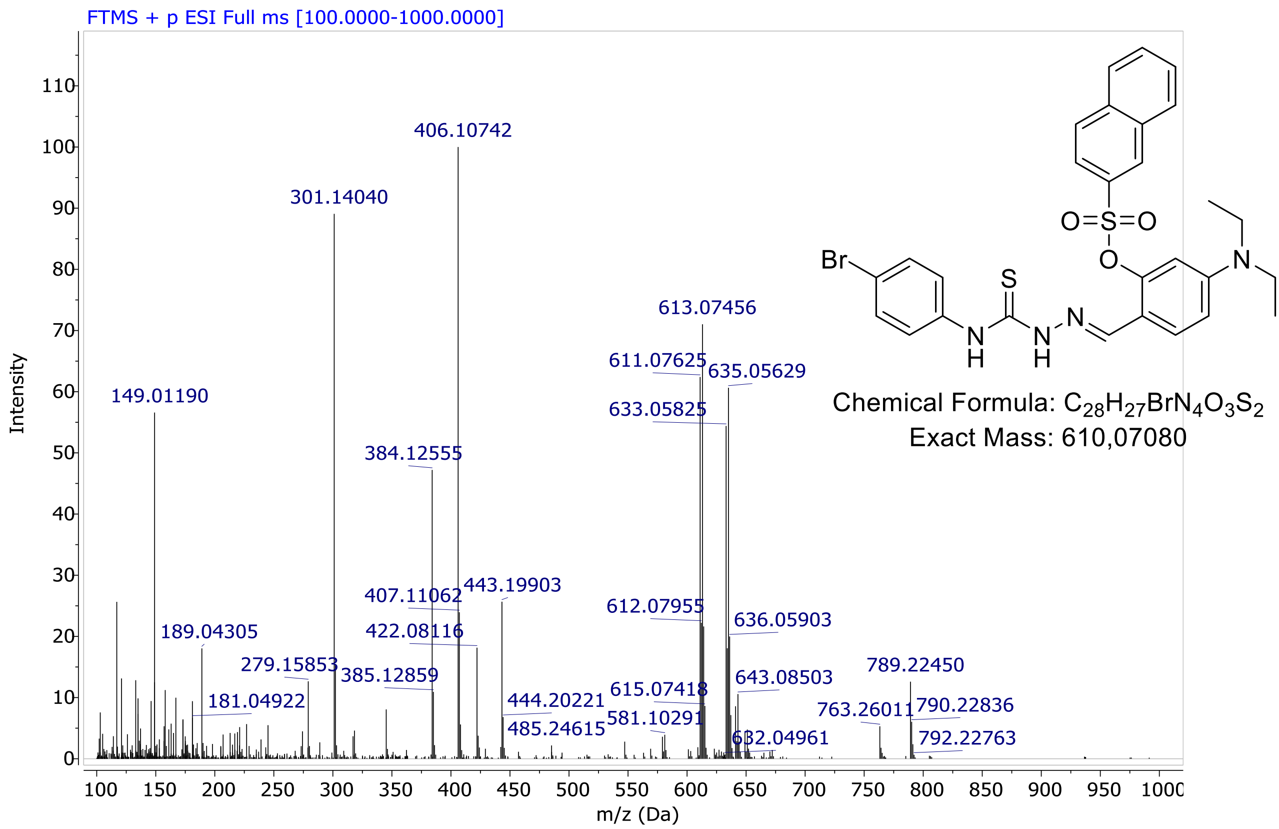

Figure S 11. ESI-HRMS Spectrum of Compound **5b**

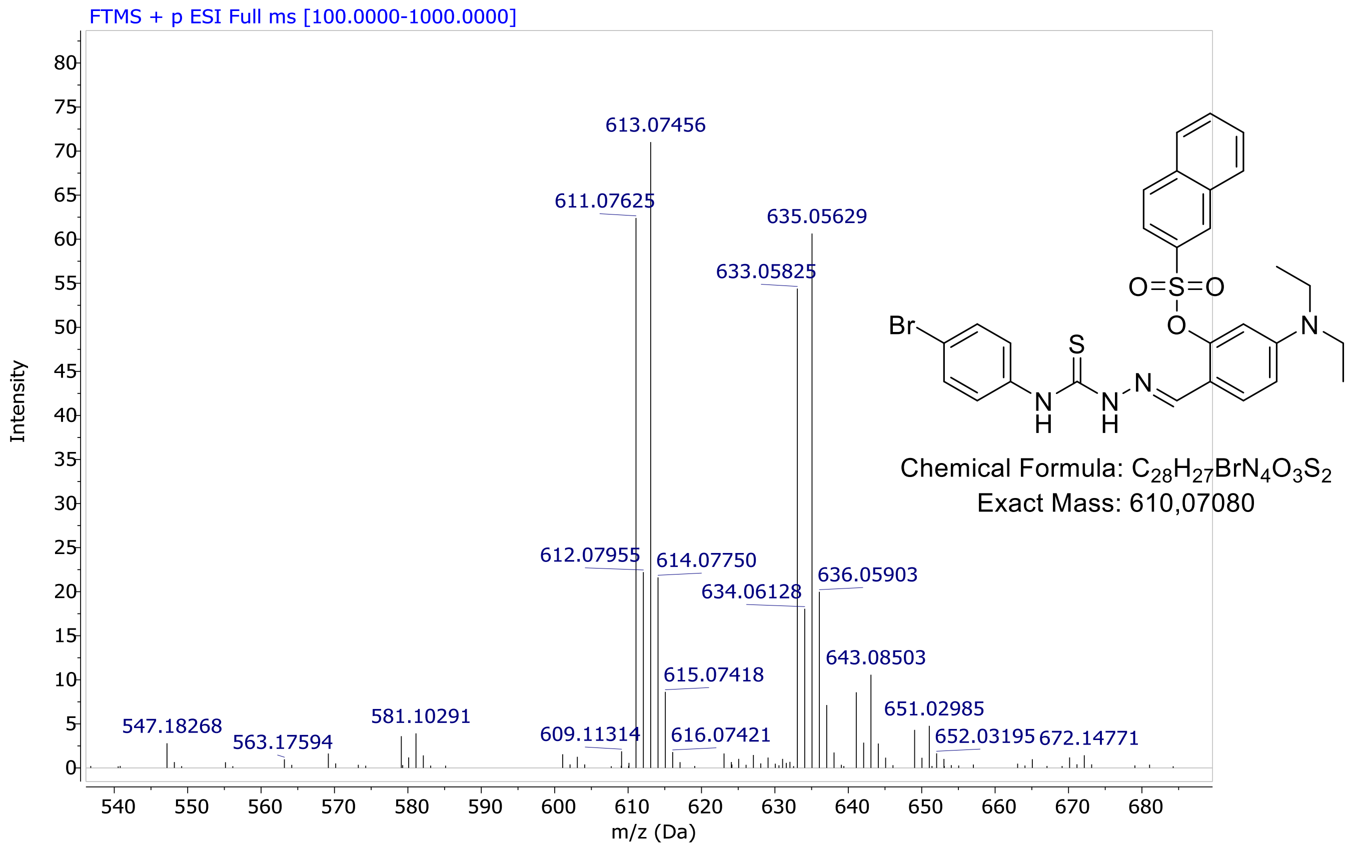

Figure S 12. ESI-HRMS Spectrum of Compound **5b** (extended)

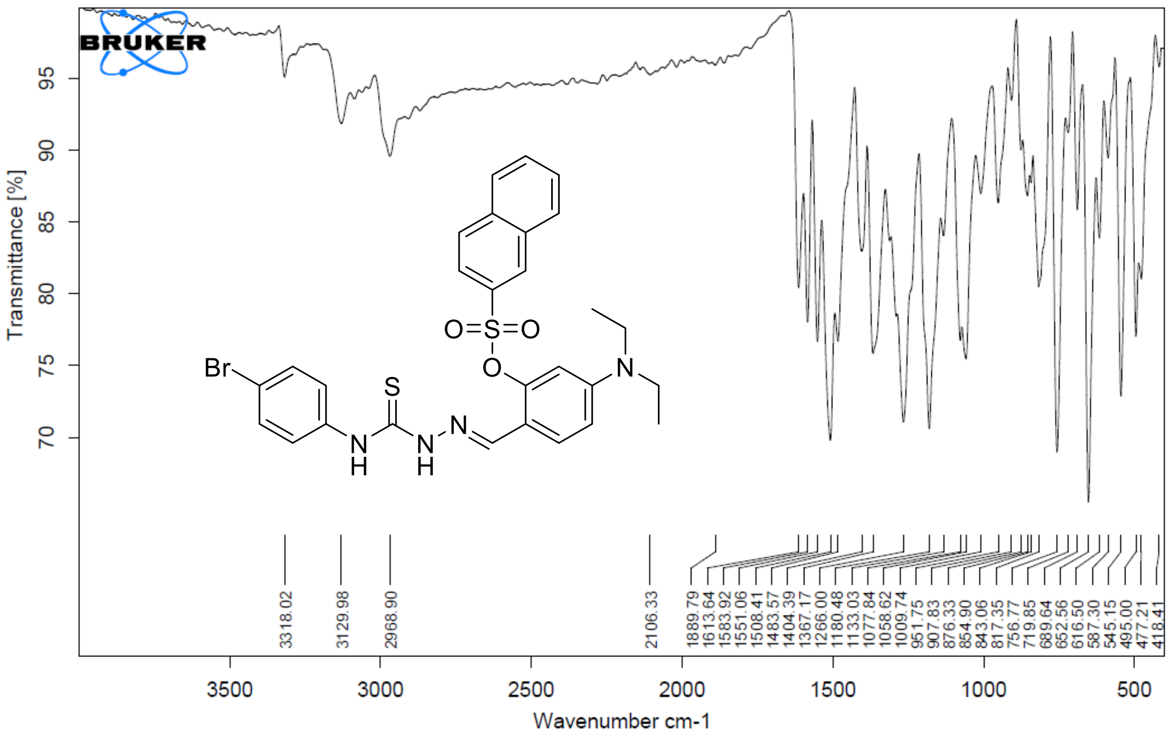

Figure S 13. FT-IR Spectrum of Compound **5b**

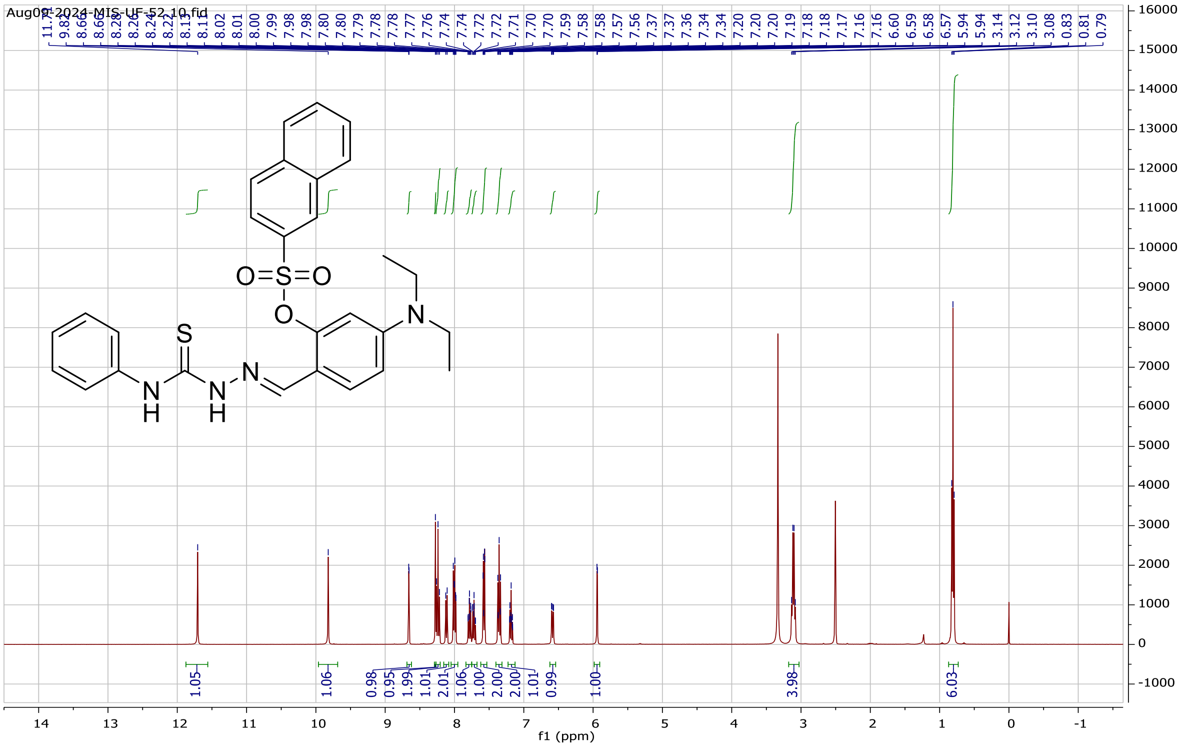

Figure S 14. ^{1}H-NMR Spectrum of Compound **5c** (DMSO-*d6*, 400 MHz)

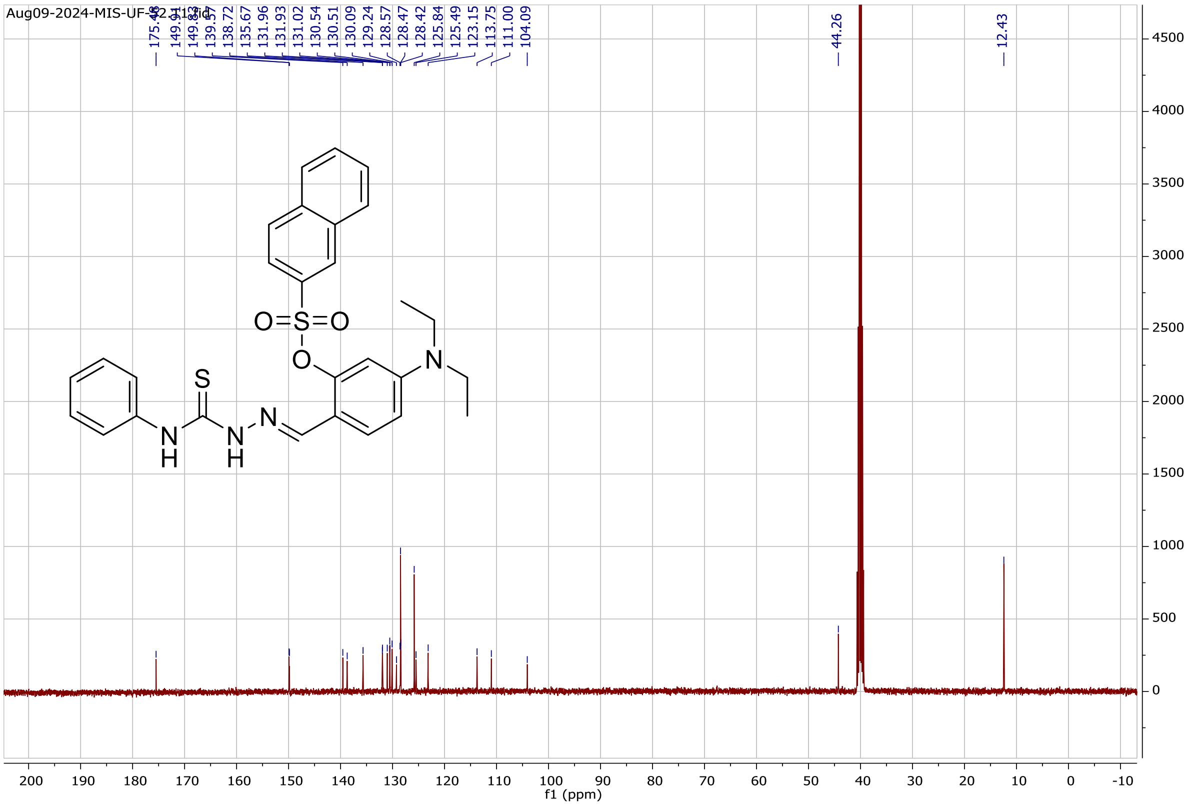

Figure S 15. ^{13}C-NMR Spectrum of Compound **5c** (DMSO-*d6*, 100 MHz)

```
==================================================================
Acq. Operator   : SYSTEM
Sample Operator : SYSTEM
Acq. Instrument : HPLC                          Location :    -
Injection Date  : 12/21/2024 1:55:33 PM              Inj :    1
                                              Inj Volume : No inj
Method          : D:\HPLC-DATA\Method\BZ-11.M
Last changed    : 5/29/2024 5:19:35 PM by SYSTEM
```

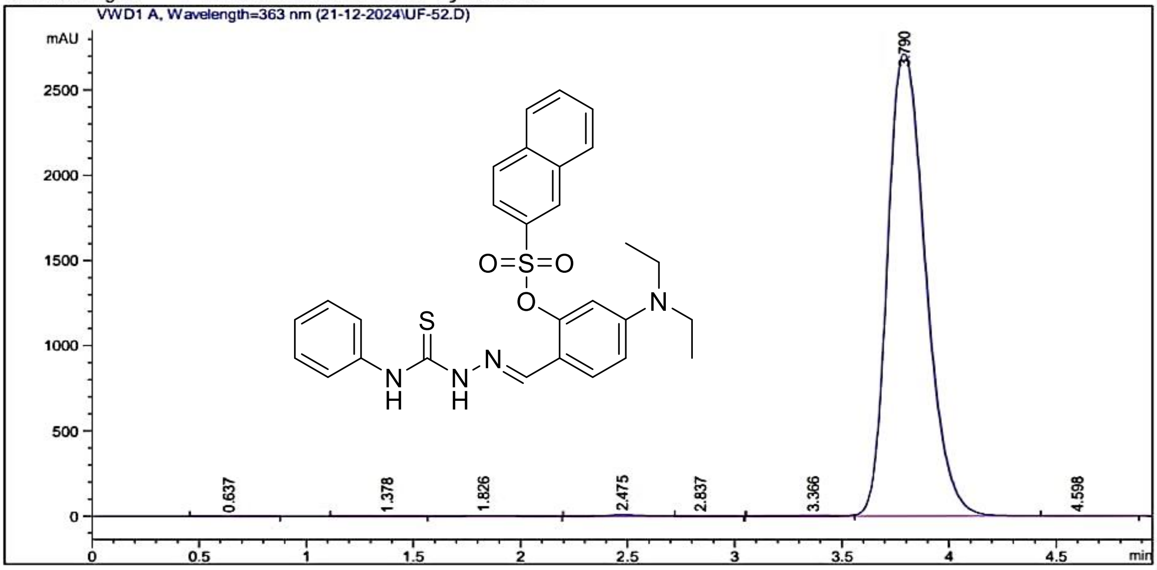


```
==================================================================
                    Area Percent Report
==================================================================

Sorted By           :      Signal
Multiplier          :      1.0000
Dilution            :      1.0000
Use Multiplier & Dilution Factor with ISTDs


Signal 1: VWD1 A, Wavelength=363 nm
```

Peak #	RetTime [min]	Type	Width [min]	Area [mAU*s]	Height [mAU]	Area %
1	0.637	BB	0.1682	2.47061	1.93153e-1	7.324e-3
2	1.378	BV E	0.2017	2.84027	1.74399e-1	8.420e-3
3	1.826	VB R	0.1618	28.39550	2.67245	0.0842
4	2.475	BV R	0.1613	76.41418	6.97383	0.2265
5	2.837	VB E	0.1402	3.08681	3.19880e-1	9.151e-3
6	3.366	BV E	0.1763	55.11078	4.64399	0.1634
7	3.790	VV R	0.1909	3.35574e4	2713.43994	99.4805
8	4.598	VBAE	0.1987	6.91442	5.01389e-1	0.0205
Totals :				3.37326e4	2728.91904	

Figure S 16. HPLC Purity Analysis of Compound **5c**

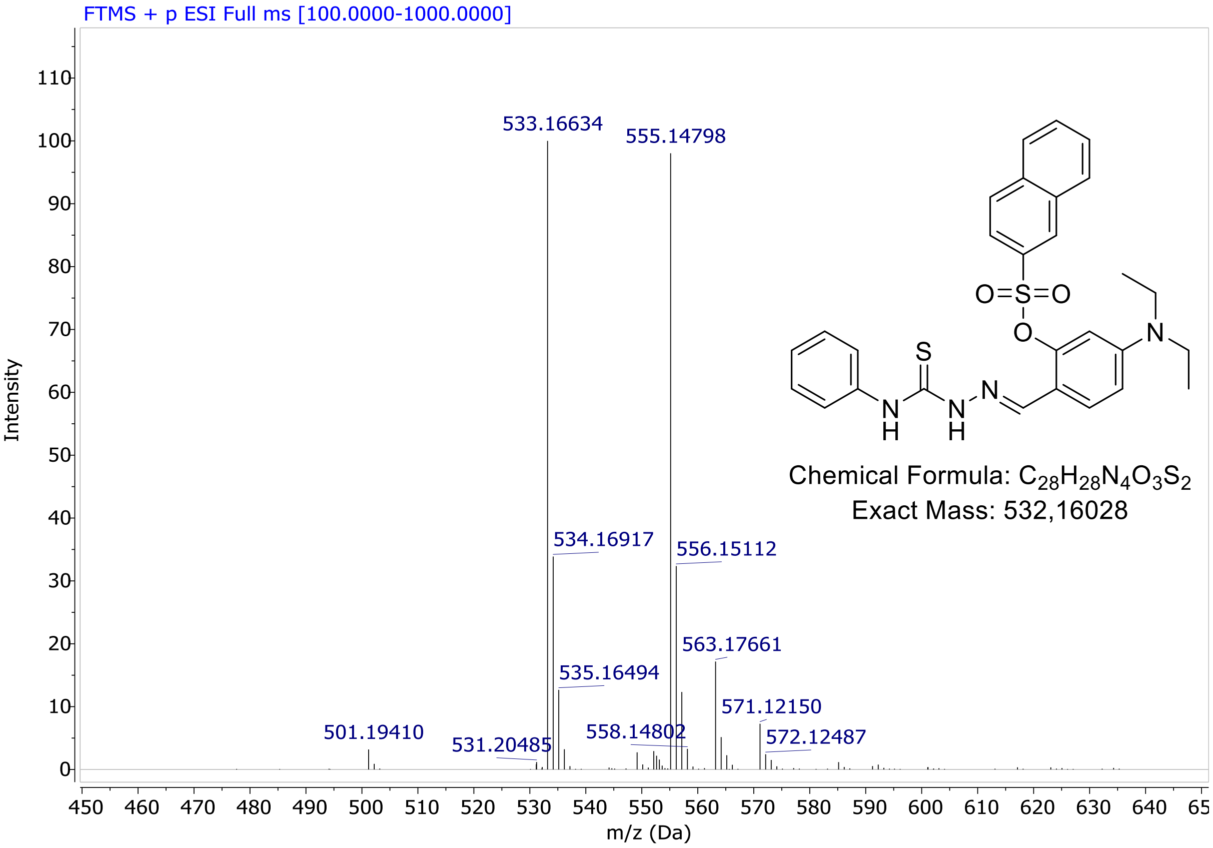

Figure S 17. ESI-HRMS Spectrum of Compound **5c**

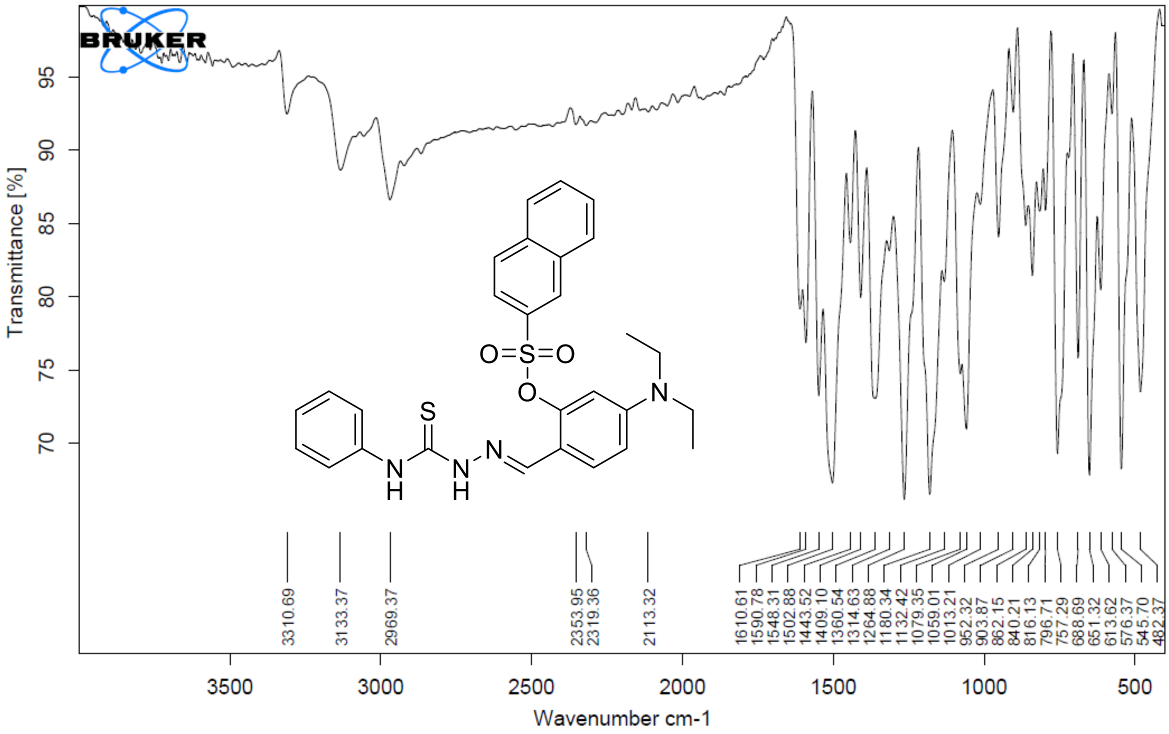

Figure S 18. FT-IR Spectrum of Compound **5c**

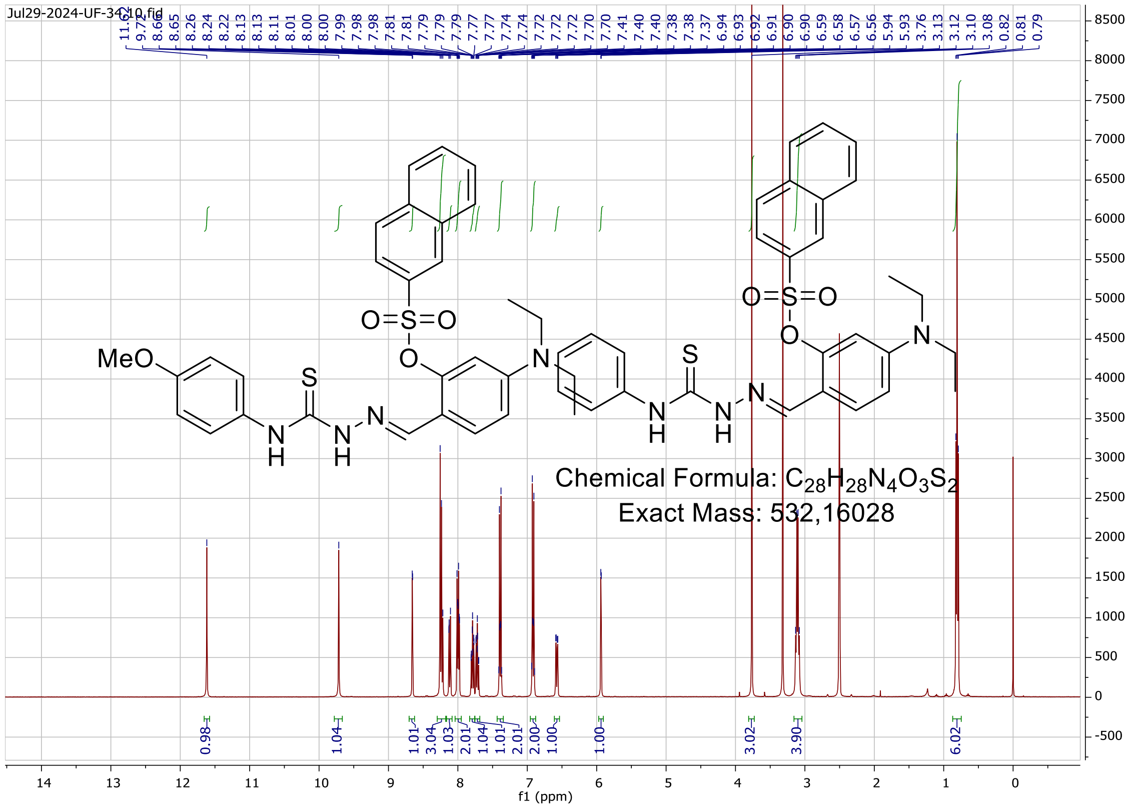

Figure S 19. ^{1}H-NMR Spectrum of Compound **5d** (DMSO-*d6*, 400 MHz)

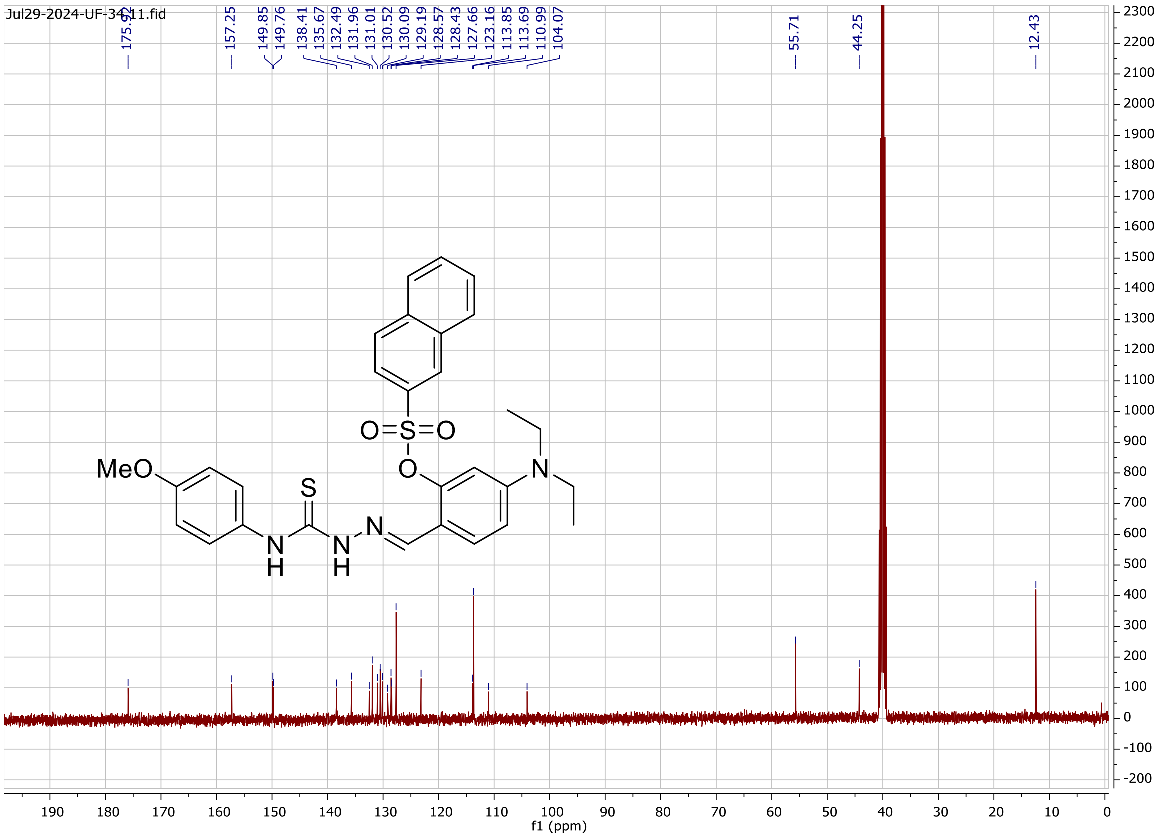

Figure S 20. ^{13}C-NMR Spectrum of Compound **5d** (DMSO-*d6*, 100 MHz)

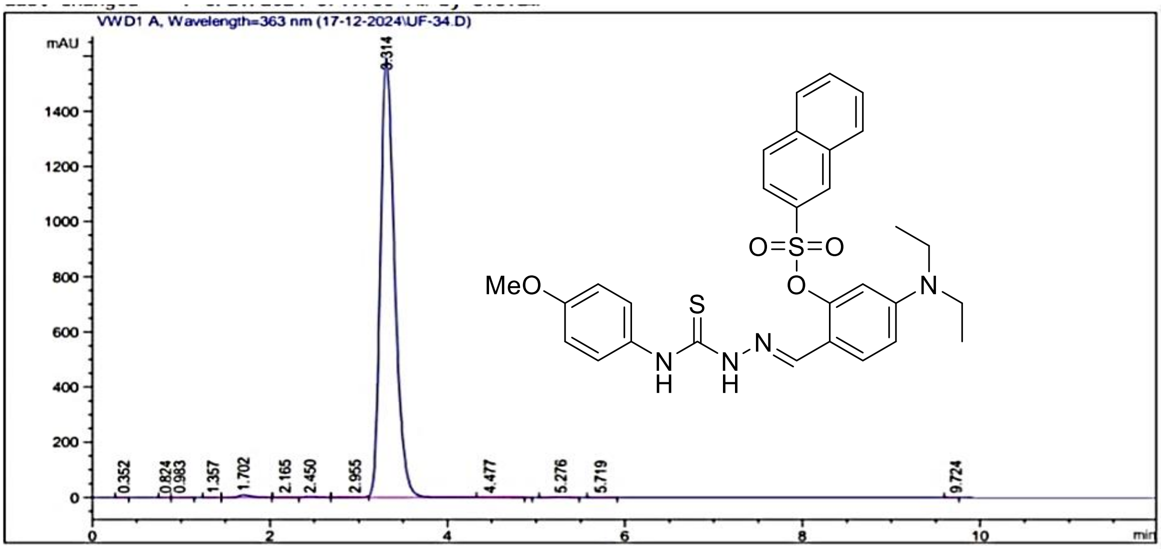

==

Area Percent Report

==

Sorted By : Signal
Multiplier : 1.0000
Dilution : 1.0000
Use Multiplier & Dilution Factor with ISTDs

Signal 1: VWD1 A, Wavelength=363 nm

Peak #	RetTime [min]	Type	Width [min]	Area [mAU*s]	Height [mAU]	Area %
1	0.352	BV	0.0679	1.31698e-1	2.87833e-2	7.366e-4
2	0.824	BB	0.0807	2.75594e-1	5.33244e-2	1.541e-3
3	0.983	BB	0.0818	3.70976e-1	6.29909e-2	2.075e-3
4	1.357	BV	0.0874	5.63858e-1	8.68938e-2	3.154e-3
5	1.702	VB	0.1424	75.30840	8.07019	0.4212
6	2.165	BV E	0.1123	2.72918	3.57561e-1	0.0153
7	2.450	VB R	0.1388	27.12757	3.00500	0.1517
8	2.955	BV E	0.1654	16.13267	1.44229	0.0902
9	3.314	VV R	0.1693	1.77502e4	1592.52966	99.2750
10	4.477	VB E	0.1926	3.63274	2.39997e-1	0.0203

Peak #	RetTime [min]	Type	Width [min]	Area [mAU*s]	Height [mAU]	Area %
11	5.276	BB	0.1685	2.09760	1.75560e-1	0.0117
12	5.719	BB	0.1400	1.06263	9.54864e-2	5.943e-3
13	9.724	BB	0.0895	1.92650e-1	2.78348e-2	1.077e-3

Totals : 1.78798e4 1606.17557

Figure S 21. HPLC Purity Analysis of Compound **5d**

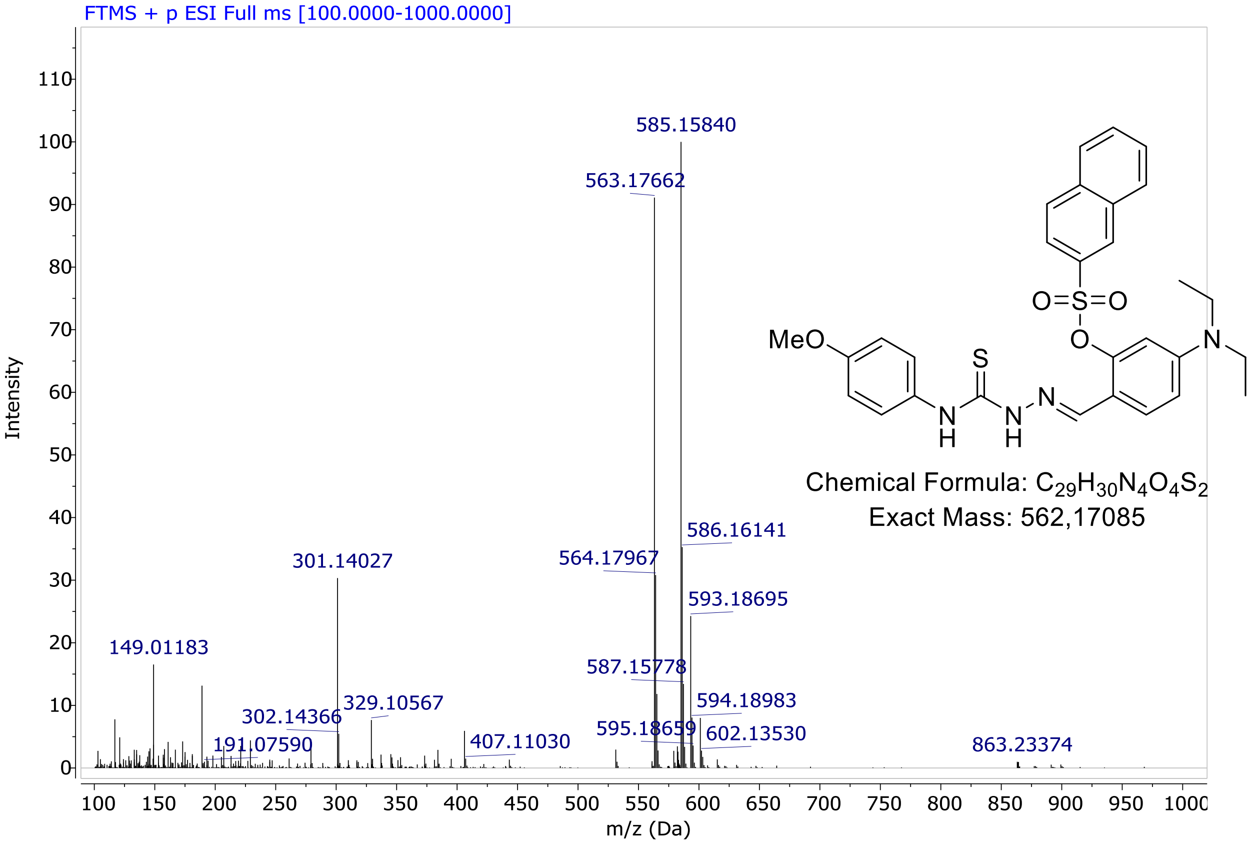

Figure S 22. ESI-HRMS Spectrum of Compound **5d**

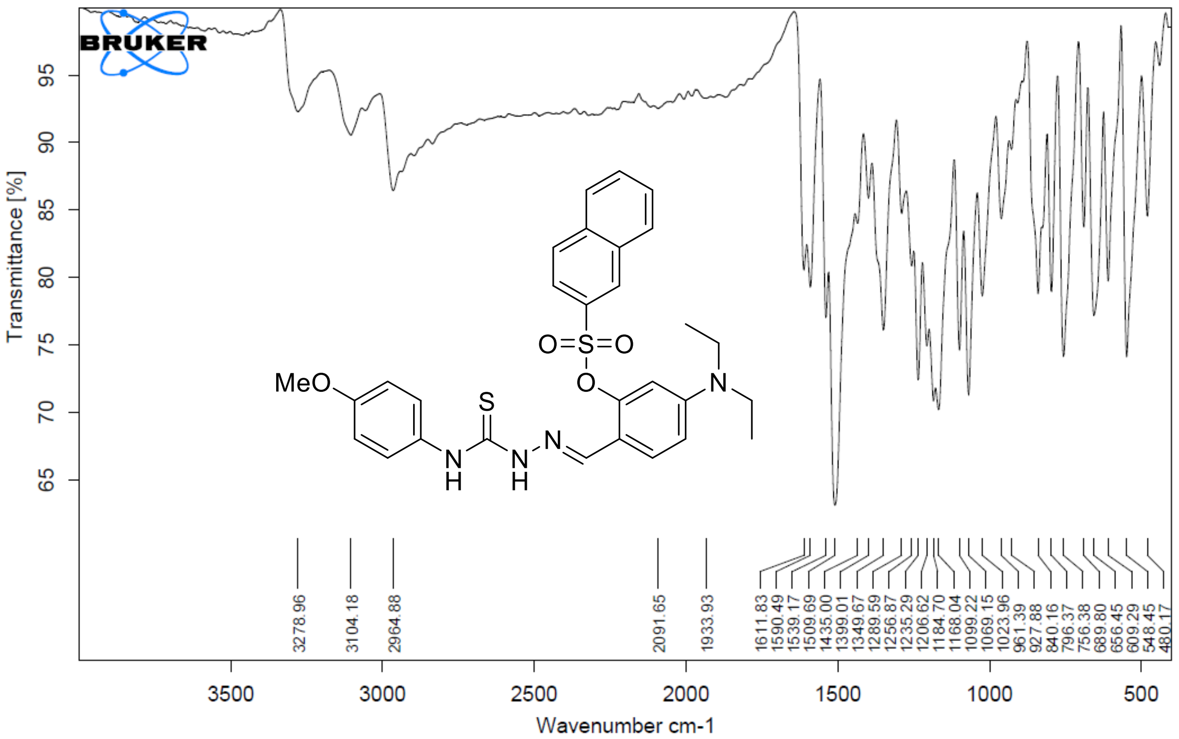

Figure S 23. FT-IR Spectrum of Compound **5d**

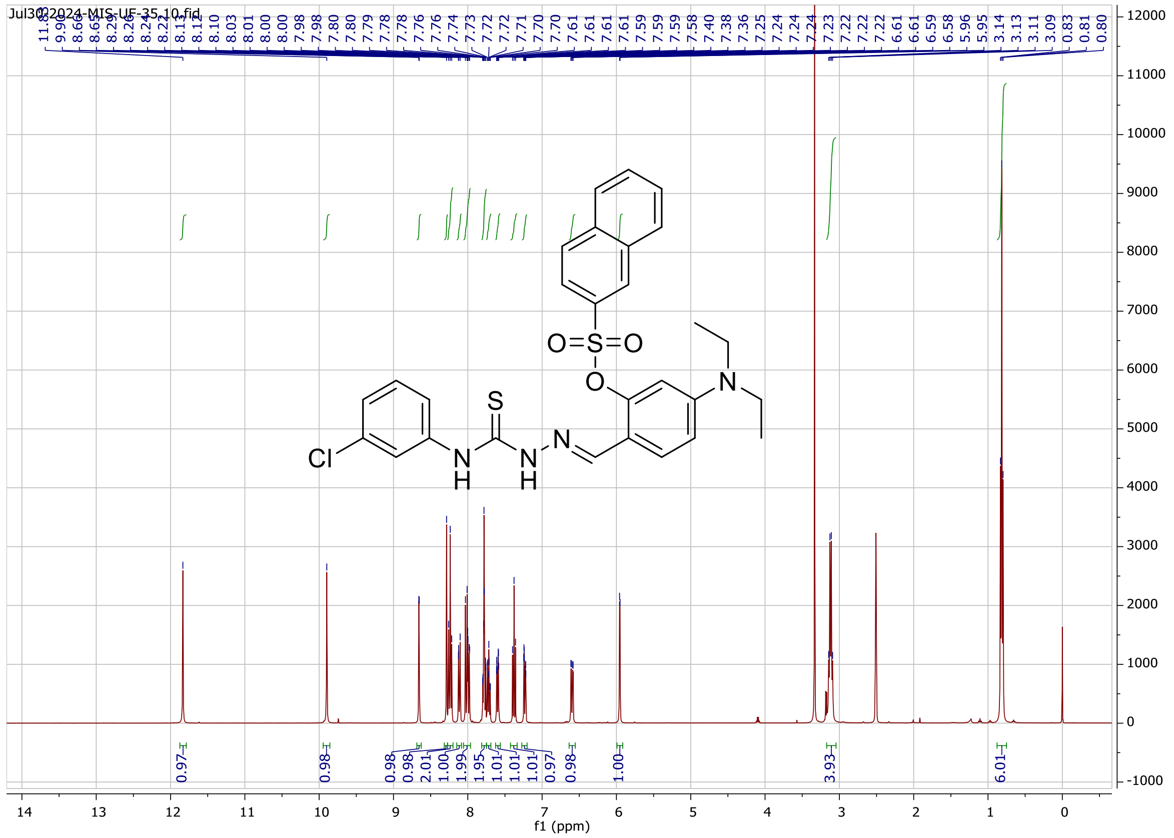

Figure S 24. ^{1}H-NMR Spectrum of Compound **5e** (DMSO-*d6*, 400 MHz)

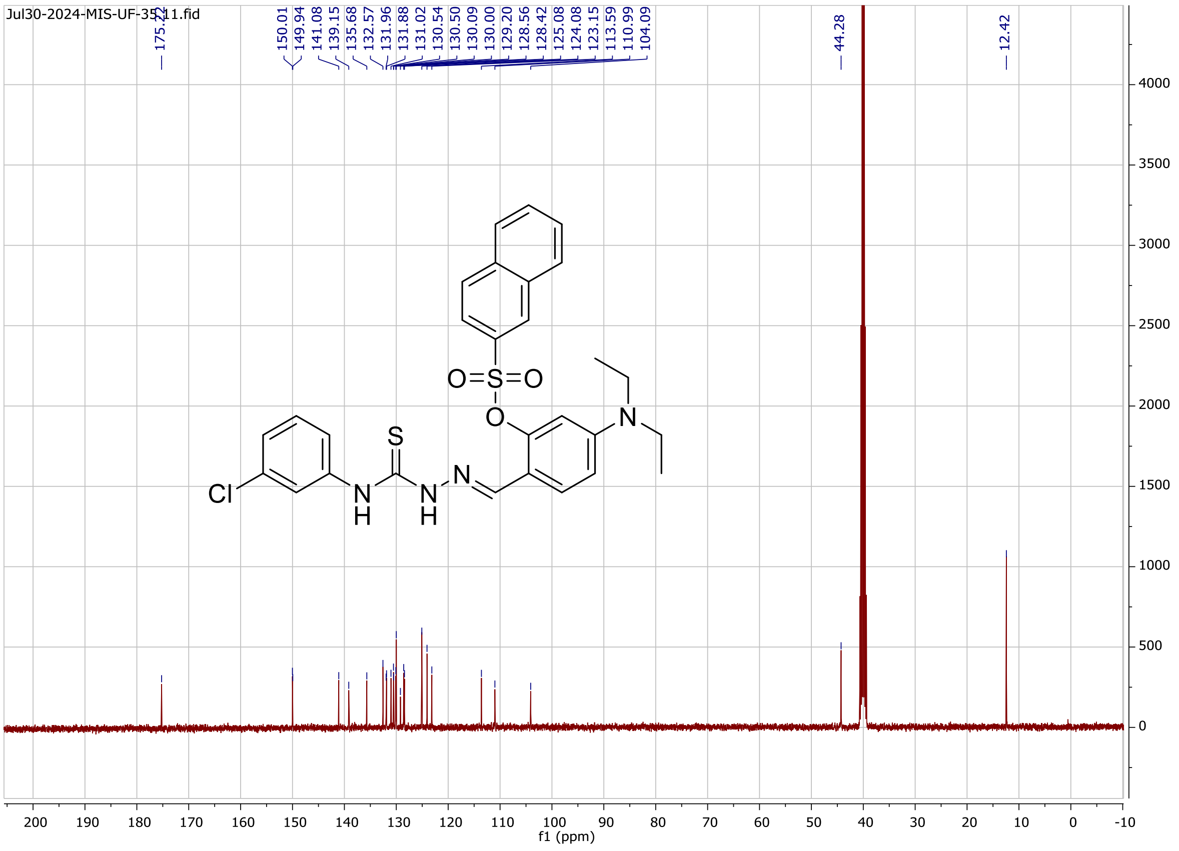

Figure S 25. ^{13}C-NMR Spectrum of Compound **5e** (DMSO-*d6*, 100 MHz)

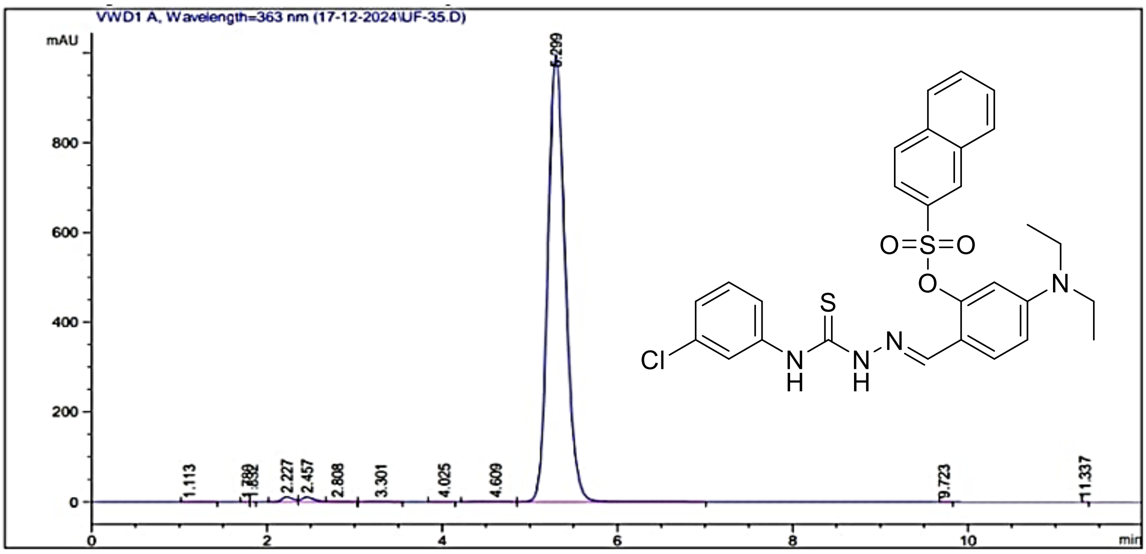

Area Percent Report

Sorted By : Signal
Multiplier : 1.0000
Dilution : 1.0000
Use Multiplier & Dilution Factor with ISTDs

Signal 1: VWD1 A, Wavelength=363 nm

Peak #	RetTime [min]	Type	Width [min]	Area [mAU*s]	Height [mAU]	Area %
1	1.113	BB	0.1303	6.28713e-1	6.10870e-2	4.541e-3
2	1.780	BV	0.0584	1.66930e-1	4.04186e-2	1.206e-3
3	1.832	VB	0.0393	7.27133e-2	2.91477e-2	5.252e-4
4	2.227	BV	0.1350	92.44135	10.62140	0.6676
5	2.457	VV R	0.1488	101.43955	10.26612	0.7326
6	2.808	VB E	0.1397	3.73237	3.93933e-1	0.0270
7	3.301	BB	0.1992	4.46080	3.16840e-1	0.0322
8	4.025	BB	0.1268	1.09115	1.16476e-1	7.881e-3
9	4.609	BV	0.2217	11.70080	8.06461e-1	0.0845
10	5.299	VB	0.2108	1.36300e4	995.16412	98.4406

HPLC 12/17/2024 3:09:29 PM SYSTEM Page 1 of 2

Data File D:\HPLC-DATA\Data\17-12-2024\UF-35.D
Sample Name: UF-35

Peak #	RetTime [min]	Type	Width [min]	Area [mAU*s]	Height [mAU]	Area %
11	9.723	BB	0.0587	1.11746e-1	2.95632e-2	8.071e-4
12	11.337	VB	0.0434	7.36662e-2	2.83354e-2	5.320e-4

Totals : 1.38460e4 1017.87391

Figure S 26. HPLC Purity Analysis of Compound **5e**

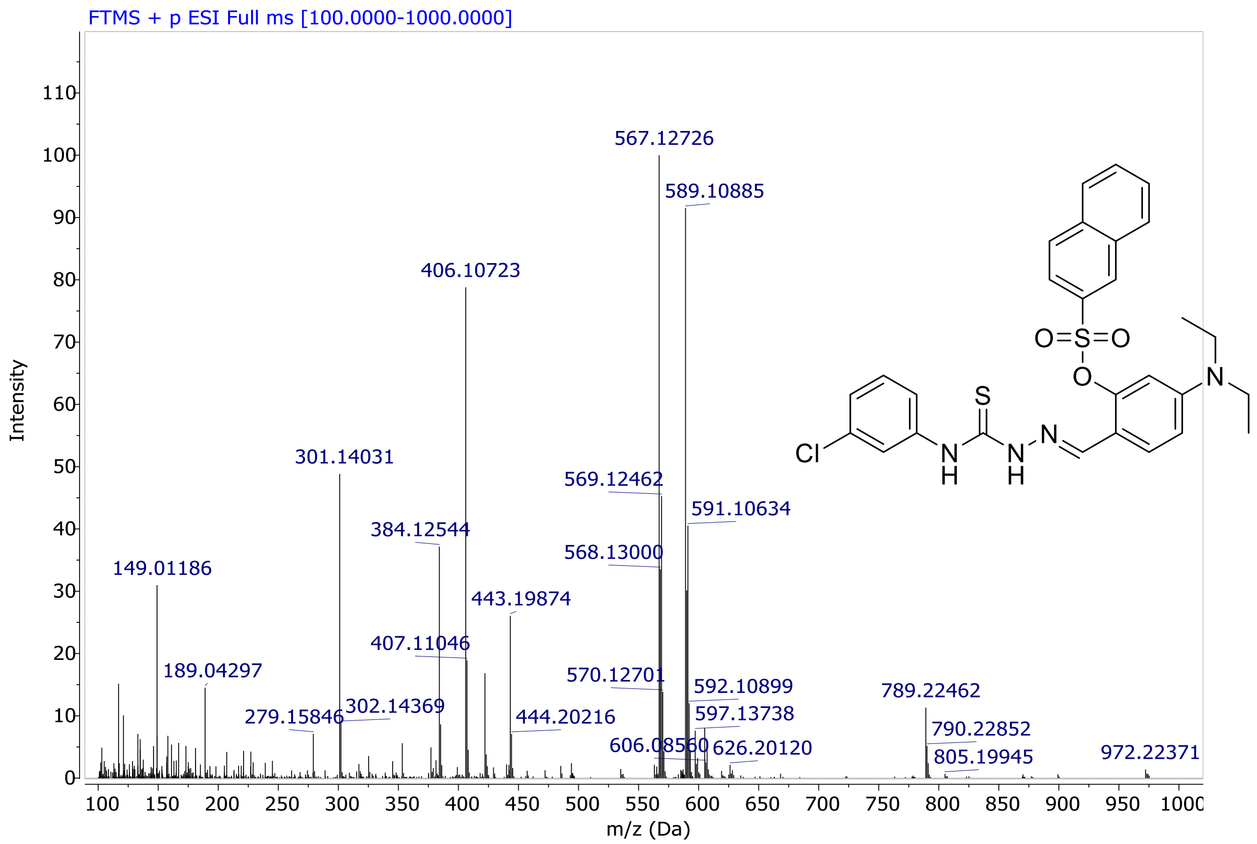

Figure S 27. ESI-HRMS Spectrum of Compound **5e**

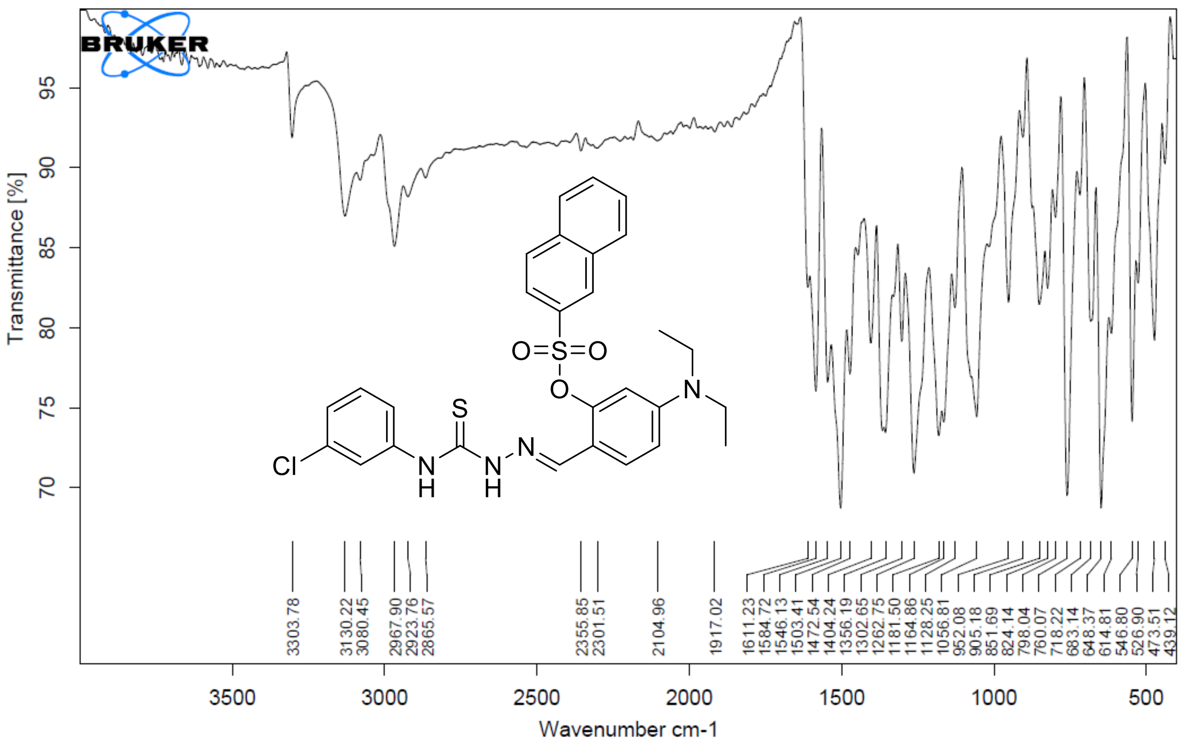

Figure S 28. FT-IR Spectrum of Compound **5e**

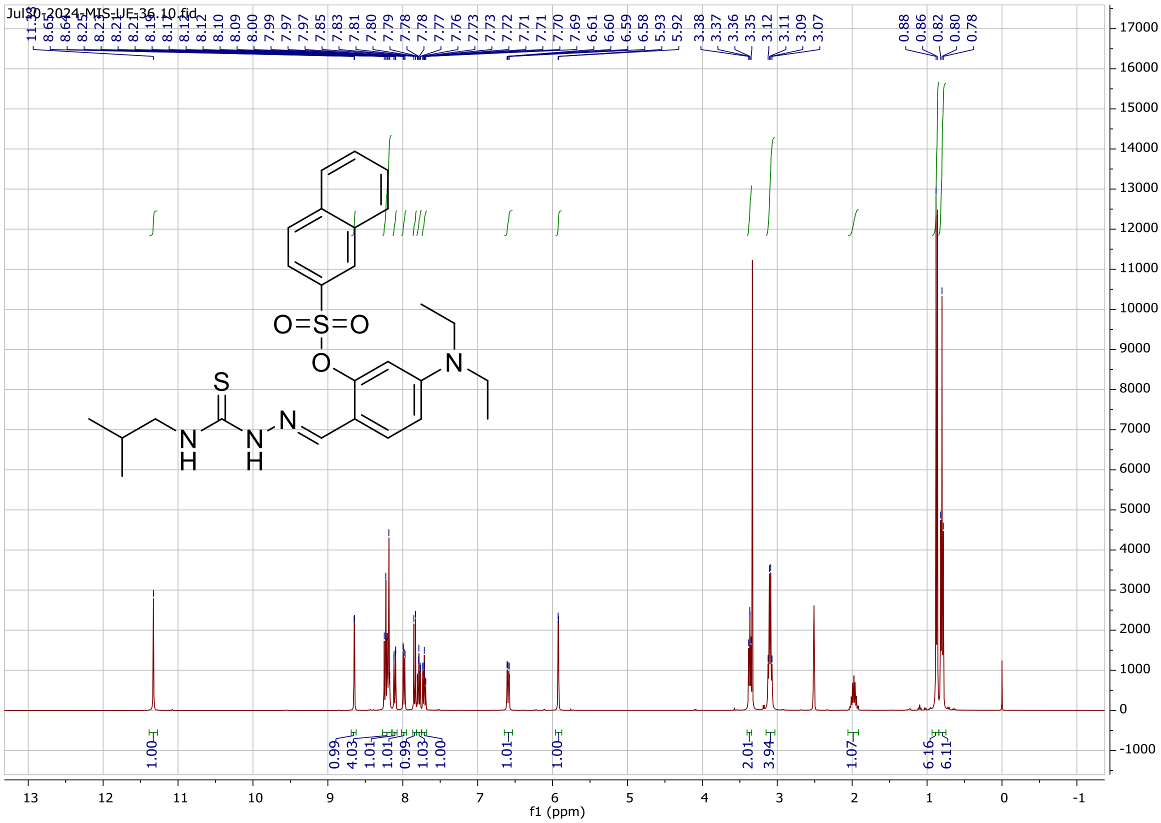

Figure S 29. ^{1}H-NMR Spectrum of Compound **5f** (DMSO-*d6*, 400 MHz)

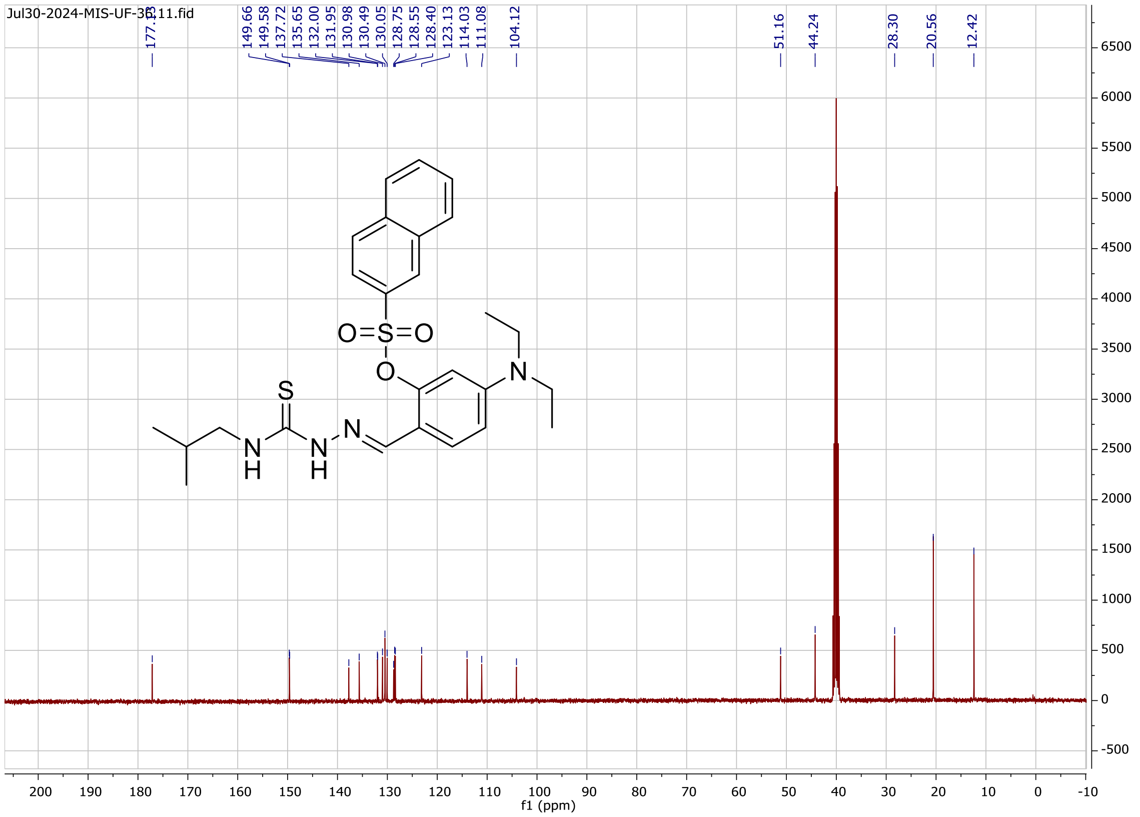

Figure S 30. ^{13}C-NMR Spectrum of Compound **5f** (DMSO-*d6*, 100 MHz)

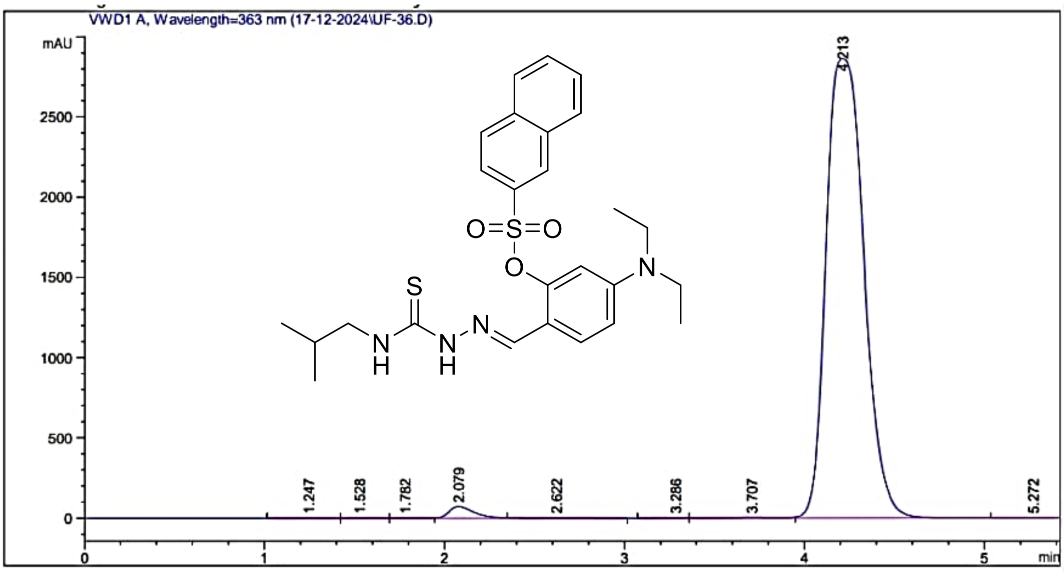

==

Area Percent Report

==

Sorted By : Signal
Multiplier : 1.0000
Dilution : 1.0000
Use Multiplier & Dilution Factor with ISTDs

Signal 1: VWD1 A, Wavelength=363 nm

Peak #	RetTime [min]	Type	Width [min]	Area [mAU*s]	Height [mAU]	Area %
1	1.247	BV E	0.1562	5.16884	4.58803e-1	0.0122
2	1.528	VV E	0.1397	11.45072	1.22425	0.0270
3	1.782	VV E	0.1429	7.14915	6.89059e-1	0.0168
4	2.079	VV R	0.1422	672.89374	72.23827	1.5857
5	2.622	VB E	0.2274	14.01569	8.24827e-1	0.0330
6	3.286	BV E	0.1593	8.30166	8.17429e-1	0.0196
7	3.707	VV E	0.2189	57.19376	3.77738	0.1348
8	4.213	VV R	0.2295	4.16524e4	2862.61401	98.1530
9	5.272	VBAE	0.2046	7.64036	5.74665e-1	0.0180

HPLC 12/17/2024 4:27:20 PM SYSTEM Page 1 of 2

Data File D:\HPLC-DATA\Data\17-12-2024\UF-36.D
Sample Name: UF-36

Totals : 4.24362e4 2943.21869

==

*** End of Report ***

Figure S 31. HPLC Purity Analysis of Compound **5f**

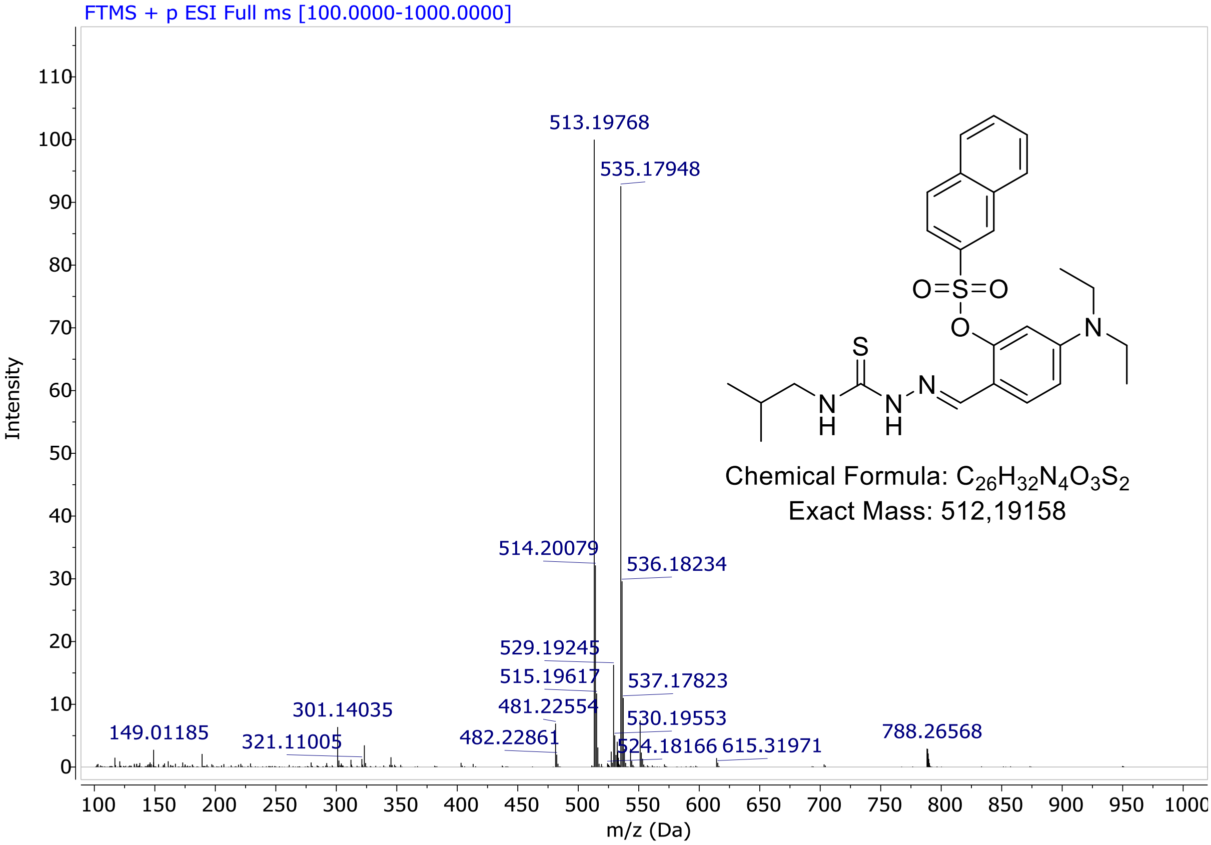

Figure S 32. ESI-HRMS Spectrum of Compound **5f**

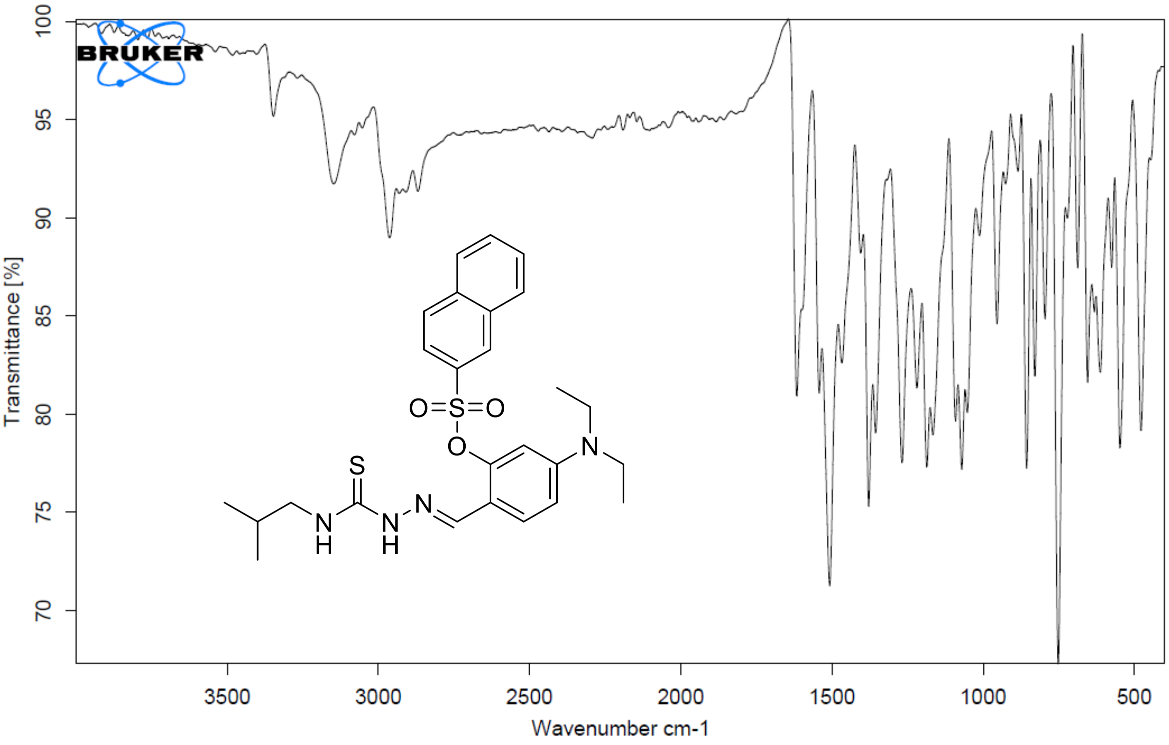

Figure S 33. FT-IR Spectrum of Compound **5f**

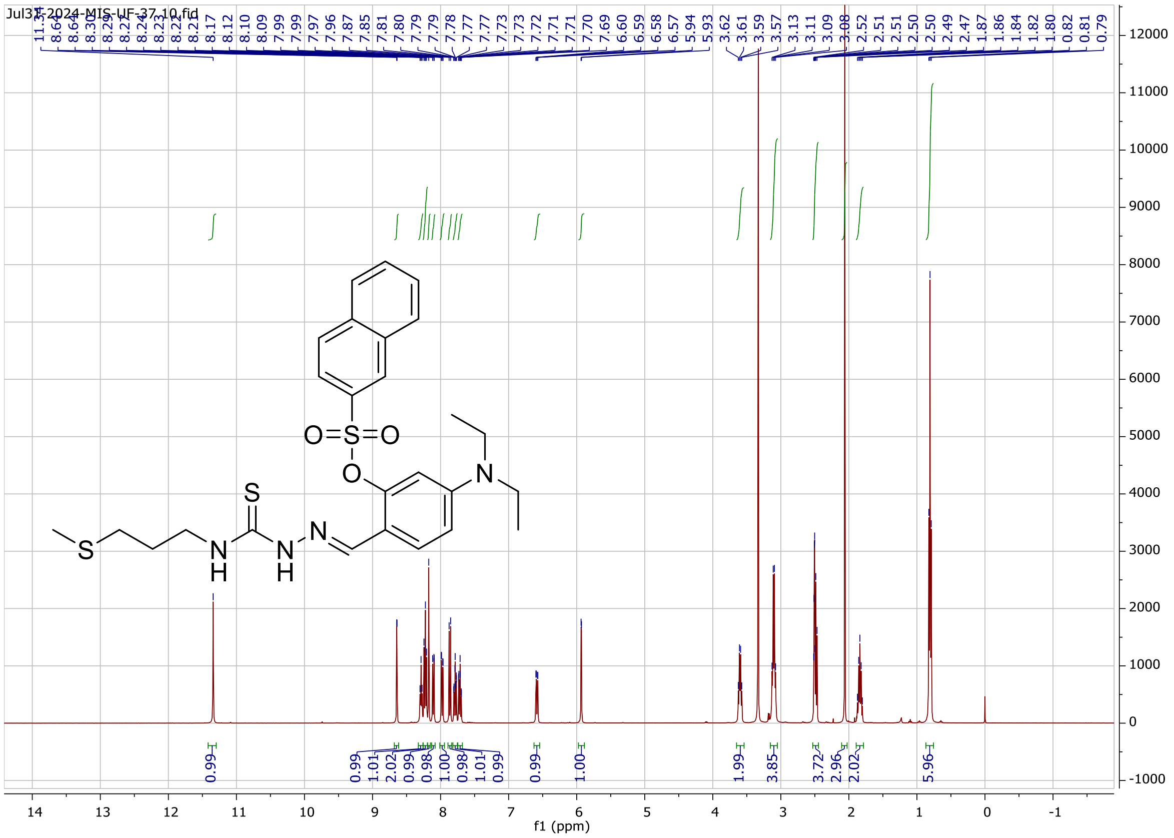

Figure S 34. ^{1}H-NMR Spectrum of Compound **5g** (DMSO-*d6*, 400 MHz)

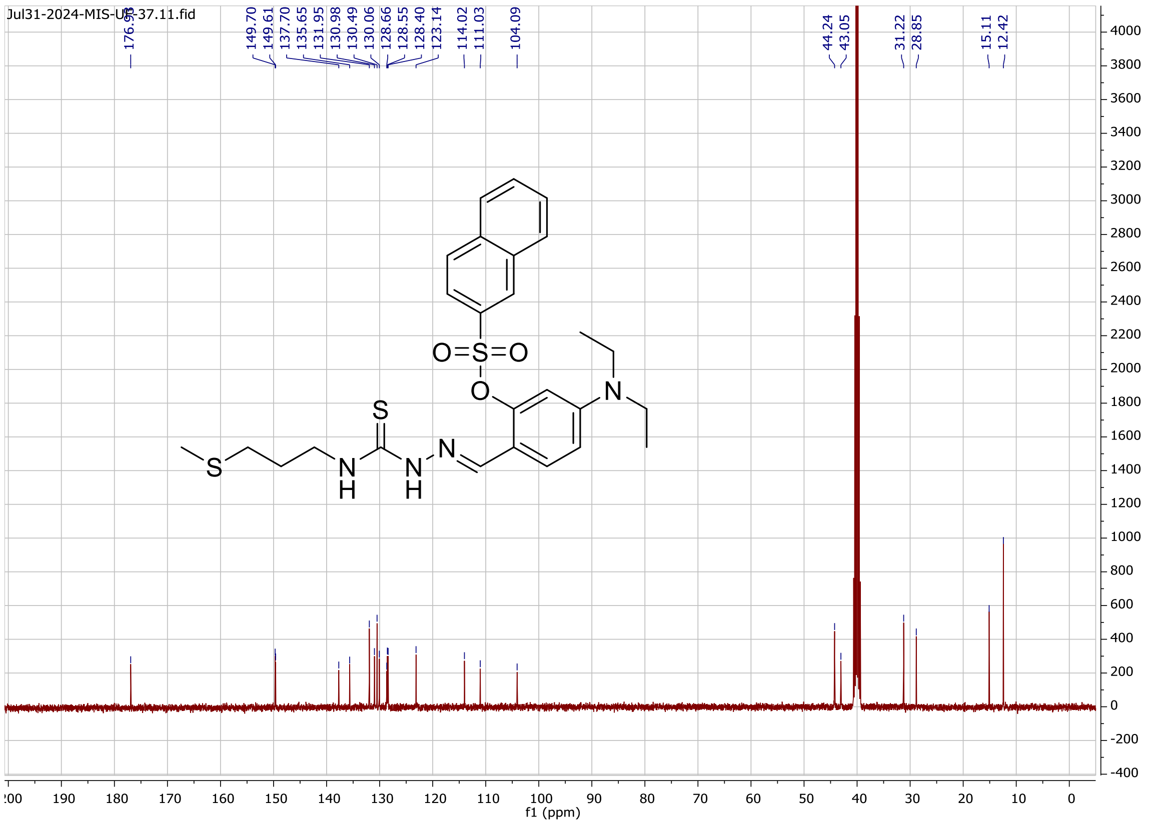

Figure S 35. ^{13}C-NMR Spectrum of Compound **5g** (DMSO-*d6*, 100 MHz)

```
=====================================================================
Acq. Operator   : SYSTEM
Sample Operator : SYSTEM
Acq. Instrument : HPLC                          Location :     -
Injection Date  : 12/17/2024 4:27:45 PM              Inj :    1
                                               Inj Volume : No inj
Method          : D:\HPLC-DATA\Method\BZ-11.M
Last changed    : 5/29/2024 5:19:35 PM by SYSTEM
```

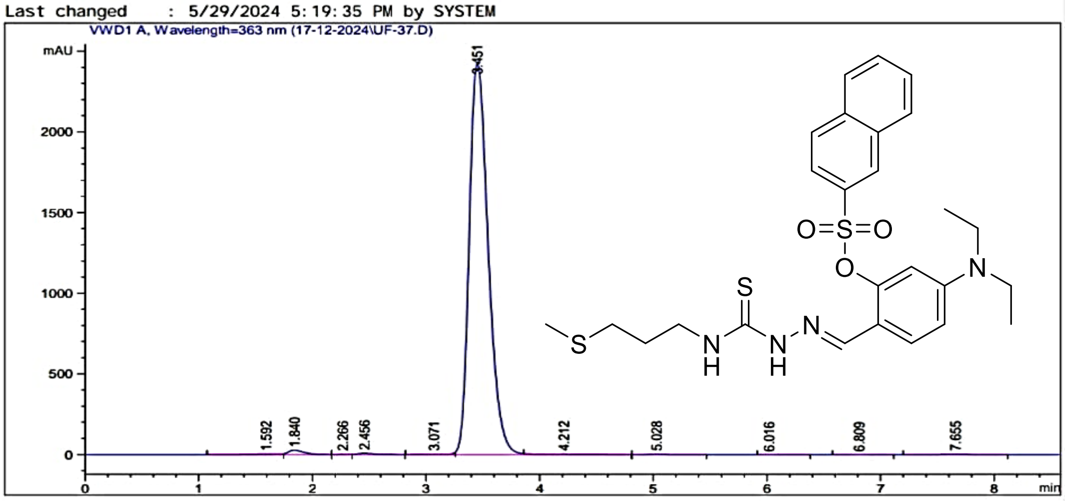


```
=====================================================================
                         Area Percent Report
=====================================================================

Sorted By              :      Signal
Multiplier             :      1.0000
Dilution               :      1.0000
Use Multiplier & Dilution Factor with ISTDs


Signal 1: VWD1 A, Wavelength=363 nm
```

Peak #	RetTime [min]	Type	Width [min]	Area [mAU*s]	Height [mAU]	Area %
1	1.592	BV E	0.1908	50.48253	4.12608	0.1767
2	1.840	VB R	0.1417	256.18890	27.26139	0.8967
3	2.266	BV E	0.0862	9.94358e-1	1.76273e-1	3.481e-3
4	2.456	VB R	0.1455	71.83395	7.58185	0.2514
5	3.071	BV E	0.1690	27.75907	2.44159	0.0972
6	3.451	VV R	0.1789	2.80901e4	2423.61255	98.3231
7	4.212	VB E	0.2604	46.22150	2.50480	0.1618
8	5.028	BB	0.2165	5.53470	3.13004e-1	0.0194
9	6.016	BB	0.2022	8.98860e-1	5.54677e-2	3.146e-3
10	6.809	BB	0.1949	2.47214	1.57429e-1	8.653e-3

HPLC 12/17/2024 4:36:28 PM SYSTEM   Page 1 of 2

Data File D:\HPLC-DATA\Data\17-12-2024\UF-37.D
Sample Name: UF-37

Peak #	RetTime [min]	Type	Width [min]	Area [mAU*s]	Height [mAU]	Area %
11	7.655	BV	0.2645	16.70424	9.25067e-1	0.0585

Totals : 2.85692e4 2469.15550

Figure S 36. HPLC Purity Analysis of Compound **5g**

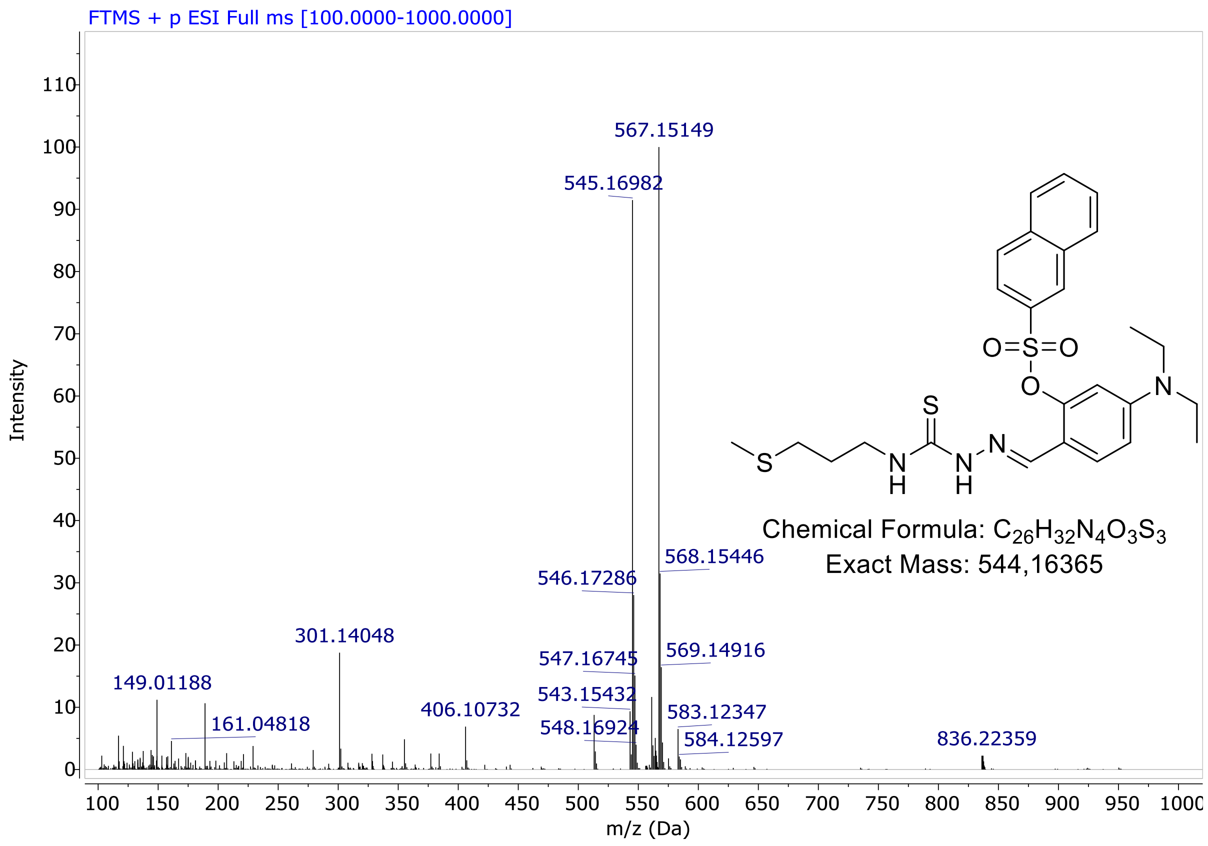

Figure S 37. ESI-HRMS Spectrum of Compound **5g**

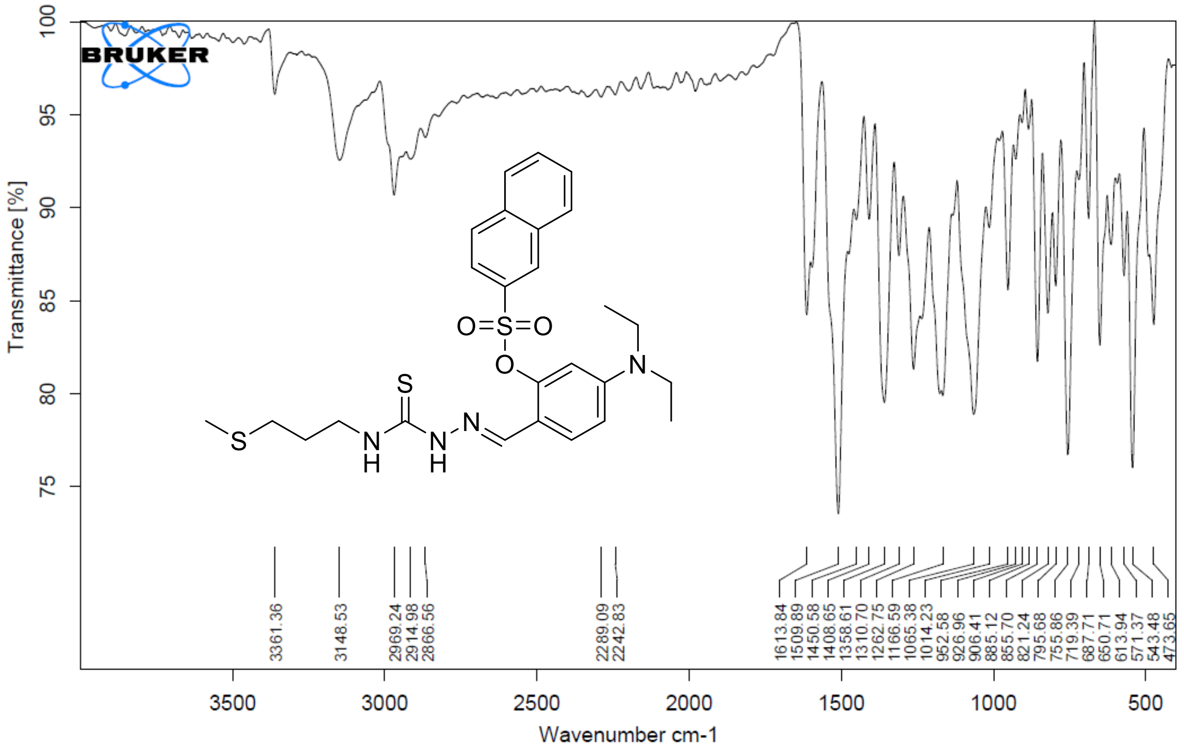

Figure S 38. FT-IR Spectrum of Compound **5g**

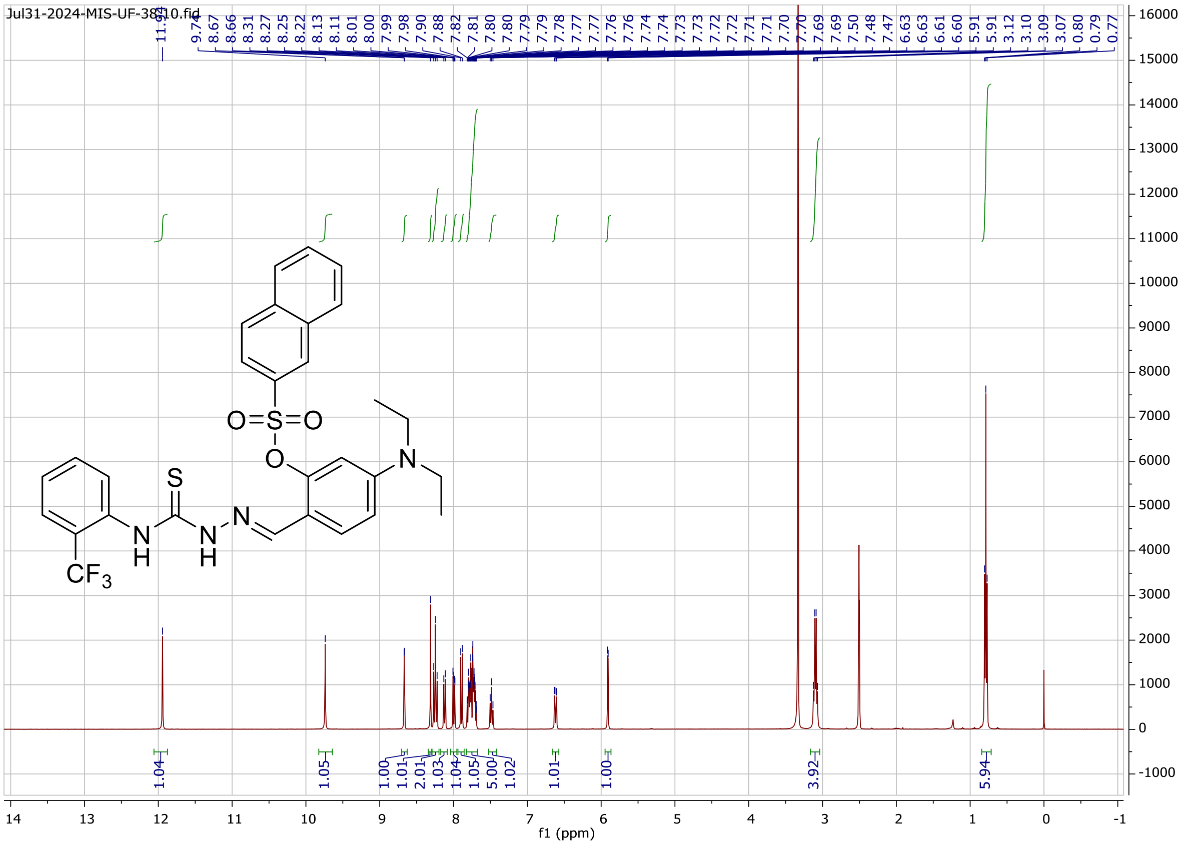

Figure S 39. ^{1}H-NMR Spectrum of Compound **5h** (DMSO-*d6*, 400 MHz)

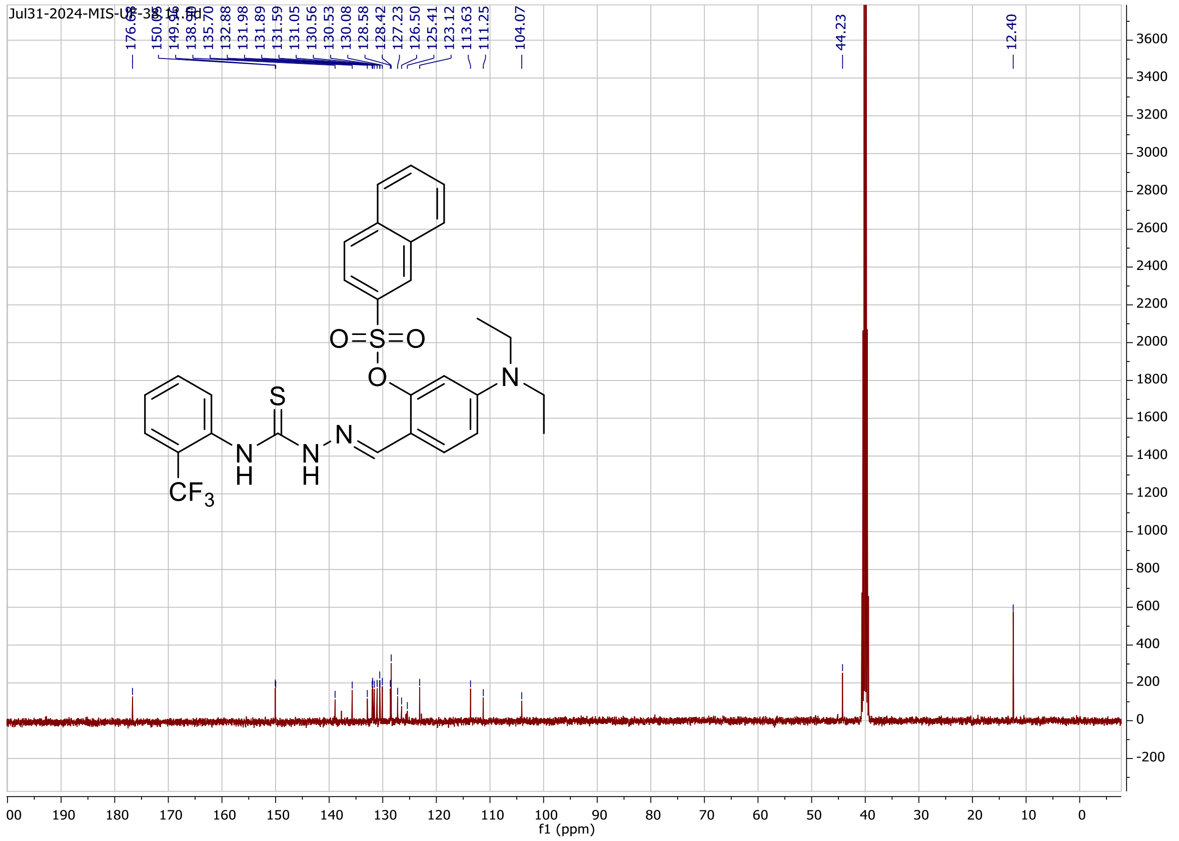

Figure S 40. ^{13}C-NMR Spectrum of Compound **5h** (DMSO-*d6*, 100 MHz)

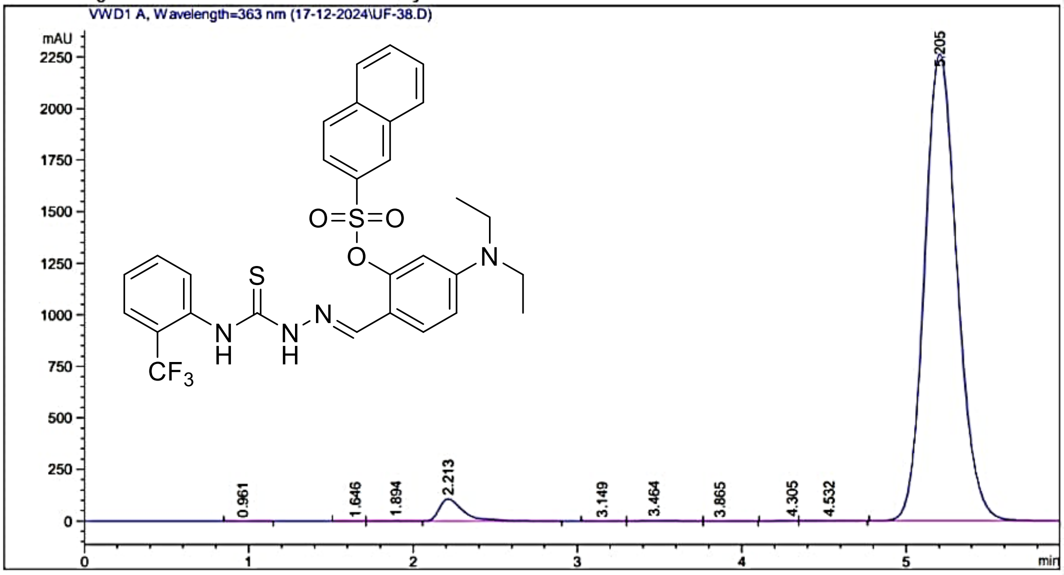

===
Area Percent Report
===

Sorted By : Signal
Multiplier : 1.0000
Dilution : 1.0000
Use Multiplier & Dilution Factor with ISTDs

Signal 1: VWD1 A, Wavelength=363 nm

Peak #	RetTime [min]	Type	Width [min]	Area [mAU*s]	Height [mAU]	Area %
1	0.961	BB	0.1183	4.54997e-1	5.42401e-2	1.408e-3
2	1.646	BV E	0.1223	1.45942	1.88630e-1	4.516e-3
3	1.894	VV E	0.1749	4.18585	3.52639e-1	0.0130
4	2.213	VB R	0.1509	1074.85986	106.79336	3.3262
5	3.149	BV	0.1512	2.09048	2.15234e-1	6.469e-3
6	3.464	VV	0.1984	8.78822	6.21574e-1	0.0272
7	3.865	VB	0.1464	2.59139	2.24413e-1	8.019e-3
8	4.305	BV	0.1177	4.29800	5.39300e-1	0.0133
9	4.532	VB	0.1889	23.69898	1.88520	0.0733
10	5.205	BBA	0.2131	3.11924e4	2264.61475	96.5266

HPLC 12/17/2024 4:43:29 PM SYSTEM Page 1 of 2

Data File D:\HPLC-DATA\Data\17-12-2024\UF-38.D
Sample Name: UF-38

Peak #	RetTime [min]	Type	Width [min]	Area [mAU*s]	Height [mAU]	Area %
Totals :				3.23148e4	2375.48934	

Figure S 41. HPLC Purity Analysis of Compound **5h**

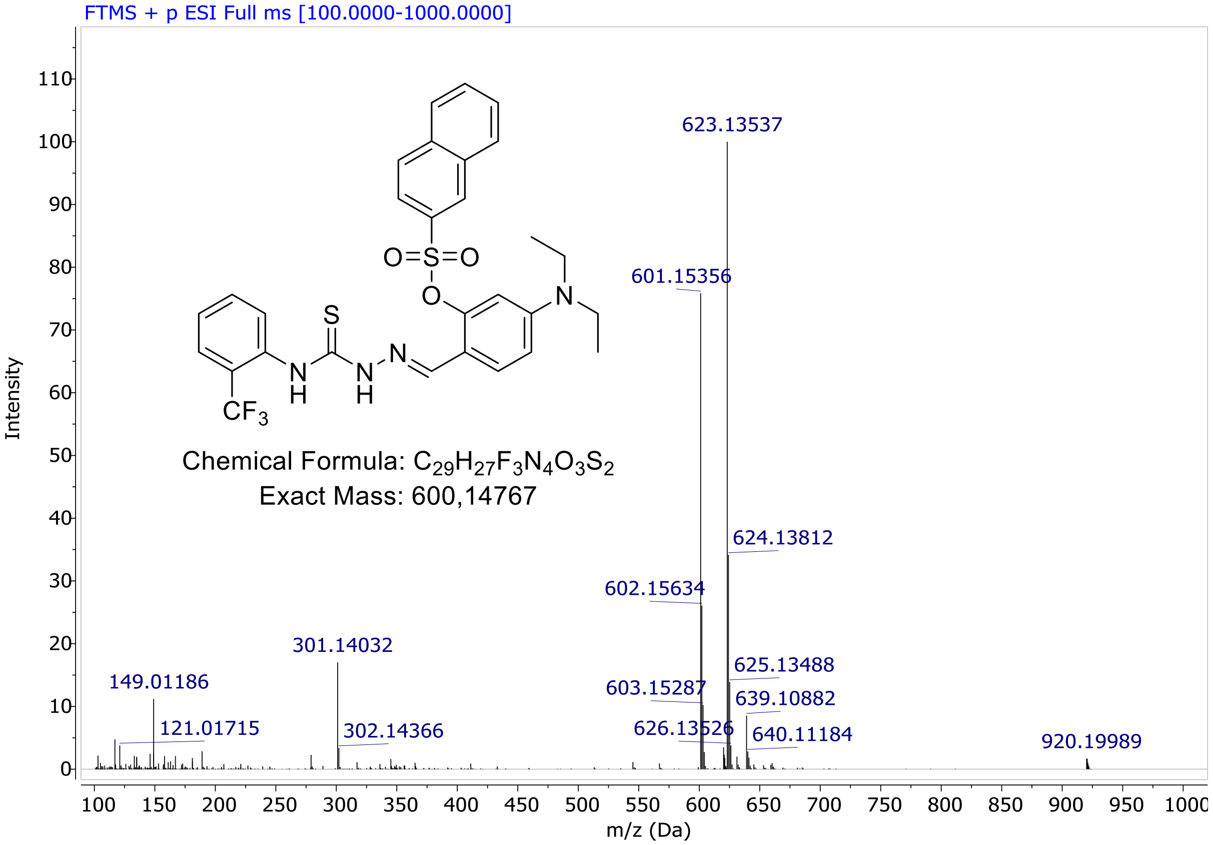

Figure S 42. ESI-HRMS Spectrum of Compound **5h**

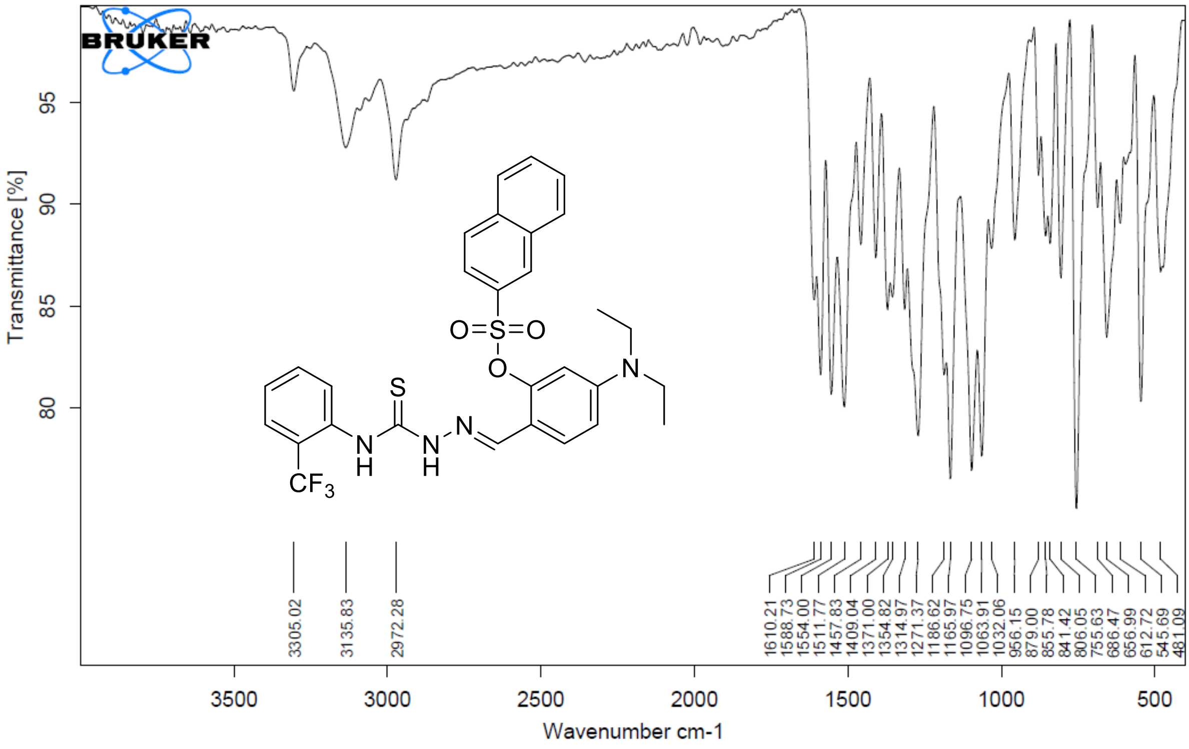

Figure S 43. FT-IR Spectrum of Compound **5h**

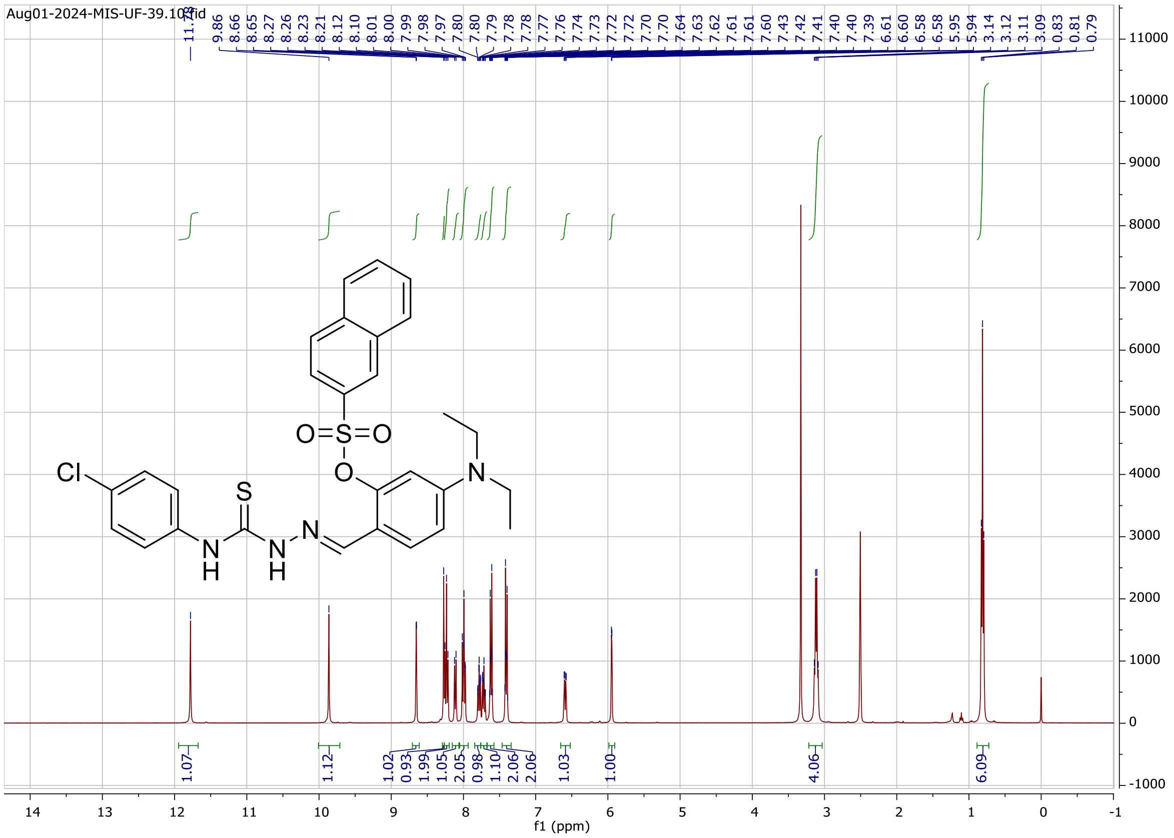

Figure S 44. ^{1}H-NMR Spectrum of Compound **5i** (DMSO-*d6*, 400 MHz)

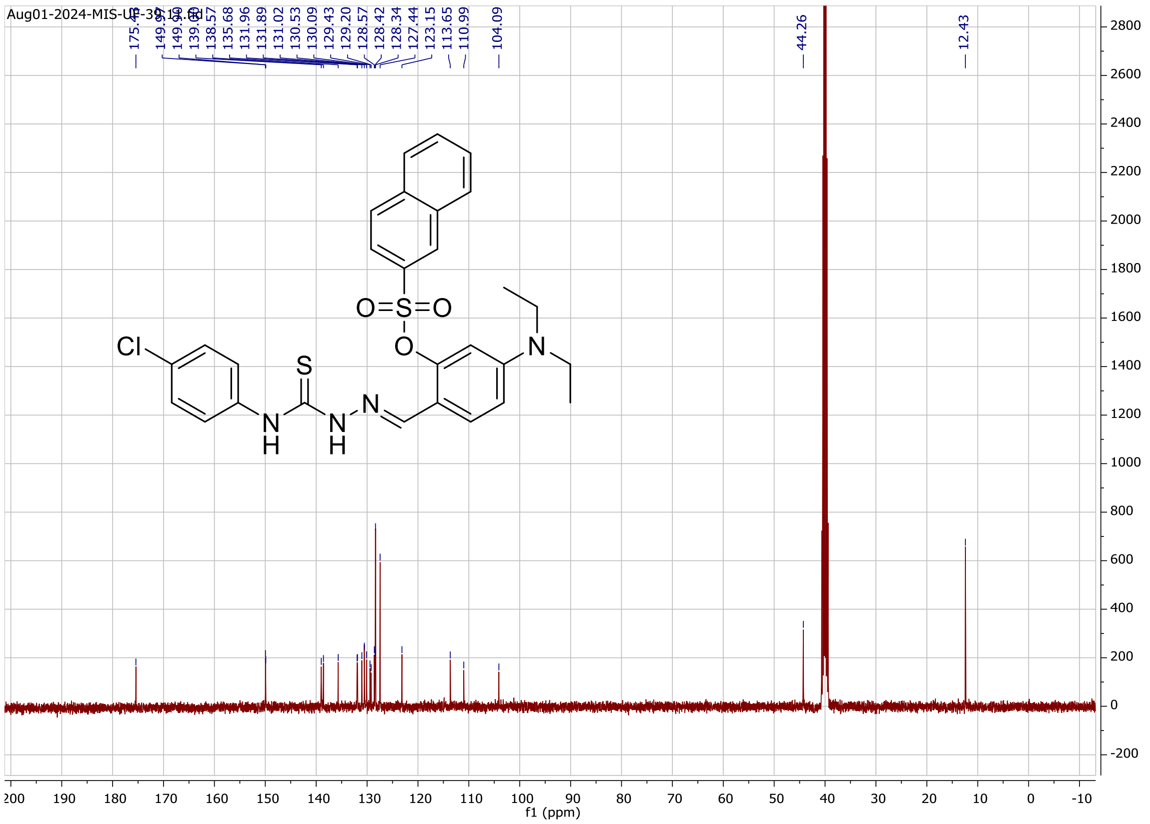

Figure S 45. ^{13}C-NMR Spectrum of Compound **5i** (DMSO-*d6*, 100 MHz)

Method : D:\HPLC-DATA\Method\BZ-11.M
Last changed : 5/29/2024 5:19:35 PM by SYSTEM

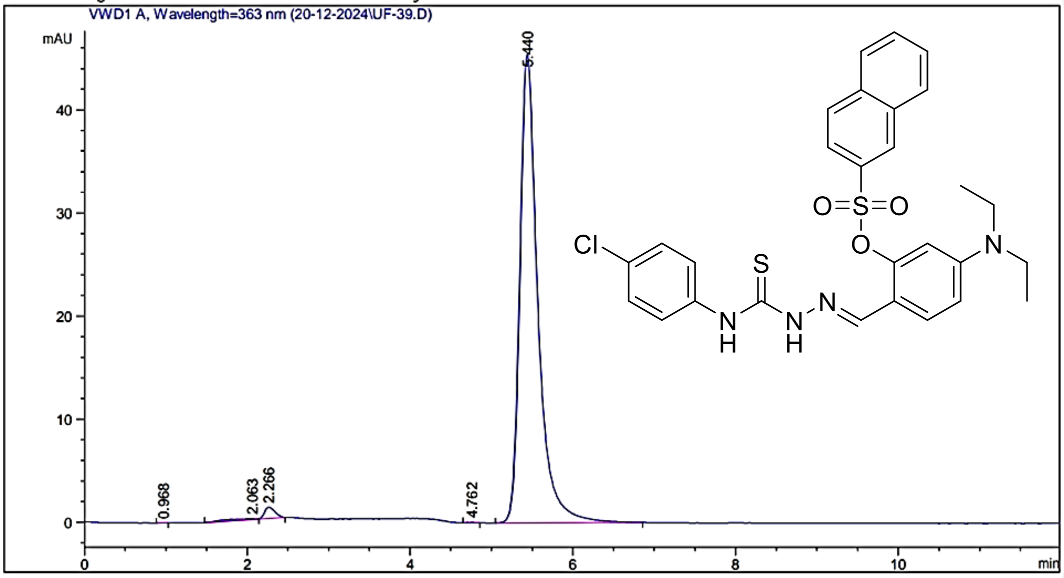

==

Area Percent Report

==

Sorted By : Signal
Multiplier : 1.0000
Dilution : 1.0000
Use Multiplier & Dilution Factor with ISTDs

Signal 1: VWD1 A, Wavelength=363 nm

Peak #	RetTime [min]	Type	Width [min]	Area [mAU*s]	Height [mAU]	Area %
1	0.968	BB	0.0680	1.71663e-1	3.22127e-2	0.0242
2	2.063	BV E	0.6392	3.38553	6.26534e-2	0.4767
3	2.266	VB R	0.1423	9.68887	1.08226	1.3641
4	4.762	BB	0.0850	1.96241e-1	3.18474e-2	0.0276
5	5.440	BB	0.2269	696.80988	45.46298	98.1074

Totals : 710.25217 46.67195

Figure S 46. HPLC Purity Analysis of Compound **5i**

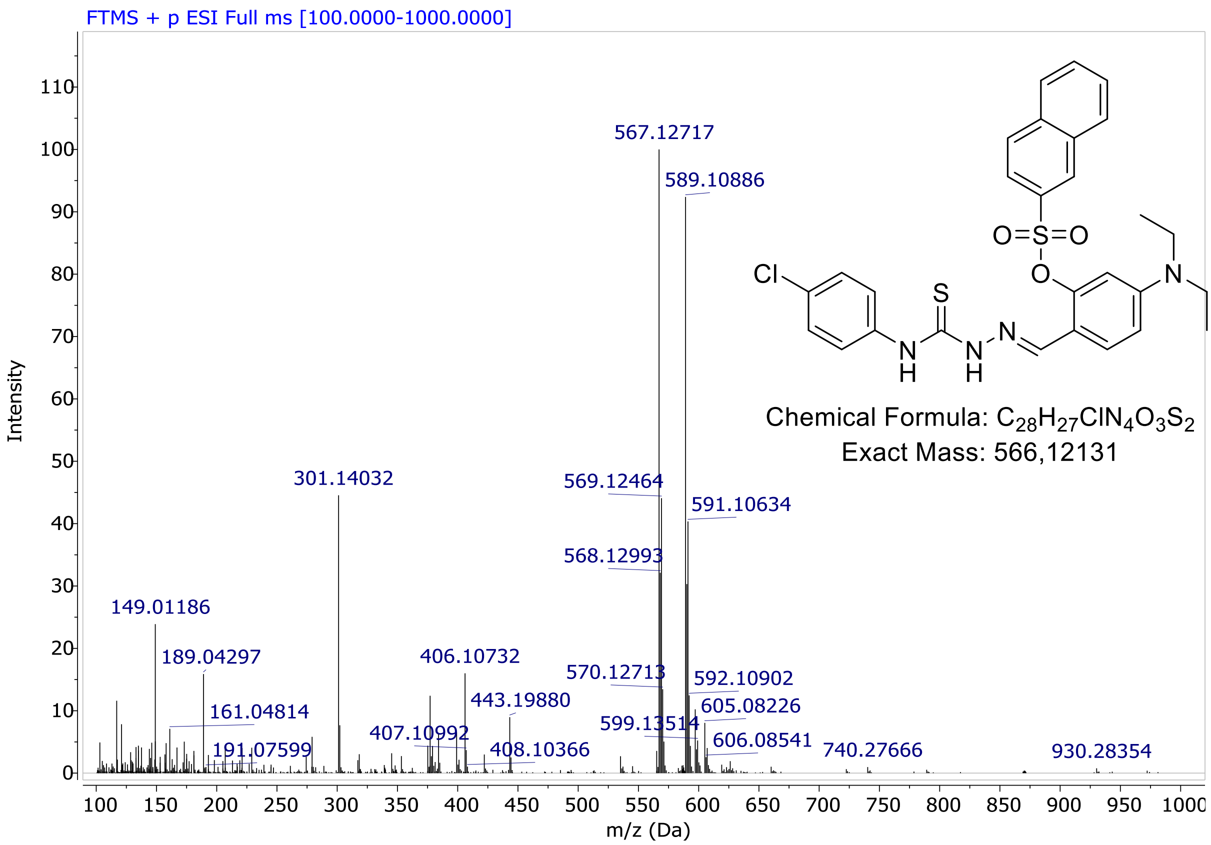

Figure S 47. ESI-HRMS Spectrum of Compound **5i**

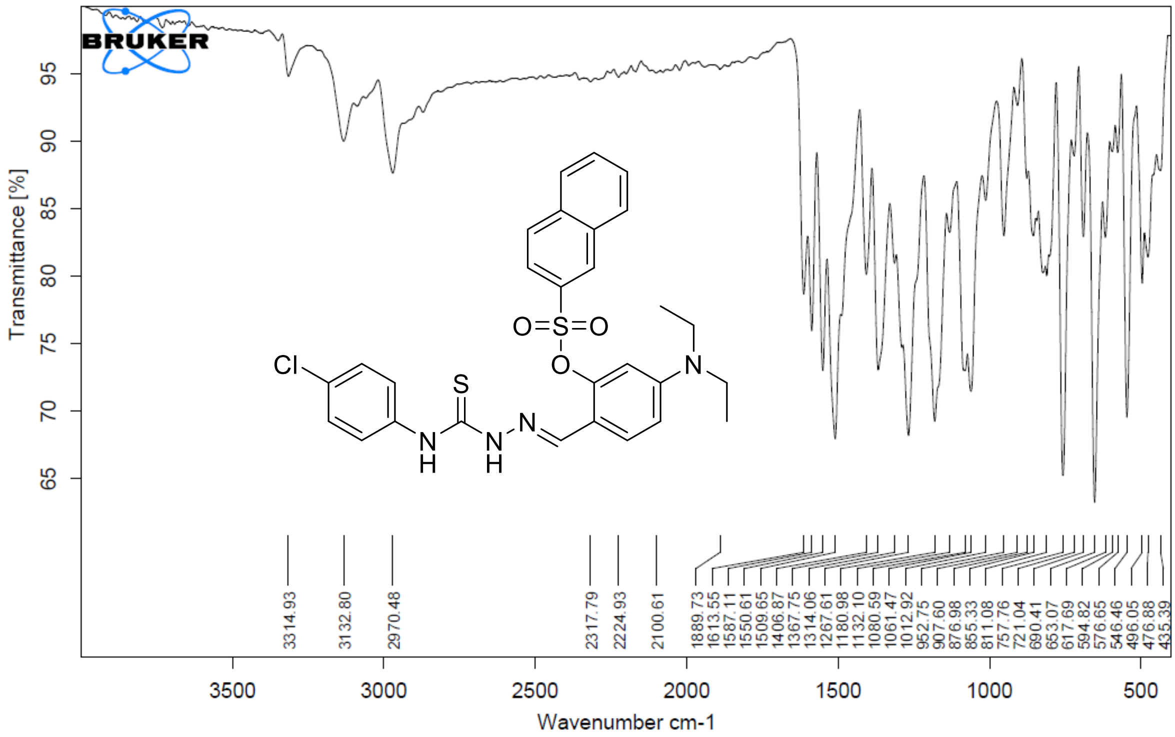

Figure S 48. FT-IR Spectrum of Compound **5i**

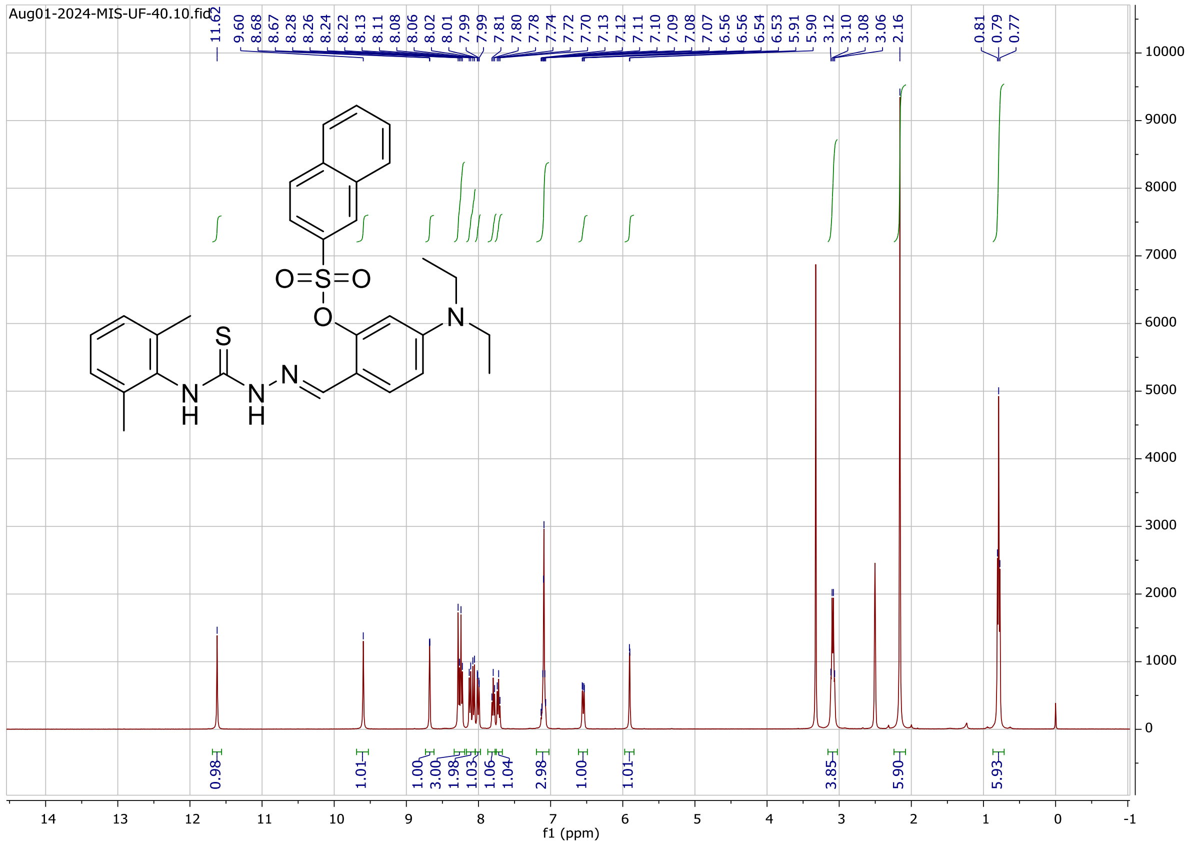

Figure S 49. ^{1}H-NMR Spectrum of Compound **5j** (DMSO-*d6*, 400 MHz)

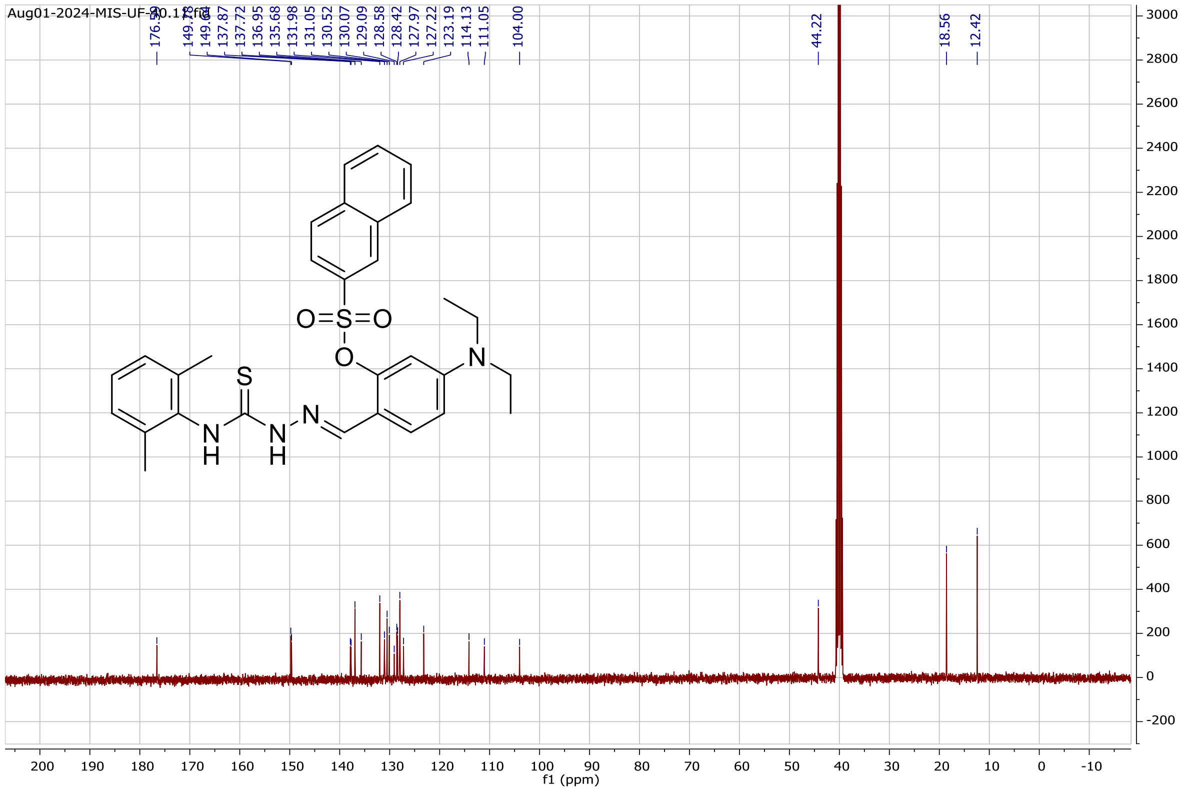

Figure S 50. ^{13}C-NMR Spectrum of Compound **5j** (DMSO-*d6*, 100 MHz)

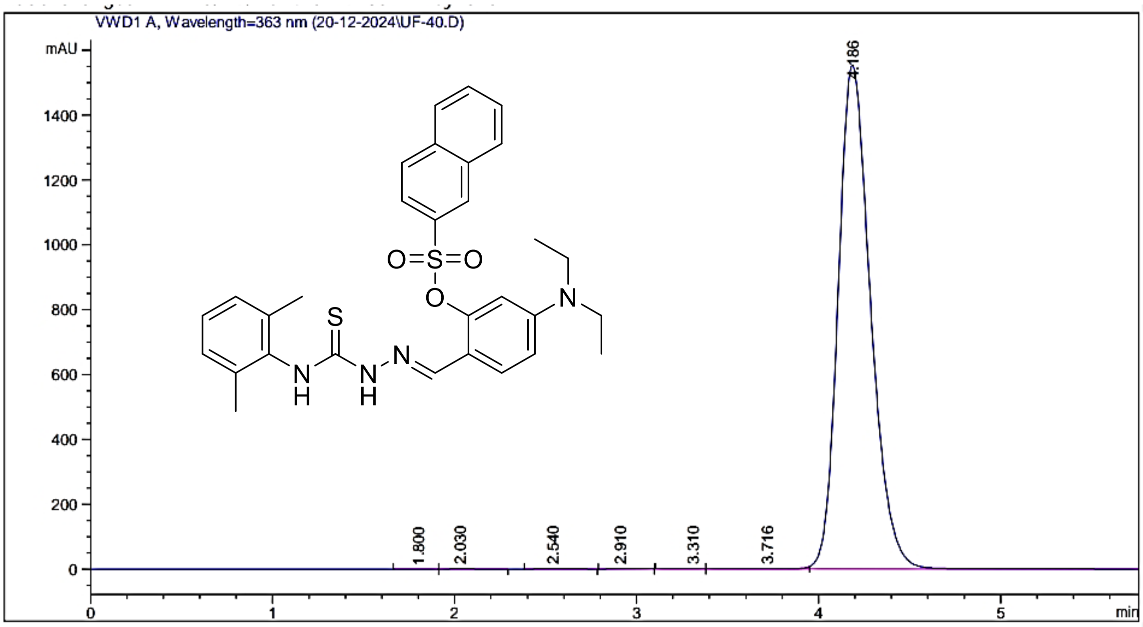

==

Area Percent Report

==

Sorted By : Signal
Multiplier : 1.0000
Dilution : 1.0000
Use Multiplier & Dilution Factor with ISTDs

Signal 1: VWD1 A, Wavelength=363 nm

Peak #	RetTime [min]	Type	Width [min]	Area [mAU*s]	Height [mAU]	Area %
1	1.800	BV	0.1125	1.16770	1.39129e-1	6.068e-3
2	2.030	VB	0.1410	4.38038	4.62744e-1	0.0228
3	2.540	BB	0.1591	16.60466	1.59797	0.0863
4	2.910	BB	0.1277	3.19885	3.84148e-1	0.0166
5	3.310	BV E	0.1210	1.09275	1.15031e-1	5.678e-3
6	3.716	VV E	0.1997	17.21306	1.23011	0.0894
7	4.186	VBAR	0.1893	1.92015e4	1554.07251	99.7732

Totals : 1.92451e4 1558.00163

Figure S 51. HPLC Purity Analysis of Compound **5j**

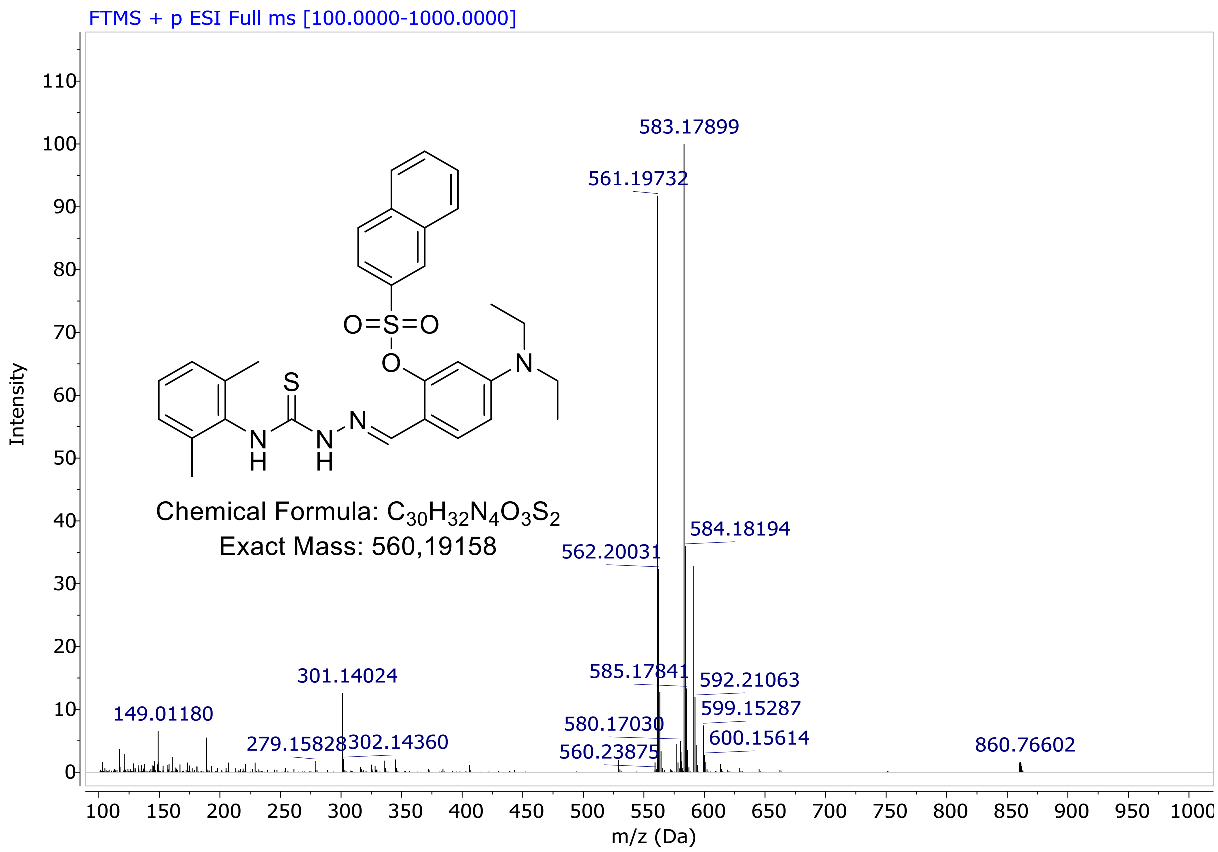

Figure S 52. ESI-HRMS Spectrum of Compound **5j**

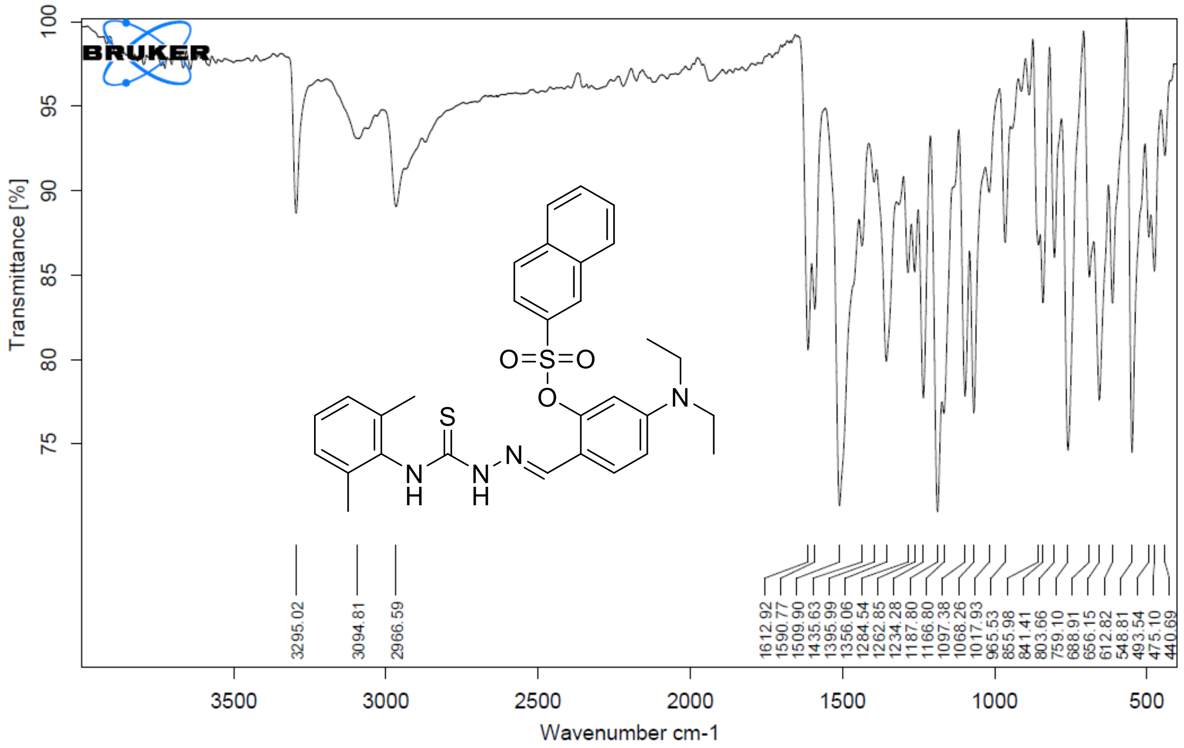

Figure S 53. FT-IR Spectrum of Compound **5j**

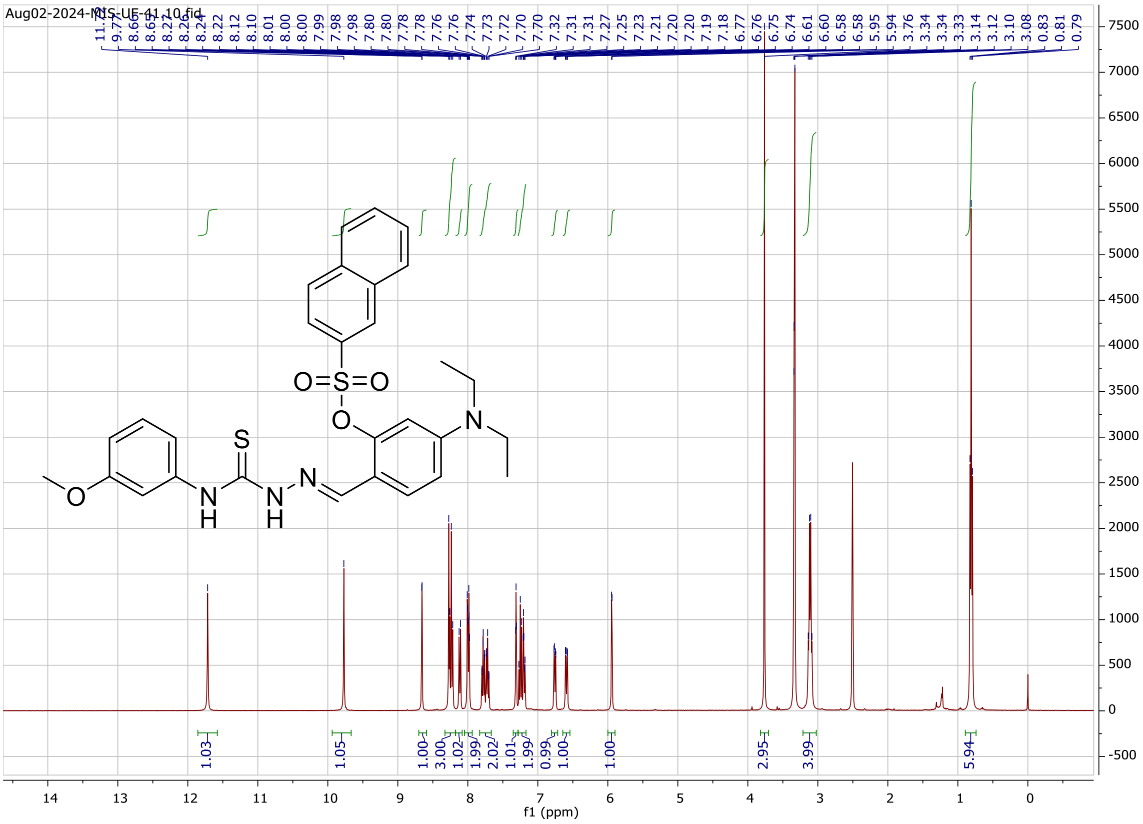

Figure S 54. ^{1}H-NMR Spectrum of Compound **5k** (DMSO-*d6*, 400 MHz)

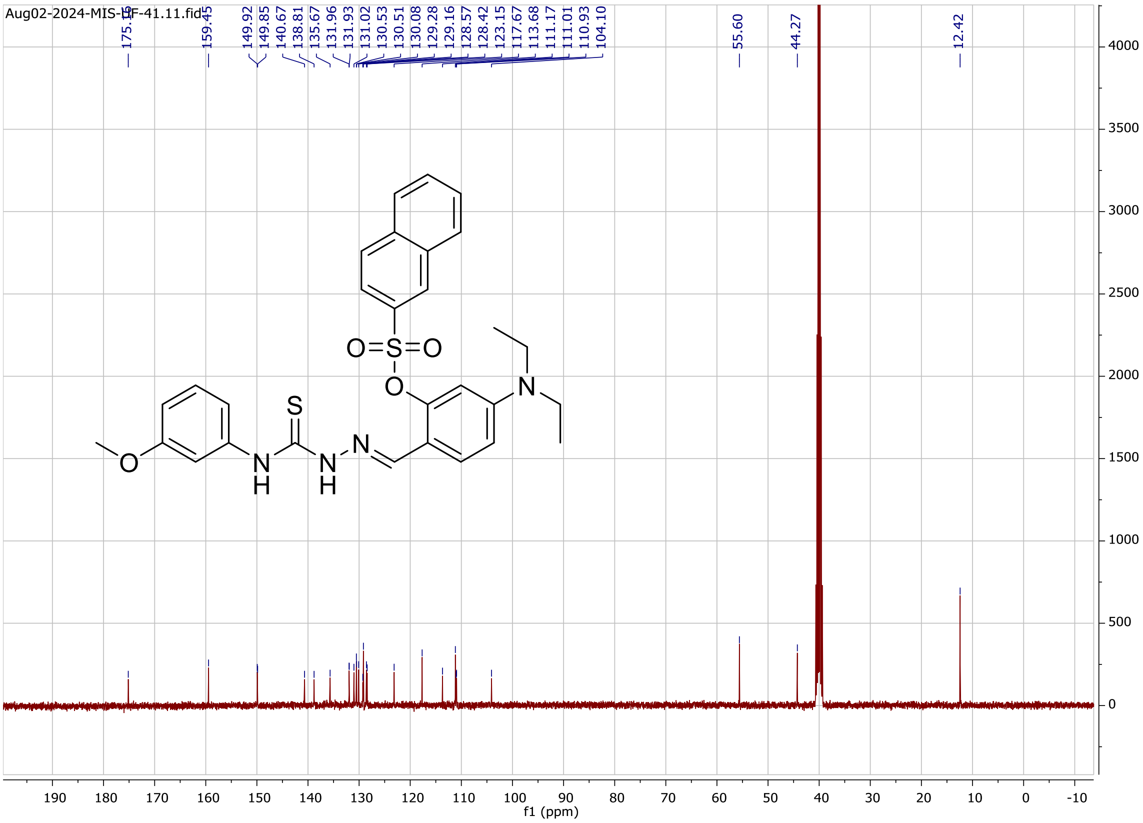

Figure S 55. ^{13}C-NMR Spectrum of Compound **5k** (DMSO-*d6*, 100 MHz)

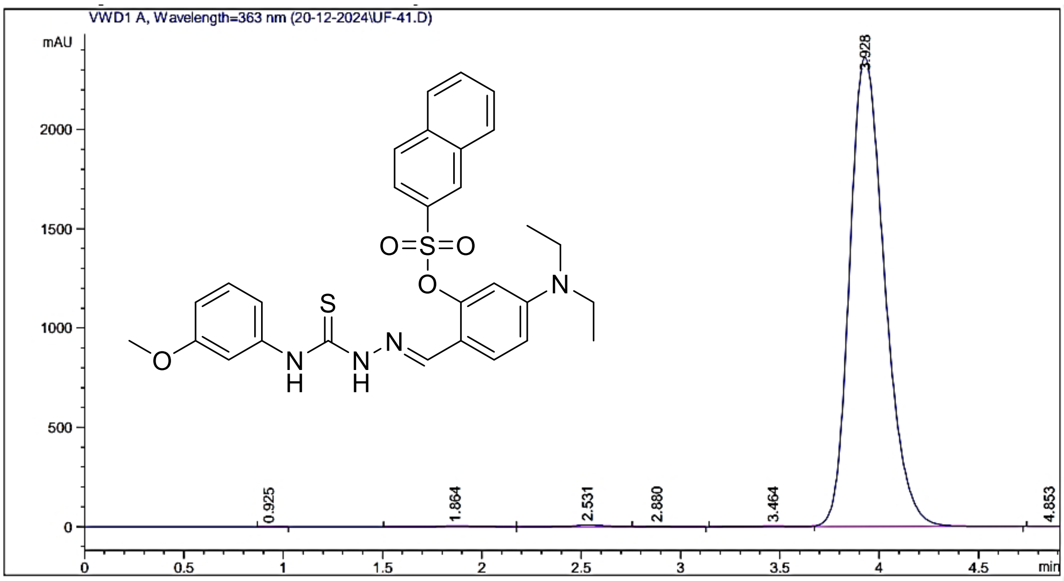


```
=====================================================================
                         Area Percent Report
=====================================================================

Sorted By             :      Signal
Multiplier            :      1.0000
Dilution              :      1.0000
Use Multiplier & Dilution Factor with ISTDs


Signal 1: VWD1 A, Wavelength=363 nm
```

Peak #	RetTime [min]	Type	Width [min]	Area [mAU*s]	Height [mAU]	Area %
1	0.925	BB	0.0761	2.00753e-1	3.55127e-2	6.891e-4
2	1.864	BV	0.1475	39.15018	4.00708	0.1344
3	2.531	VV R	0.1530	85.74770	8.37039	0.2943
4	2.880	VB E	0.1482	3.79124	3.42094e-1	0.0130
5	3.464	BV E	0.1861	36.94952	2.90814	0.1268
6	3.928	VB R	0.1897	2.89643e4	2361.51831	99.4268
7	4.853	BBA	0.0988	1.14209	1.92683e-1	3.920e-3
Totals :				2.91313e4	2377.37420	

Figure S 56. HPLC Purity Analysis of Compound **5k**

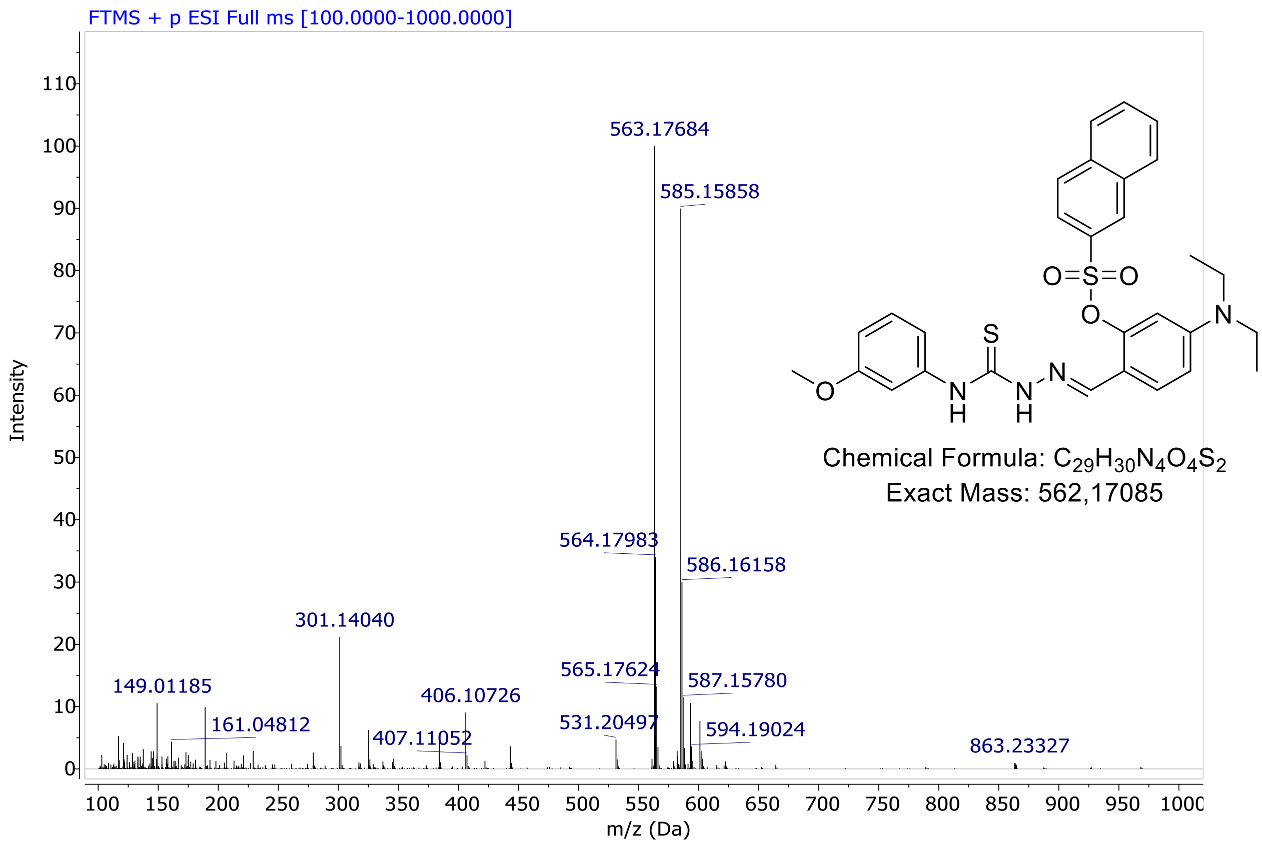

Figure S 57. ESI-HRMS Spectrum of Compound **5k**

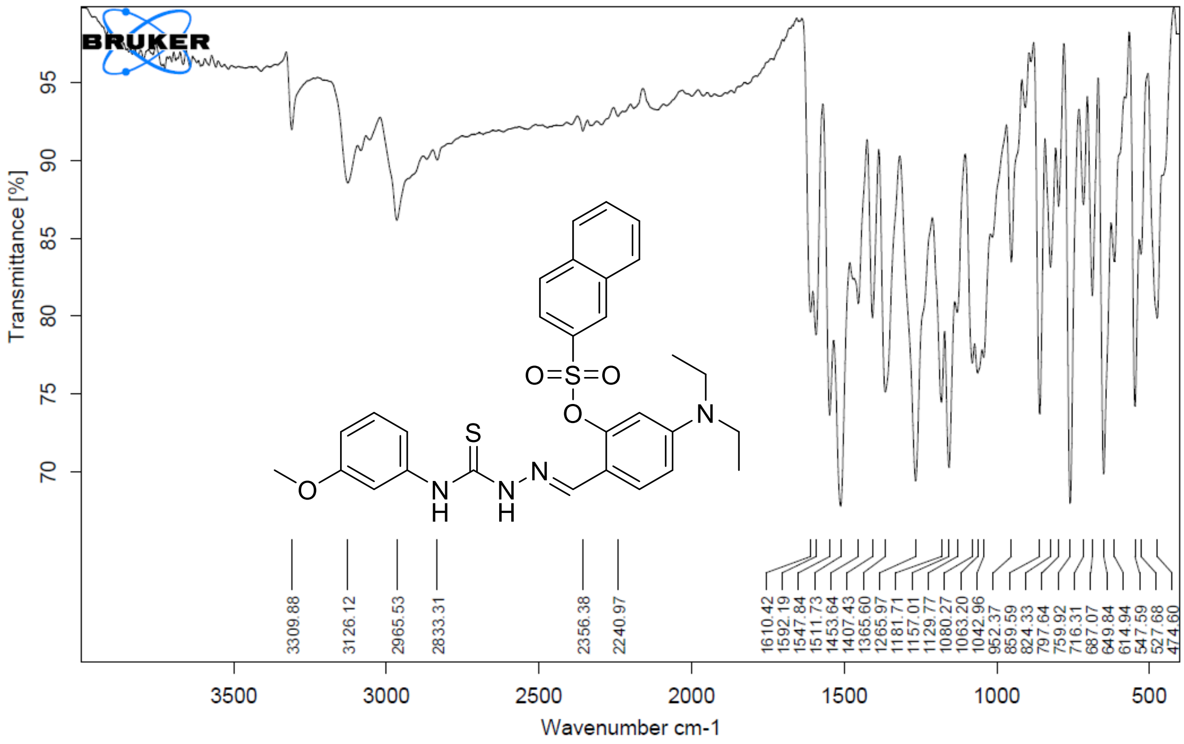

Figure S 58. FT-IR Spectrum of Compound **5k**

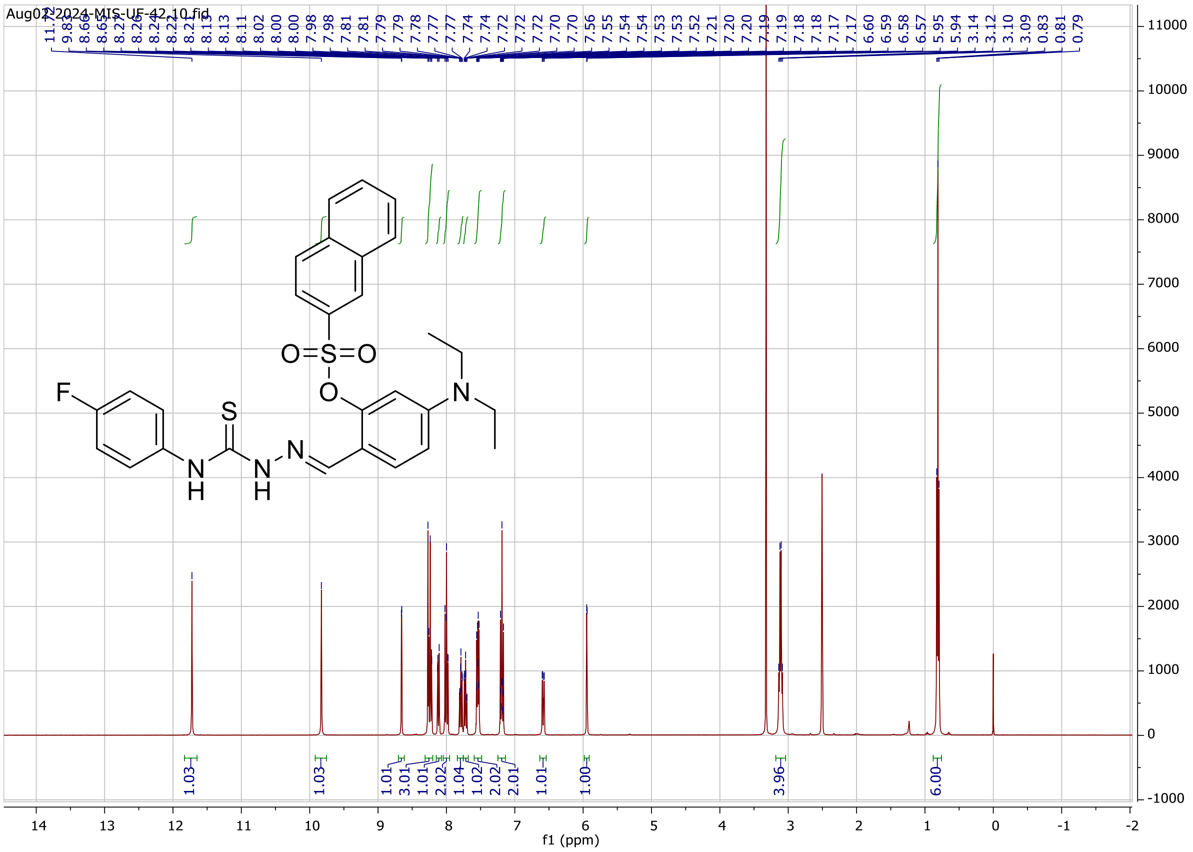

Figure S 59. ^{1}H-NMR Spectrum of Compound **5l** (DMSO-*d6*, 400 MHz)

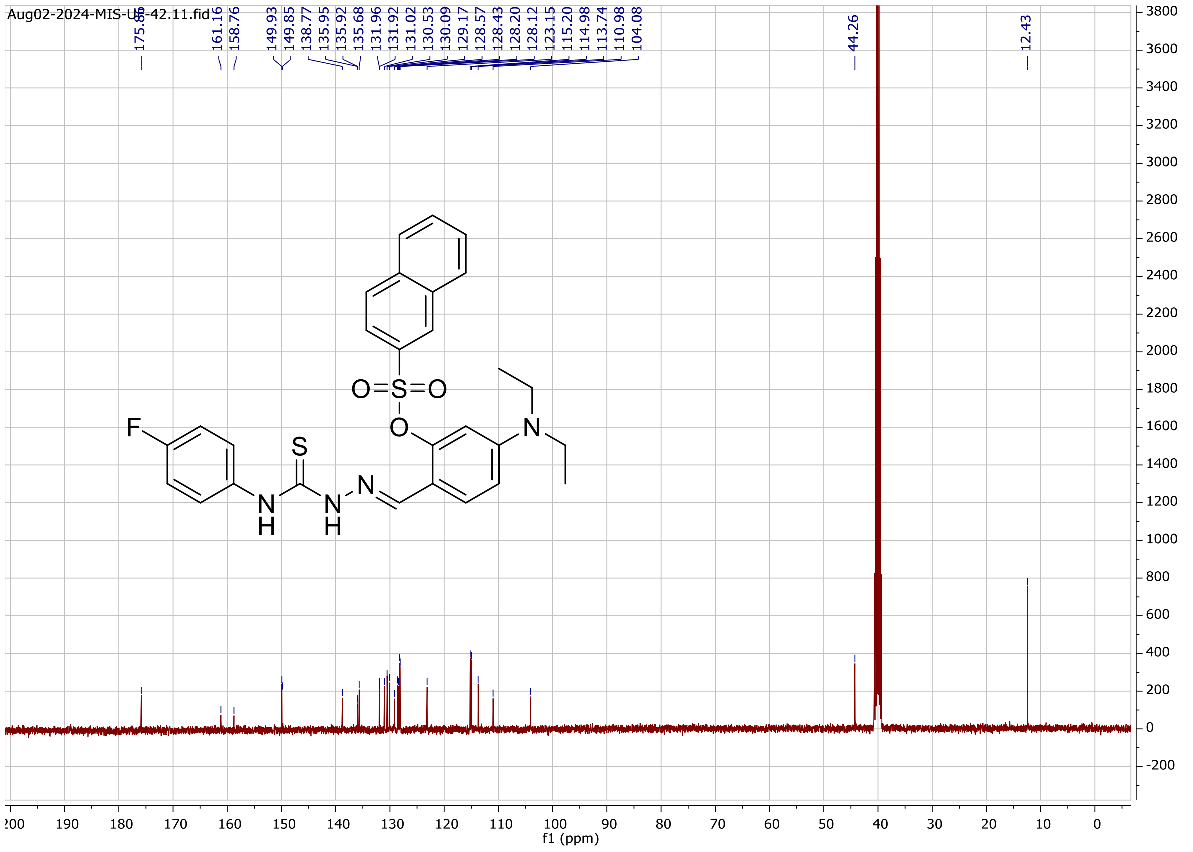

Figure S 60. ^{13}C-NMR Spectrum of Compound **5l** (DMSO-*d6*, 100 MHz)

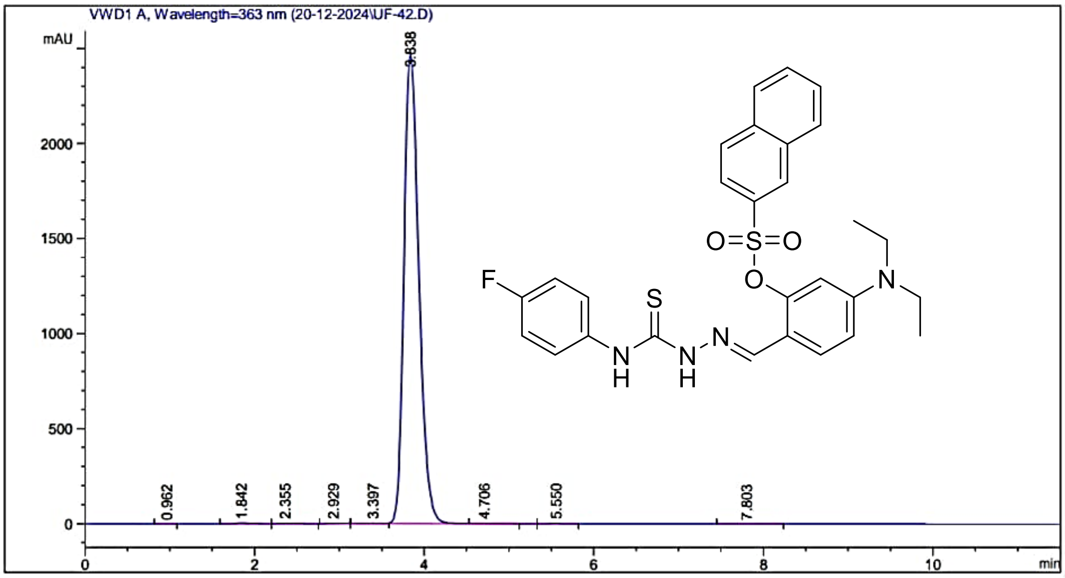

===
Area Percent Report
===

Sorted By : Signal
Multiplier : 1.0000
Dilution : 1.0000
Use Multiplier & Dilution Factor with ISTDs

Signal 1: VWD1 A, Wavelength=363 nm

Peak #	RetTime [min]	Type	Width [min]	Area [mAU*s]	Height [mAU]	Area %
1	0.962	BB	0.1055	4.28311e-1	5.86818e-2	1.412e-3
2	1.842	BV	0.1495	38.42909	3.81664	0.1267
3	2.355	VB	0.2099	16.58397	1.08800	0.0547
4	2.929	BB	0.1492	18.63580	1.97934	0.0614
5	3.397	BV E	0.1740	26.35806	2.30864	0.0869
6	3.838	VV R	0.1894	3.02208e4	2468.54541	99.6192
7	4.706	VB E	0.2160	8.20460	5.20430e-1	0.0270
8	5.550	BB	0.1800	3.07651	2.33347e-1	0.0101
9	7.803	BB	0.2318	3.79024	1.99545e-1	0.0125

HPLC 12/20/2024 4:40:27 PM SYSTEM   Page 1 of 2

Data File D:\HPLC-DATA\Data\20-12-2024\UF-42.D
Sample Name: UF-42

Totals : 3.03363e4 2478.75004

Figure S 61. HPLC Purity Analysis of Compound **5l**

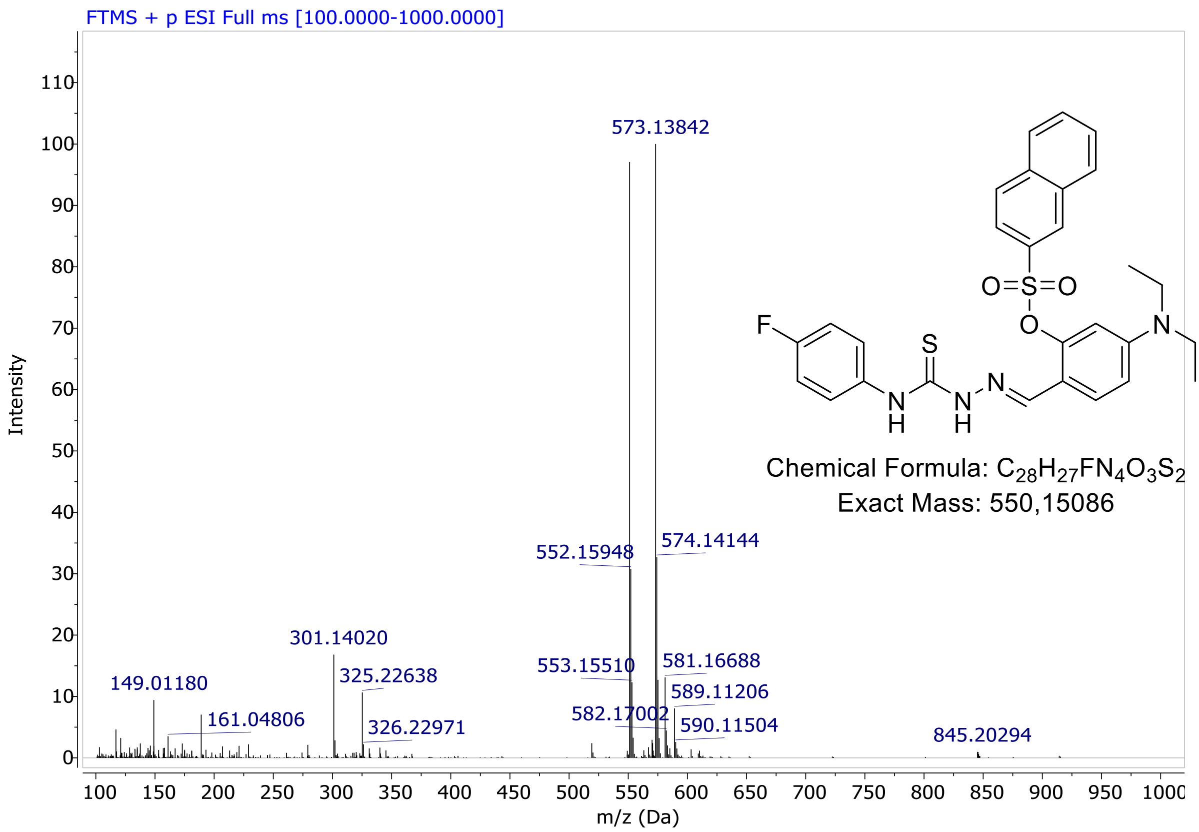

Figure S 62. ESI-HRMS Spectrum of Compound **5l**

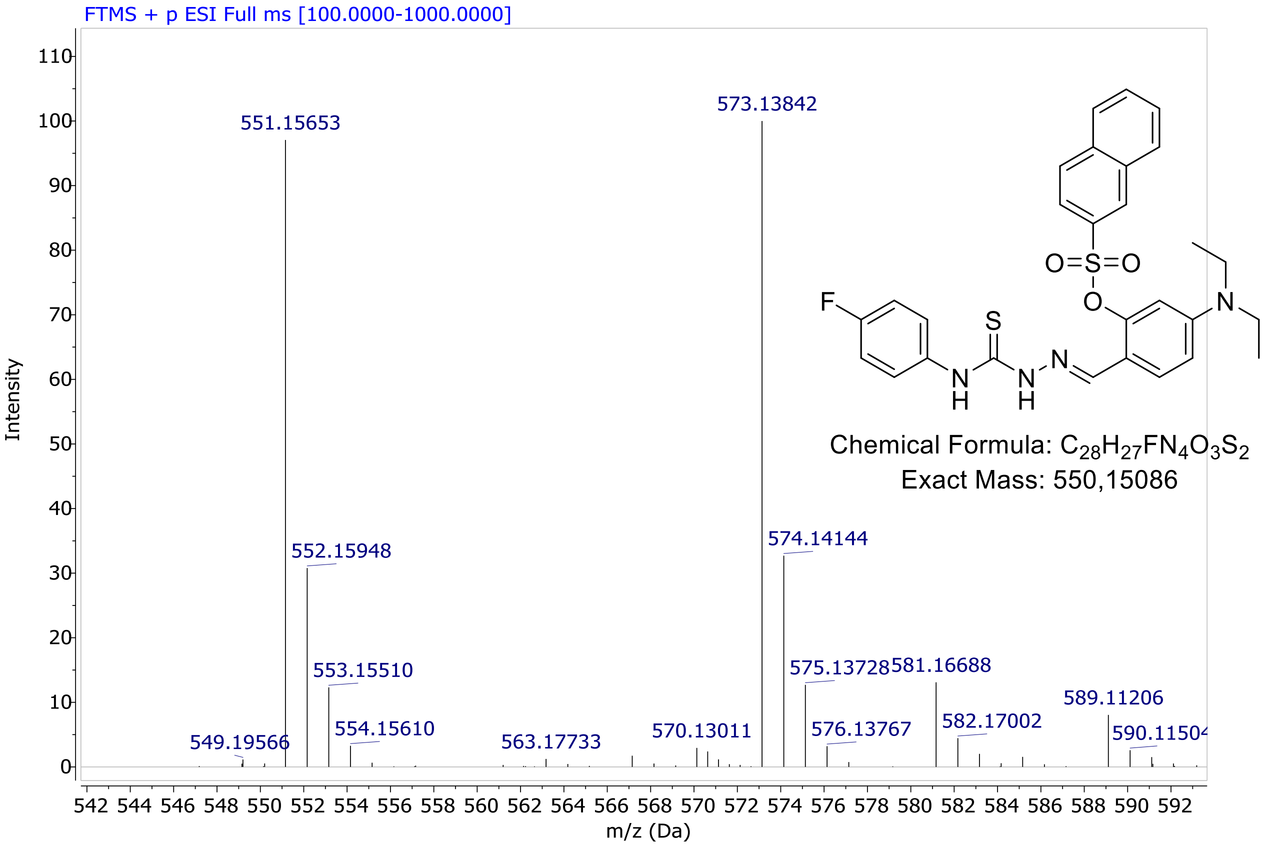

Figure S 63. ESI-HRMS Spectrum of Compound **5l** (extended)

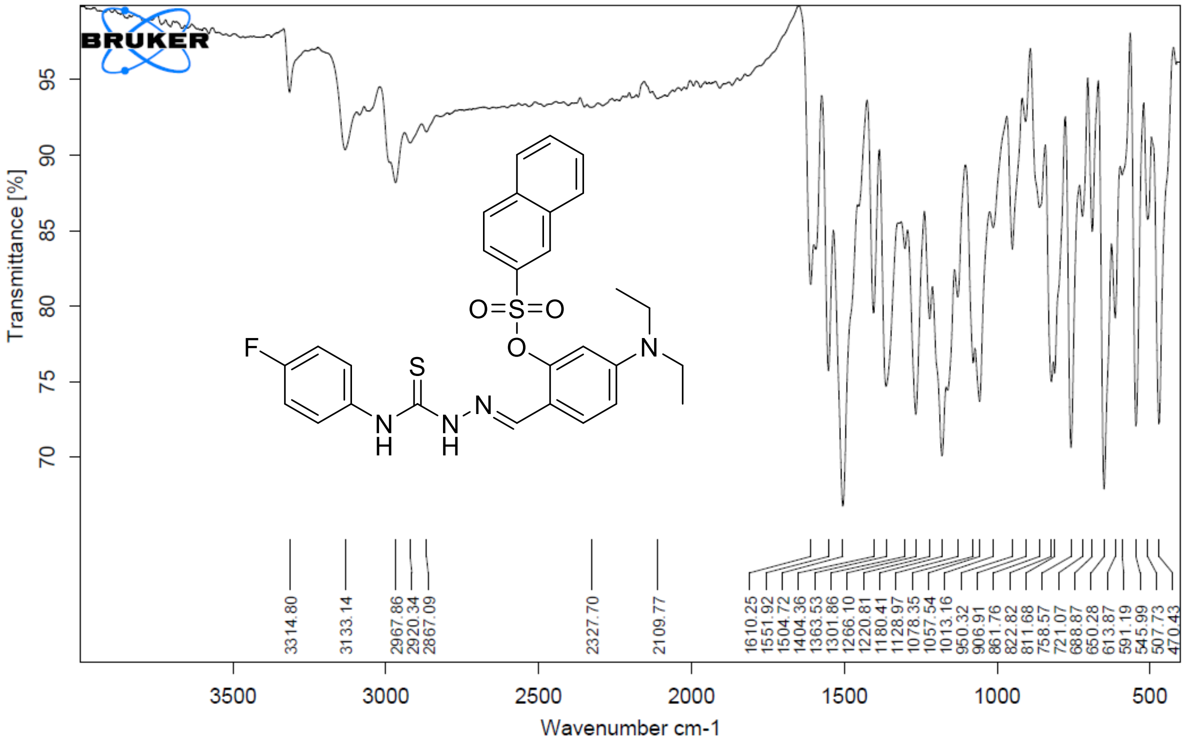

Figure S 64. FT-IR Spectrum of Compound **5l**

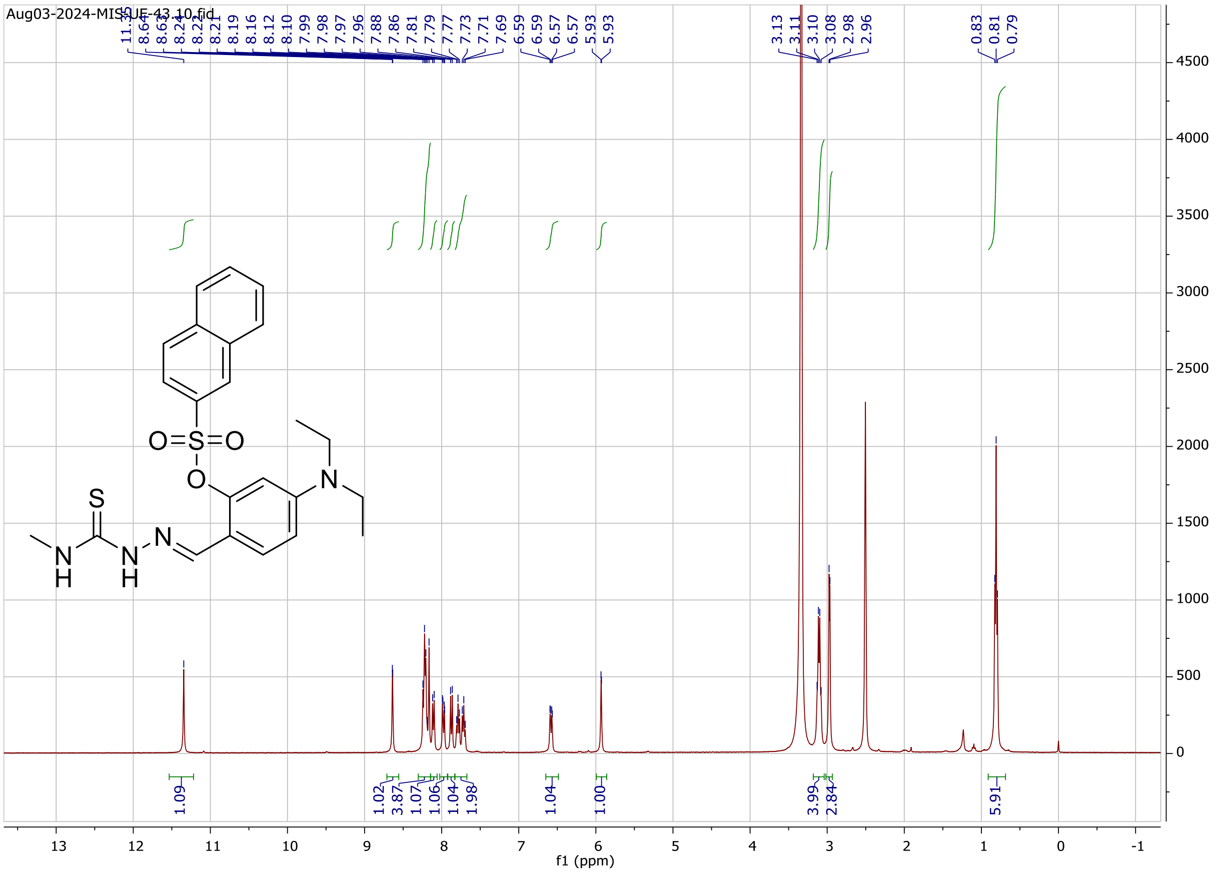

Figure S 65. ^{1}H-NMR Spectrum of Compound **5m** (DMSO-*d6*, 400 MHz)

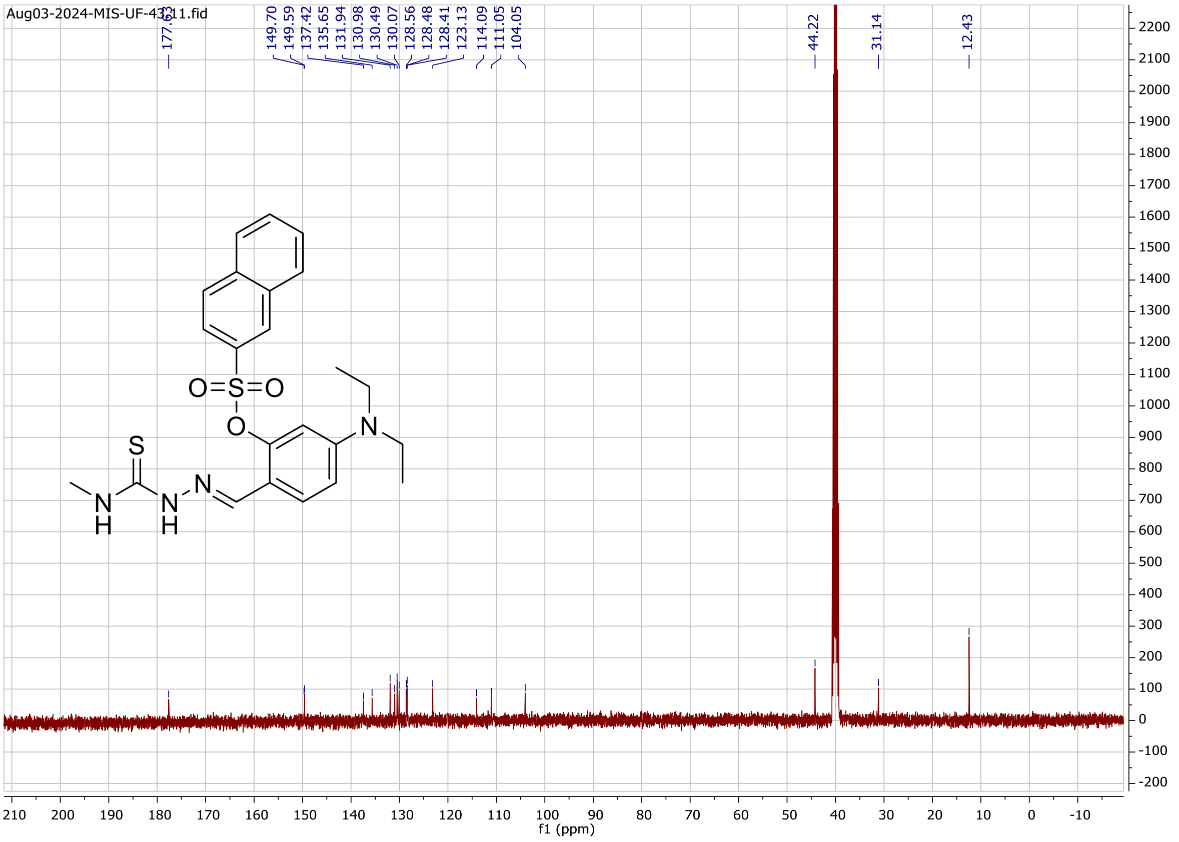

Figure S 66. ^{13}C-NMR Spectrum of Compound **5m** (DMSO-*d6*, 100 MHz)

Last changed : 5/29/2024 5:19:35 PM by SYSTEM
Analysis Method : D:\HPLC-DATA\Method\washing.M
Last changed : 12/2/2024 10:20:36 AM by SYSTEM

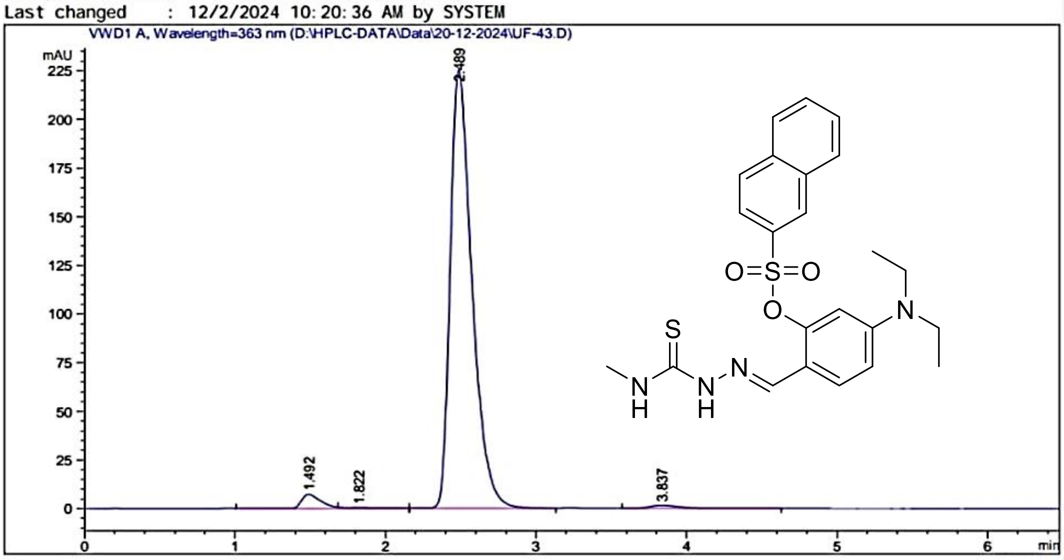

Area Percent Report

Sorted By : Signal
Calib. Data Modified : Saturday, October 29, 2022 12:16:02 PM
Multiplier : 1.0000
Dilution : 1.0000
Use Multiplier & Dilution Factor with ISTDs

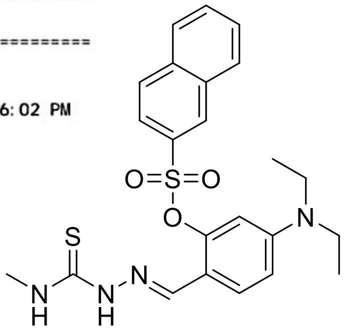

Signal 1: VWD1 A, Wavelength=363 nm

Peak #	RetTime [min]	Type	Width [min]	Area [mAU*s]	Area %	Name
1	1.492	BV R	0.1412	65.05709	2.7610	?
2	1.822	VV E	0.2375	6.45450	0.2739	?
3	2.489	VB	0.1551	2262.73022	96.0311	?
4	3.837	BB	0.2170	22.00531	0.9339	?

Totals : 2356.24713

HPLC 12/21/2024 2:05:55 PM SYSTEM Page 1 of 2

Data File D:\HPLC-DATA\Data\20-12-2024\UF-43.D
Sample Name: UF-43

Signal 2: VWD1 A, Wavelength=254 nm not found

Peak #	RetTime [min]	Type	Width [min]	Area [mAU*s]	Area %	Name
1	1.977		0.0000	0.00000	0.0000	Thiophenate

Totals : 0.00000

Figure S 67. HPLC Purity Analysis of Compound **5m**

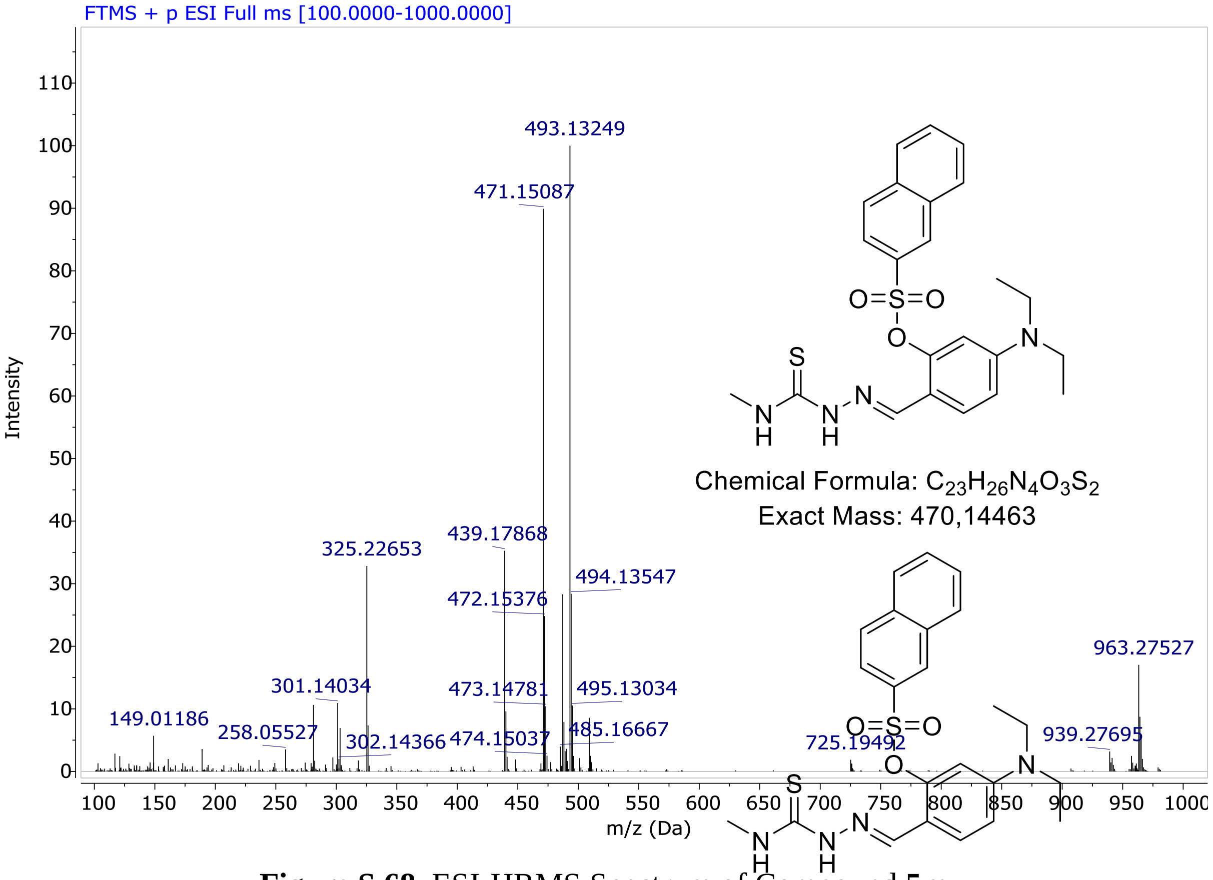

Figure S 68. ESI-HRMS Spectrum of Compound **5m**

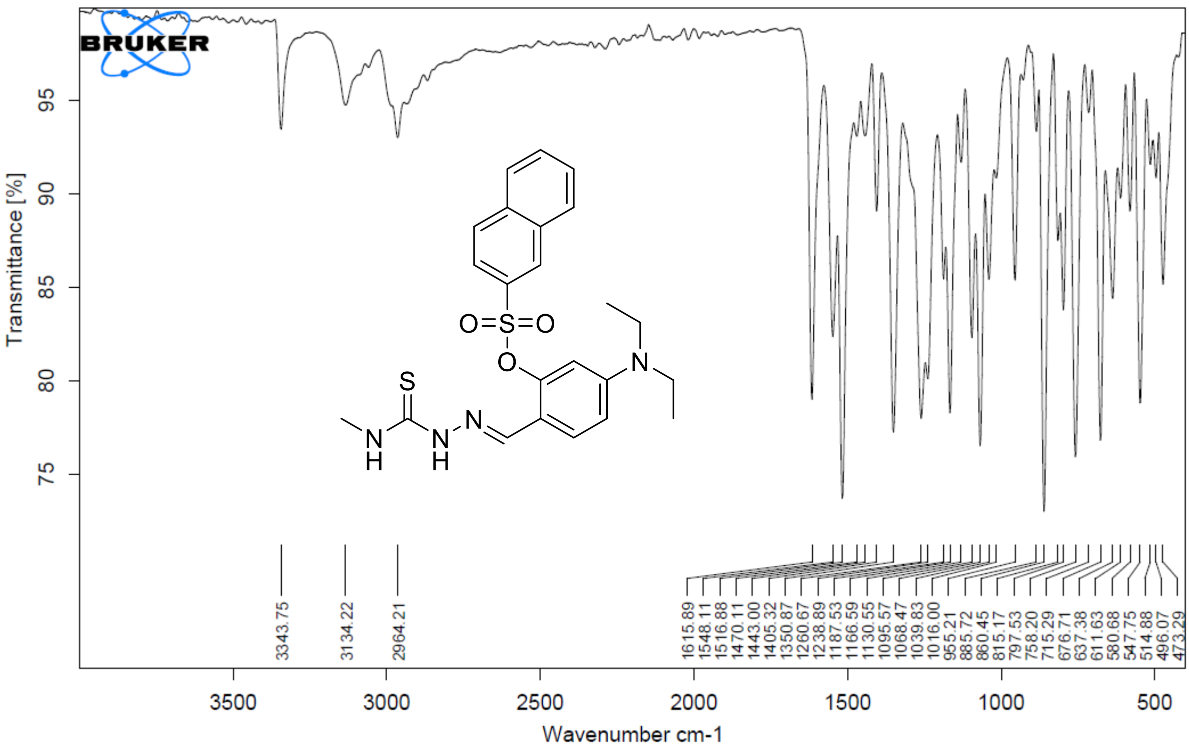

Figure S 69. FT-IR Spectrum of Compound **5m**

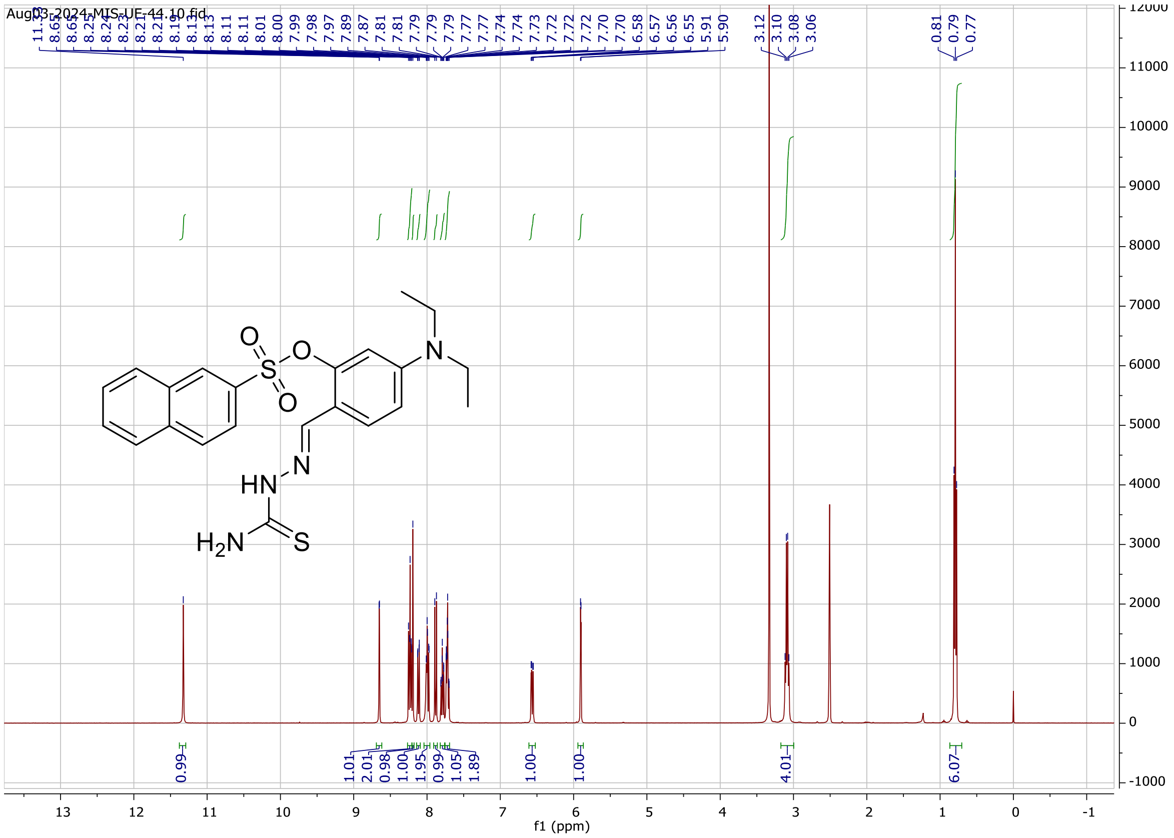

Figure S 70. ^{1}H-NMR Spectrum of Compound **5n** (DMSO-*d6*, 400 MHz)

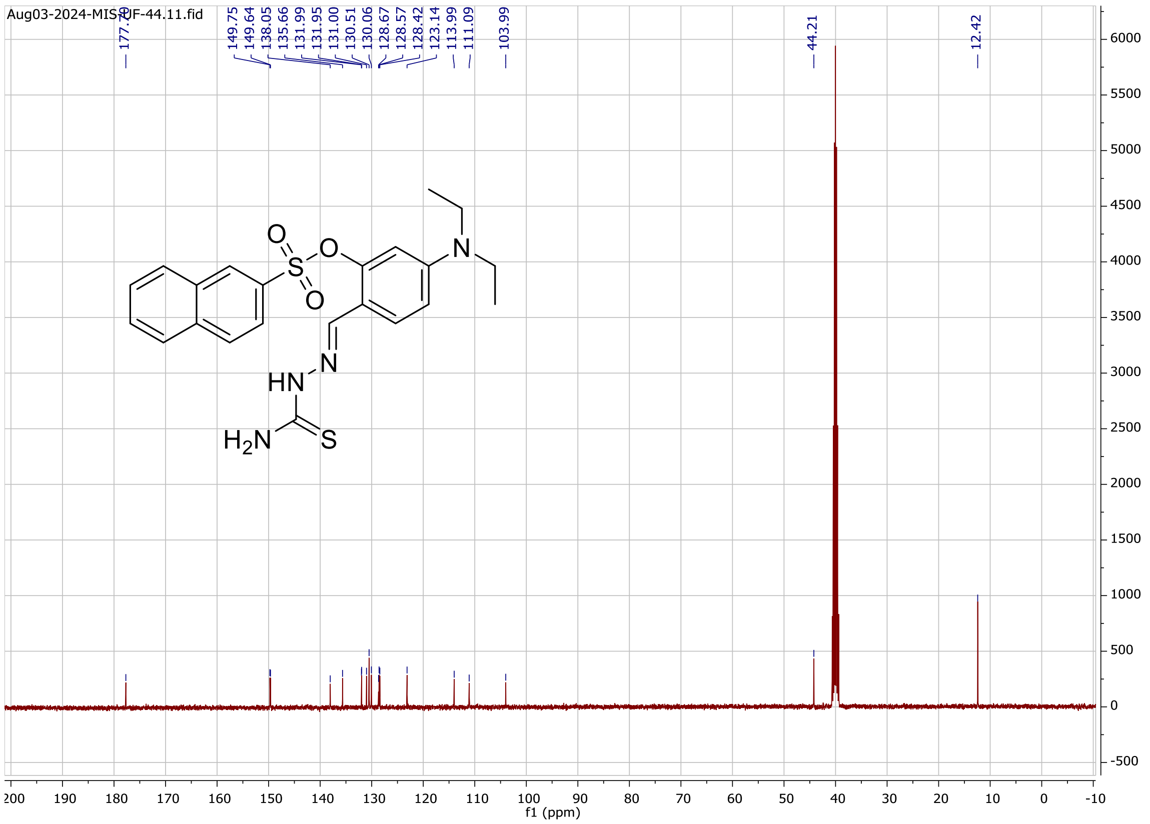

Figure S 71. ^{13}C-NMR Spectrum of Compound **5n** (DMSO-*d6*, 100 MHz)

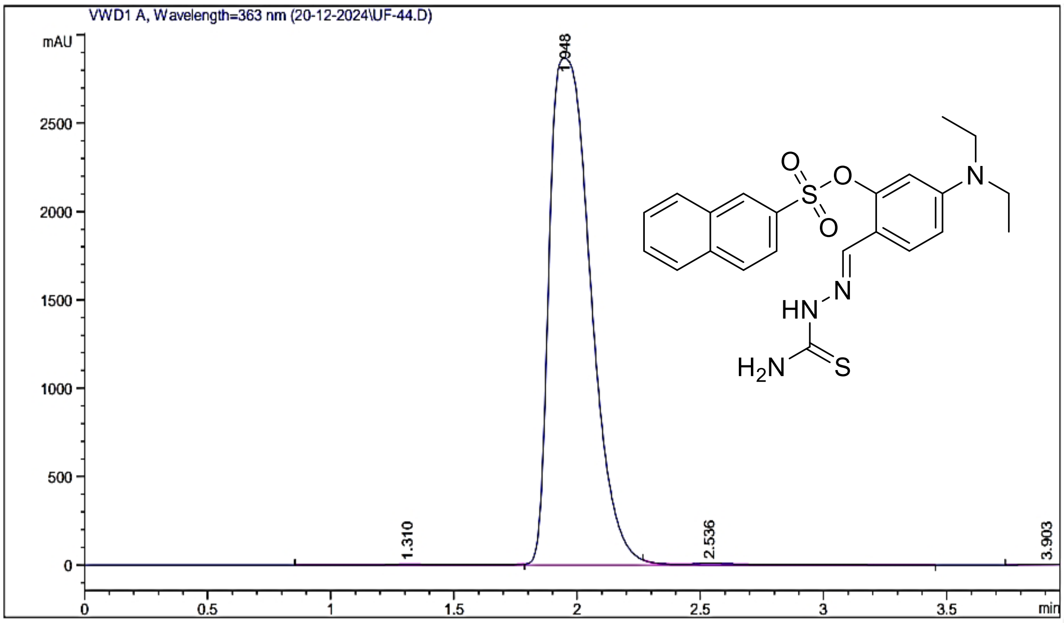

===

Area Percent Report

===

Sorted By : Signal
Multiplier : 1.0000
Dilution : 1.0000
Use Multiplier & Dilution Factor with ISTDs

Signal 1: VWD1 A, Wavelength=363 nm

Peak #	RetTime [min]	Type	Width [min]	Area [mAU*s]	Height [mAU]	Area %
1	1.310	BV E	0.1984	61.04853	4.31784	0.1819
2	1.948	VV R	0.1838	3.33529e4	2866.76587	99.3883
3	2.536	VB E	0.2055	142.70123	9.84370	0.4252
4	3.903	BBA	0.1201	1.53341	2.03024e-1	4.569e-3

Totals : 3.35581e4 2881.13044

Figure S 72. HPLC Purity Analysis of Compound **5n**

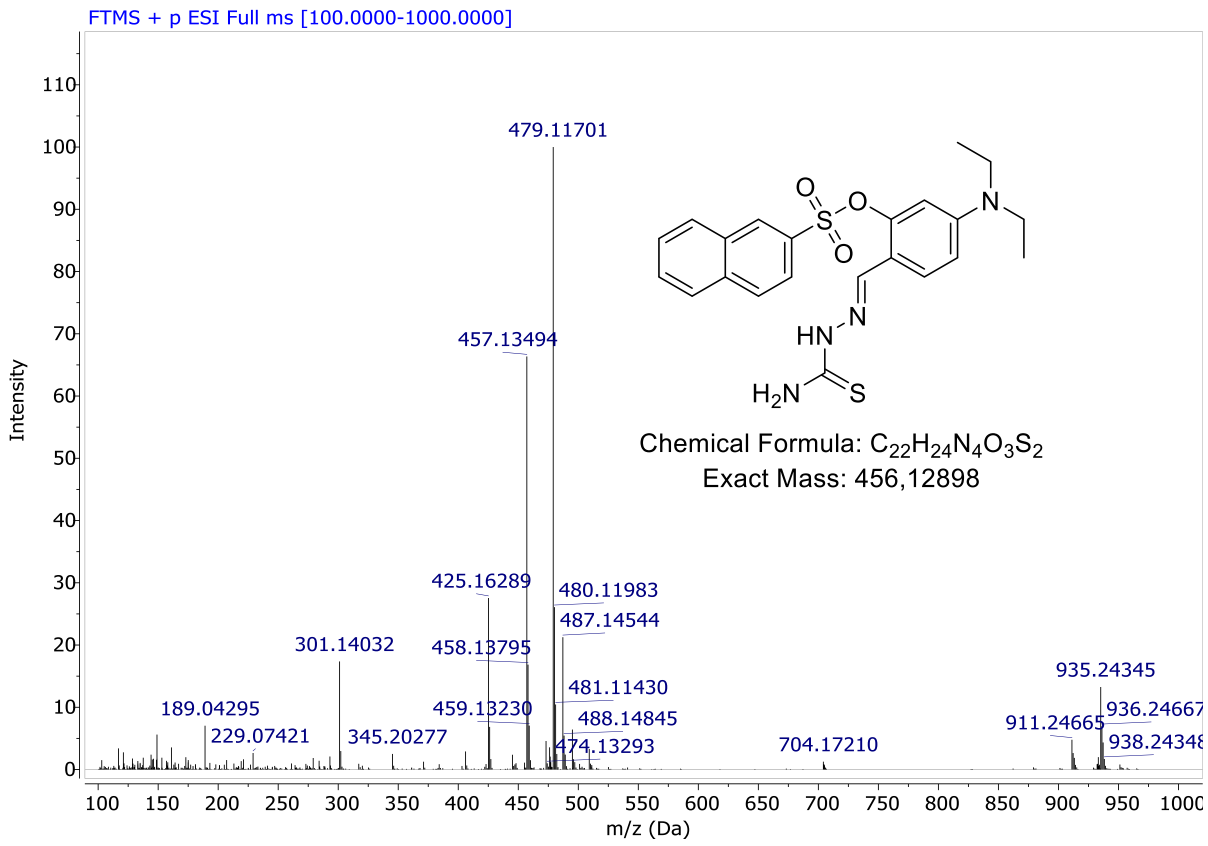

Figure S 73. ESI-HRMS Spectrum of Compound **5n**

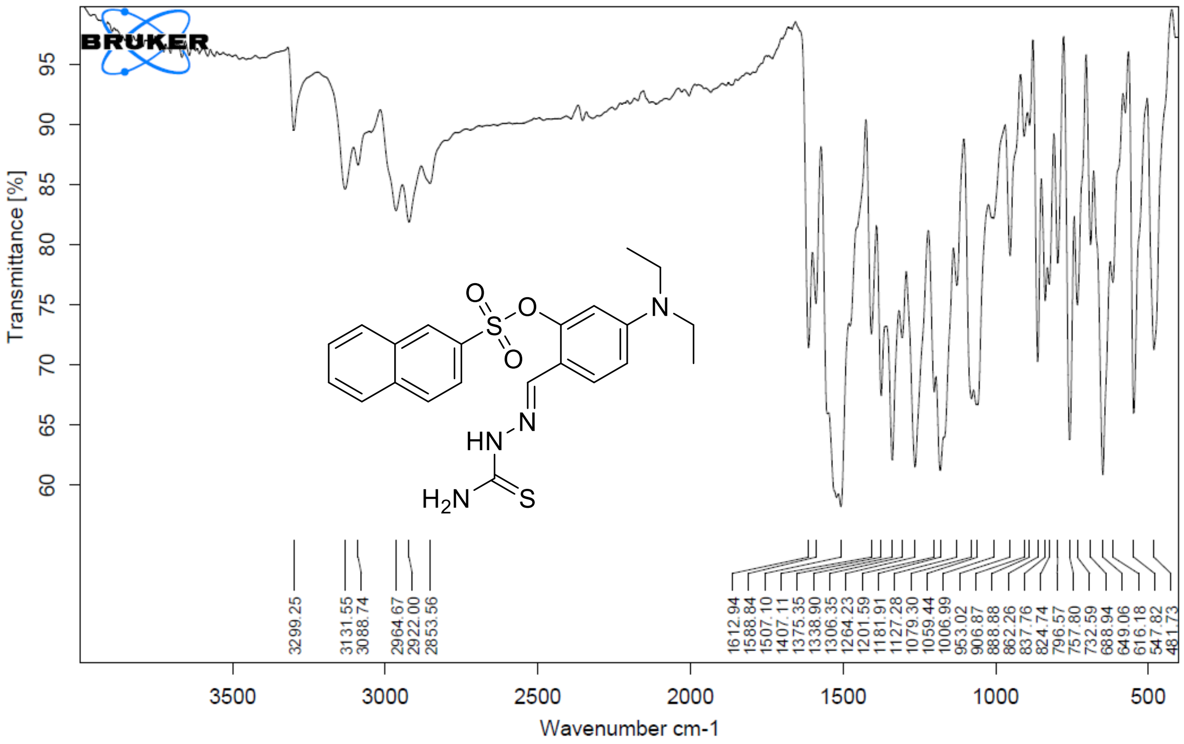

Figure S 74. FT-IR Spectrum of Compound **5n**

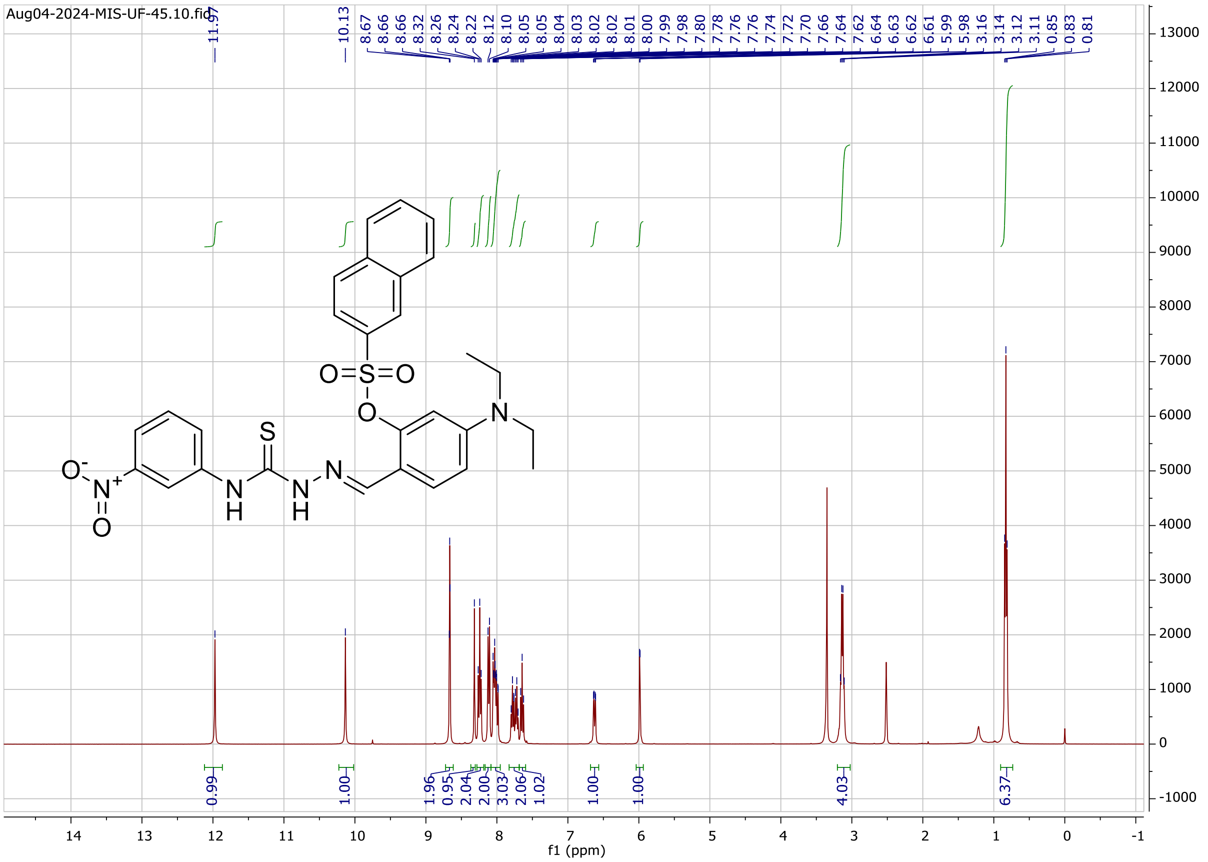

Figure S 75. ^{1}H-NMR Spectrum of Compound **5o** (DMSO-*d6*, 400 MHz)

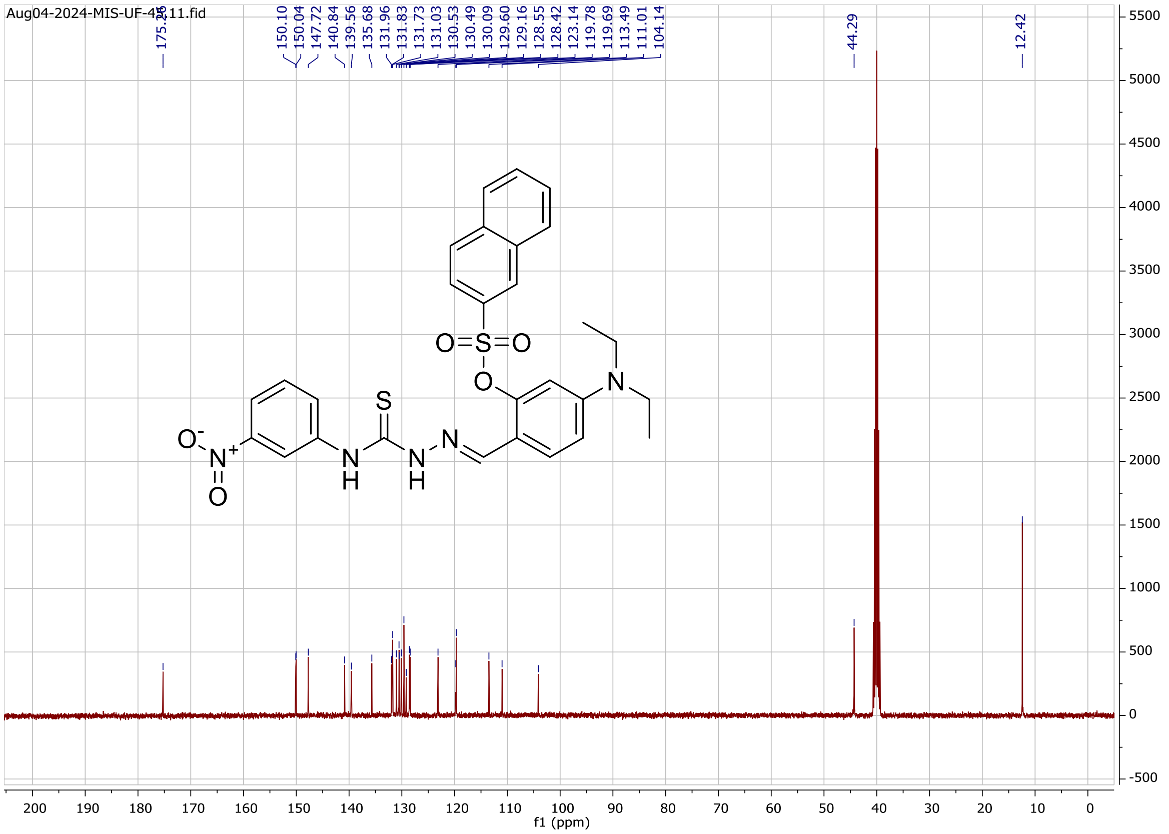

Figure S 76. ^{13}C-NMR Spectrum of Compound **5o** (DMSO-*d6*, 100 MHz)

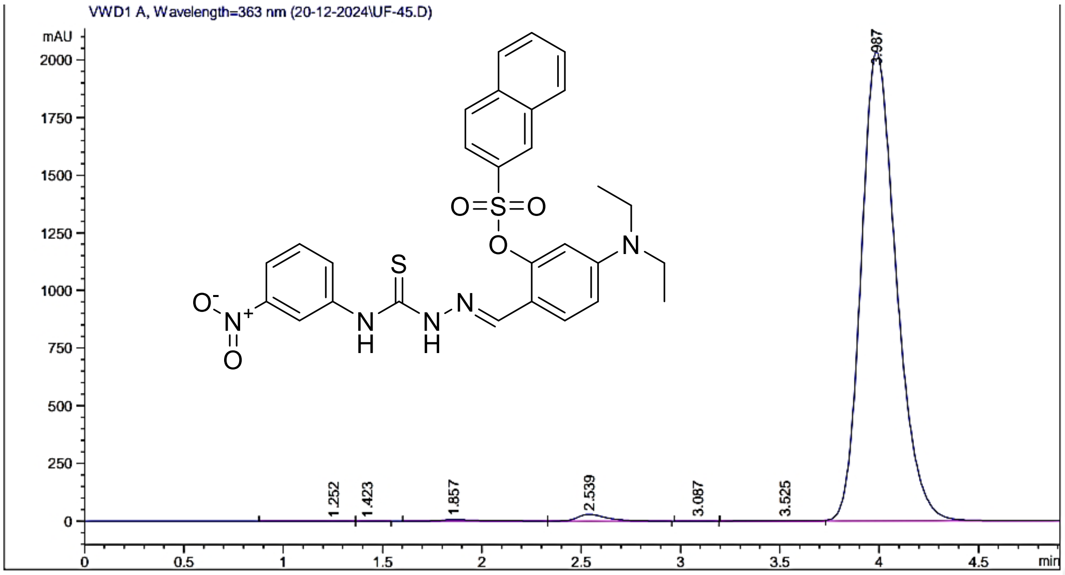


```
==================================================================
                        Area Percent Report
==================================================================

Sorted By             :      Signal
Multiplier            :      1.0000
Dilution              :      1.0000
Use Multiplier & Dilution Factor with ISTDs


Signal 1: VWD1 A, Wavelength=363 nm
```

Peak #	RetTime [min]	Type	Width [min]	Area [mAU*s]	Height [mAU]	Area %
1	1.252	BV	0.1621	1.41147	1.10548e-1	5.570e-3
2	1.423	VB	0.0894	6.38199e-1	9.57481e-2	2.519e-3
3	1.857	BV	0.1707	62.80178	5.34108	0.2478
4	2.539	VB	0.1496	280.99582	28.60305	1.1089
5	3.087	BB	0.1297	9.45516e-1	1.20155e-1	3.731e-3
6	3.525	BV E	0.1894	24.16012	1.89643	0.0953
7	3.987	VBAR	0.1869	2.49691e4	2034.59998	98.5361

Totals : 2.53400e4 2070.76698

Figure S 77. HPLC Purity Analysis of Compound **5o**

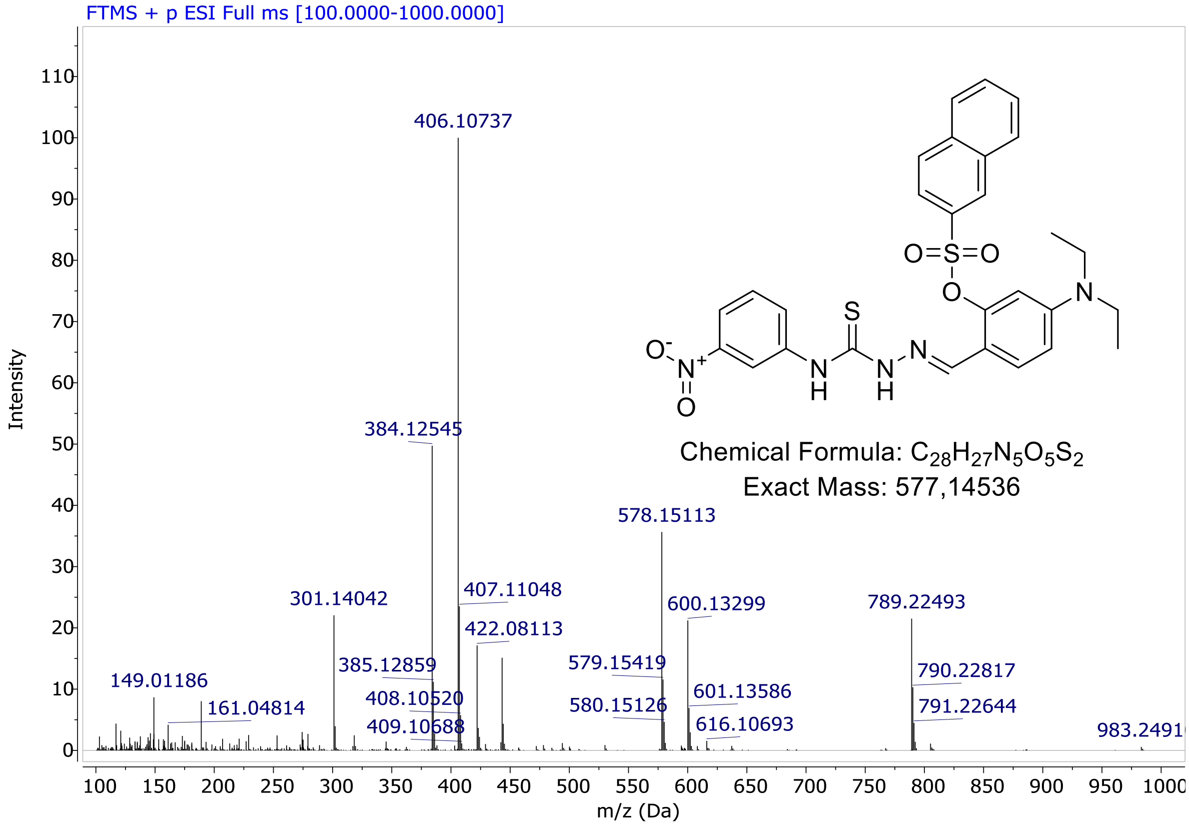

Figure S 78. ESI-HRMS Spectrum of Compound **5o**

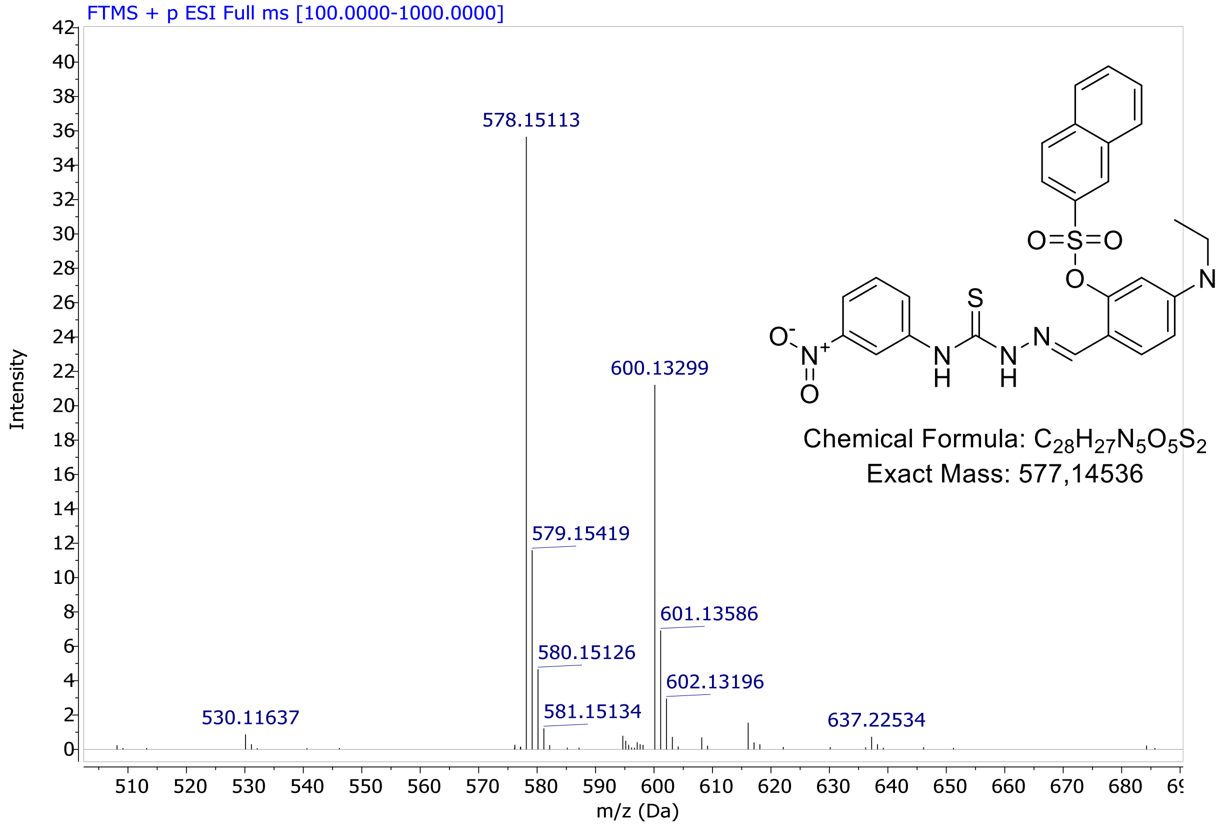

Figure S 79. ESI-HRMS Spectrum of Compound **5o** (extended)

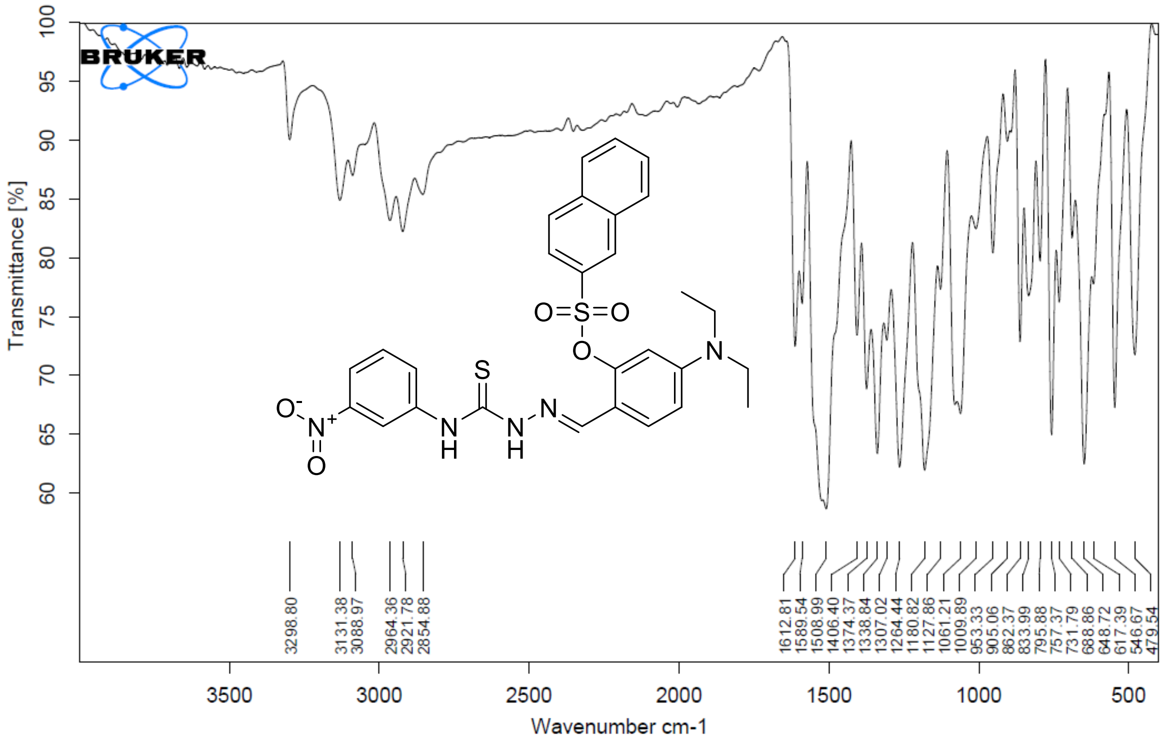

Figure S 80. FT-IR Spectrum of Compound **5o**

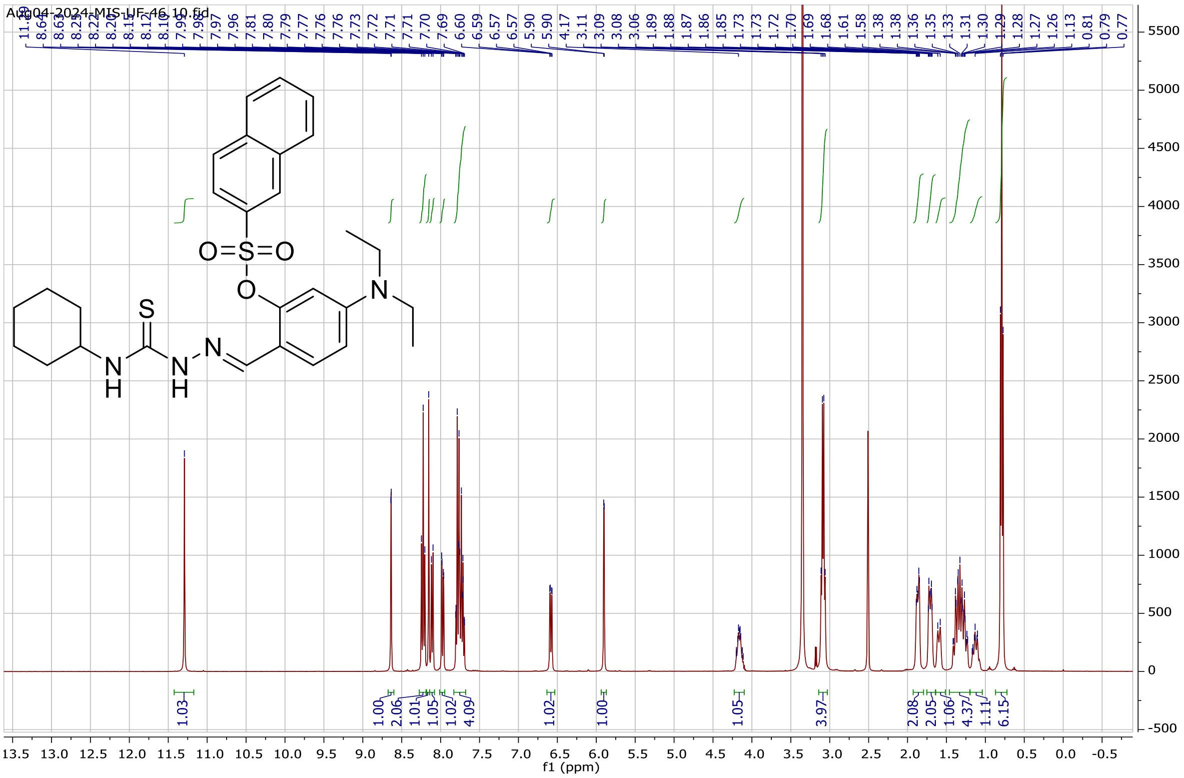

Figure S 81. ^{1}H-NMR Spectrum of Compound **5p** (DMSO-*d6*, 400 MHz)

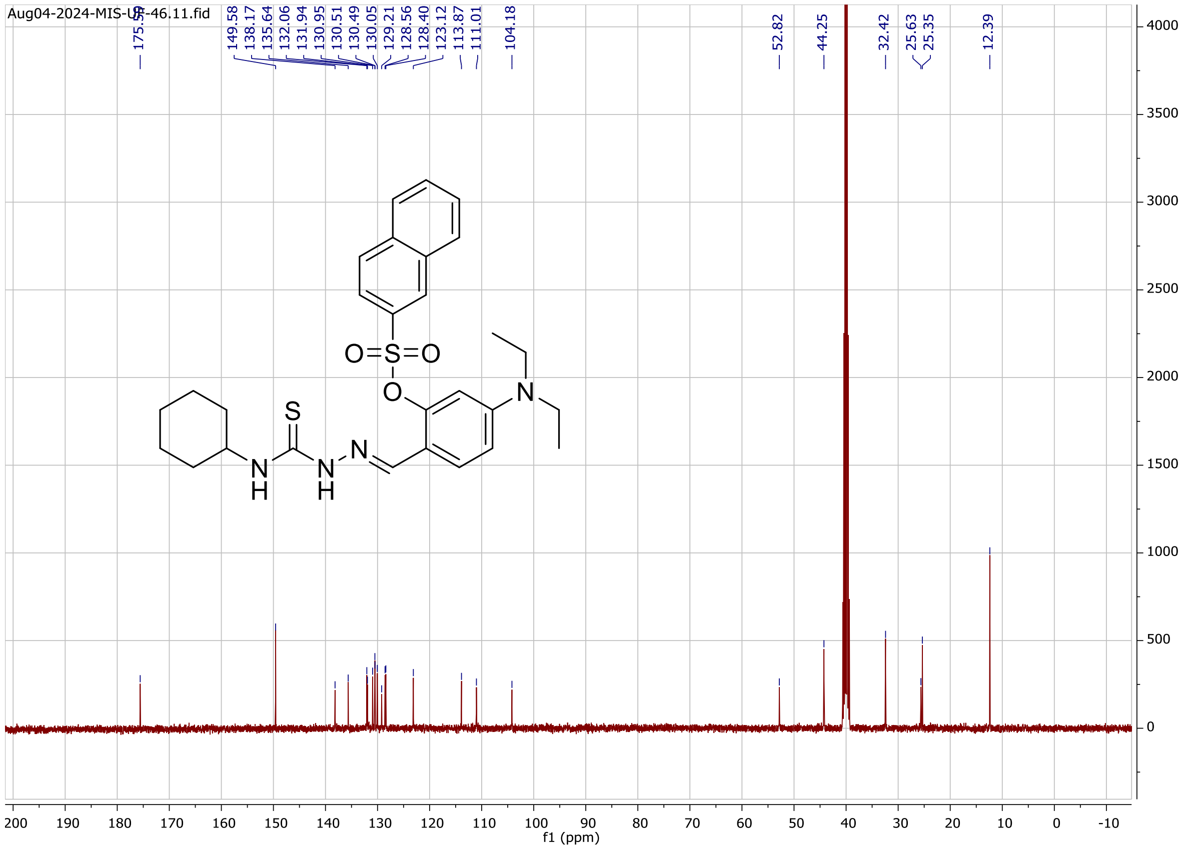

Figure S 82. ^{13}C-NMR Spectrum of Compound **5p** (DMSO-*d6*, 100 MHz)

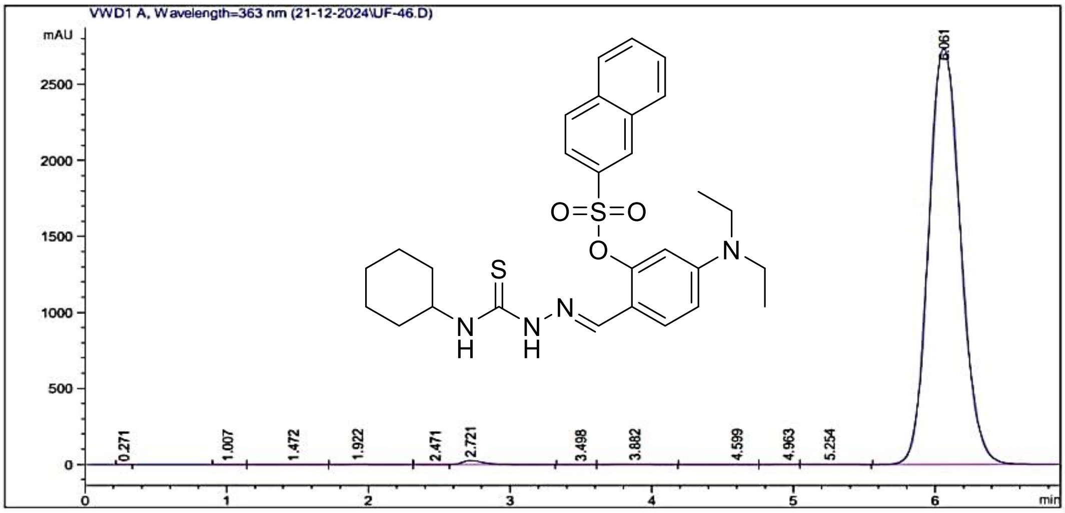


```
=====================================================================
                         Area Percent Report
=====================================================================

Sorted By             :      Signal
Multiplier            :      1.0000
Dilution              :      1.0000
Use Multiplier & Dilution Factor with ISTDs


Signal 1: VWD1 A, Wavelength=363 nm
```

Peak #	RetTime [min]	Type	Width [min]	Area [mAU*s]	Height [mAU]	Area %
1	0.271	BB	0.0469	9.01729e-2	2.86881e-2	2.084e-4
2	1.007	BV	0.1383	7.96371e-1	7.16990e-2	1.840e-3
3	1.472	VB	0.2551	3.51967	1.68655e-1	8.134e-3
4	1.922	BB	0.1479	8.18339	8.03984e-1	0.0189
5	2.471	BV E	0.0942	5.12744e-1	7.12198e-2	1.185e-3
6	2.721	VB R	0.1542	258.94632	25.64293	0.5984
7	3.498	BV	0.1343	2.43296	2.42985e-1	5.623e-3
8	3.882	VB	0.2251	12.49943	7.85070e-1	0.0289
9	4.599	BB	0.1695	2.78652	2.24580e-1	6.440e-3
10	4.963	BV	0.1470	5.37204	5.38273e-1	0.0124

HPLC 12/21/2024 12:14:20 PM SYSTEM   Page 1 of 2

Data File D:\HPLC-DATA\Data\21-12-2024\UF-46.D
Sample Name: UF-46

Peak #	RetTime [min]	Type	Width [min]	Area [mAU*s]	Height [mAU]	Area %
11	5.254	VB	0.2164	33.12867	2.37852	0.0766
12	6.061	BBA	0.2444	4.29414e4	2734.56421	99.2413
Totals :				4.32697e4	2765.52081	

Figure S 83. HPLC Purity Analysis of Compound **5p**

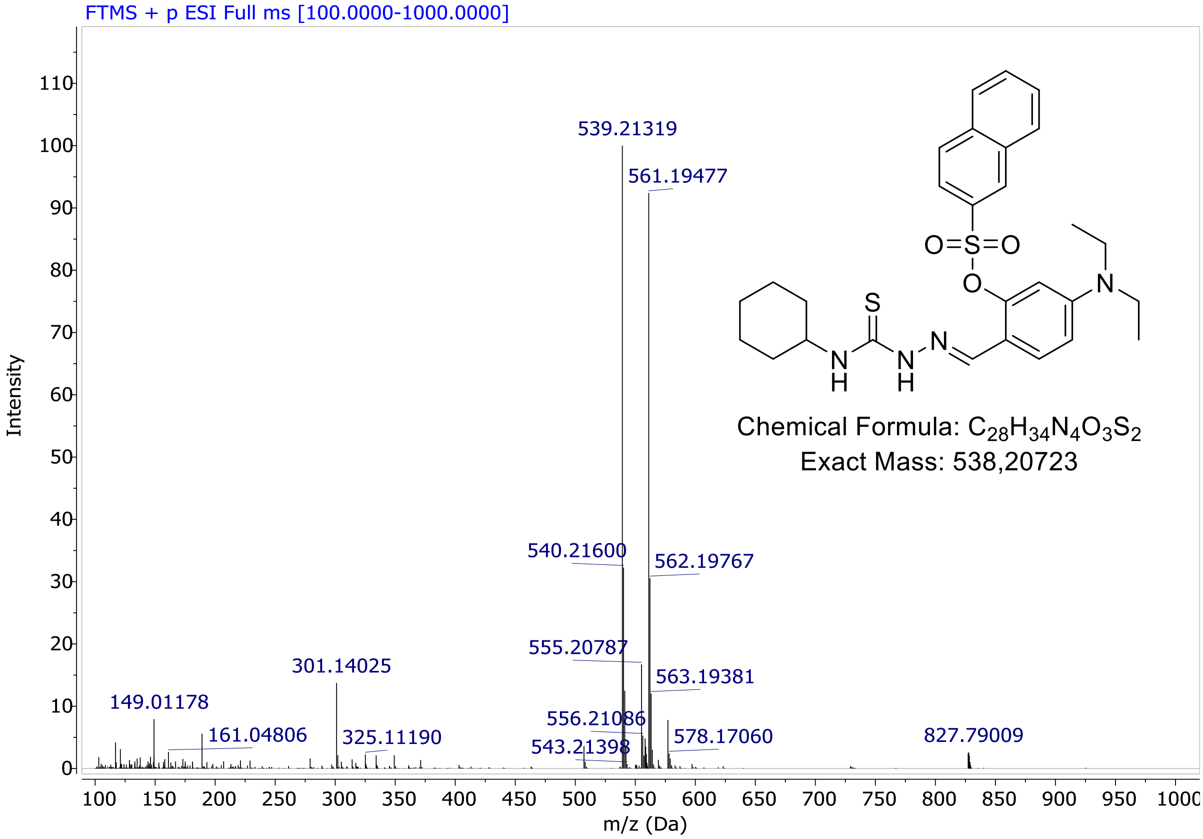

Figure S 84. ESI-HRMS Spectrum of Compound **5p**

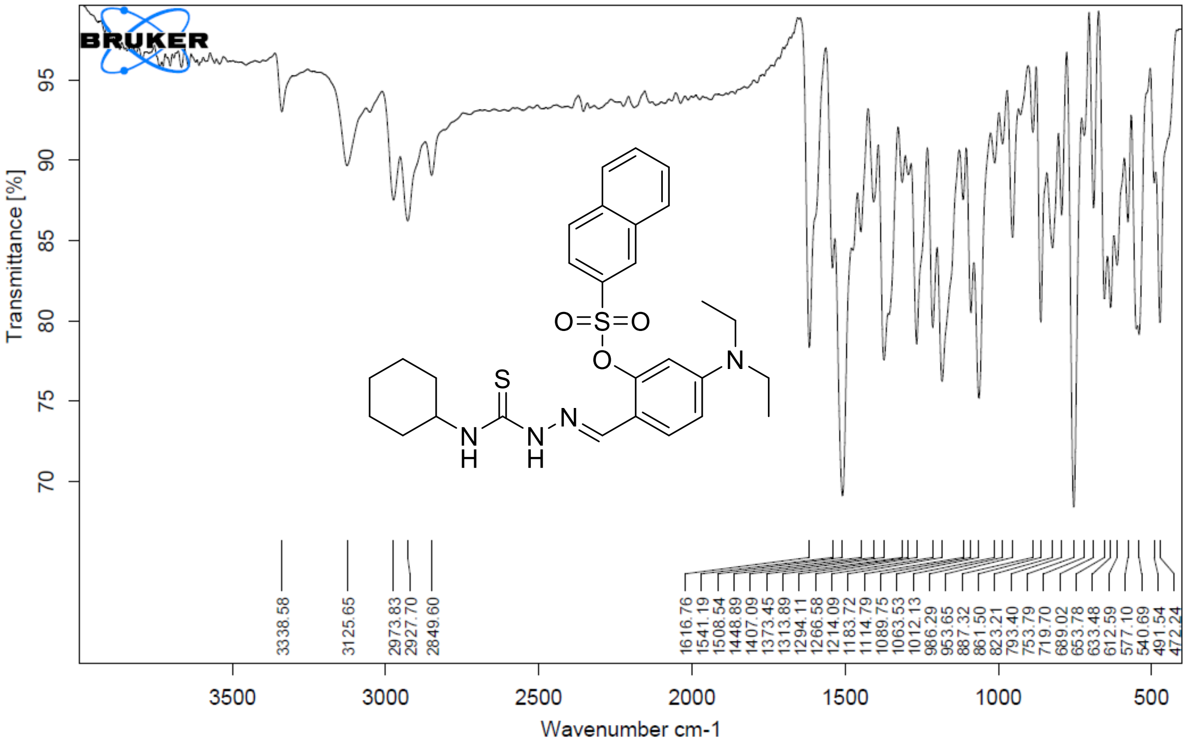

Figure S 85. FT-IR Spectrum of Compound **5p**

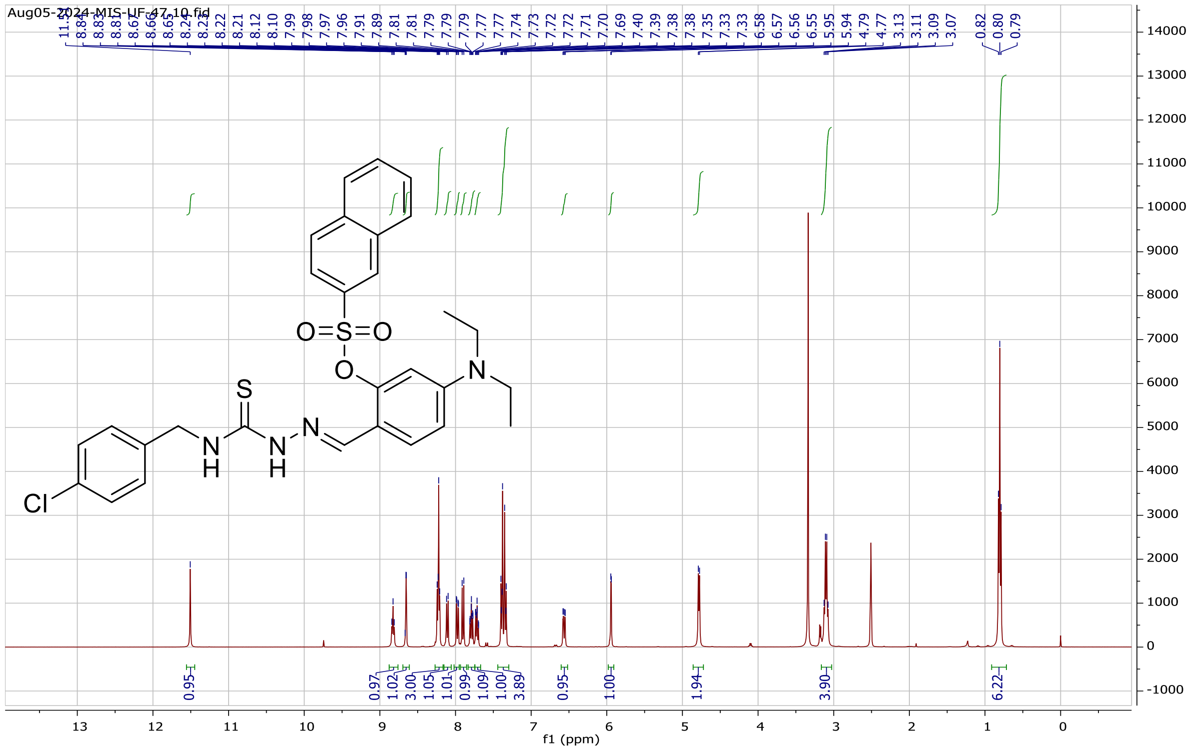

Figure S 86. ^{1}H-NMR Spectrum of Compound **5q** (DMSO-*d6*, 400 MHz)

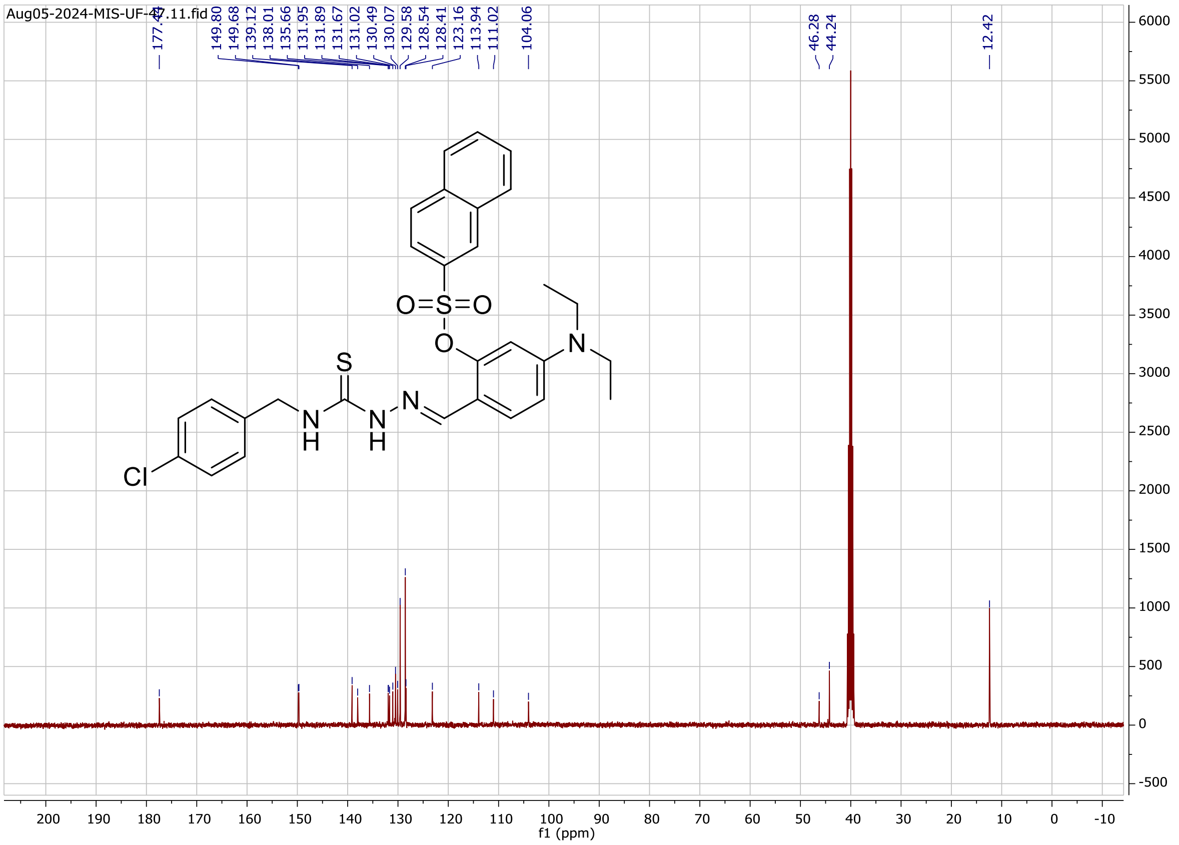

Figure S 87. ^{13}C-NMR Spectrum of Compound **5q** (DMSO-*d6*, 100 MHz)

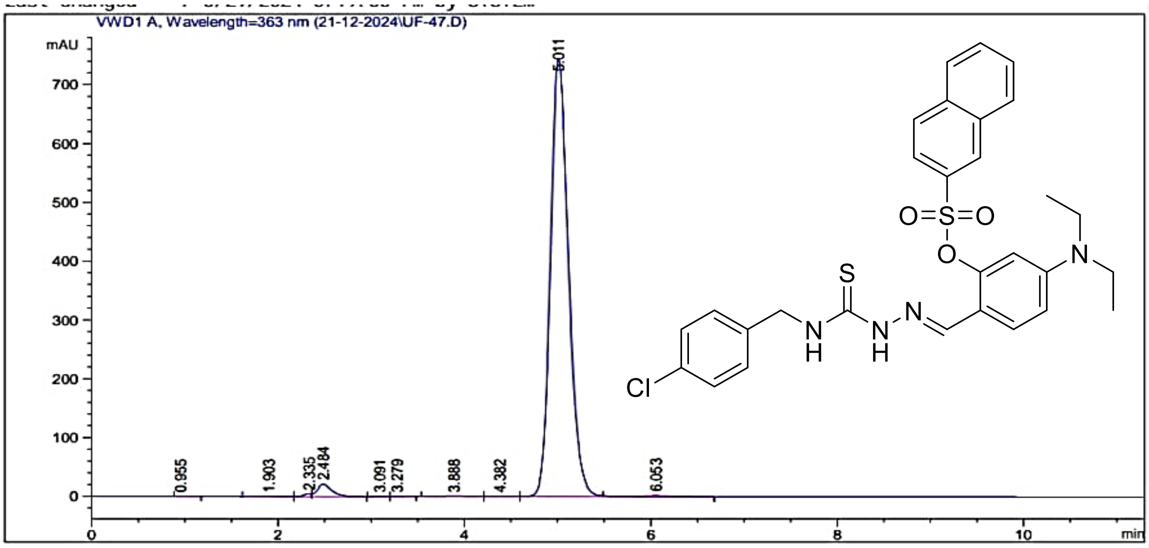

==

Area Percent Report

==

Sorted By : Signal
Multiplier : 1.0000
Dilution : 1.0000
Use Multiplier & Dilution Factor with ISTDs

Signal 1: VWD1 A, Wavelength=363 nm

Peak #	RetTime [min]	Type	Width [min]	Area [mAU*s]	Height [mAU]	Area %
1	0.955	BB	0.1288	6.93325e-1	7.46014e-2	6.733e-3
2	1.903	BV	0.1473	6.34860	6.42840e-1	0.0617
3	2.335	VV	0.0823	24.96835	4.70733	0.2425
4	2.484	VB	0.1558	225.25810	21.22508	2.1875
5	3.091	BV	0.1337	1.41315	1.51264e-1	0.0137
6	3.279	VB	0.1409	1.34782	1.30476e-1	0.0131
7	3.888	BV	0.2117	18.34126	1.30772	0.1781
8	4.382	VB	0.1758	6.35814	5.37870e-1	0.0617
9	5.011	BV R	0.2061	9982.56934	743.77142	96.9408
10	6.053	VB E	0.2889	30.29249	1.50387	0.2942

HPLC 12/21/2024 12:26:19 PM SYSTEM Page 1 of 2

Data File D:\HPLC-DATA\Data\21-12-2024\UF-47.D
Sample Name: UF-47

Peak #	RetTime [min]	Type	Width [min]	Area [mAU*s]	Height [mAU]	Area %
Totals :				1.02976e4	774.05248	

Figure S 88. HPLC Purity Analysis of Compound **5q**

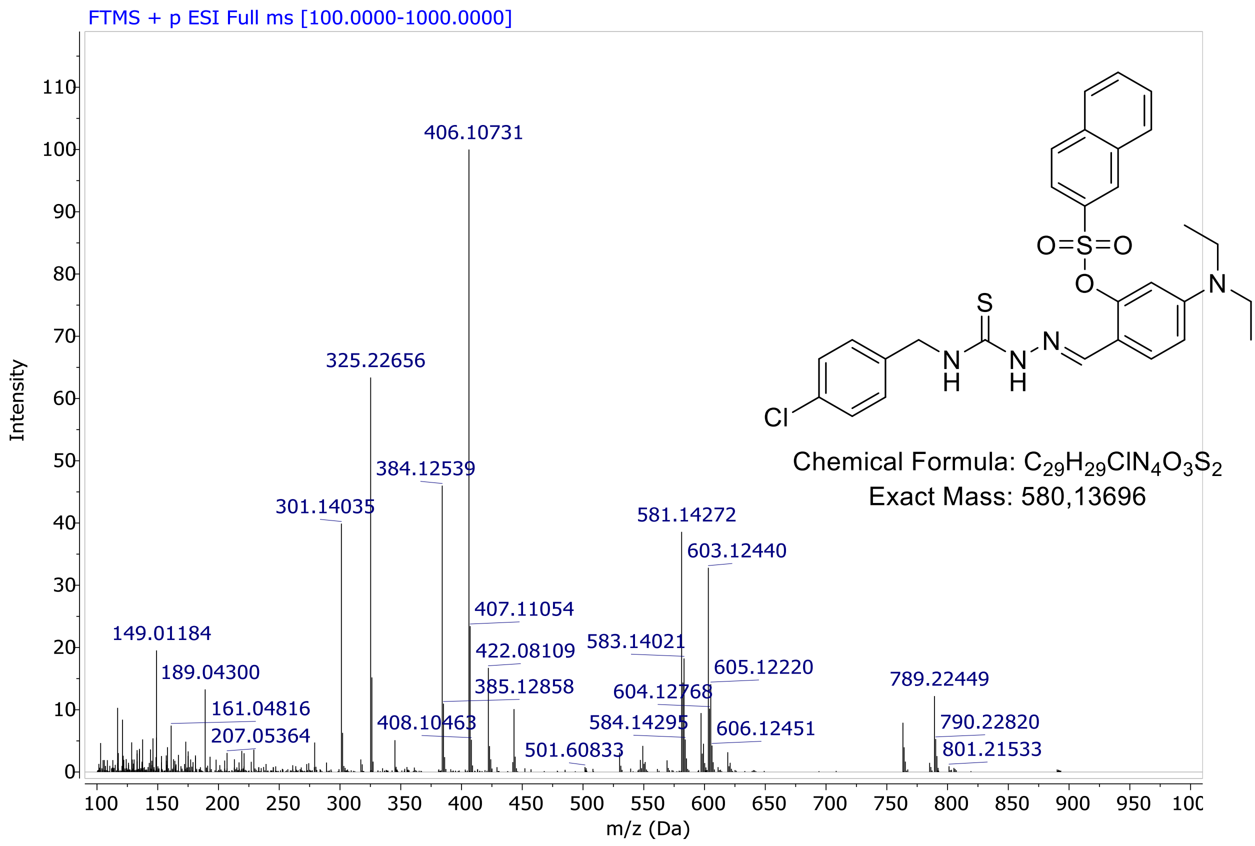

Figure S 89. ESI-HRMS Spectrum of Compound **5q**

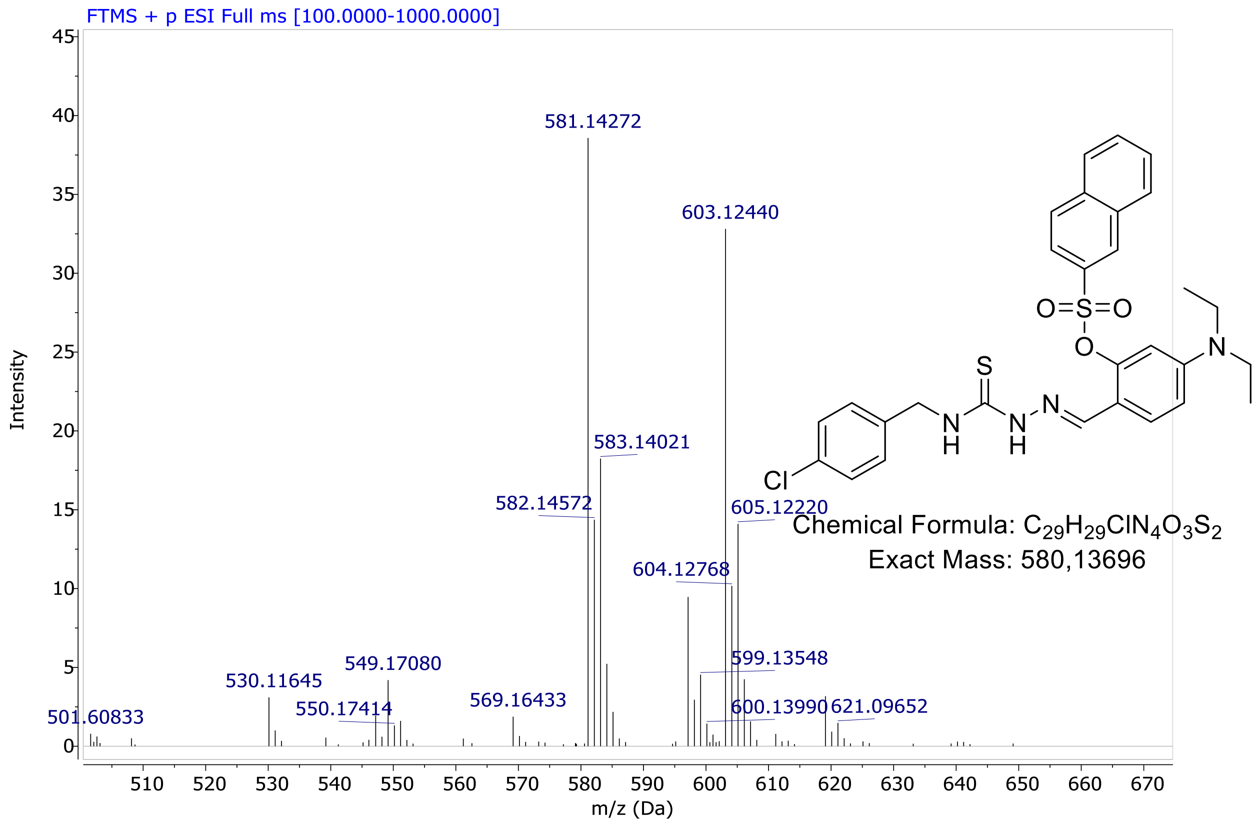

Figure S 90. ESI-HRMS Spectrum of Compound **5q** (extended)

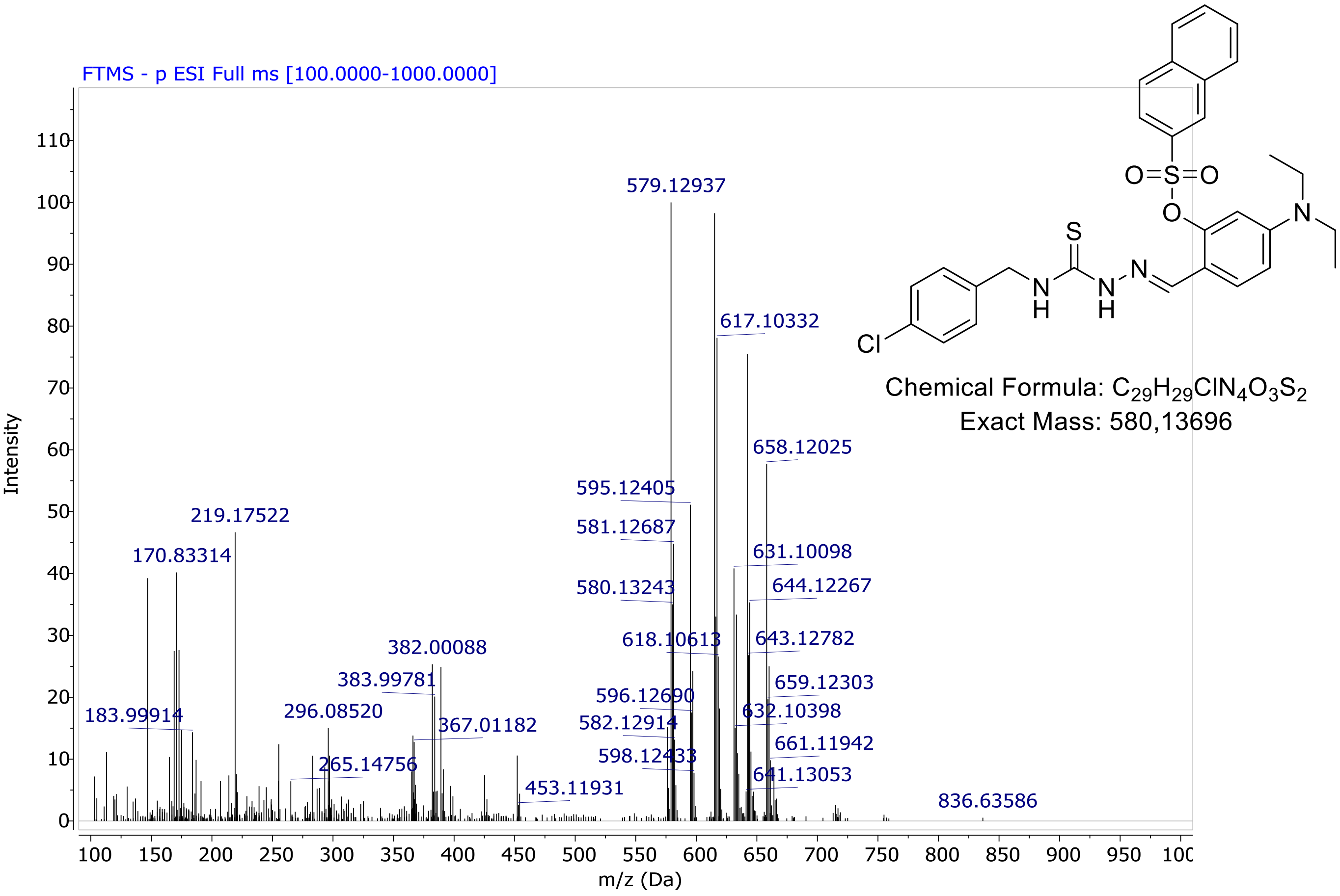

Figure S 91. ESI-HRMS Spectrum of Compound **5q** (negative)

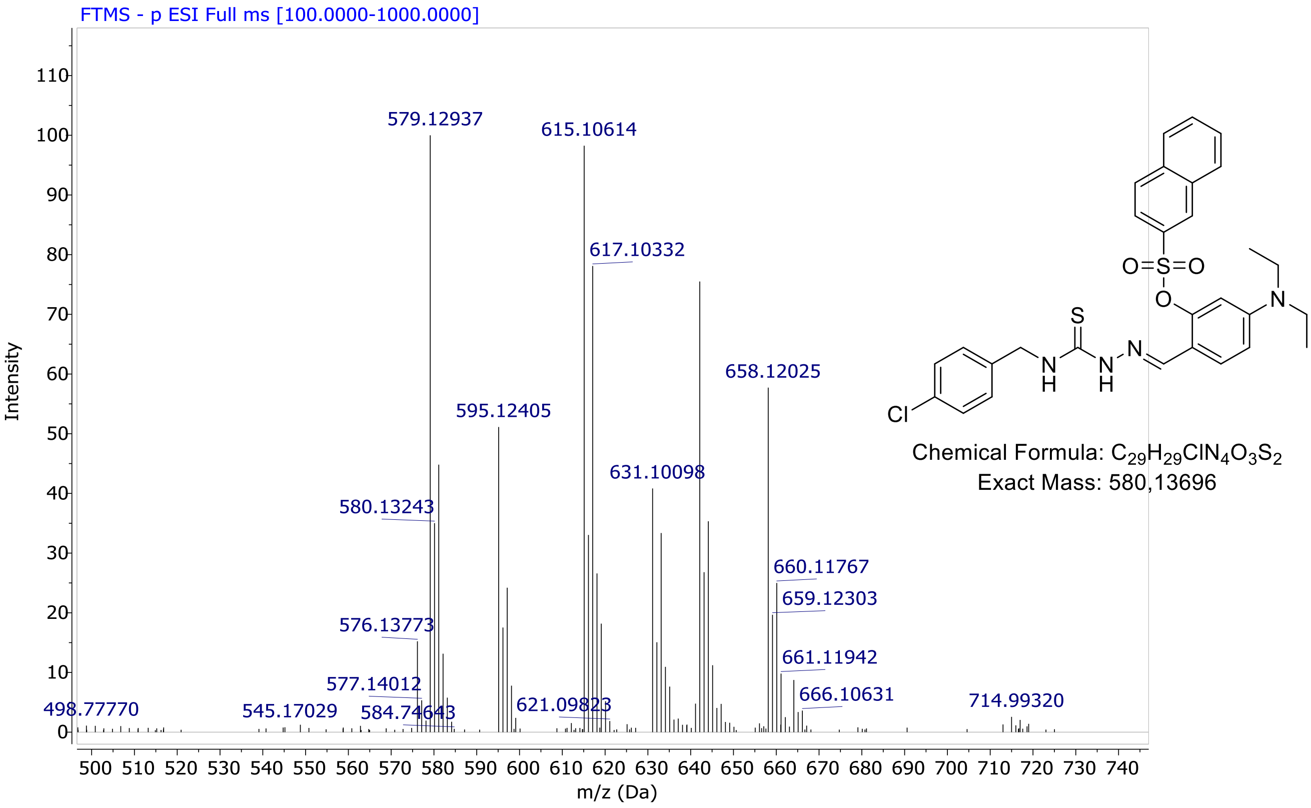

Figure S 92. ESI-HRMS Spectrum of Compound **5q** (negative-extended)

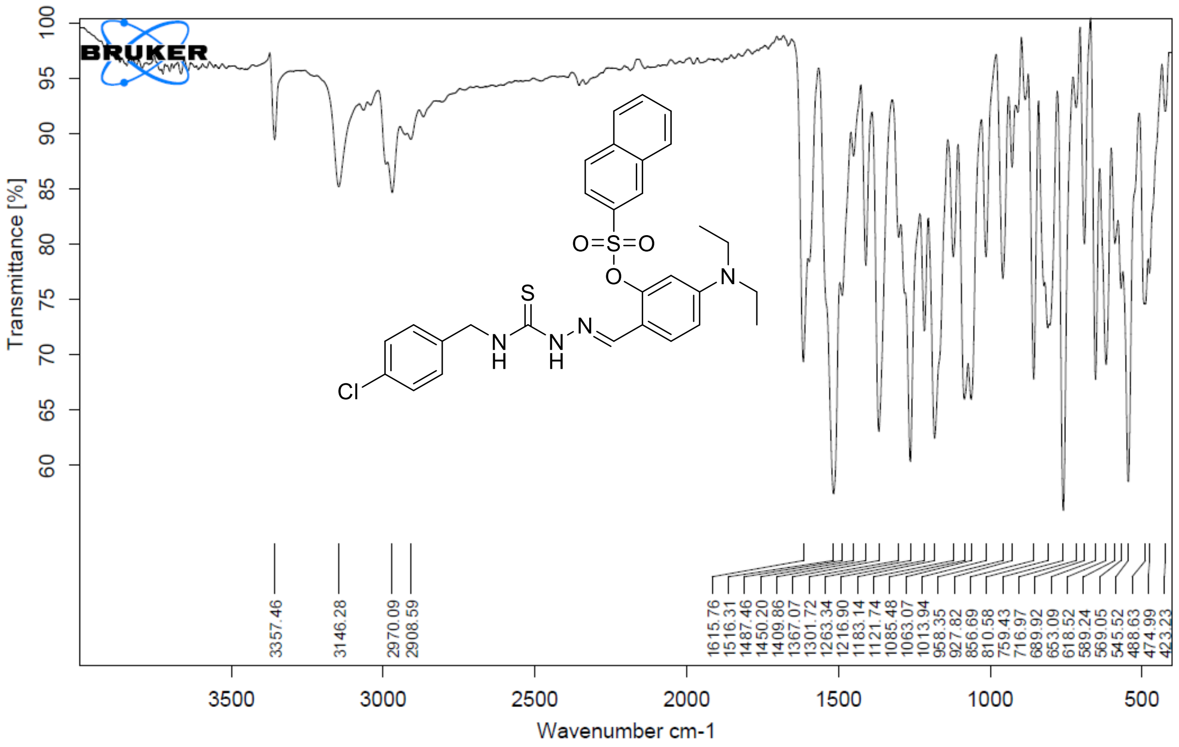

Figure S 93. FT-IR Spectrum of Compound **5q**

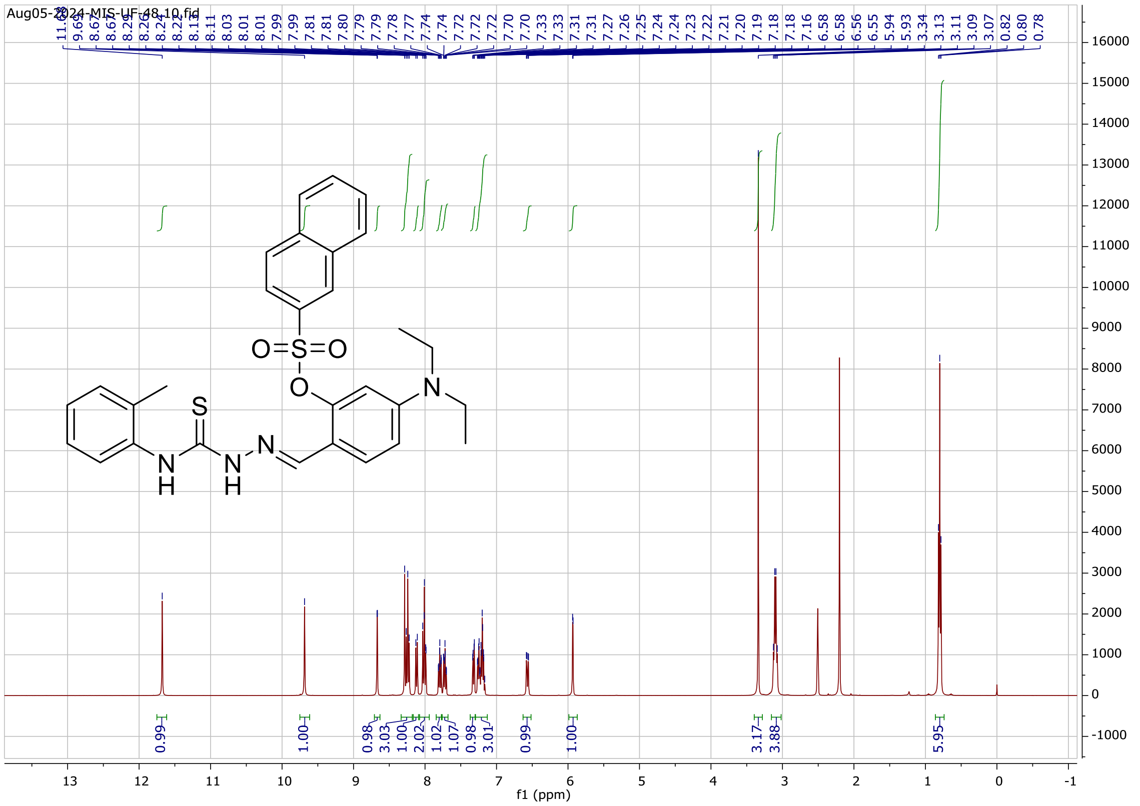

Figure S 94. ^{1}H-NMR Spectrum of Compound **5r** (DMSO-*d6*, 400 MHz)

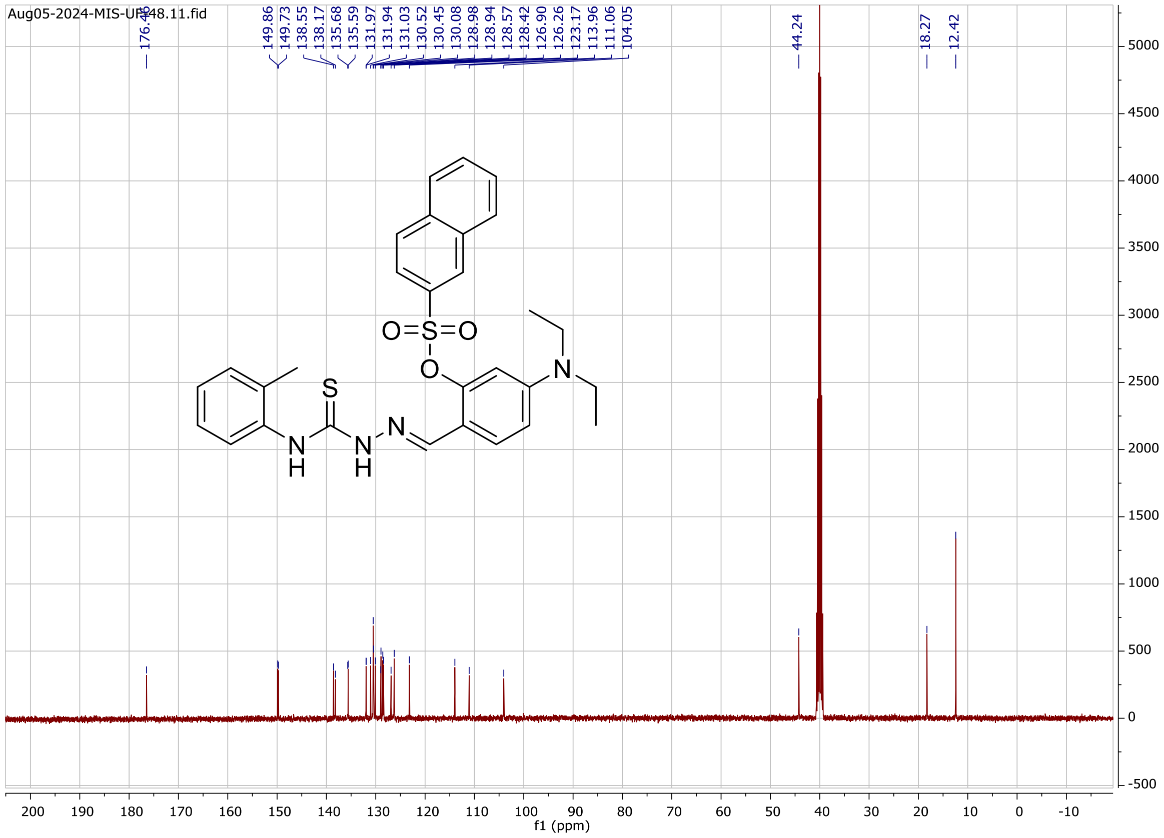

Figure S 95. ^{13}C-NMR Spectrum of Compound **5r** (DMSO-*d6*, 100 MHz)

Acq. Operator : SYSTEM
Sample Operator : SYSTEM
Acq. Instrument : HPLC Location : -
Injection Date : 12/21/2024 12:27:00 PM Inj : 1
Inj Volume : No inj
Method : D:\HPLC-DATA\Method\BZ-11.M
Last changed : 5/29/2024 5:19:35 PM by SYSTEM

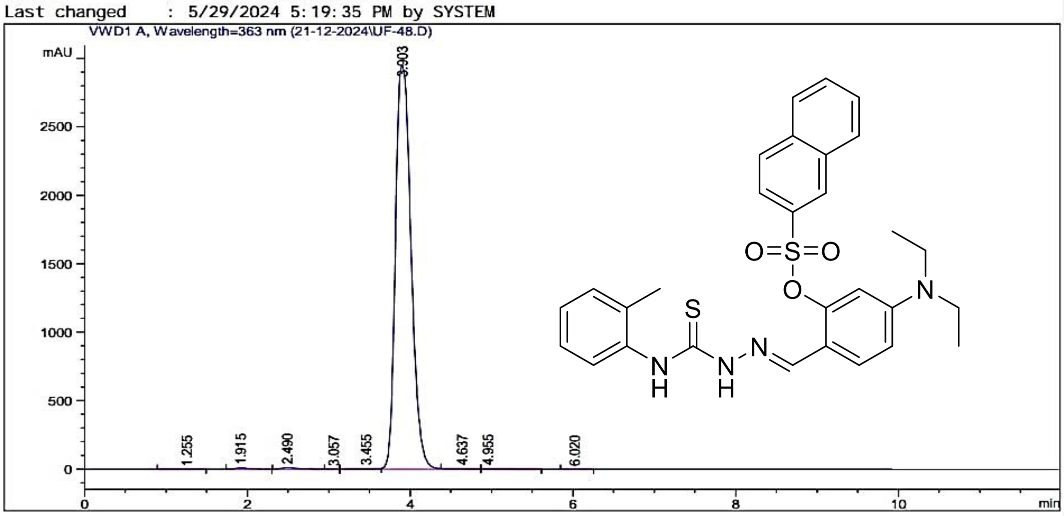

Area Percent Report

Sorted By : Signal
Multiplier : 1.0000
Dilution : 1.0000
Use Multiplier & Dilution Factor with ISTDs

Signal 1: VWD1 A, Wavelength=363 nm

Peak #	RetTime [min]	Type	Width [min]	Area [mAU*s]	Height [mAU]	Area %
1	1.255	BB	0.1811	2.44717	1.65617e-1	6.195e-3
2	1.915	BB	0.1395	78.98338	8.57653	0.1999
3	2.490	BV R	0.1545	120.32886	11.46142	0.3046
4	3.057	VB E	0.0757	3.77875e-1	6.58048e-2	9.565e-4
5	3.455	BV E	0.1823	37.00126	2.98792	0.0937
6	3.903	VV R	0.2077	3.92148e4	2946.11011	99.2663
7	4.637	VV E	0.2554	31.62241	1.74244	0.0800
8	4.955	VB E	0.2429	16.69571	9.91988e-1	0.0423
9	6.020	BB	0.1829	2.37197	1.74945e-1	6.004e-3

HPLC 12/21/2024 12:39:04 PM SYSTEM Page 1 of 2

Data File D:\HPLC-DATA\Data\21-12-2024\UF-48.D
Sample Name: UF-48

Totals : 3.95046e4 2972.27677

Figure S 96. HPLC Purity Analysis of Compound **5r**

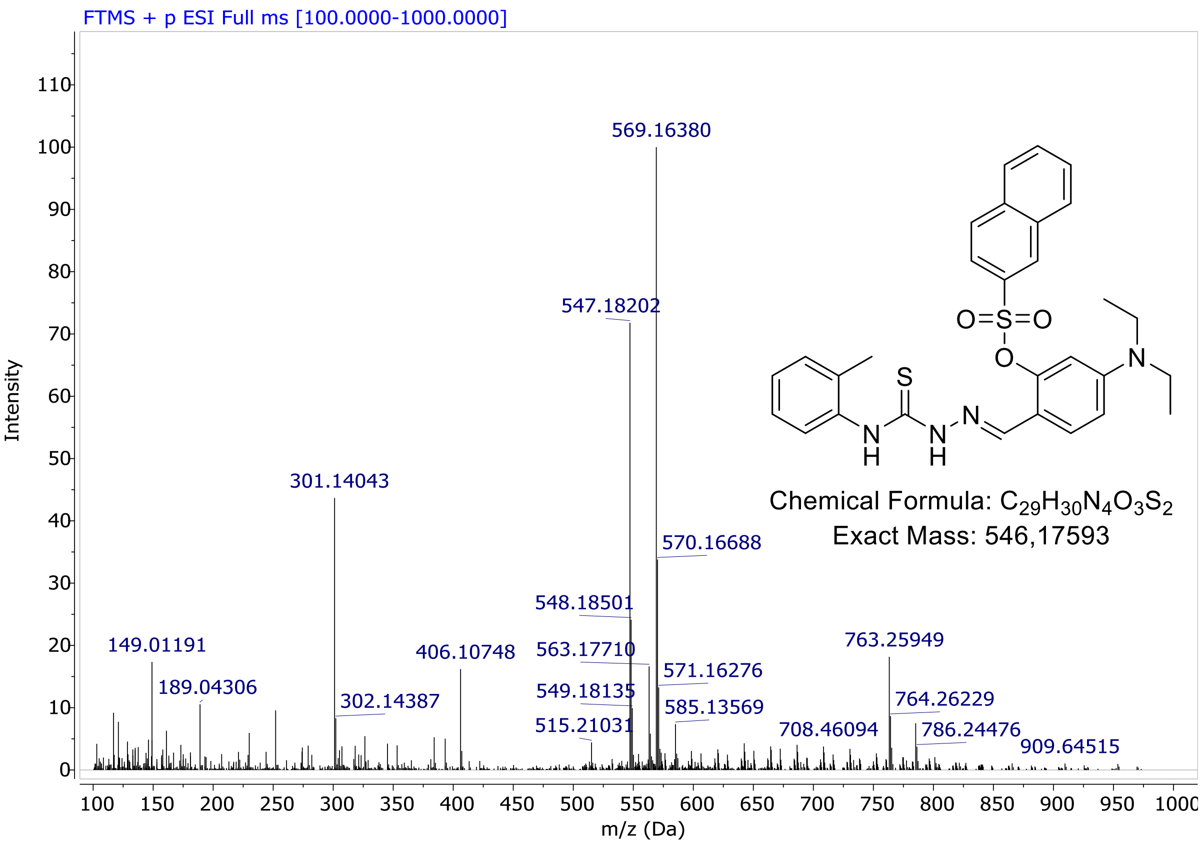

Figure S 97. ESI-HRMS Spectrum of Compound **5r**

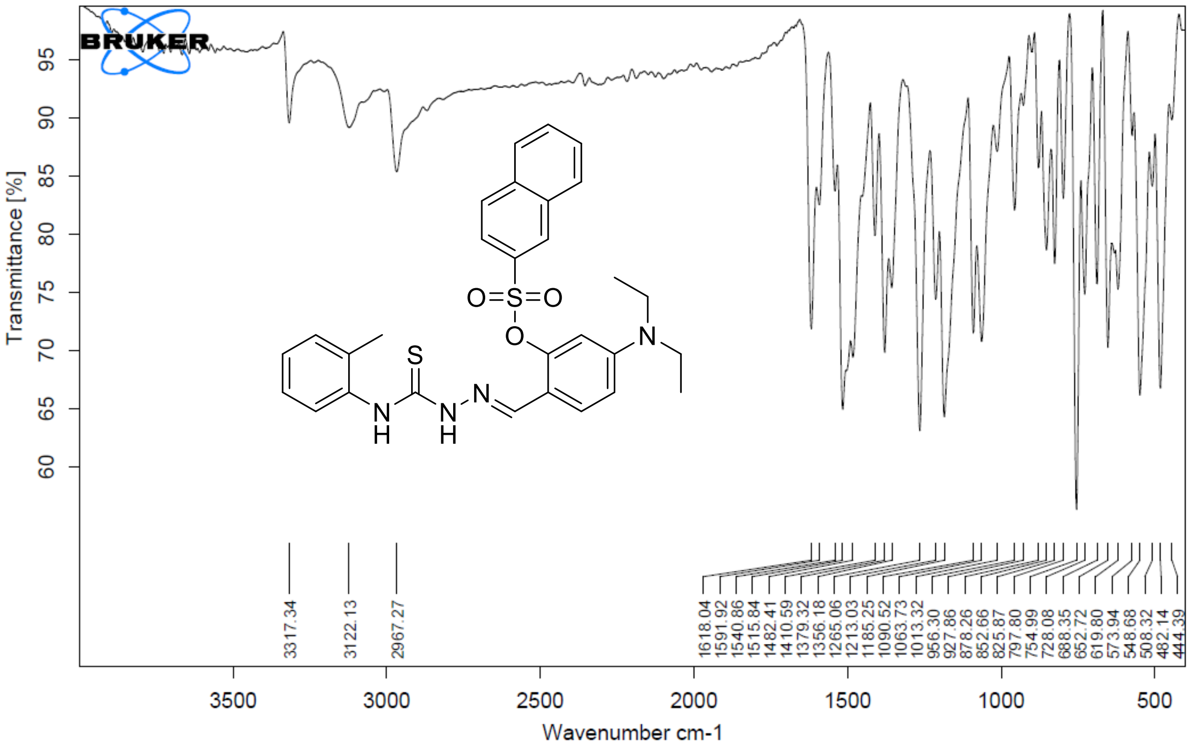

Figure S 98. FT-IR Spectrum of Compound **5r**

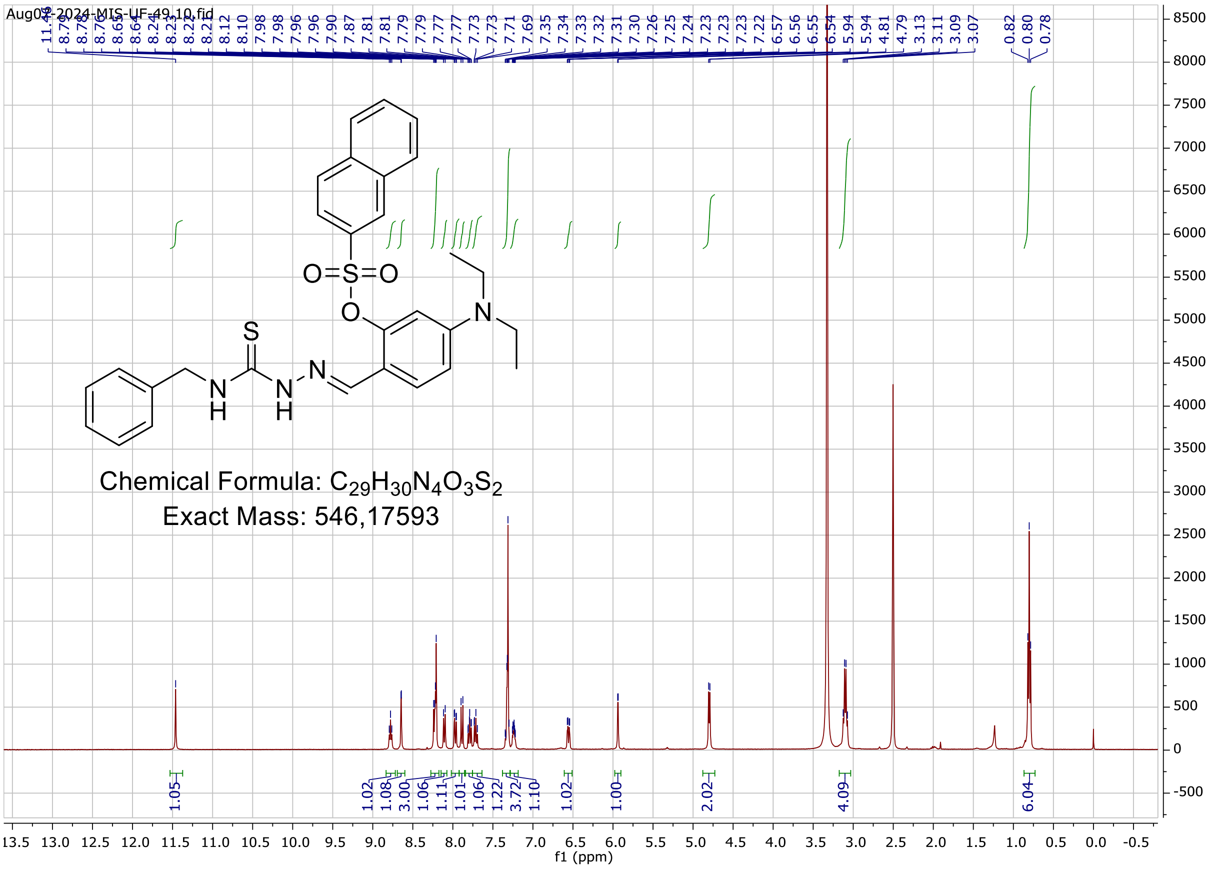

Figure S 99. ^{1}H-NMR Spectrum of Compound **5s** (DMSO-*d6*, 400 MHz)

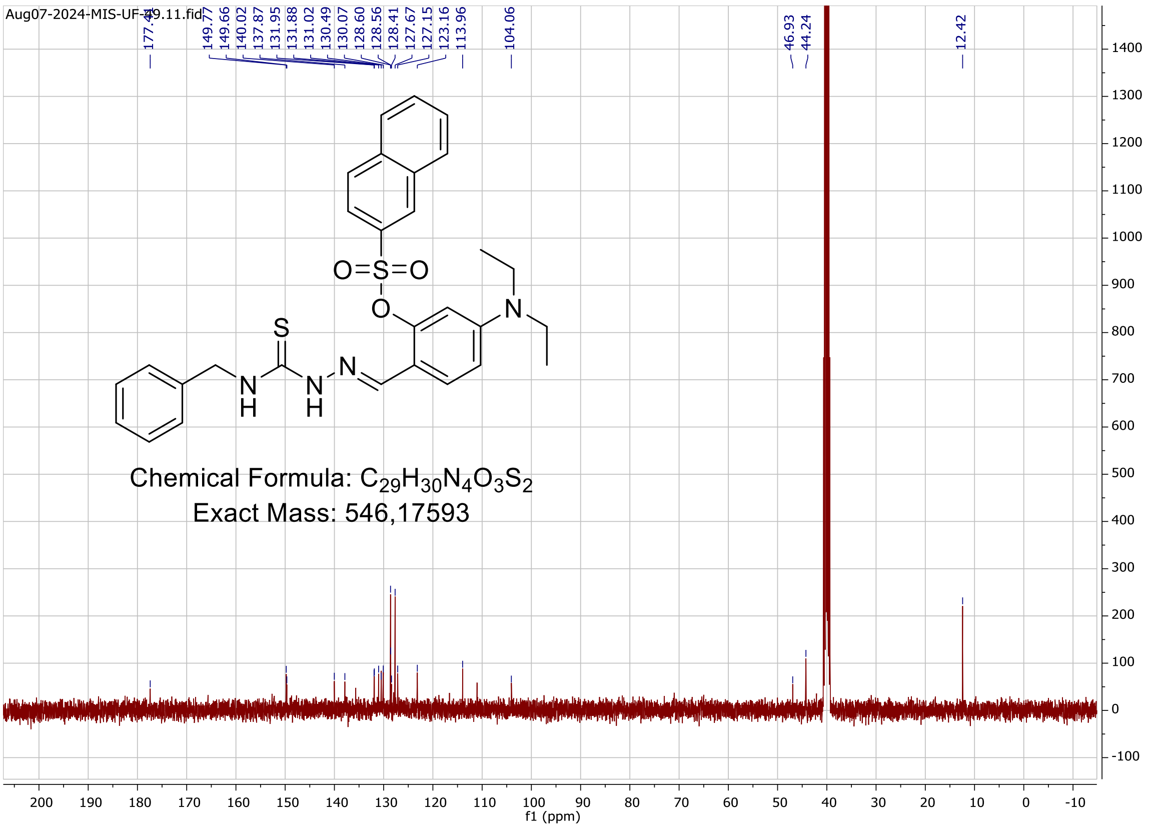

Figure S 100. ^{13}C-NMR Spectrum of Compound **5s** (DMSO-*d6*, 100 MHz)

Acq. Operator : SYSTEM
Sample Operator : SYSTEM
Acq. Instrument : HPLC Location : -
Injection Date : 12/21/2024 2:01:04 PM Inj : 1
Inj Volume : No inj
Method : D:\HPLC-DATA\Method\BZ-11.M
Last changed : 5/29/2024 5:19:35 PM by SYSTEM

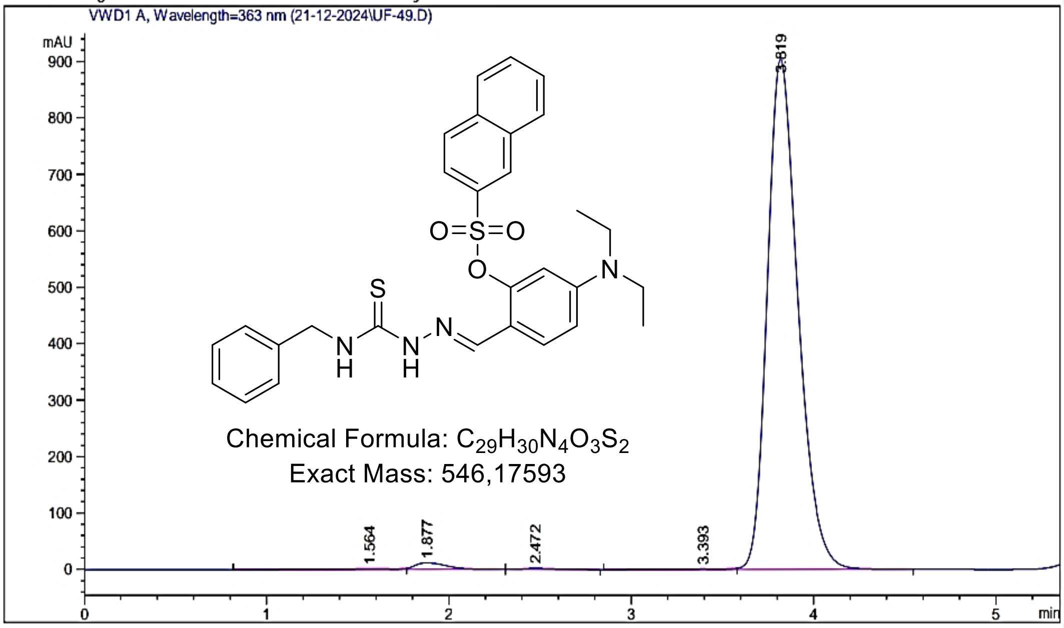

===
Area Percent Report
===

Sorted By : Signal
Multiplier : 1.0000
Dilution : 1.0000
Use Multiplier & Dilution Factor with ISTDs

Signal 1: VWD1 A, Wavelength=363 nm

Peak #	RetTime [min]	Type	Width [min]	Area [mAU*s]	Height [mAU]	Area %
1	1.564	BV E	0.2220	23.69232	1.45524	0.2204
2	1.877	VB R	0.1919	141.70142	11.49523	1.3179
3	2.472	BB	0.1564	20.30195	1.95004	0.1888
4	3.393	BV E	0.2277	13.83963	8.19171e-1	0.1287
5	3.819	VB R	0.1783	1.05524e4	904.45453	98.1442

Totals : 1.07520e4 920.17422

Figure S 101. HPLC Purity Analysis of Compound **5s**

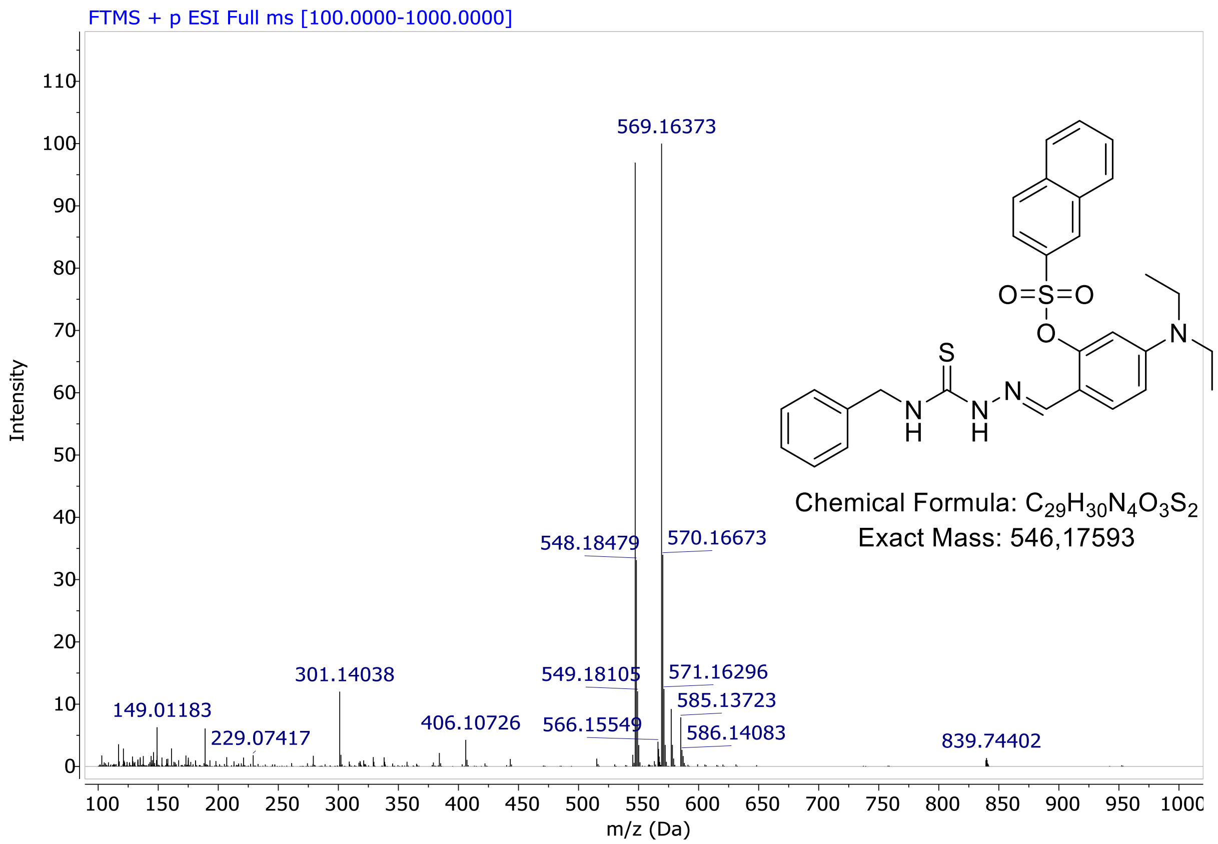

Figure S 102. ESI-HRMS Spectrum of Compound **5s**

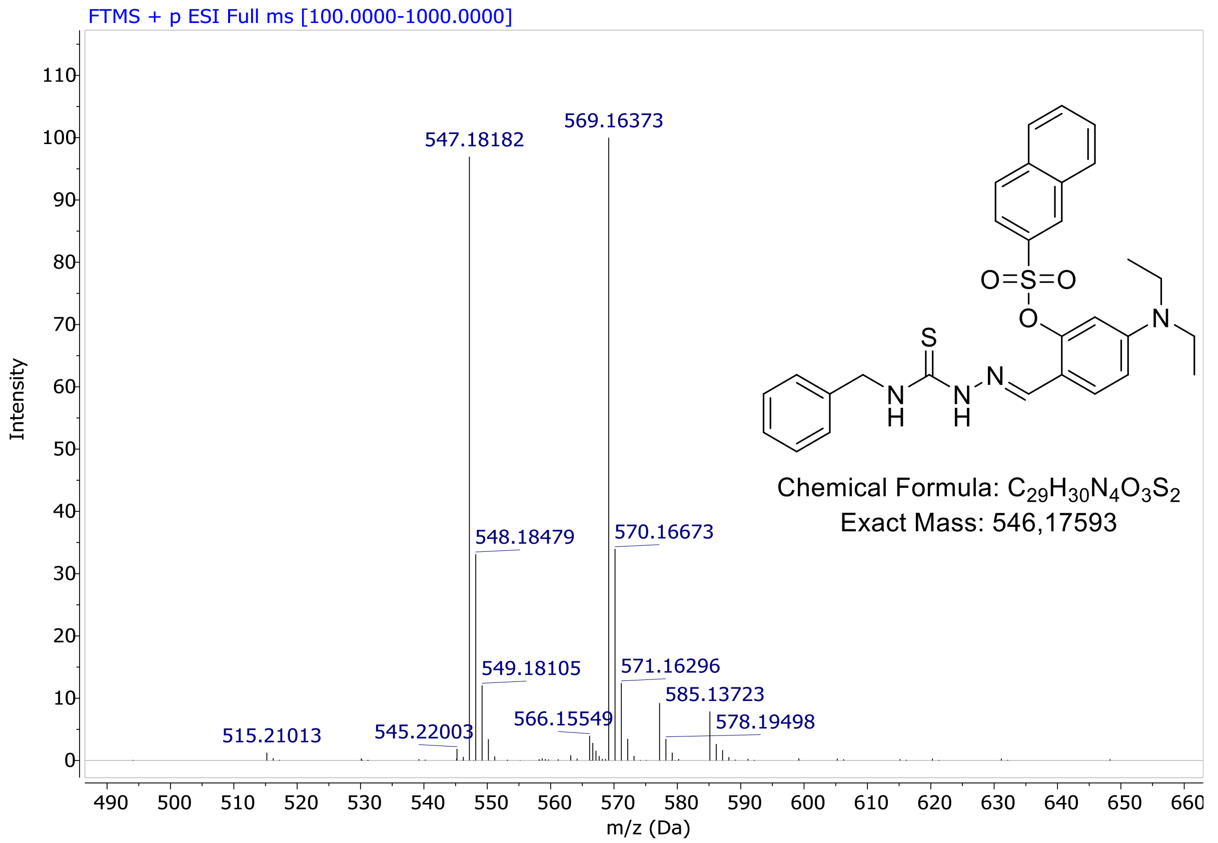

Figure S 103. ESI-HRMS Spectrum of Compound **5s** (extended)

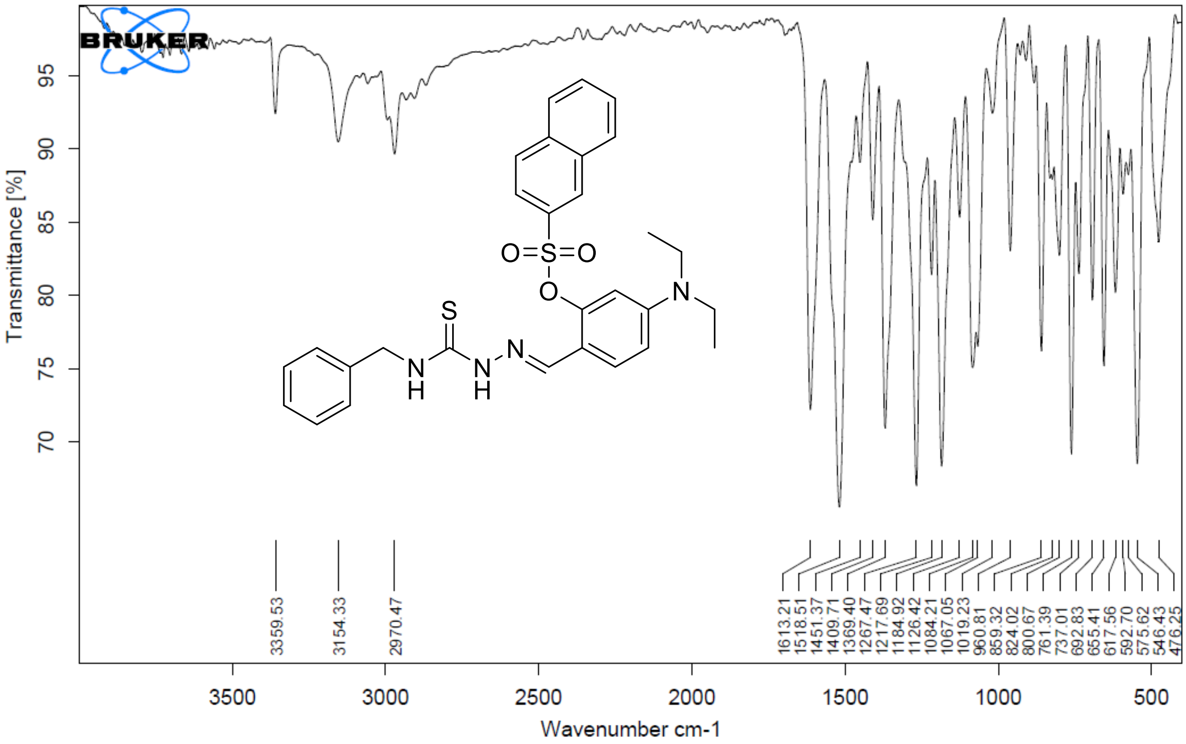

Figure S 104. FT-IR Spectrum of Compound **5s**

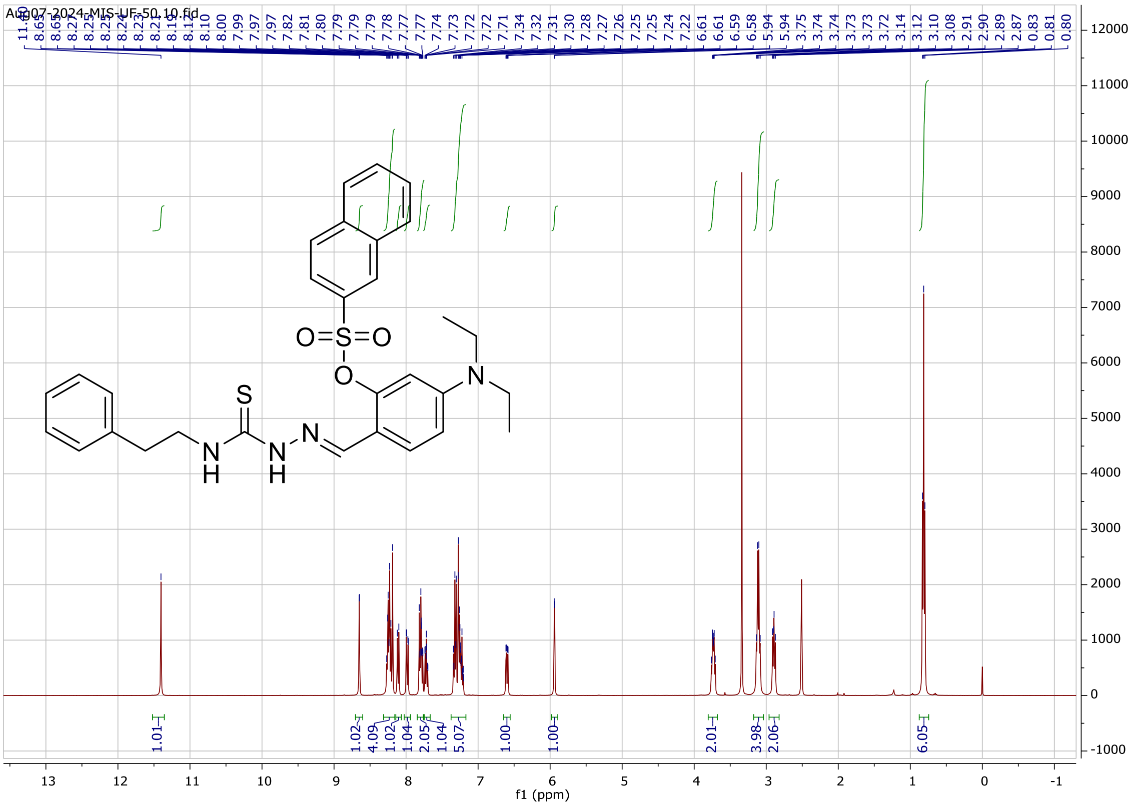

Figure S 105. ^{1}H-NMR Spectrum of Compound **5t** (DMSO-*d6*, 400 MHz)

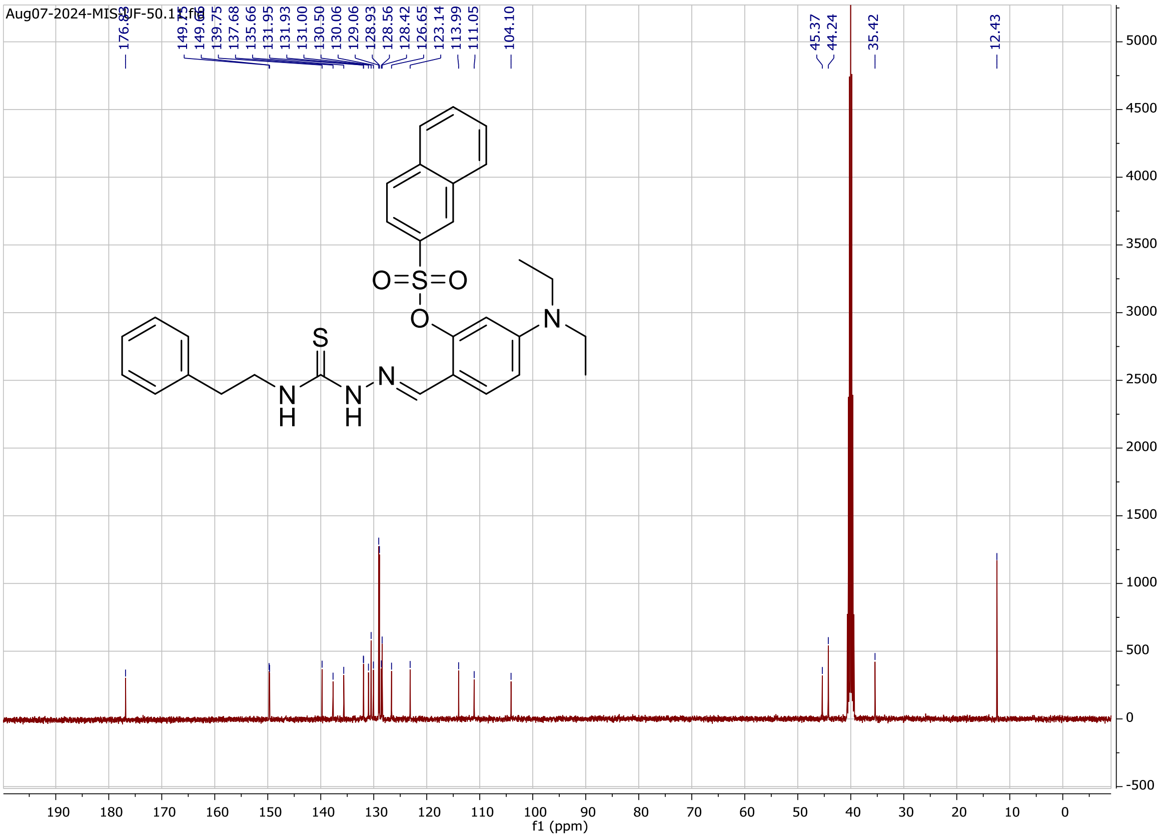

Figure S 106. ^{13}C-NMR Spectrum of Compound **5t** (DMSO-*d6*, 100 MHz)

```
===================================================================
Acq. Operator   : SYSTEM
Sample Operator : SYSTEM
Acq. Instrument : HPLC                          Location :     -
Injection Date  : 12/21/2024 1:40:50 PM               Inj :    1
                                             Inj Volume : No inj
Method          : D:\HPLC-DATA\Method\BZ-11.M
Last changed    : 5/29/2024 5:19:35 PM by SYSTEM
```

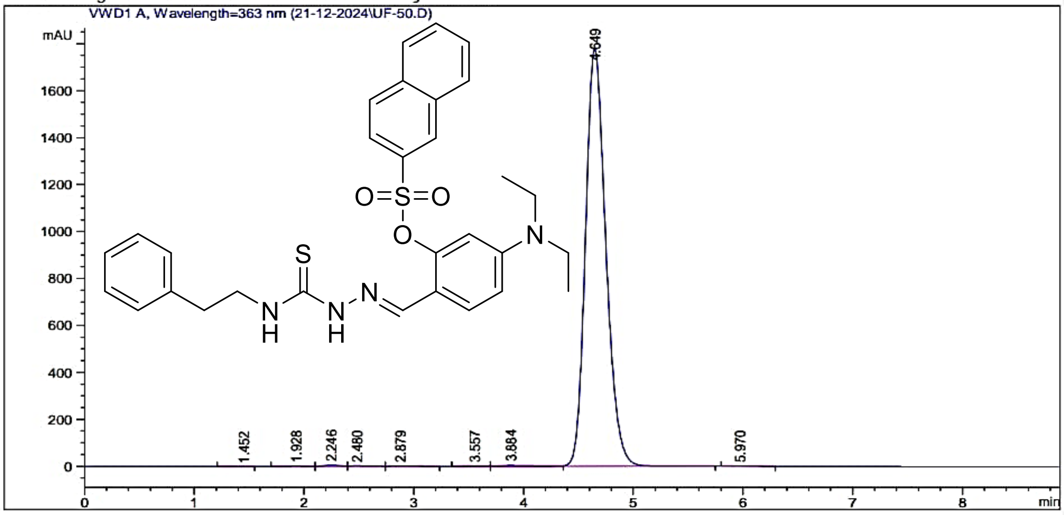


```
===================================================================
                      Area Percent Report
===================================================================

Sorted By             :      Signal
Multiplier            :      1.0000
Dilution              :      1.0000
Use Multiplier & Dilution Factor with ISTDs


Signal 1: VWD1 A, Wavelength=363 nm
```

Peak #	RetTime [min]	Type	Width [min]	Area [mAU*s]	Height [mAU]	Area %
1	1.452	BB	0.1612	6.54231e-1	5.05506e-2	2.844e-3
2	1.928	BB	0.1500	3.21834	2.96447e-1	0.0140
3	2.246	BV	0.1373	44.98896	5.05414	0.1955
4	2.480	VB	0.1452	20.86225	2.09753	0.0907
5	2.879	BB	0.1769	10.21445	8.48364e-1	0.0444
6	3.557	BV E	0.1817	8.02471	6.44265e-1	0.0349
7	3.884	VV E	0.2301	63.31086	3.84301	0.2752
8	4.649	VB R	0.1981	2.28522e4	1777.04077	99.3252
9	5.970	BB	0.1938	3.97989	3.09367e-1	0.0173

HPLC 12/21/2024 1:49:50 PM SYSTEM   Page 1 of 2

Data File D:\HPLC-DATA\Data\21-12-2024\UF-50.D
Sample Name: UF-50

Totals :	2.30075e4	1790.18445

Figure S 107. HPLC Purity Analysis of Compound **5t**

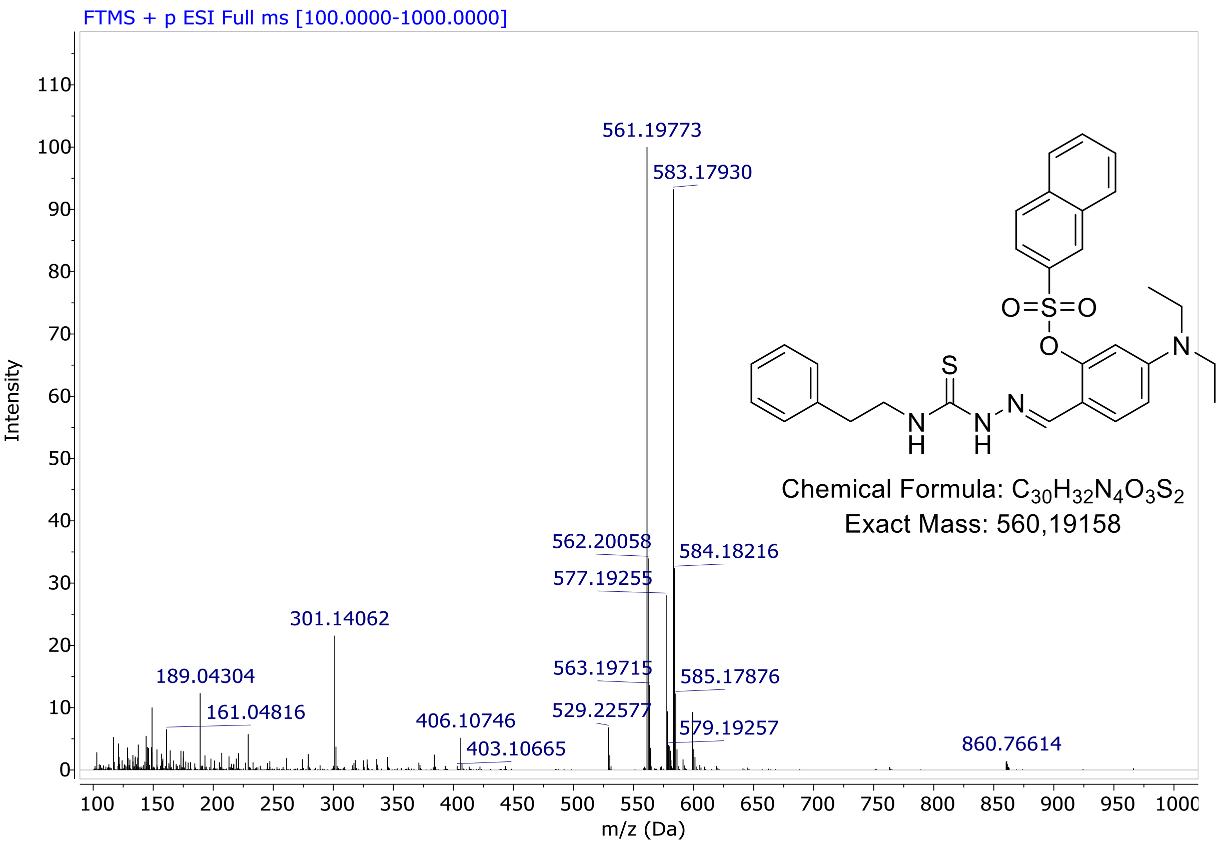

Figure S 108. ESI-HRMS Spectrum of Compound **5t**

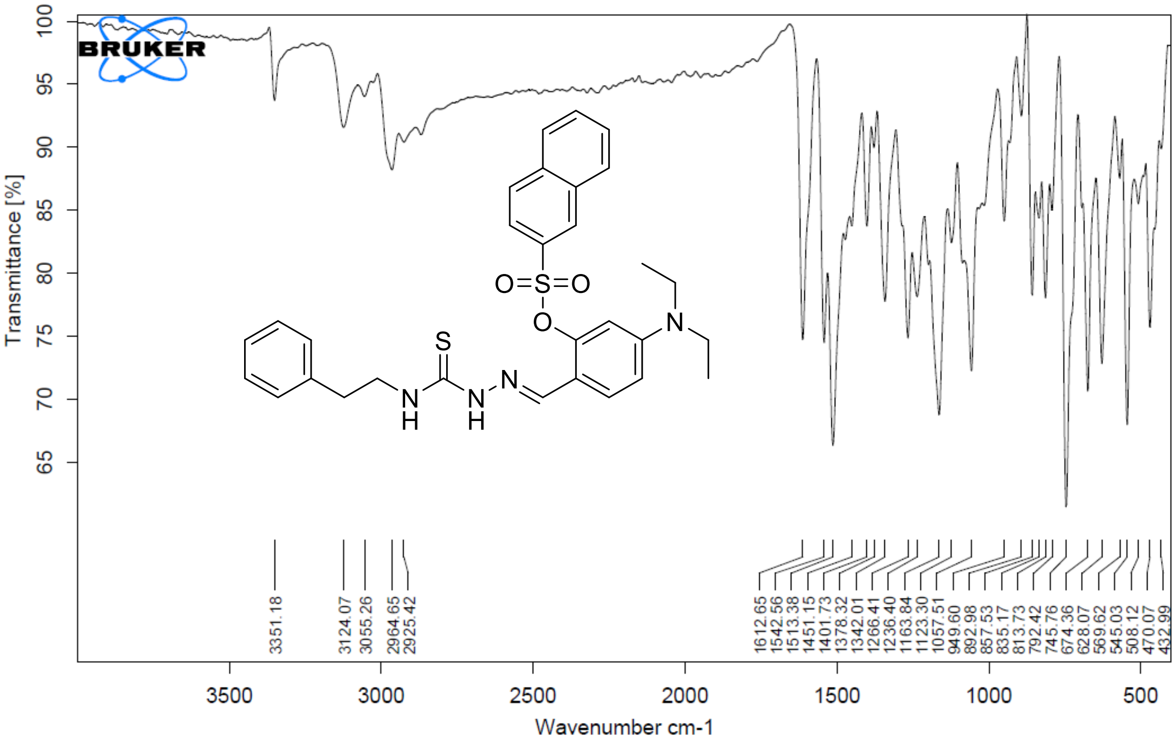

Figure S 109. FT-IR Spectrum of Compound **5t**

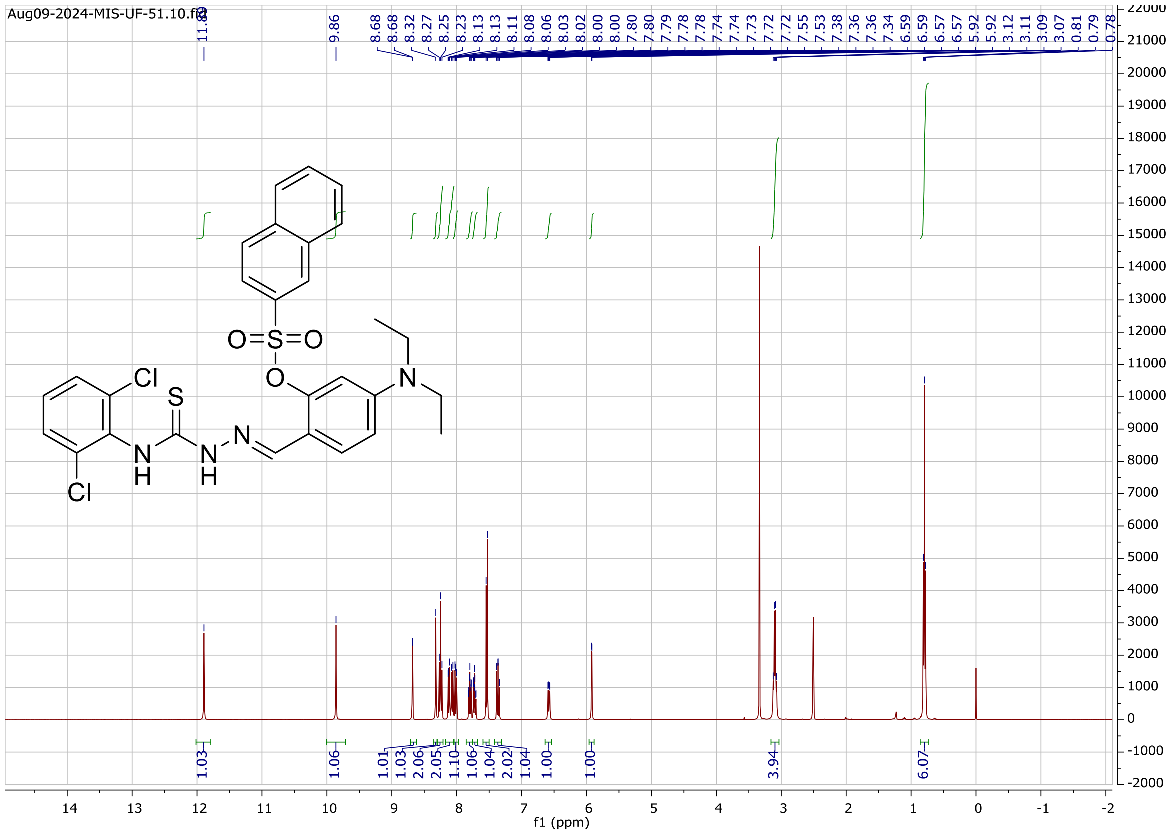

Figure S 110. ^{1}H-NMR Spectrum of Compound **5u** (DMSO-*d6*, 400 MHz)

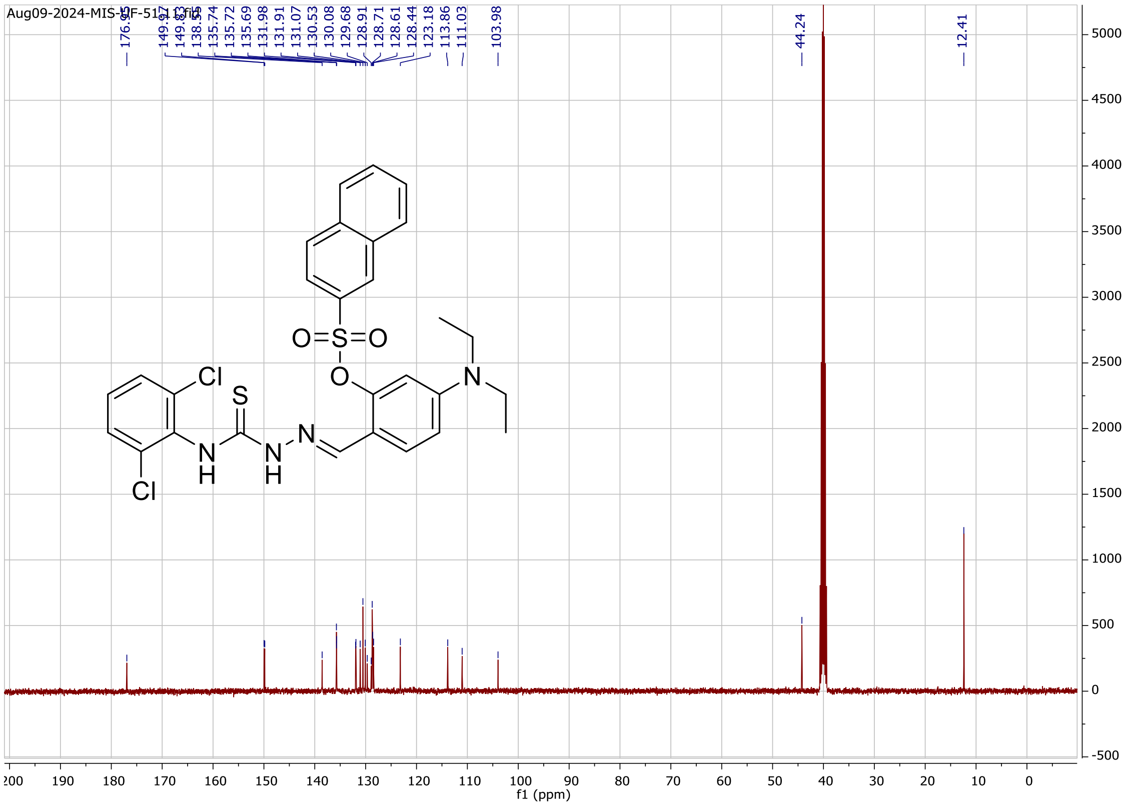

Figure S 111. ^{13}C-NMR Spectrum of Compound **5u** (DMSO-*d6*, 100 MHz)

```
==================================================================
Acq. Operator   : SYSTEM
Sample Operator : SYSTEM
Acq. Instrument : HPLC                          Location :     -
Injection Date  : 12/21/2024 1:50:15 PM              Inj :    1
                                              Inj Volume : No inj
Method          : D:\HPLC-DATA\Method\BZ-11.M
Last changed    : 5/29/2024 5:19:35 PM by SYSTEM
```

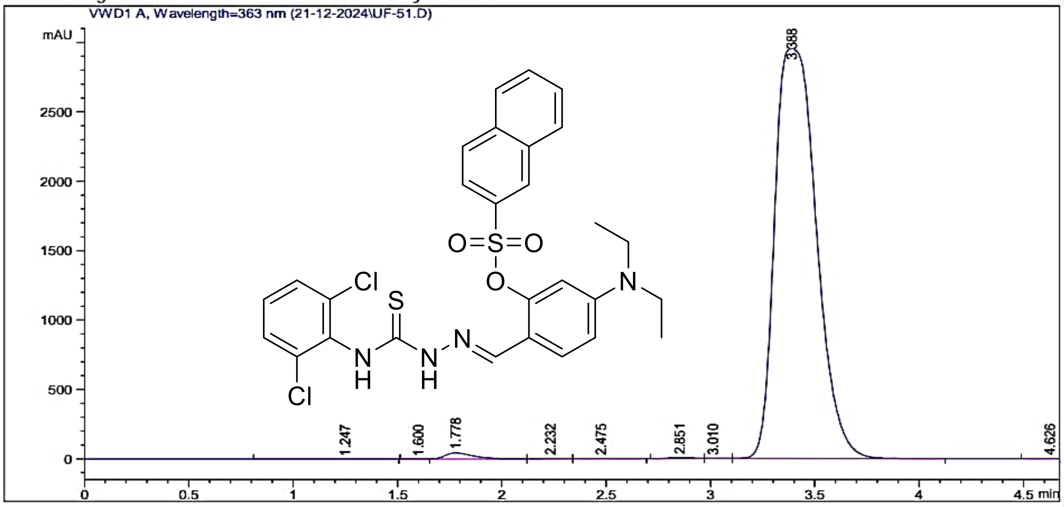


```
==================================================================
                     Area Percent Report
==================================================================

Sorted By             :      Signal
Multiplier            :      1.0000
Dilution              :      1.0000
Use Multiplier & Dilution Factor with ISTDs


Signal 1: VWD1 A, Wavelength=363 nm
```

Peak #	RetTime [min]	Type	Width [min]	Area [mAU*s]	Height [mAU]	Area %
1	1.247	BB	0.2172	4.89873	3.03933e-1	0.0119
2	1.600	BV E	0.0731	4.82358e-1	1.03841e-1	1.168e-3
3	1.778	VB R	0.1383	389.60217	42.79192	0.9430
4	2.232	BV	0.1139	4.29310	5.62105e-1	0.0104
5	2.475	VB	0.1386	24.67865	2.66429	0.0597
6	2.851	BV	0.1318	55.34562	6.47528	0.1340
7	3.010	VB	0.0829	10.20602	1.86067	0.0247
8	3.388	BB	0.2181	4.08224e4	2952.30591	98.8102
9	4.626	BBA	0.1086	2.04718	3.14230e-1	4.955e-3

Data File D:\HPLC-DATA\Data\21-12-2024\UF-51.D
Sample Name: UF-51

Totals : 4.13139e4 3007.38218

Figure S 112. HPLC Purity Analysis of Compound **5u**

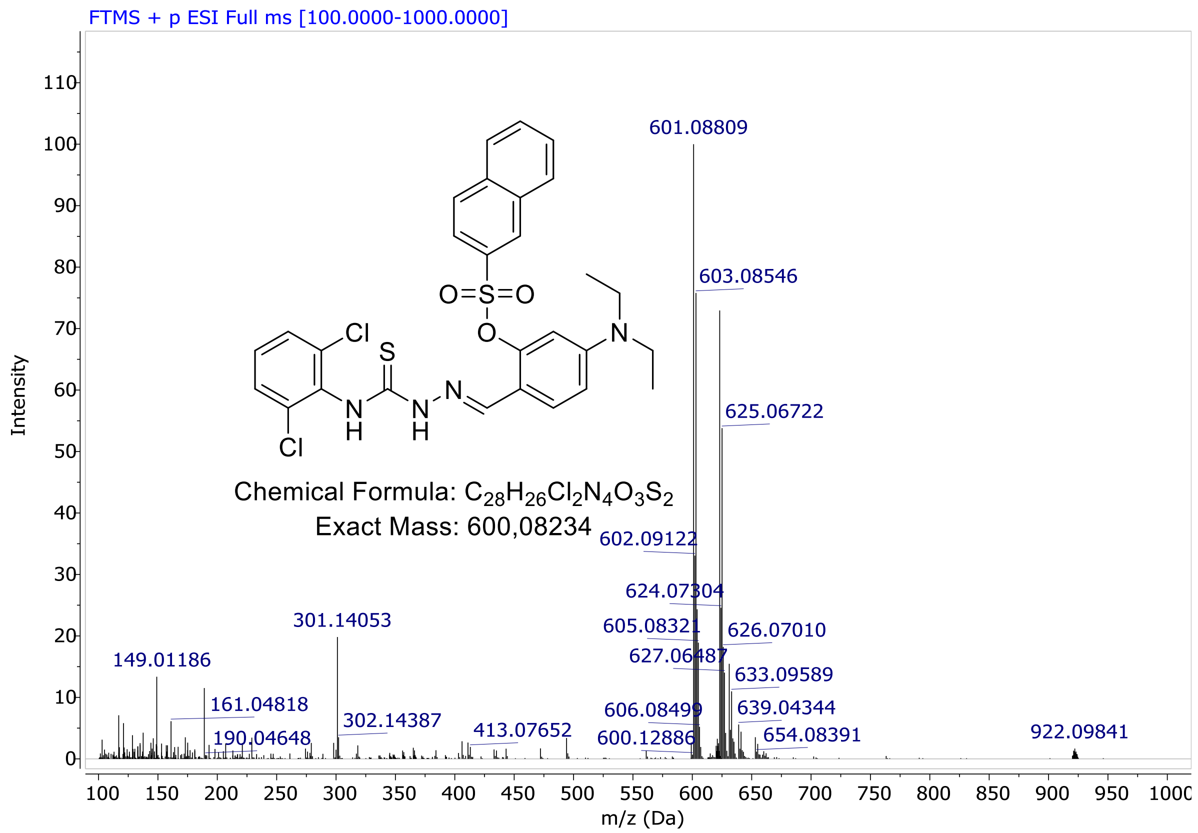

Figure S 113. ESI-HRMS Spectrum of Compound **5u**

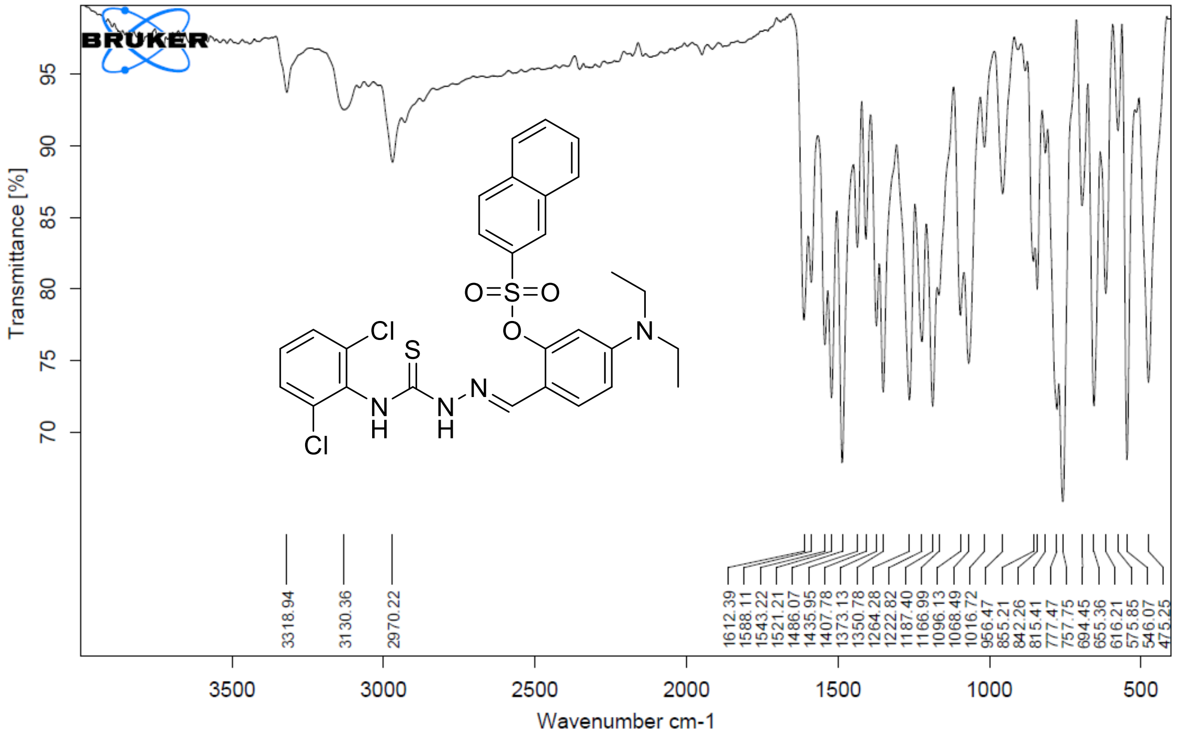

Figure S 114. FT-IR Spectrum of Compound **5u**

0	0.04201	0.07501	0.105603	0.12042	0.140511
100	73.63	65	49.09	41	36.36

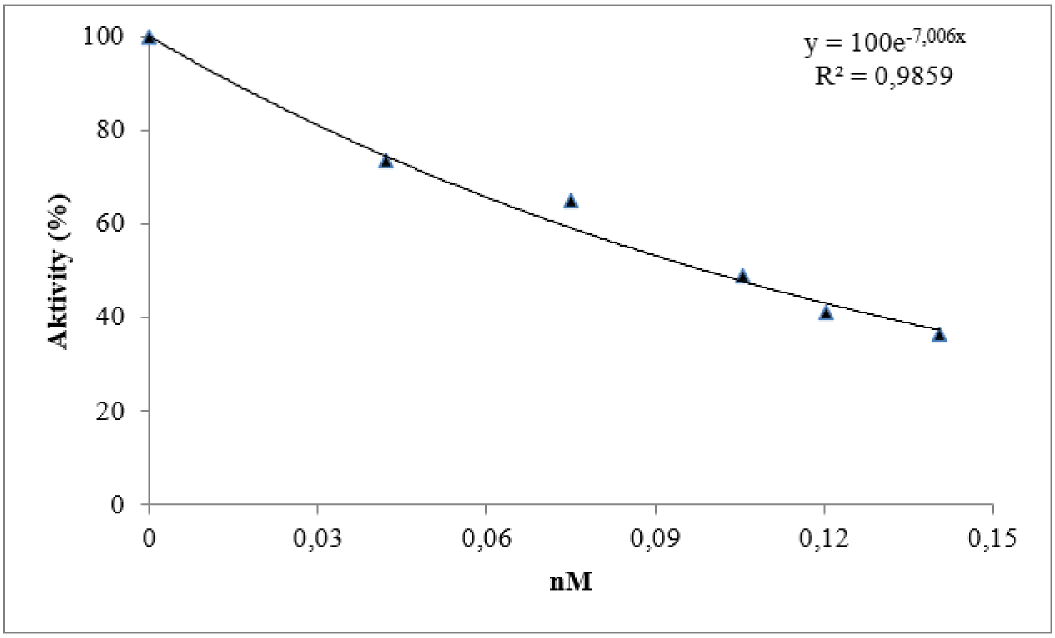

Figure S 115. Inhibition Curve of Compound **5a**

0	0.052469	0.083703	0.135062	0.150802	0.170926
100	73.37	60.68	51.28	42.37	38.46

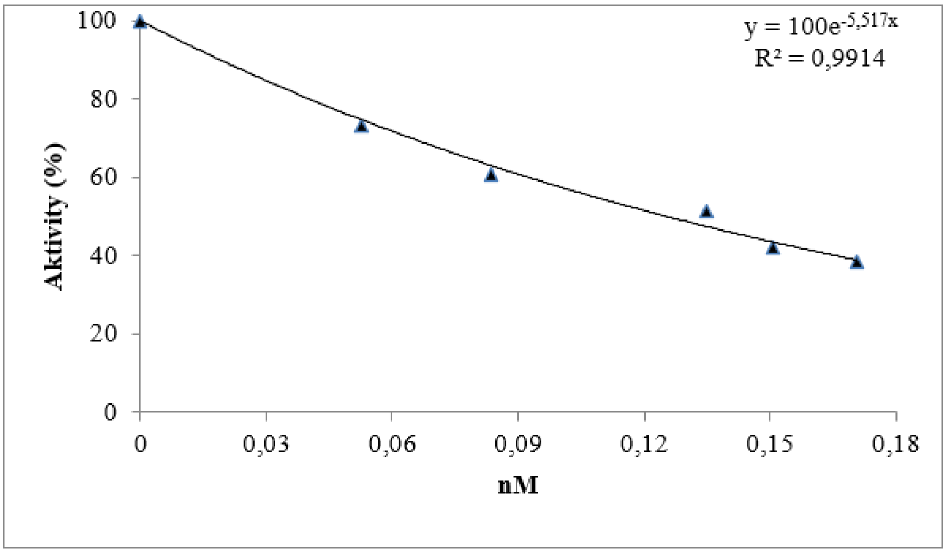

Figure S 116. Inhibition Curve of Compound **5b**

0	0.081315	0.121842	0.19315	0.223947	0.250473
100	68.33	58.33	47.5	37.5	30.83

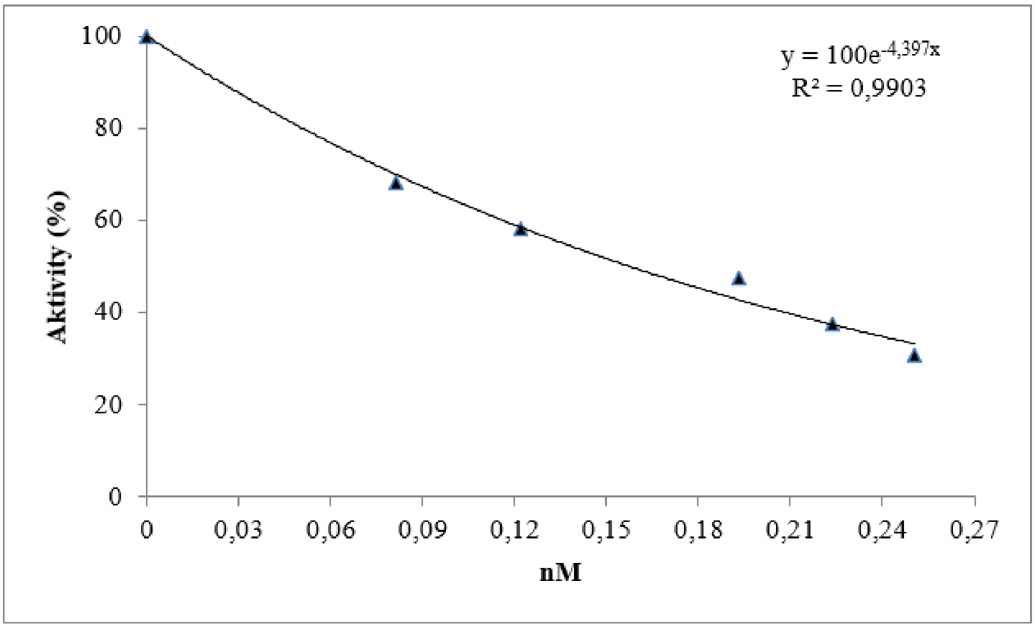

Figure S 117. Inhibition Curve of Compound **5c**

0	0.081015	0.130152	0.180267	0.195036	0.210393
100	80.83	67.5	48.33	40.83	35

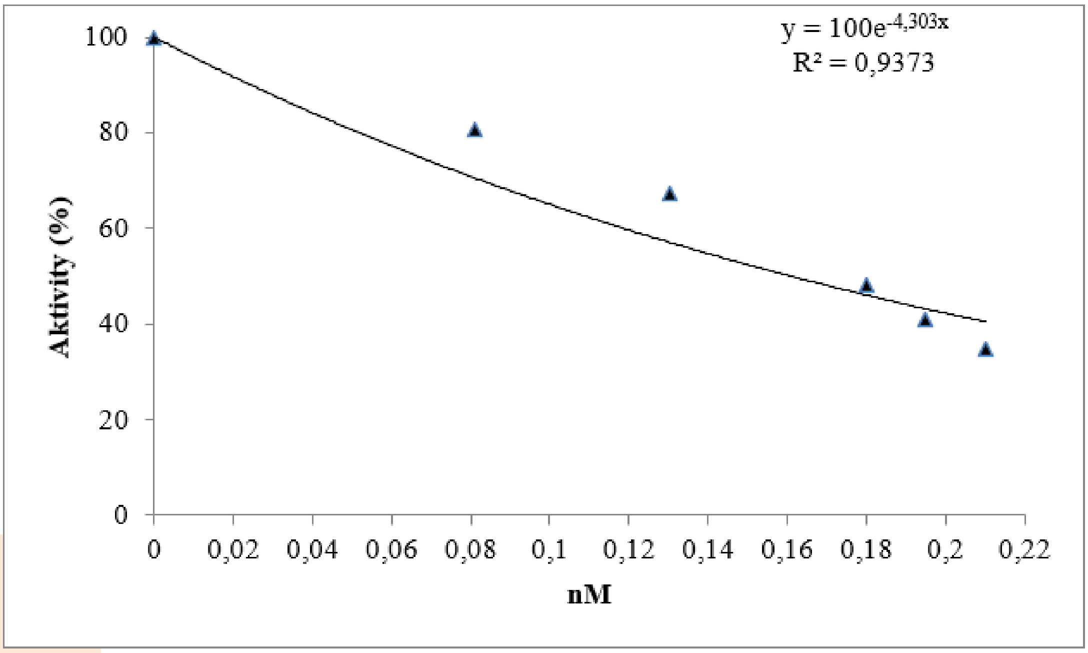

Figure S 118. Inhibition Curve of Compound **5d**

0	0.041522	0.072538	0.120406	0.130507	0.140558
100	76	65	52	40	34

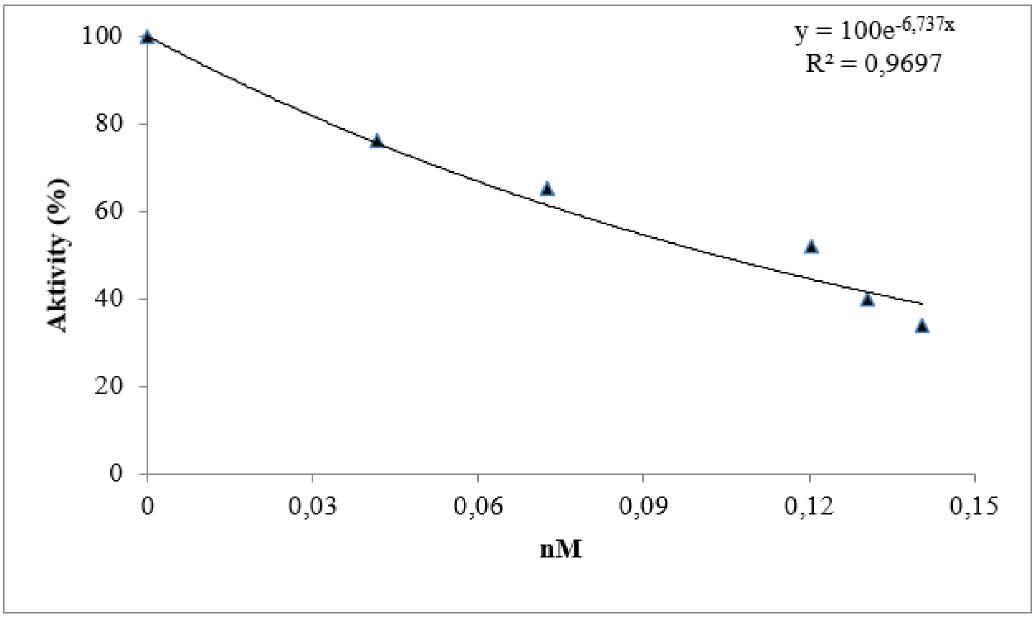

Figure S 119. Inhibition Curve of Compound **5e**

0	0.03127	0.071776	0.140317	0.160406	0.174568
100	78.33	65.83	50	44.16	38.33

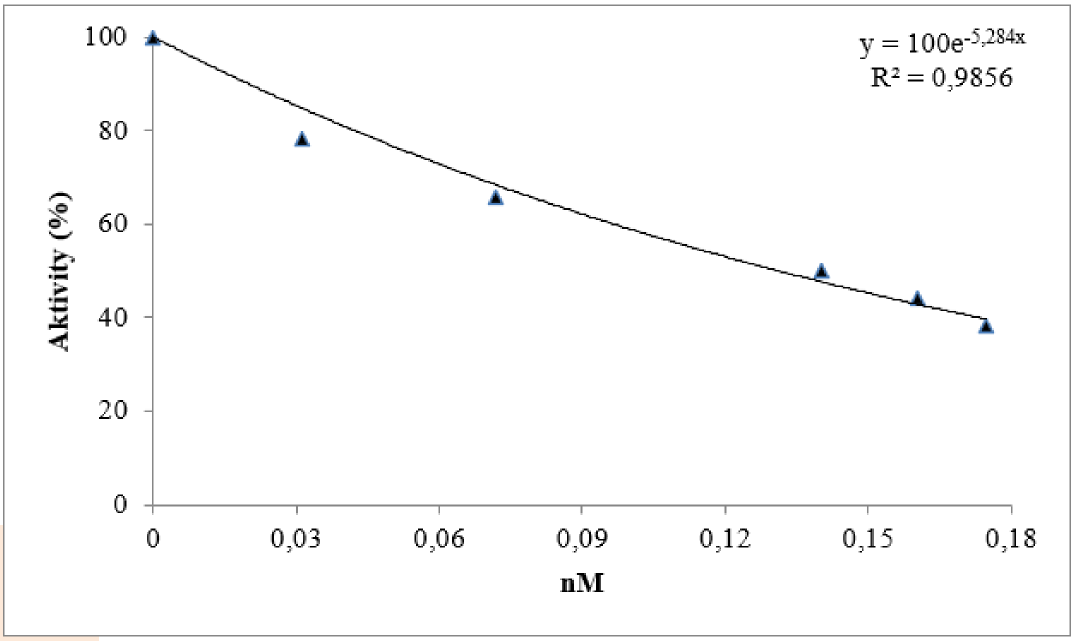

Figure S 120. Inhibition Curve of Compound **5f**

0	0.07906	0.130277	0.190451	0.210519	0.230624
100	72.5	63.33	48.33	41.66	36.66

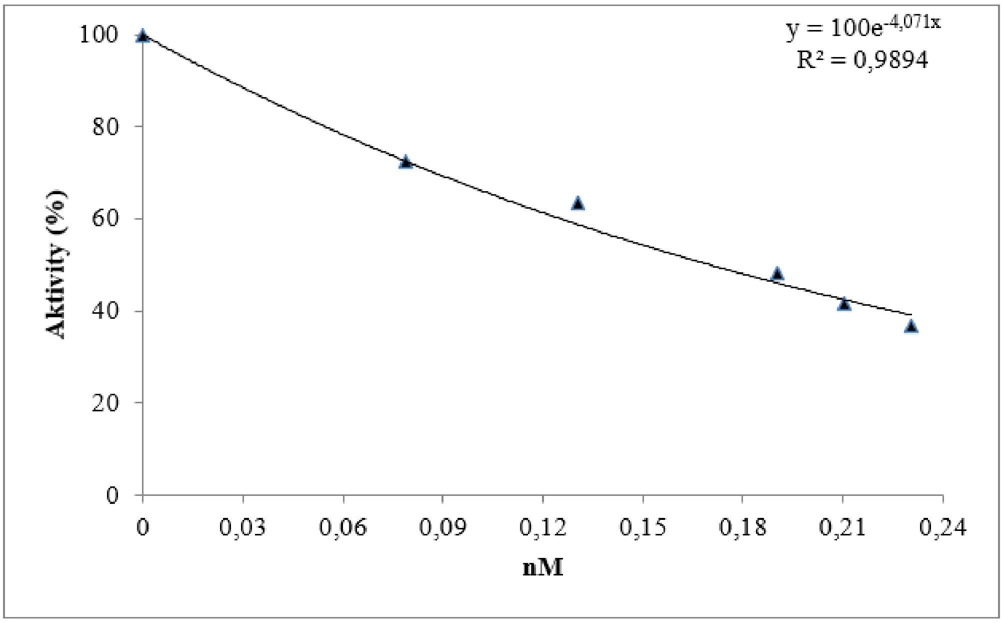

Figure S 121. Inhibition Curve of Compound **5g**

0	0.031317	0.051976	0.103624	0.110461	0.124056
100	75.55	66.66	48.88	44.44	37.77

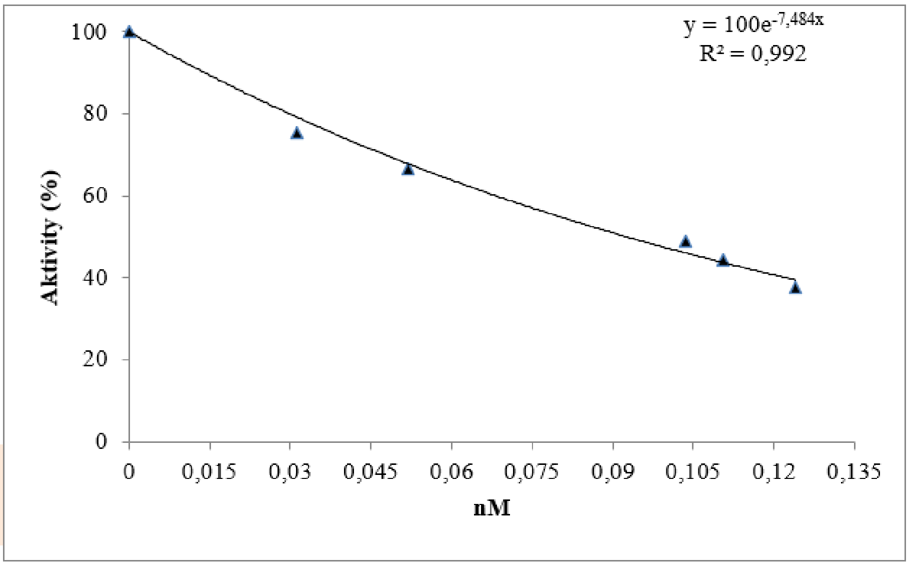

Figure S 122. Inhibition Curve of Compound **5h**

0	0.041317	0.072306	0.124041	0.140461	0.155053
100	72.38	57.14	48.57	38.09	33.33

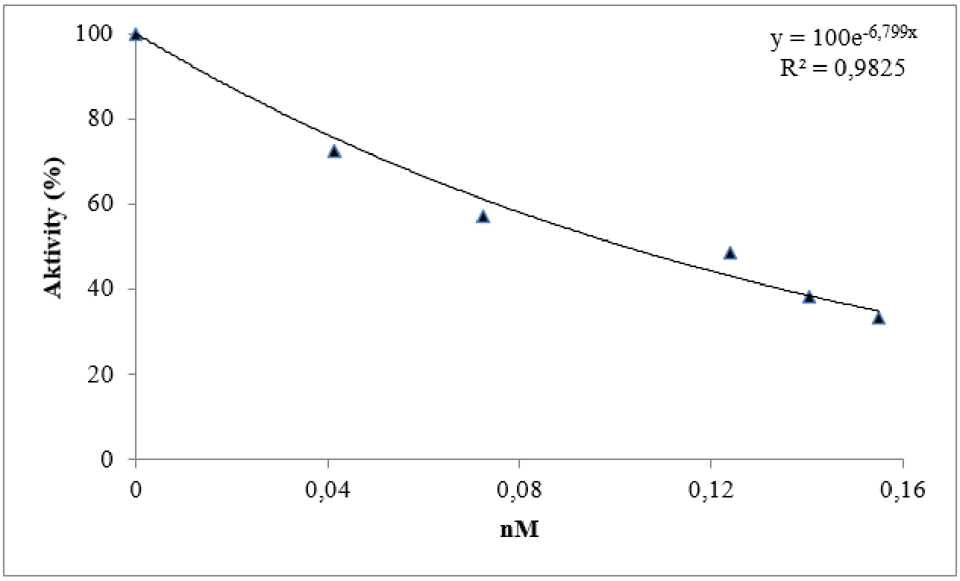

Figure S 123. Inhibition Curve of Compound **5i**

0	0.051812	0.082635	0.139048	0.150527	0.166061
100	76.2	64.76	48.57	40	33.33

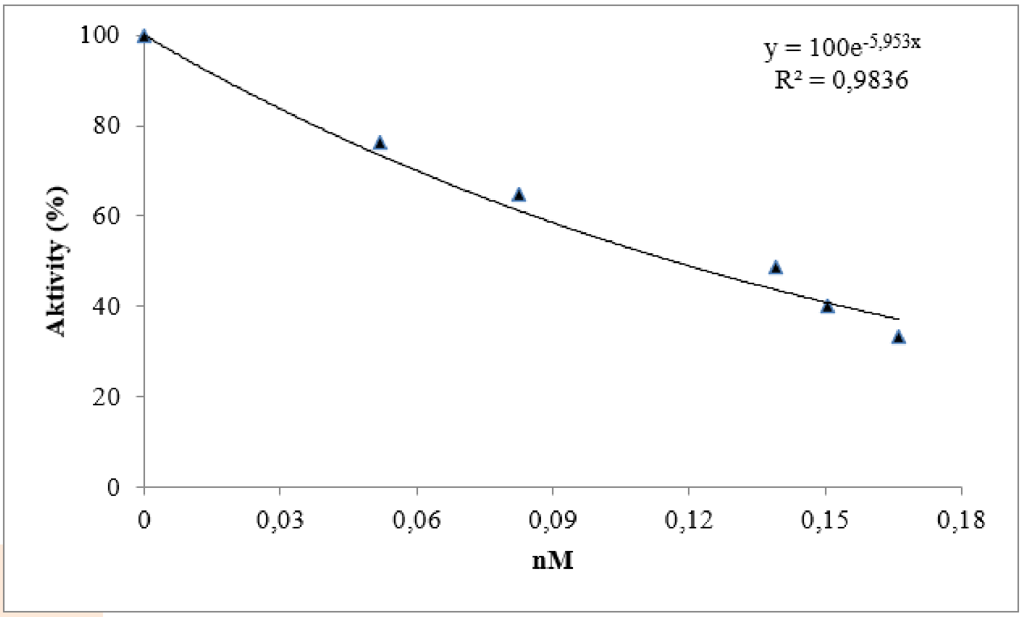

Figure S 124. Inhibition Curve of Compound **5j**

0	0.061497	0.092694	0.149045	0.160538	0.170598
100	77.77	67.77	47.77	41.11	32.22

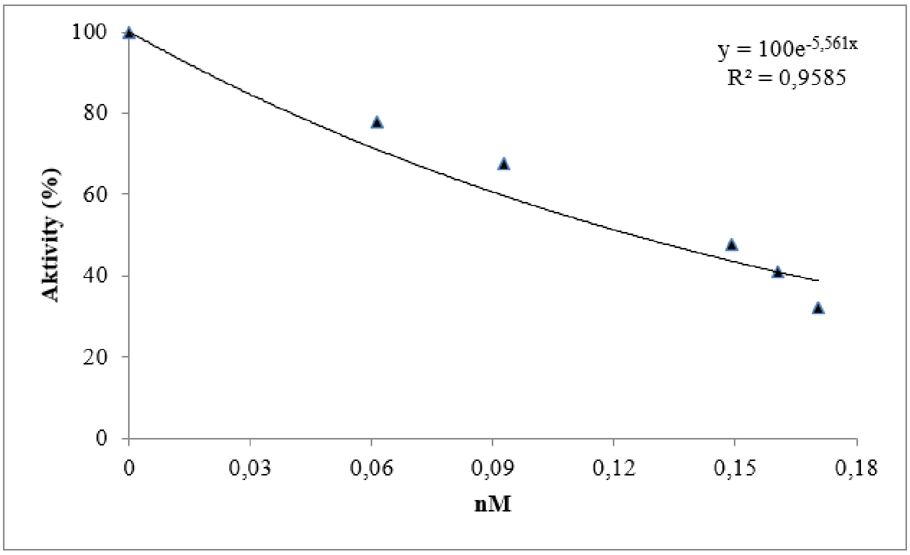

Figure S 125. Inhibition Curve of Compound **5k**

0	0.031644	0.061973	0.115036	0.124049	0.135056
100	71.11	63.33	50	41.11	35.55

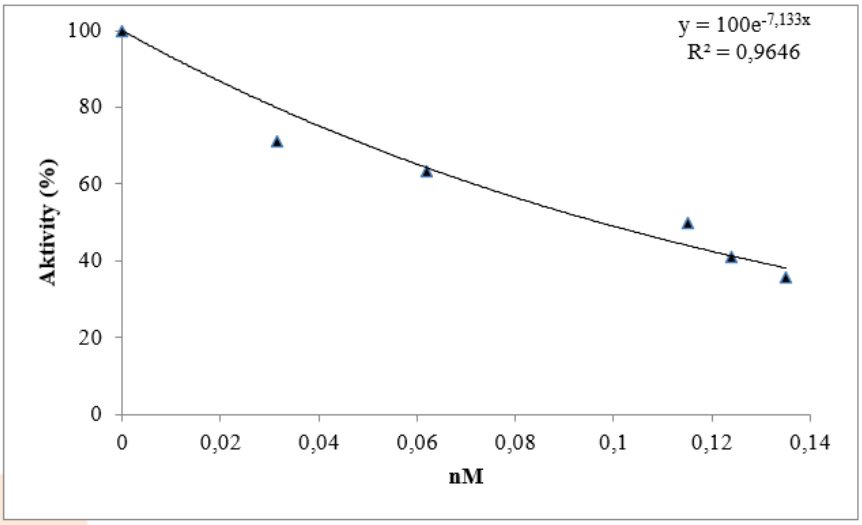

Figure S 126. Inhibition Curve of Compound **5l**

0	0.041506	0.082711	0.140407	0.150422	0.166051
100	76.66	60	48.88	44.44	38.88

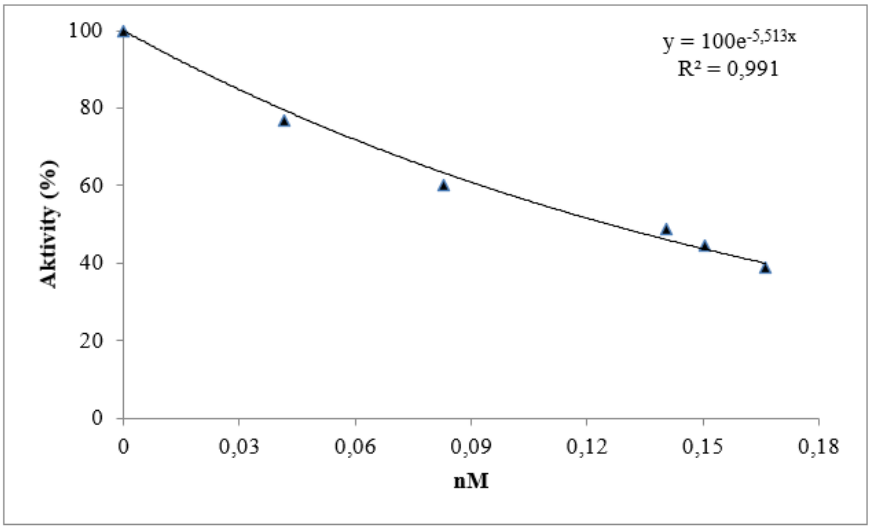

Figure S 127. Inhibition Curve of Compound **5m**

0	0.051257	0.0822	0.140394	0.154044	0.160503
100	74.46	64.89	48.93	42.55	34.04

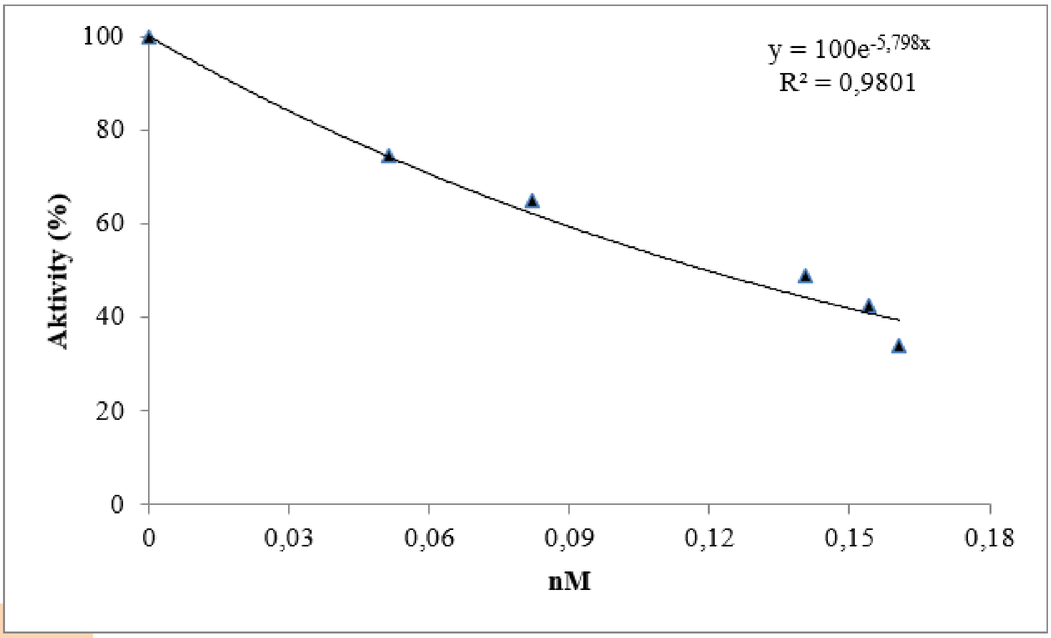

Figure S 128. Inhibition Curve of Compound **5n**

0	0.041256	0.071758	0.125028	0.140302	0.150377
100	72	60	50	45	35

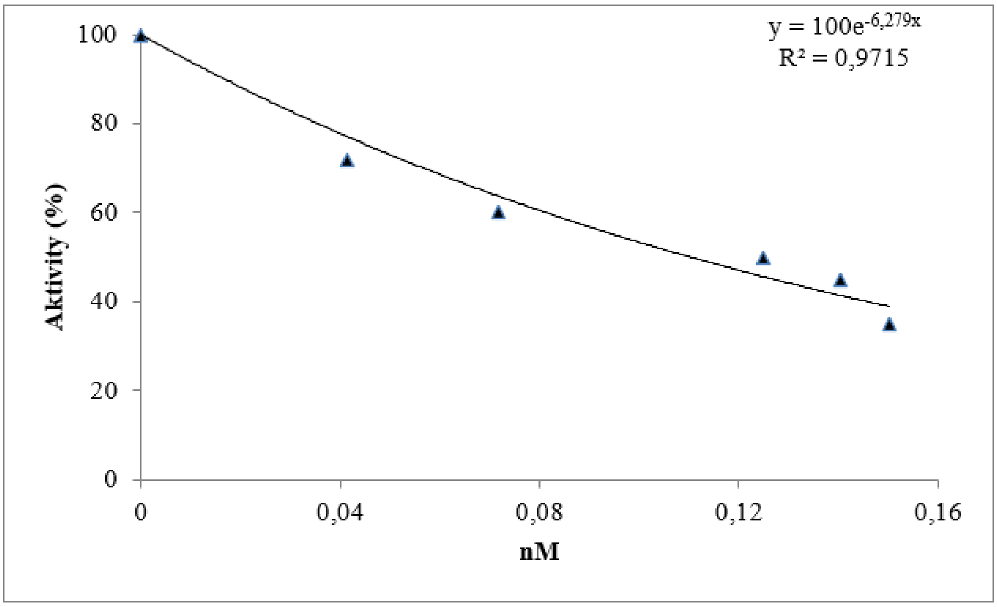

Figure S 129. Inhibition Curve of Compound **5o**

0	0.051716	0.093004	0.146558	0.156866	0.177725
100	80	72	47	40	34

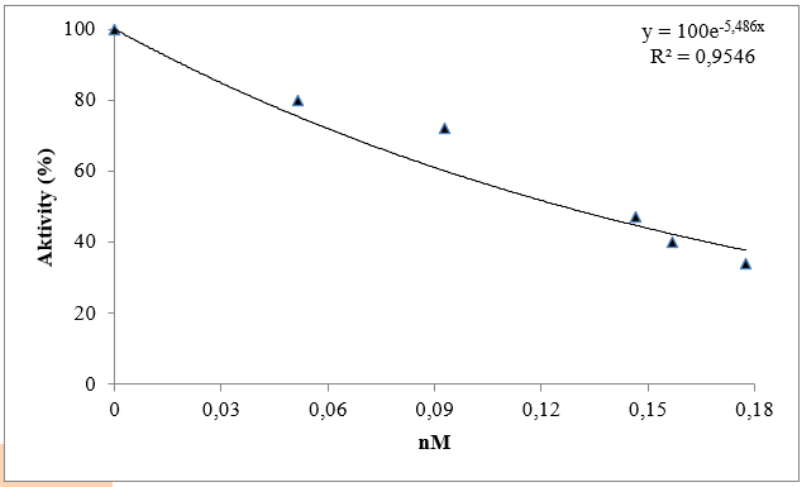

Figure S 130. Inhibition Curve of Compound **5p**

0	0.031087	0.061739	0.123043	0.130391	0.144347
100	75.83	66.66	50	44.16	39.16

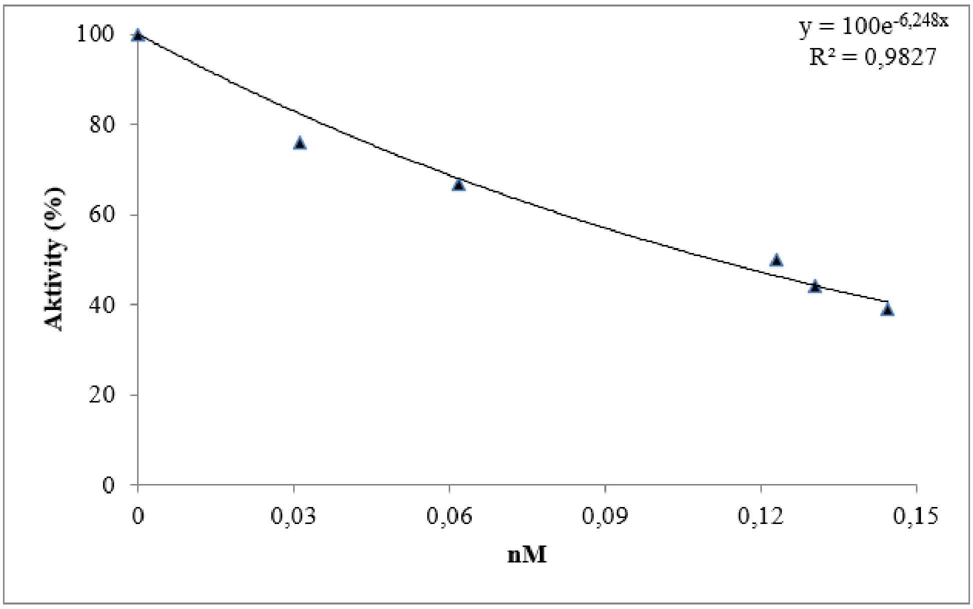

Figure S 131. Inhibition Curve of Compound **5q**

0	0.061121	0.091793	0.145291	0.157314	0.173811
100	73.07	65.38	47.7	45.38	38.46

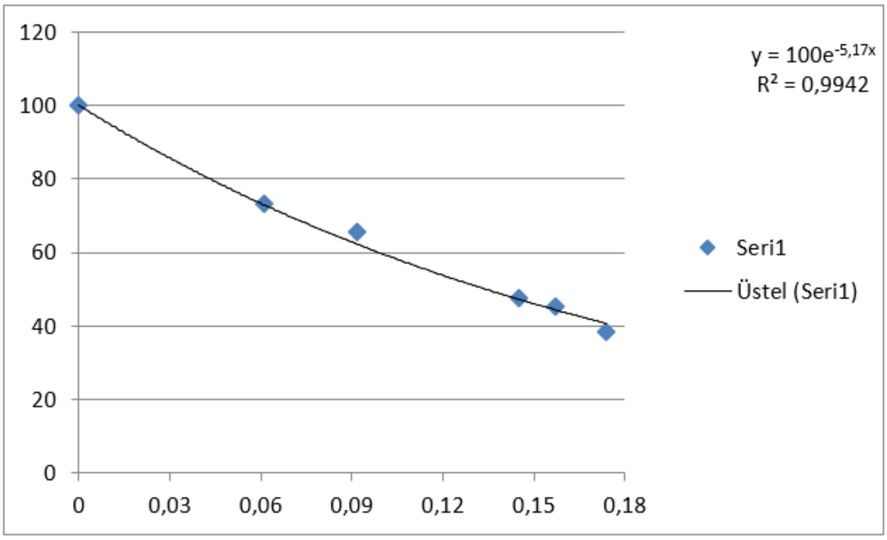

Figure S 132. Inhibition Curve of Compound **5r**

0	0.041674	0.062392	0.133468	0.144067	0.152478
100	70.4	60.8	48.81	42.4	36

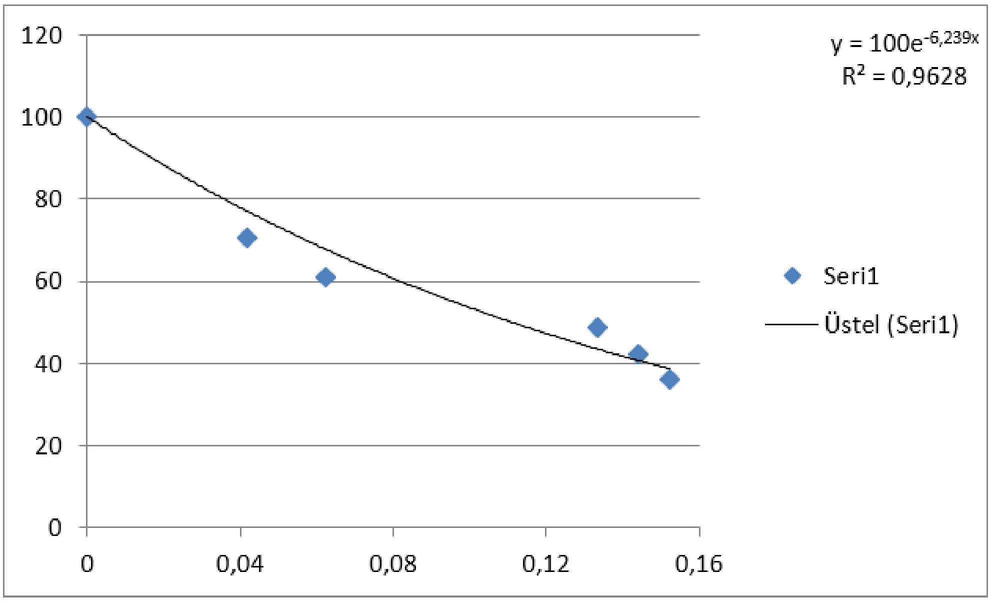

Figure S 133. Inhibition Curve of Compound **5s**

0	0.051674	0.10287	0.143827	0.154545	0.165023
100	70.83	60	47.55	41.66	37.5

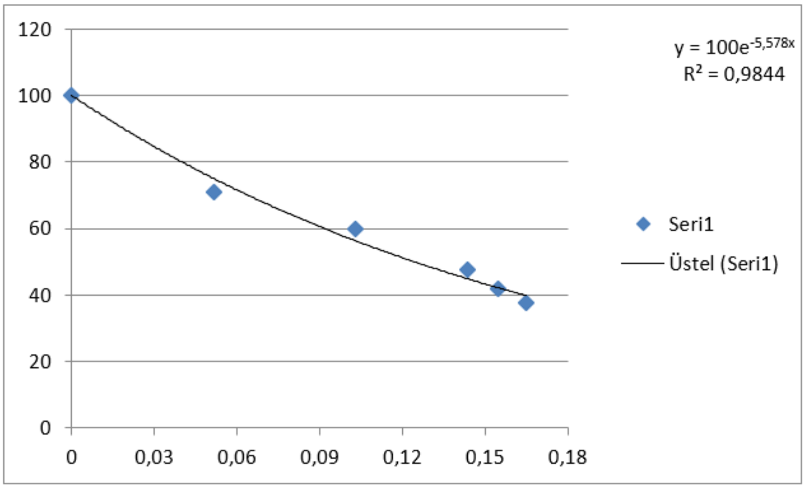

Figure S 134. Inhibition Curve of Compound **5t**

0	0.031054	0.051687	0.098253	0.123375	0.143692
100	76.33	67.93	49.61	43.51	38.16

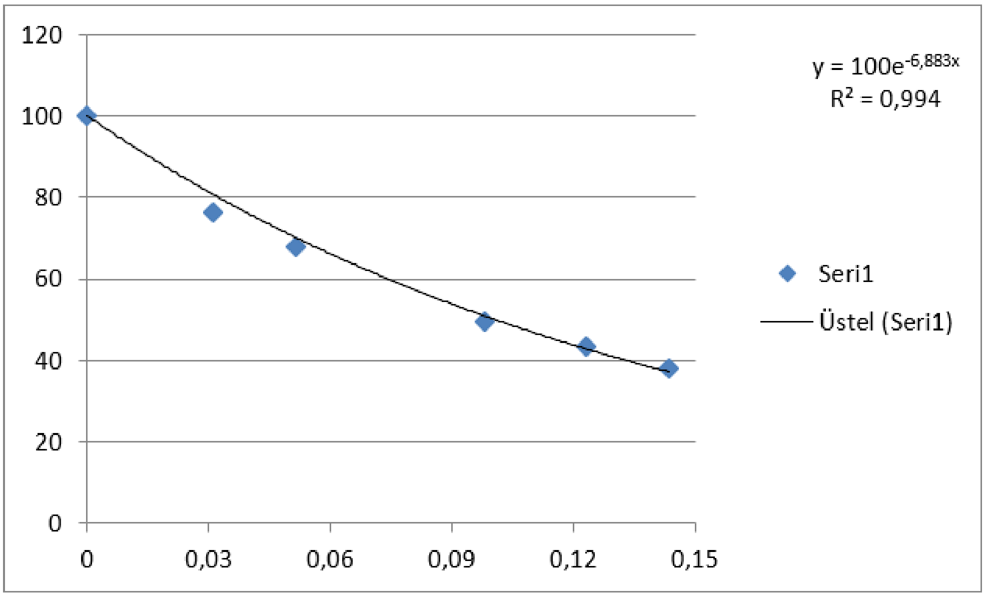

Figure S 135. Inhibition Curve of Compound **5u**

0	0.16237	0.348198	0.473465	0.494207	0.514702
100	72.32	66.07	50	43.75	35.71

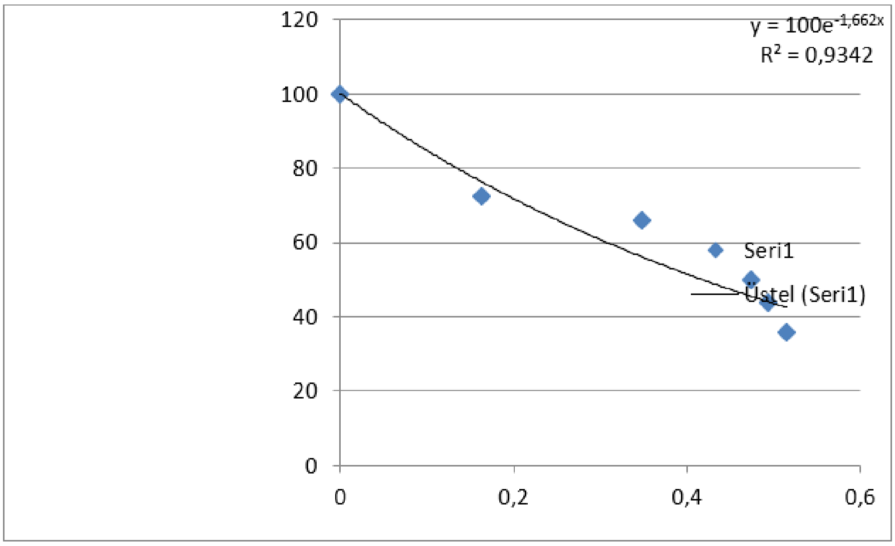

Figure S 136. Inhibition Curve of Compound **Clorgyline**
